# Supplementary material for: Functional space analyses reveal the function and evolution of the most bizarre theropod manual unguals
Source: Commun Biol. 2023 Feb 16;6:181. doi: 10.1038/s42003-023-04552-4 (PMC9935540; doi:10.1038/s42003-023-04552-4)
Supplement: Supplementary file 2 — Supplementary Materials [file 42003_2023_4552_MOESM2_ESM.pdf]

Supplementary materials to:

Functional space analyses reveal the function and evolution of the most bizarre theropod manual unguals

Zichuan Qin[1]*, Chun-Chi Liao[2], Michael J. Benton[1]* and Emily J. Rayfield[1]*

This PDF file includes:

Figs. S1 to S8
Tables S1 to S2
References (1 to 10)

Other Supplementary Materials for this manuscript include the following:
Data S1 to S2

Fig. S1.

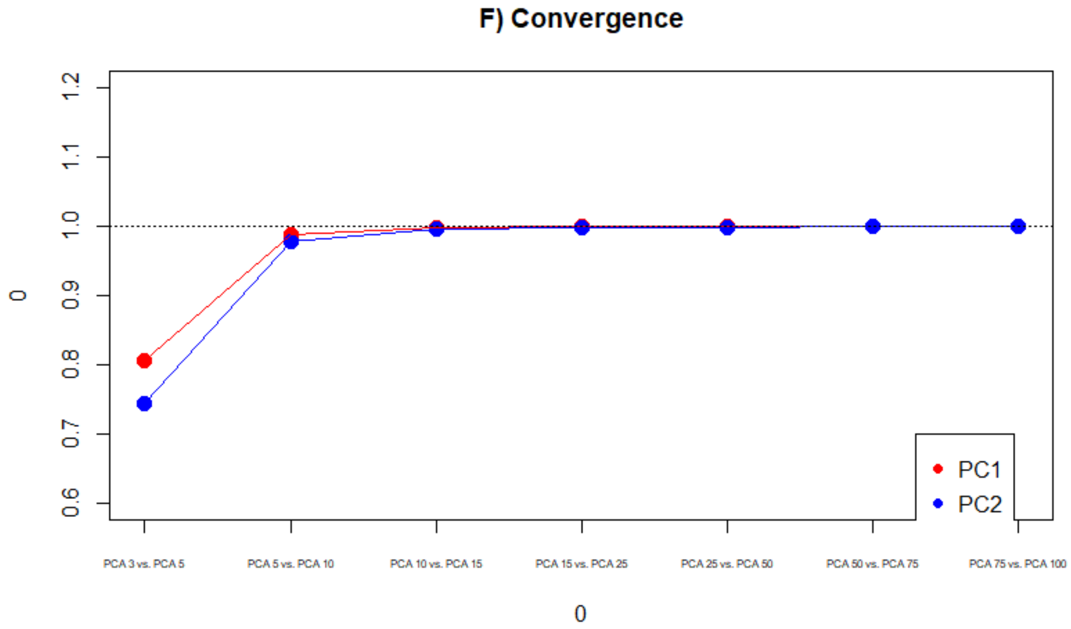

Consistency analysis of interval choice under the simulation simulations of 200 N piercing, showing high convergence (close to 1) when intervals over 50.

Fig. S2.

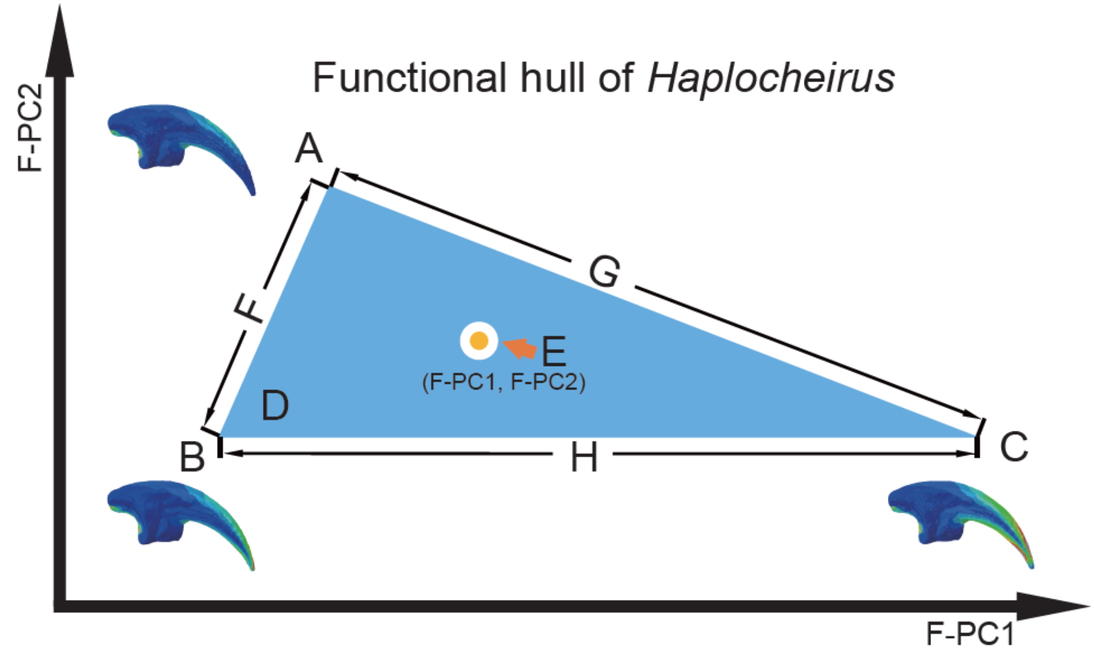

Fig. S3.

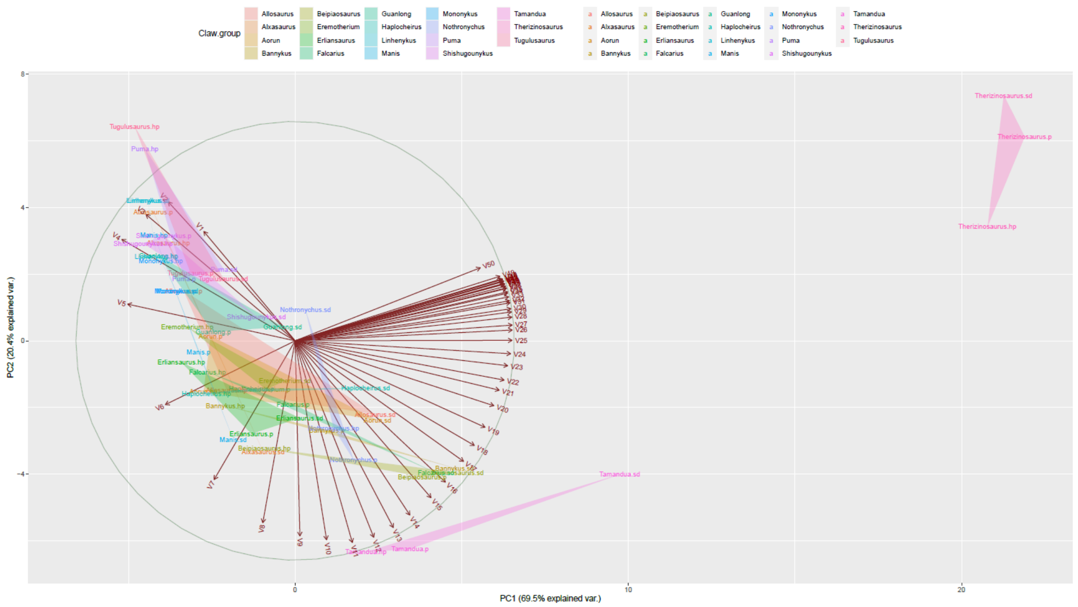

FSA shows our simulation results as functional hulls in a PCA plot. Functional triangles of different species are labelled by color. Arrows in the central part represent the overall stress levels.

Fig. S4.

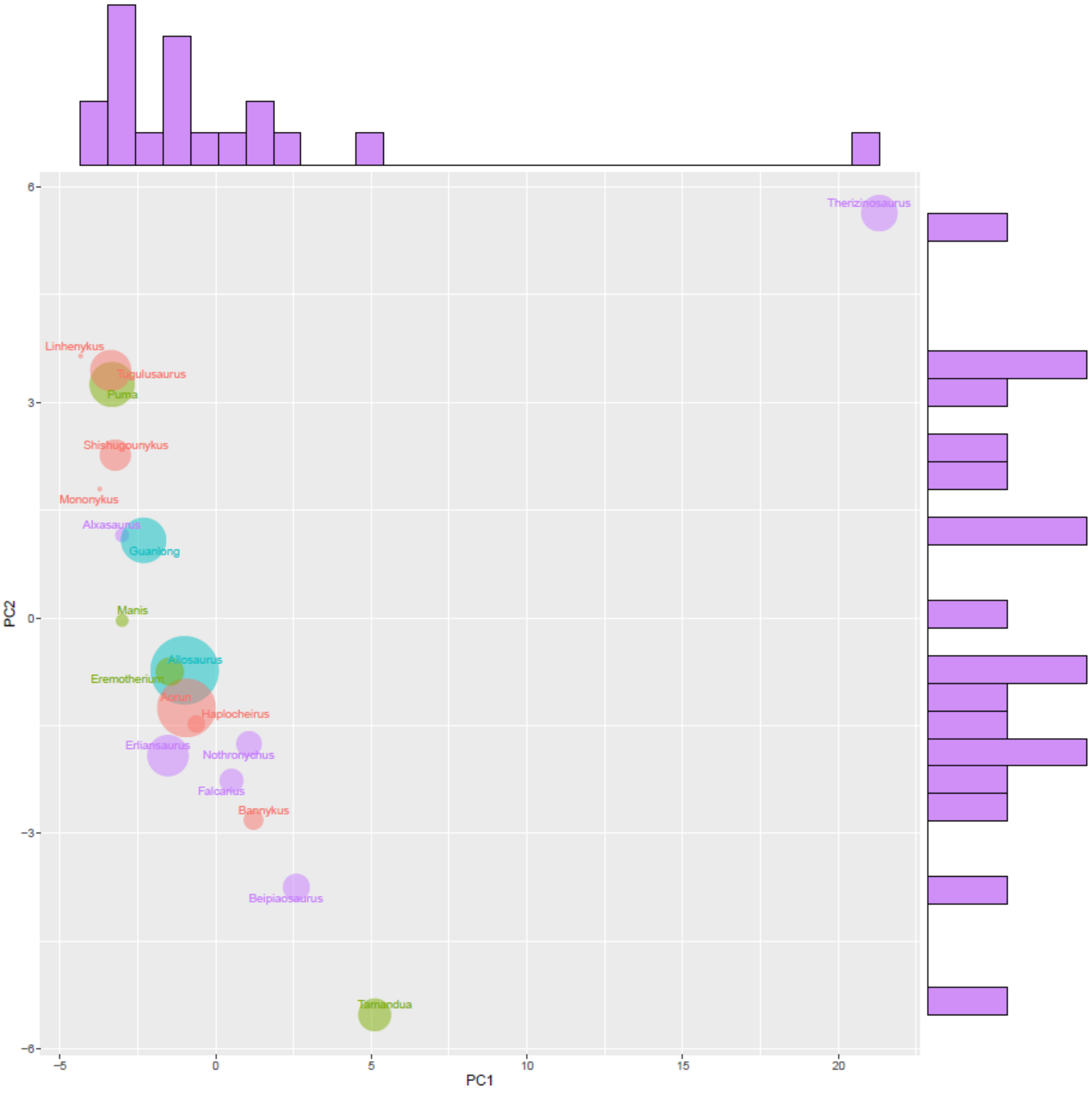

The centroids show the coordinate position of each functional triangle in Figure S1; the dot sizes represent the area of functional hulls; the column charts on borders show their distribution along PC1 and PC2. Dots of different species are labelled by clade, alvarezsauroids are red, therizinosaurians are purple, non-maniraptorans are blue and mammals are green.

Fig. S5.

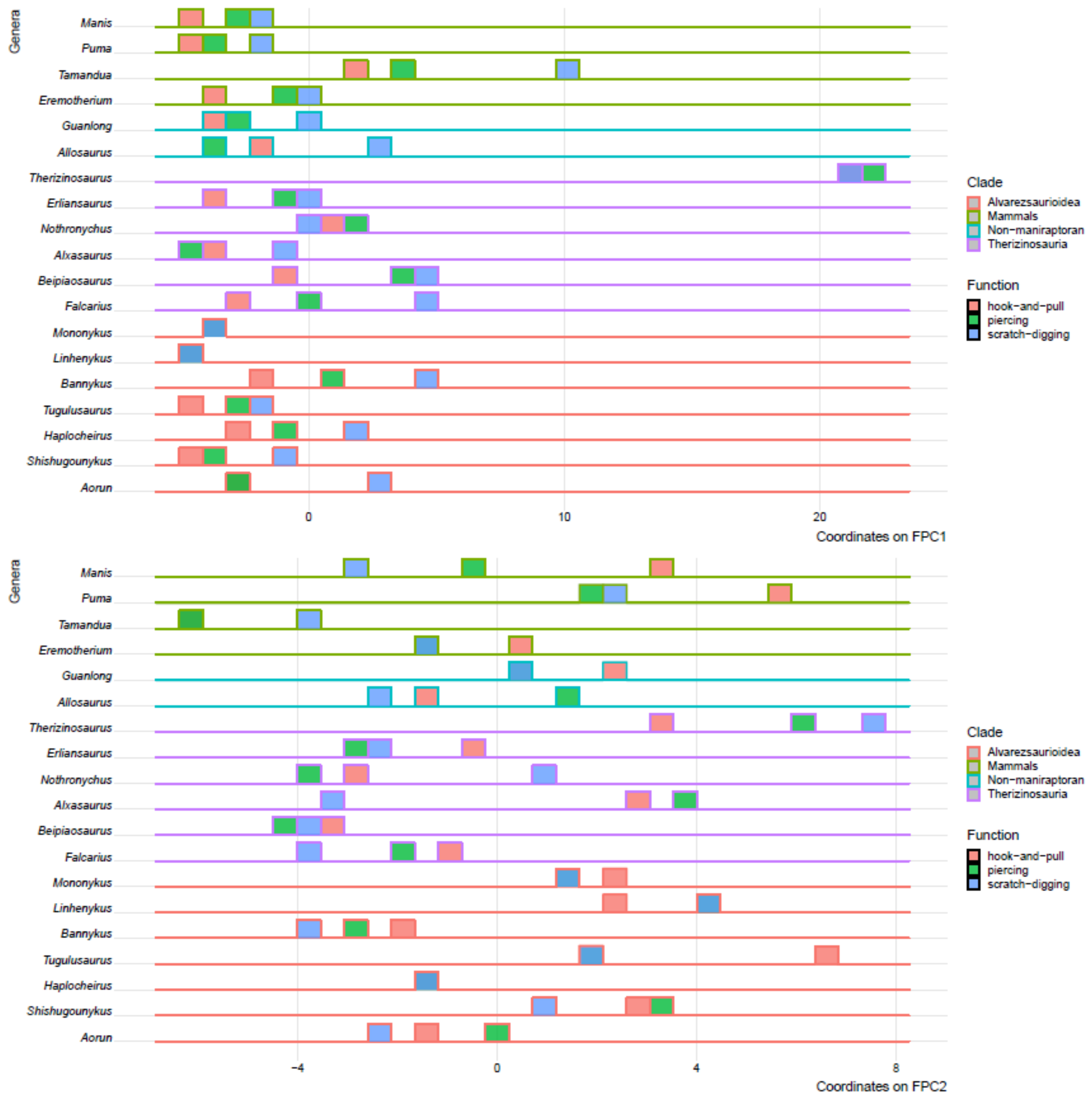

Functional performance in three simulations (hook-and-pull, piercing and scratch-digging), represent by each three simulation's result coordinates in PC1. Outlines of different species are labelled by clade, alvarezsauroids are red, therizinosaurians are purple, non-maniraptorans are blue and mammals are green. Functional scenarios are labelled by filled colour, hook-and-pull is red, piercing is green and scratch-digging is blue.

Fig. S6.

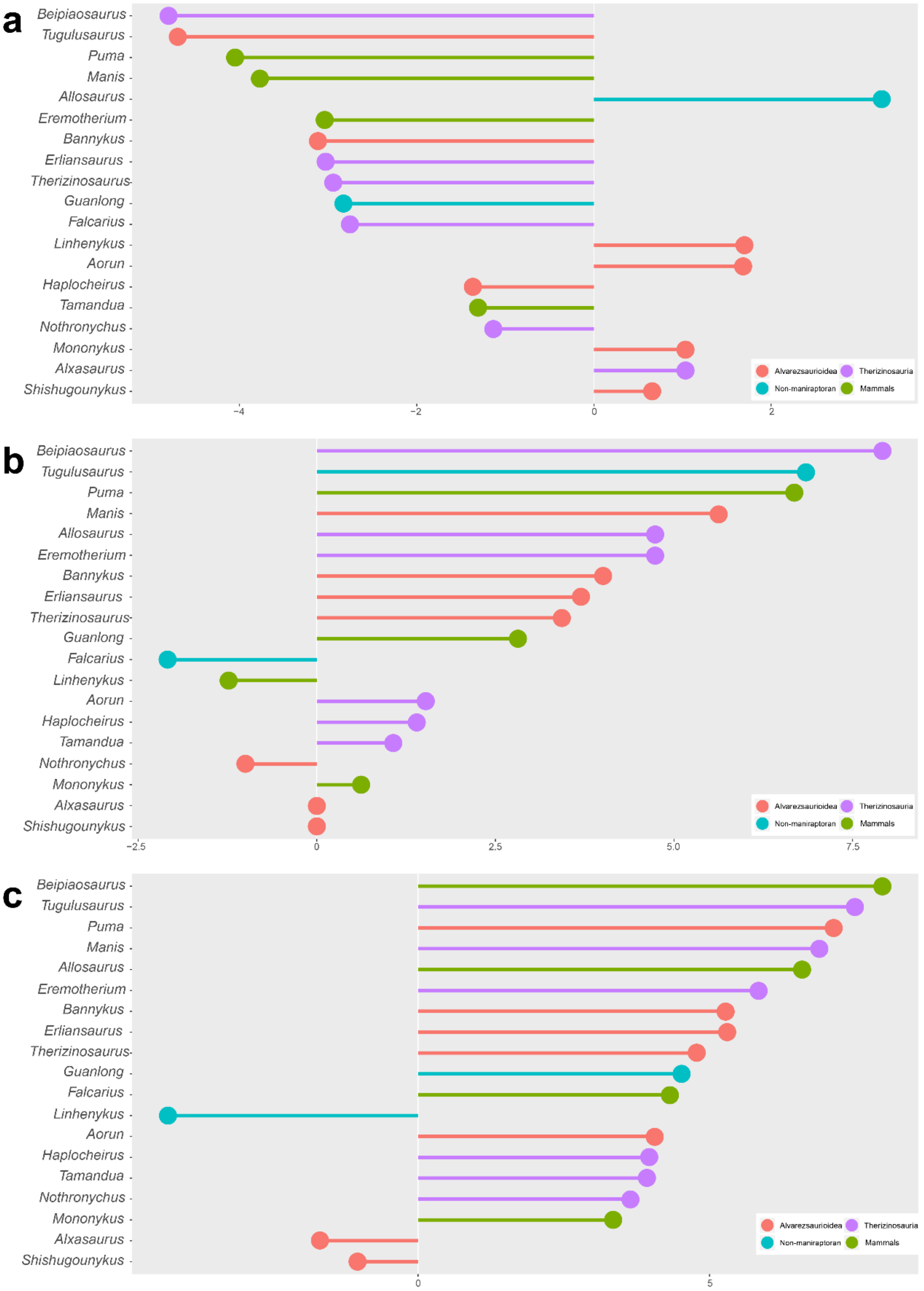

Quantified manual ungual functional divergence and overall functional performance estimation. Functional divergence between hook-and-pull and piercing (a), scratch-digging and piercing (b), scratch-digging and hook-and-pull (c).

Fig. S7.

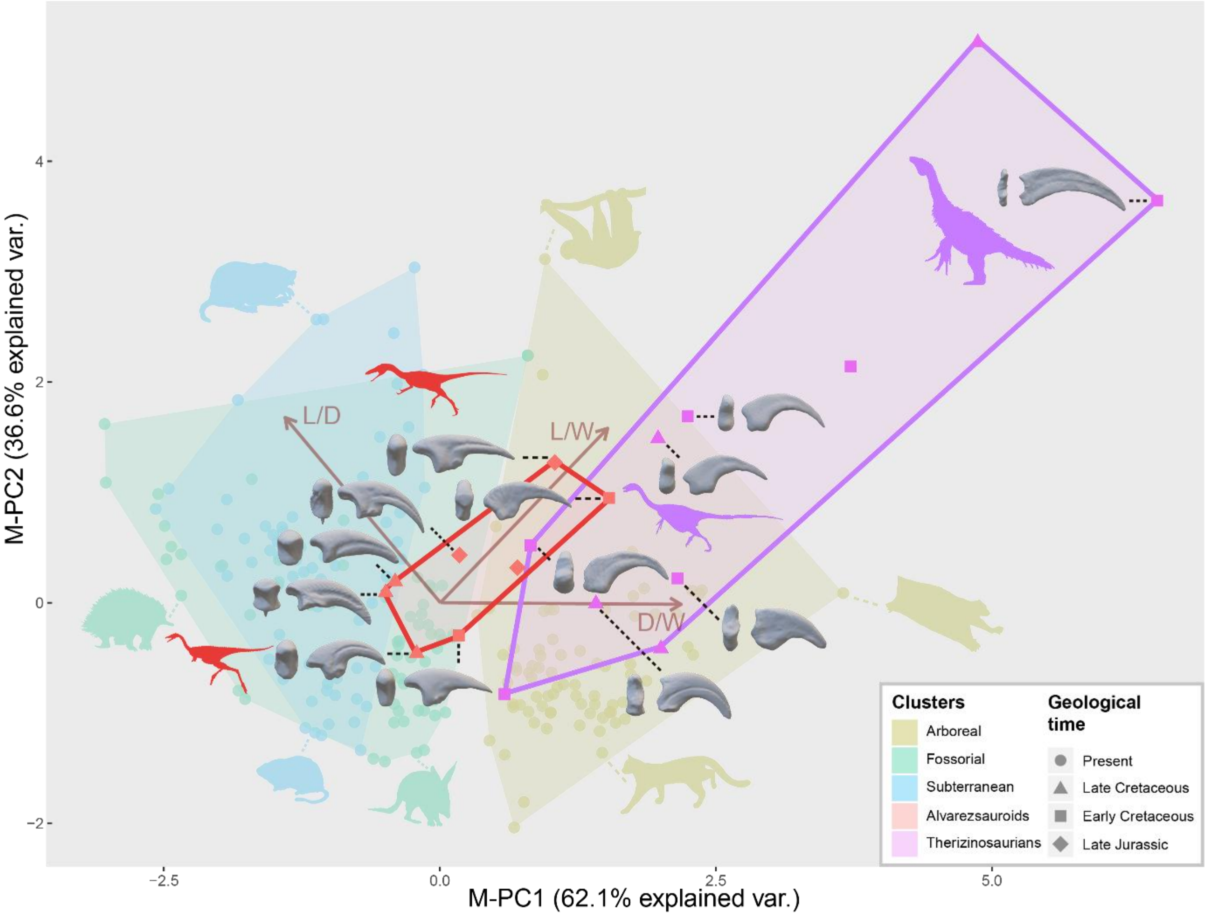

Quantized morphological divergences of three-dimensional manual ungual shapes, based on the first two principal component axes for the combined dataset(Jenkins et al., 2020). The D/W represent the ratio of depth to width, the L/W represent the ratio of length to width, and the L/D represent the ratio of length to depth.

Fig. S8.

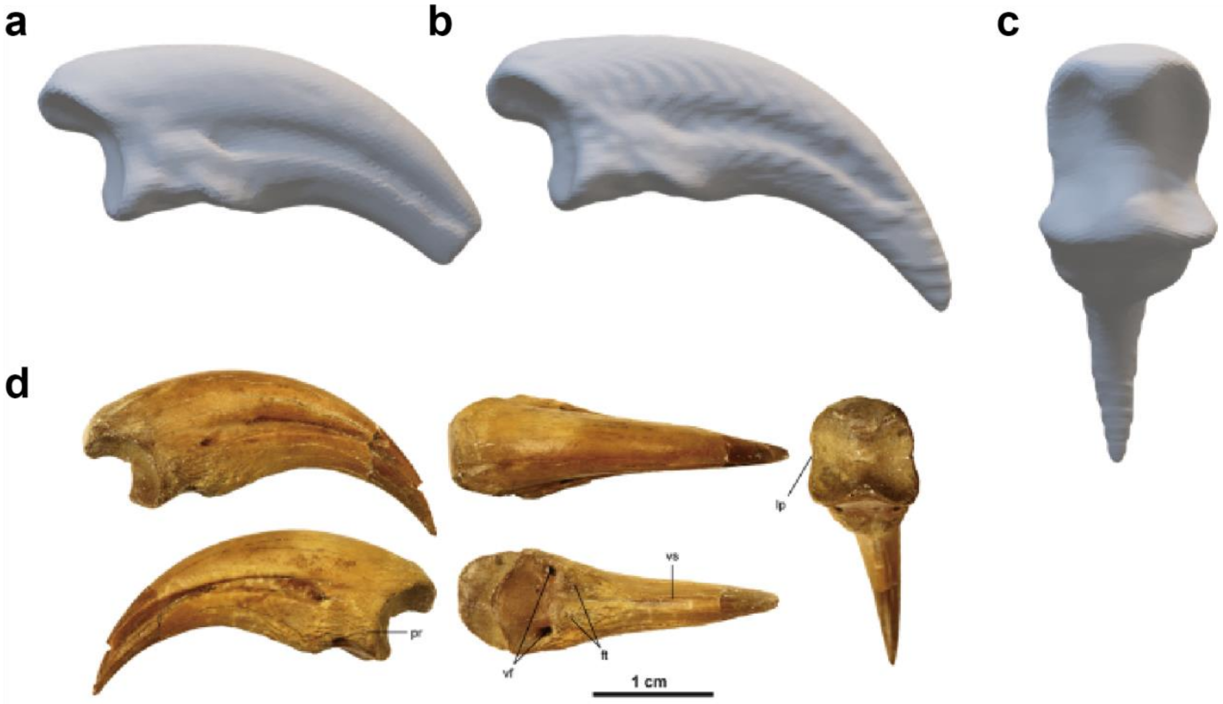

Reconstruction of pointy tip of uncomplete original claw models of *Mononykus* (a), and the new model in lateral view (b) and proximal view (c), referencing the only late-branching alvarezsauroids *Trierarchunchus* with sharp pointed tips (d) (Fowler et al., 2020).

Table S1.

Software and algorithms

**Abaqus 6.141**	Dassault Systemes Simulia Corp.	https://www.3ds.com/productsservices/ simulia/products/abaqus/
**Avizo 2021.1**	Thermo Fisher Scientific	https://www.thermofisher.com/hk/en/ home/electron-microscopy/products/ software-em-3d-vis/avizo-software.html
**Blender 2.79b**	Blender Foundation	https://www.blender.org/
**Fiji**	(Schindelin et al., 2012)	https://imagej.net/software/fiji/
**R 4.1.1**	(Team, 2015)	https://cran.r-project.org

Table S2.

Data resources

Species	Specimen No.	3D models	3D measurements	Data type
**alvarezsauroids**				
Aorun zhaoi	IVPP V15709 (IVPP)	Yes	Yes	3D model(Qin et al., 2019)
Bannykus wulatensis	IVPP V25026 (IVPP)	Yes	Yes	3D model(Qin et al., 2019)
Haplocheirus sollers	IVPP V15988 (IVPP)	Yes	Yes	3D model(Qin et al., 2019)
Linhenykus monodactylus	IVPP V17608 (IVPP)	Yes (repaired pointy tip)	Yes	3D model(Qin et al., 2019)
Mononykus olecranus	AMNH FARB 28508 (American Museum of Natural History)	Yes (repaired pointy tip)	Yes	3D model from Mark Norell and Congyu Yu
Shishugounykus inexpectus	IVPP V23567 (IVPP)	Yes	Yes	3D model(Qin et al., 2019)
Albertonykus borealis	TMP 2000.45.86 (Royal Tyrrell Museum of Palaeontology)	-	Yes	(Longrich & Currie, 2009)
Tugulusaurus faciles	IVPP V4025 (IVPP)	Yes	Yes	CT scanning
**therizinosaurians**				
Alxasaurus elesitaiensis	IVPP 88402 (IVPP)	Yes	Yes	3D model(Lautenschlager, 2014)
Beipiaosaurus inexpectus	IVPP V11559 (IVPP)	Yes	Yes	3D model(Lautenschlager, 2014)
Erliansaurus bellamanus	LH V 0002 (Long Hao Geologic and Paleontological Research Center)	Yes	Yes	surface scanning
Falcarius utahensis	UMNH VP 12320 (Natural History Museum of Utah)	Yes	Yes	3D model(Lautenschlager, 2014)
Nothronychus graffami	UMNH V16420 (Natural History Museum of Utah)	Yes	Yes	3D model(Lautenschlager, 2014)
Therizinosaurus cheloniformis	IGM 100/17 (Geological Institute of the Mongolian Academy of Sciences)<br><br>Mongolia)	Yes	Yes	3D model(Lautenschlager, 2014)

Martharaptor greenriverensis	UMNH VP 21400 (Natural History Museum of Utah)	-	Yes	(Senter et al., 2012)
IVPP new specimen	IVPP V 18957 (IVPP)	-	Yes	Personal observations
Jianchangosaurus yixianensis	41HIII-0308A (Henan Geological Museum)	-	Yes	(Yao et al., 2019)
Bissekty therizinosauroids	ZIN PH 19/16 (Paleoherpetological Collection, Zoological Institute, Russian Academy)	-	Yes	(Sues & Averianov, 2016)
**non-maniraptorans**				
Allosaurus fragilis	MOR 693 (Wyoming Geological Museum)	Yes	Yes	Sketchfab
Guanlong wucaii	IVPP V14531 (IVPP)	Yes	Yes	3D model(Qin et al., 2019)
**mammals**				
Eremotherium laurillardi	ChM-PV4803 (The Charleston Museum)	Yes	Yes	Sketchfab
Manis tricuspis	CZA- 7104 (Chicago Zoological Society)	Yes	Yes	Sketchfab
Puma concolor	IMNH R-2372 (Idaho Museum of Natural History)	Yes	Yes	Sketchfab
Tamandua tetradactyla	IMNH R-997 (Idaho Museum of Natural History)	Yes	Yes	Sketchfab

Table S3.

Regression and histogram dataset

Taxon	LM (g)	LV (cm^3)	L/W	L/D	D/W	M-PC1	M-PC2	F-PC1	F-PC2	RC/M
Aorun	3.10	-0.98	5.88	2.68	2.19	1.04	1.28	-0.93	-1.25	0.27
Linhenykus	3.30	-1.04	3.75	2.61	1.44	-0.44	0.17	-4.33	3.65	0.47
Mononykus	3.67	-0.03	3.63	2.60	1.40	-0.51	0.10	-3.72	1.79	0.46
Shishugounykus	3.78	0.09	4.67	2.28	2.05	0.70	0.31	-3.22	2.27	0.23
Tugulusaurus	4.06	1.07	3.71	2.12	1.75	0.16	-0.31	-3.37	3.44	0.43
Haplocheirus	4.31	0.57	4.41	2.53	1.74	0.16	0.43	-0.62	-1.48	0.30
Bannykus	4.46	0.72	5.85	2.34	2.50	1.54	0.95	1.22	-2.81	0.47
Alxasaurus	5.55	1.73	5.39	1.82	2.97	2.15	0.22	-3.00	1.15	
Beipiaosaurus	4.44	1.31	4.96	2.35	2.11	0.82	0.52	2.59	-3.75	
Erliansaurus	5.14	1.17	4.78	1.92	2.49	1.40	0.02	-1.53	-1.92	
Falcarius	5.11	1.18	7.08	2.49	2.85	2.25	1.69	0.51	-2.27	
Nothronychus	5.83	2.28	6.74	2.49	2.70	1.97	1.53	1.07	-1.75	
Therizinosaurus	6.82	2.76	12.60	3.21	3.92	4.87	5.10	21.31	5.63	

LM (g) =Log transformed body mass (g); LV (cm^3) =Log transformed ungual volume (cm^3); L/W=ratio of length to width; L/D=ratio of length to depth; D/W=ratio of depth to width; M-PC1=Morphological principal component 1; M-PC2=Morphological principal component 2; F-PC1=Functional principal component 1; F-PC2= Functional principal component 2; RC/M=ratio of claw length to manus length.

Table S4.

Regression formulas and parameters

**Therizinosaurians**			
	**formula**	**R^2**	**p-value**
Morphological PC1 VS Log-Body mass	$y = -2.302 + 0.7374x$	0.82	0.013*
Log-Volume VS Log-Body mass	$y = -6.4526 + 1.5868x$	0.8491	0.009**
**Alvarezsauroids**			
	**formula**	**R^2**	**p-value**
Functional PC1 VS Log-Body mass	$y = -10.01 + 2.09x$	0.26	0.238
Morphological PC1 VS Log-Body mass	$y = -1.28 + 0.434x$	0.08	0.531
Log-Volume VS Log-Body mass	$y = -5.57 + 1.48x$	0.83	0.005**
Functional PC1 VS Morphological PC1	$y = -2.96 + 2.17x$	0.65	0.029*
Morphological PC1 VS Log-Volume	$y = 0.369 + 0.183x$	0.04	0.671
Functional PC1PC1 VS Log-Volume	$y = -2.18 + 0.755x$	0.09	0.510
Ratio of Claw/Manus VS Geo-time	$y = 0.663 + 0.00219x$	0.70	0.019*

References

Fowler, D. W., Wilson, J. P., Fowler, E. A. F., Noto, C. R., Anduza, D., & Horner, J. R. (2020). Trierarchuncus prairiensis gen. et sp. nov., the last alvarezsaurid: Hell Creek Formation (uppermost Maastrichtian), Montana. *Cretaceous Research*, *116*, 104560.

Jenkins, X. A., Pritchard, A. C., Marsh, A. D., Kligman, B. T., Sidor, C. A., & Reed, K. E. (2020). Using manual ungual morphology to predict substrate use in the Drepanosauromorpha and the description of a new species. *Journal of Vertebrate Paleontology*, *40*(5), e1810058.

Lautenschlager, S. (2014). Morphological and functional diversity in therizinosaur claws and the implications for theropod claw evolution. *proceedings of the Royal Society Biological Science*, *281*(1785), 20140497.

Longrich, N. R., & Currie, P. J. (2009). *Albertonykus borealis*, a new alvarezsaur (Dinosauria: Theropoda) from the Early Maastrichtian of Alberta, Canada: implications for the systematics and ecology of the Alvarezsauridae. *Cretaceous Research*, *30*(1), 239-252.

Qin, Z., Clark, J., Choiniere, J., & Xu, X. (2019). A new alvarezsaurian theropod from the Upper Jurassic Shishugou Formation of western China. *Scientific Reports*, *9*(1), 11727. https://doi.org/10.1038/s41598-019-48148-7

Schindelin, J., Arganda-Carreras, I., Frise, E., Kaynig, V., Longair, M., Pietzsch, T., Preibisch, S., Rueden, C., Saalfeld, S., Schmid, B., Tinevez, J.-Y., White, D. J., Hartenstein, V., Eliceiri, K., Tomancak, P., & Cardona, A. (2012). Fiji: an open-source platform for biological-image analysis. *Nature methods*, *9*(7), 676-682. https://doi.org/10.1038/nmeth.2019

Senter, P., Kirkland, J. I., & Deblieux, D. D. (2012). Martharaptor greenriverensis, a New Theropod Dinosaur from the Lower Cretaceous of Utah. *PLoS ONE*, *7*(8), e43911. https://doi.org/10.1371/journal.pone.0043911

Sues, H.-D., & Averianov, A. (2016). Therizinosauroidea (Dinosauria: Theropoda) from the Upper Cretaceous of Uzbekistan. *Cretaceous Research*, *59*, 155-178. https://doi.org/10.1016/j.cretres.2015.11.003

Team, R. C. (2015). *R: A language and environment for statistical computing*. In R Foundation for Statistical Computing. https://www.R-project.org/

Yao, X., Liao, C.-C., Sullivan, C., & Xu, X. (2019). A new transitional therizinosaurian theropod from the Early Cretaceous Jehol Biota of China. *Scientific Reports*, *9*(1), 1-12.
