# Supplementary material for: Functional space analyses reveal the function and evolution of the most bizarre theropod manual unguals
Source: Commun Biol. 2023 Feb 16;6:181. doi: 10.1038/s42003-023-04552-4 (PMC9935540; doi:10.1038/s42003-023-04552-4)
Supplement: Supplementary file 3 — Description of Additional Supplementary Files [file 42003_2023_4552_MOESM3_ESM.pdf]

Description of Additional Supplementary Files

File name: Supplementary Data 1
Description: The code of how to apply 3D shape analyses.

File name: Supplementary Data 2
Description: The code of how to apply functional space analyses.
