# Supplementary material for: Functional space analyses reveal the function and evolution of the most bizarre theropod manual unguals
Source: Commun Biol. 2023 Feb 16;6:181. doi: 10.1038/s42003-023-04552-4 (PMC9935540; doi:10.1038/s42003-023-04552-4)
Supplement: Supplementary file 4 — Supplementary Data 1 [file 42003_2023_4552_MOESM4_ESM.zip › FSA file/FSA method_R_markdown.html]

Untitled


# Untitled

#### Zichuan Qin of UoB

#### 2022-02-22

# 1. Packages needed

These are the packages needed in Functional space analyses.

```
# Check whether necessary packages have been installed. If not, these will be installed...
if(!"usethis" %in% installed.packages()) install.packages("usethis")
if(!"devtools" %in% installed.packages()) install.packages("devtools")
if(!"ggplot2" %in% installed.packages()) install.packages("ggplot2")
if(!"plyr" %in% installed.packages()) install.packages("plyr")
if(!"scales" %in% installed.packages()) install.packages("scales")
if(!"grid" %in% installed.packages()) install.packages("grid")
if(!"ggrepel" %in% installed.packages()) install.packages("ggrepel")
if(!"ggbiplot" %in% installed.packages()) install.packages("ggbiplot")
if(!"stats" %in% installed.packages()) install.packages("stats")
if(!"dplyr" %in% installed.packages()) install.packages("dplyr")
if(!"gridExtra" %in% installed.packages()) install.packages("gridExtra")
if(!"svglite" %in% installed.packages()) install.packages("svglite")
if(!"ggthemr" %in% installed.packages()) install.packages("ggthemr")
if(!"tvthemes" %in% installed.packages()) install.packages("tvthemes")
if(!"ggExtra" %in% installed.packages()) install.packages("ggExtra")
if(!"ggridges" %in% installed.packages()) install.packages("ggridges")


# Make the packages available in your R session.
lapply(c("usethis","devtools","ggplot2","plyr","scales","grid","ggrepel","ggbiplot","stats","dplyr",
         "gridExtra","svglite","ggthemr","tvthemes","ggExtra","ggridges"),
       require,                 # Using require instead of library
       character.only = TRUE)   # apply concatenated strings to require function
```

```
## [[1]]
## [1] TRUE
## 
## [[2]]
## [1] TRUE
## 
## [[3]]
## [1] TRUE
## 
## [[4]]
## [1] TRUE
## 
## [[5]]
## [1] TRUE
## 
## [[6]]
## [1] TRUE
## 
## [[7]]
## [1] TRUE
## 
## [[8]]
## [1] TRUE
## 
## [[9]]
## [1] TRUE
## 
## [[10]]
## [1] TRUE
## 
## [[11]]
## [1] TRUE
## 
## [[12]]
## [1] TRUE
## 
## [[13]]
## [1] TRUE
## 
## [[14]]
## [1] TRUE
## 
## [[15]]
## [1] TRUE
## 
## [[16]]
## [1] TRUE
```

# 2.PCA of function data

## 2.1 Reading function data

```
Claw<-read.csv('matrix-of-intervals-50.csv',header = TRUE,row.names=1)
```

## 2.2 PCA of function data & Plot PCA

```
Claw.pca<-prcomp(Claw[,c(1:50)],center=TRUE,scale. = TRUE)
summary(Claw.pca)
```

```
## Importance of components:
##                           PC1    PC2     PC3     PC4     PC5     PC6     PC7
## Standard deviation     5.8957 3.1899 1.55779 1.05169 0.72518 0.57762 0.51753
## Proportion of Variance 0.6952 0.2035 0.04853 0.02212 0.01052 0.00667 0.00536
## Cumulative Proportion  0.6952 0.8987 0.94722 0.96935 0.97986 0.98654 0.99189
##                            PC8     PC9    PC10    PC11    PC12   PC13   PC14
## Standard deviation     0.35699 0.28462 0.23336 0.18394 0.16031 0.1580 0.1234
## Proportion of Variance 0.00255 0.00162 0.00109 0.00068 0.00051 0.0005 0.0003
## Cumulative Proportion  0.99444 0.99606 0.99715 0.99783 0.99834 0.9988 0.9991
##                           PC15    PC16    PC17    PC18    PC19    PC20    PC21
## Standard deviation     0.11153 0.08147 0.06216 0.05683 0.05387 0.05033 0.04315
## Proportion of Variance 0.00025 0.00013 0.00008 0.00006 0.00006 0.00005 0.00004
## Cumulative Proportion  0.99939 0.99953 0.99960 0.99967 0.99973 0.99978 0.99982
##                           PC22    PC23    PC24    PC25    PC26    PC27    PC28
## Standard deviation     0.03661 0.03539 0.03116 0.03070 0.02797 0.02444 0.02371
## Proportion of Variance 0.00003 0.00003 0.00002 0.00002 0.00002 0.00001 0.00001
## Cumulative Proportion  0.99984 0.99987 0.99989 0.99991 0.99992 0.99993 0.99994
##                           PC29    PC30    PC31    PC32    PC33   PC34    PC35
## Standard deviation     0.02256 0.01999 0.01853 0.01665 0.01481 0.0138 0.01311
## Proportion of Variance 0.00001 0.00001 0.00001 0.00001 0.00000 0.0000 0.00000
## Cumulative Proportion  0.99995 0.99996 0.99997 0.99997 0.99998 1.0000 0.99999
##                           PC36    PC37     PC38     PC39     PC40     PC41
## Standard deviation     0.01161 0.01139 0.009857 0.009413 0.007457 0.006803
## Proportion of Variance 0.00000 0.00000 0.000000 0.000000 0.000000 0.000000
## Cumulative Proportion  0.99999 0.99999 0.999990 1.000000 1.000000 1.000000
##                            PC42     PC43    PC44     PC45     PC46     PC47
## Standard deviation     0.006201 0.005301 0.00458 0.003927 0.002761 0.002391
## Proportion of Variance 0.000000 0.000000 0.00000 0.000000 0.000000 0.000000
## Cumulative Proportion  1.000000 1.000000 1.00000 1.000000 1.000000 1.000000
##                            PC48      PC49      PC50
## Standard deviation     0.001851 0.0008959 1.432e-10
## Proportion of Variance 0.000000 0.0000000 0.000e+00
## Cumulative Proportion  1.000000 1.0000000 1.000e+00
```

```
str(Claw.pca)
```

```
## List of 5
##  $ sdev    : num [1:50] 5.896 3.19 1.558 1.052 0.725 ...
##  $ rotation: num [1:50, 1:50] -0.0706 -0.0982 -0.1154 -0.1341 -0.1296 ...
##   ..- attr(*, "dimnames")=List of 2
##   .. ..$ : chr [1:50] "V1" "V2" "V3" "V4" ...
##   .. ..$ : chr [1:50] "PC1" "PC2" "PC3" "PC4" ...
##  $ center  : Named num [1:50] 3.09 8.63 11.39 11.45 10.85 ...
##   ..- attr(*, "names")= chr [1:50] "V1" "V2" "V3" "V4" ...
##  $ scale   : Named num [1:50] 3.34 6.69 6.45 4.91 3.93 ...
##   ..- attr(*, "names")= chr [1:50] "V1" "V2" "V3" "V4" ...
##  $ x       : num [1:57, 1:50] -3.343 -4.255 -2.537 0.952 3.818 ...
##   ..- attr(*, "dimnames")=List of 2
##   .. ..$ : chr [1:57] "Allosaurus.p" "Alxasaurus.p" "Aorun.p" "Bannykus.p" ...
##   .. ..$ : chr [1:50] "PC1" "PC2" "PC3" "PC4" ...
##  - attr(*, "class")= chr "prcomp"
```

```
##Plot PCA
ggbiplot(Claw.pca) #Normal plot#
```

```
ggbiplot(Claw.pca, labels=rownames(Claw))  #Plot with labels#
```

```
###Groups PCA by Clade###
group<-read.csv('Group data.csv',header = F)

Claw.group<-c(group[,c(1)])
Test.group<-c(group[,c(3)])
Clade.group<-c(group[,c(4)])

ggbiplot(Claw.pca,ellipse=TRUE, groups=Claw.group)
```

```
ggbiplot(Claw.pca,ellipse=TRUE, groups=Test.group)
```

## 2.3 Add a functions of making covex hulls

```
StatBag <- ggproto("Statbag", Stat,
                   compute_group = function(data, scales, prop = 0.5) {
                     
                     #################################
                     #################################
                     # originally from aplpack package, plotting functions removed
                     plothulls_ <- function(x, y, fraction, n.hull = 1,
                                            col.hull, lty.hull, lwd.hull, density=0, ...){
                       # function for data peeling:
                       # x,y : data
                       # fraction.in.inner.hull : max percentage of points within the hull to be drawn
                       # n.hull : number of hulls to be plotted (if there is no fractiion argument)
                       # col.hull, lty.hull, lwd.hull : style of hull line
                       # plotting bits have been removed, BM 160321
                       # pw 130524
                       if(ncol(x) == 2){ y <- x[,2]; x <- x[,1] }
                       n <- length(x)
                       if(!missing(fraction)) { # find special hull
                         n.hull <- 1
                         if(missing(col.hull)) col.hull <- 1
                         if(missing(lty.hull)) lty.hull <- 1
                         if(missing(lwd.hull)) lwd.hull <- 1
                         x.old <- x; y.old <- y
                         idx <- chull(x,y); x.hull <- x[idx]; y.hull <- y[idx]
                         for( i in 1:(length(x)/3)){
                           x <- x[-idx]; y <- y[-idx]
                           if( (length(x)/n) < fraction ){
                             return(cbind(x.hull,y.hull))
                           }
                           idx <- chull(x,y); x.hull <- x[idx]; y.hull <- y[idx];
                         }
                       }
                       if(missing(col.hull)) col.hull <- 1:n.hull
                       if(length(col.hull)) col.hull <- rep(col.hull,n.hull)
                       if(missing(lty.hull)) lty.hull <- 1:n.hull
                       if(length(lty.hull)) lty.hull <- rep(lty.hull,n.hull)
                       if(missing(lwd.hull)) lwd.hull <- 1
                       if(length(lwd.hull)) lwd.hull <- rep(lwd.hull,n.hull)
                       result <- NULL
                       for( i in 1:n.hull){
                         idx <- chull(x,y); x.hull <- x[idx]; y.hull <- y[idx]
                         result <- c(result, list( cbind(x.hull,y.hull) ))
                         x <- x[-idx]; y <- y[-idx]
                         if(0 == length(x)) return(result)
                       }
                       result
                     } # end of definition of plothulls
                     #################################
                     
                     
                     # prepare data to go into function below
                     the_matrix <- matrix(data = c(data$x, data$y), ncol = 2)
                     
                     # get data out of function as df with names
                     setNames(data.frame(plothulls_(the_matrix, fraction = prop)), nm = c("x", "y"))
                     # how can we get the hull and loop vertices passed on also?
                   },
                   
                   required_aes = c("x", "y")
)

#' @inheritParams ggplot2::stat_identity
#' @param prop Proportion of all the points to be included in the bag (default is 0.5)
stat_bag <- function(mapping = NULL, data = NULL, geom = "polygon",
                     position = "identity", na.rm = FALSE, show.legend = NA, 
                     inherit.aes = TRUE, prop = 0.5, alpha = 0.3, ...) {
  layer(
    stat = StatBag, data = data, mapping = mapping, geom = geom, 
    position = position, show.legend = show.legend, inherit.aes = inherit.aes,
    params = list(na.rm = na.rm, prop = prop, alpha = alpha, ...)
  )
}


geom_bag <- function(mapping = NULL, data = NULL,
                     stat = "identity", position = "identity",
                     prop = 0.5, 
                     alpha = 0.3,
                     ...,
                     na.rm = FALSE,
                     show.legend = NA,
                     inherit.aes = TRUE) {
  layer(
    data = data,
    mapping = mapping,
    stat = StatBag,
    geom = GeomBag,
    position = position,
    show.legend = show.legend,
    inherit.aes = inherit.aes,
    params = list(
      na.rm = na.rm,
      alpha = alpha,
      prop = prop,
      ...
    )
  )
}

#' @rdname ggplot2-ggproto
#' @format NULL
#' @usage NULL
#' @export
GeomBag <- ggproto("GeomBag", Geom,
                   draw_group = function(data, panel_scales, coord) {
                     n <- nrow(data)
                     if (n == 1) return(zeroGrob())
                     
                     munched <- coord_munch(coord, data, panel_scales)
                     # Sort by group to make sure that colors, fill, etc. come in same order
                     munched <- munched[order(munched$group), ]
                     
                     # For gpar(), there is one entry per polygon (not one entry per point).
                     # We'll pull the first value from each group, and assume all these values
                     # are the same within each group.
                     first_idx <- !duplicated(munched$group)
                     first_rows <- munched[first_idx, ]
                     
                     ggplot2:::ggname("geom_bag",
                                      grid:::polygonGrob(munched$x, munched$y, default.units = "native",
                                                         id = munched$group,
                                                         gp = grid::gpar(
                                                           col = first_rows$colour,
                                                           fill = alpha(first_rows$fill, first_rows$alpha),
                                                           lwd = first_rows$size * .pt,
                                                           lty = first_rows$linetype
                                                         )
                                      )
                     )
                     
                     
                   },
                   
                   default_aes = aes(colour = "NA", fill = "grey20", size = 0.5, linetype = 1,
                                     alpha = NA, prop = 0.5),
                   
                   handle_na = function(data, params) {
                     data
                   },
                   
                   required_aes = c("x", "y"),
                   
                   draw_key = draw_key_polygon
)
```

## 2.4 Plots of function PCA of hulls

```
#Plot with hull-labels and axes#
g <- ggbiplot(Claw.pca, obs.scale = 1, var.scale = 1, labels=rownames(Claw),
              groups = Claw.group, ellipse = FALSE, circle = TRUE)
g <- g + scale_color_discrete(name = '')
g <- g + theme(legend.direction = 'horizontal', legend.position = 'top')
g<- g + geom_bag(aes(group = Claw.group, fill = Claw.group), prop = 1) 
g
```

```
ggsave(file='Plot with hull-labels and axes.png', plot=g,units="mm", width=500, height=500)
ggsave(file='Plot with hull-labels and axes.svg', plot=g,units="mm", width=500, height=500)
ggsave(file='Plot with hull-labels and axes.pdf', plot=g,units="mm", width=500, height=500)

#Plot with labels and no axes#
g <- ggbiplot(Claw.pca, obs.scale = 1, var.scale = 1, labels=rownames(Claw),
              groups = Claw.group, ellipse = FALSE, circle = TRUE,var.axes=FALSE)
g <- g + scale_color_discrete(name = '')
g <- g + theme(legend.direction = 'horizontal', legend.position = 'top')
g<-g + geom_bag(aes(group = Claw.group, fill = Claw.group), prop = 1) 
g
```

```
ggsave(file='Plot with hull-labels and no axes.png', plot=g,units="mm", width=500, height=500)
ggsave(file='Plot with hull-labels and no axes.svg', plot=g,units="mm", width=500, height=500)
ggsave(file='Plot with hull-labels and no axes.pdf', plot=g,units="mm", width=500, height=500)

#Plot with hull-no labels and no axes#
USEfigure <- ggbiplot(Claw.pca, obs.scale = 1, var.scale = 1,
              groups = Test.group,ellipse = FALSE, circle = FFALSE,var.axes=FALSE)
USEfigure <- USEfigure + scale_color_discrete(name = '')
USEfigure <- USEfigure + theme(legend.direction = 'horizontal', legend.position = 'top')
USEfigure<-USEfigure + geom_bag(aes(group = Claw.group, fill = Claw.group), prop = 1) 
USEfigure
```

```
ggsave(file='Plot with hull-no labels and no axes.png', plot=USEfigure,units="mm", width=500, height=500)
ggsave(file='Plot with hull-no labels and no axes.svg', plot=USEfigure,units="mm", width=500, height=500)
ggsave(file='Plot with hull-no labels and no axes.pdf', plot=USEfigure,units="mm", width=500, height=500)


#Plot with hull-labels and axes-A-T-NM and axes#
USEfigure2 <- ggbiplot(Claw.pca, obs.scale = 1, var.scale = 1,labels=rownames(Claw),groups = Clade.group,
              ellipse = FALSE, circle = FALSE,var.axes=TRUE)
USEfigure2  <- USEfigure2  + scale_color_discrete(name = '')
USEfigure2  <- USEfigure2  + theme(legend.direction = 'horizontal', legend.position = 'top')
USEfigure2 <-USEfigure2  + geom_bag(aes(group = Claw.group, fill =Clade.group), prop = 1) 
USEfigure2
```

```
ggsave(file='Plot with hull-labels and axes-A-T-NM and axes.png', plot=USEfigure2 ,units="mm", width=500, height=500)
ggsave(file='Plot with hull-labels and axes-A-T-NM and axes.svg', plot=USEfigure2 ,units="mm", width=500, height=500)
ggsave(file='Plot with hull-labels and axes-A-T-NM and axes.pdf', plot=USEfigure2 ,units="mm", width=500, height=500)
```

## 2.5 Plots of Combined figures of Functional PCA

```
g <- ggbiplot(Claw.pca, obs.scale = 1, var.scale = 1,labels=rownames(Claw),groups = Clade.group,
              ellipse = FALSE, circle = FALSE,var.axes=FALSE,alpha=0)
g <- g + scale_color_discrete(name = '')
g <- g + theme(legend.direction = 'horizontal', legend.position = 'top')
g<-g + geom_bag(aes(group = Claw.group, fill =Clade.group), prop = 1) 
g
```

```
f <- ggbiplot(Claw.pca, obs.scale = 1, var.scale = 1,
              ellipse = FALSE, circle = FFALSE,var.axes=FALSE,alpha=0)
f <- f + scale_color_discrete(name = '')
f <- f + theme(legend.direction = 'horizontal', legend.position = 'top')
f<-f + geom_bag(aes(group = Claw.group, fill = Clade.group), prop = 1) 
f
```

```
myplot<-grid.arrange(g,f, ncol=1, nrow=2)
```

```
myplot
```

```
## TableGrob (2 x 1) "arrange": 2 grobs
##   z     cells    name           grob
## 1 1 (1-1,1-1) arrange gtable[layout]
## 2 2 (2-2,1-1) arrange gtable[layout]
```

```
ggsave(file='Plot with hull-labels and axes-A-T-NM.png', plot=myplot,units="mm", width=500, height=500)
ggsave(file='Plot with hull-labels and axes-A-T-NM.svg', plot=myplot,units="mm", width=500, height=500)
ggsave(file='Plot with hull-labels and axes-A-T-NM.pdf', plot=myplot,units="mm", width=500, height=500)
```

# 3.Caculating the hull areas

## 3.1 Hull areas

```
rownames(Claw.pca$x)
```

```
##  [1] "Allosaurus.p"       "Alxasaurus.p"       "Aorun.p"           
##  [4] "Bannykus.p"         "Beipiaosaurus.p"    "Eremotherium.p"    
##  [7] "Erliansaurus.p"     "Falcarius.p"        "Guanlong.p"        
## [10] "Haplocheirus.p"     "Linhenykus.p"       "Manis.p"           
## [13] "Mononykus.p"        "Nothronychus.p"     "Puma.p"            
## [16] "Shishugounykus.p"   "Tamandua.p"         "Therizinosaurus.p" 
## [19] "Tugulusaurus.p"     "Allosaurus.sd"      "Alxasaurus.sd"     
## [22] "Aorun.sd"           "Bannykus.sd"        "Beipiaosaurus.sd"  
## [25] "Eremotherium.sd"    "Erliansaurus.sd"    "Falcarius.sd"      
## [28] "Guanlong.sd"        "Haplocheirus.sd"    "Linhenykus.sd"     
## [31] "Manis.sd"           "Mononykus.sd"       "Nothronychus.sd"   
## [34] "Puma.sd"            "Shishugounykus.sd"  "Tamandua.sd"       
## [37] "Therizinosaurus.sd" "Tugulusaurus.sd"    "Allosaurus.hp"     
## [40] "Alxasaurus.hp"      "Aorun.hp"           "Bannykus.hp"       
## [43] "Beipiaosaurus.hp"   "Eremotherium.hp"    "Erliansaurus.hp"   
## [46] "Falcarius.hp"       "Guanlong.hp"        "Haplocheirus.hp"   
## [49] "Linhenykus.hp"      "Manis.hp"           "Mononykus.hp"      
## [52] "Nothronychus.hp"    "Puma.hp"            "Shishugounykus.hp" 
## [55] "Tamandua.hp"        "Therizinosaurus.hp" "Tugulusaurus.hp"
```

```
names(Claw.group)
```

```
## NULL
```

```
pc.scores <- Claw.pca$x
plot(pc.scores[,1], pc.scores[,2], cex.main=1, ylab="PC2", xlab="PC1", cex.lab=1.2, pch=19, col= "black", cex=1.2)
abline(h=0, lty=2,col="gray60")
abline(v=0, lty=2, col="gray60")
```

```
Allosaurus<-c("Allosaurus.p", "Allosaurus.sd", "Allosaurus.hp")
Alxasaurus<-c("Alxasaurus.p", "Alxasaurus.sd", "Alxasaurus.hp")
Aorun<-c("Aorun.p", "Aorun.sd", "Aorun.hp")
Bannykus<-c("Bannykus.p", "Bannykus.sd", "Bannykus.hp")
Beipiaosaurus<-c("Beipiaosaurus.p", "Beipiaosaurus.sd", "Beipiaosaurus.hp")
Eremotherium<-c("Eremotherium.p", "Eremotherium.sd", "Eremotherium.hp")
Erliansaurus<-c("Erliansaurus.p", "Erliansaurus.sd", "Erliansaurus.hp")
Falcarius<-c("Falcarius.p", "Falcarius.sd", "Falcarius.hp")
Guanlong<-c("Guanlong.p", "Guanlong.sd", "Guanlong.hp")
Haplocheirus<-c("Haplocheirus.p", "Haplocheirus.sd", "Haplocheirus.hp")
Linhenykus<-c("Linhenykus.p", "Linhenykus.sd", "Linhenykus.hp")
Manis<-c("Manis.p", "Manis.sd", "Manis.hp")
Mononykus<-c("Mononykus.p", "Mononykus.sd", "Mononykus.hp")
Nothronychus<-c("Nothronychus.p", "Nothronychus.sd", "Nothronychus.hp")
Puma<-c("Puma.p", "Puma.sd", "Puma.hp")
Shishugounykus<-c("Shishugounykus.p", "Shishugounykus.sd", "Shishugounykus.hp")
Tamandua<-c("Tamandua.p", "Tamandua.sd", "Tamandua.hp")
Therizinosaurus<-c("Therizinosaurus.p", "Therizinosaurus.sd", "Therizinosaurus.hp")
Tugulusaurus<-c("Tugulusaurus.p", "Tugulusaurus.sd", "Tugulusaurus.hp")

taxon_bins <- list(Allosaurus,
                   Alxasaurus,
                   Aorun,
                   Bannykus,
                   Beipiaosaurus,
                   Eremotherium,
                   Erliansaurus,
                   Falcarius,
                   Guanlong,
                   Haplocheirus,
                   Linhenykus,
                   Manis,
                   Mononykus,
                   Nothronychus,
                   Puma,
                   Shishugounykus,
                   Tamandua,
                   Therizinosaurus,
                   Tugulusaurus)

rownames(Claw.pca$x)
```

```
##  [1] "Allosaurus.p"       "Alxasaurus.p"       "Aorun.p"           
##  [4] "Bannykus.p"         "Beipiaosaurus.p"    "Eremotherium.p"    
##  [7] "Erliansaurus.p"     "Falcarius.p"        "Guanlong.p"        
## [10] "Haplocheirus.p"     "Linhenykus.p"       "Manis.p"           
## [13] "Mononykus.p"        "Nothronychus.p"     "Puma.p"            
## [16] "Shishugounykus.p"   "Tamandua.p"         "Therizinosaurus.p" 
## [19] "Tugulusaurus.p"     "Allosaurus.sd"      "Alxasaurus.sd"     
## [22] "Aorun.sd"           "Bannykus.sd"        "Beipiaosaurus.sd"  
## [25] "Eremotherium.sd"    "Erliansaurus.sd"    "Falcarius.sd"      
## [28] "Guanlong.sd"        "Haplocheirus.sd"    "Linhenykus.sd"     
## [31] "Manis.sd"           "Mononykus.sd"       "Nothronychus.sd"   
## [34] "Puma.sd"            "Shishugounykus.sd"  "Tamandua.sd"       
## [37] "Therizinosaurus.sd" "Tugulusaurus.sd"    "Allosaurus.hp"     
## [40] "Alxasaurus.hp"      "Aorun.hp"           "Bannykus.hp"       
## [43] "Beipiaosaurus.hp"   "Eremotherium.hp"    "Erliansaurus.hp"   
## [46] "Falcarius.hp"       "Guanlong.hp"        "Haplocheirus.hp"   
## [49] "Linhenykus.hp"      "Manis.hp"           "Mononykus.hp"      
## [52] "Nothronychus.hp"    "Puma.hp"            "Shishugounykus.hp" 
## [55] "Tamandua.hp"        "Therizinosaurus.hp" "Tugulusaurus.hp"
```

```
names(Claw.group)
```

```
## NULL
```

```
library(geometry)

# this has tbe function convhulln for getting hull volume/area
Allosaurus.hull <- convhulln(pc.scores[Allosaurus,][,1:2], output.options=TRUE)$vol
Allosaurus.hull
```

```
## [1] 6.180542
```

```
Alxasaurus.hull <- convhulln(pc.scores[Alxasaurus,][,1:2], output.options=TRUE)$vol
Alxasaurus.hull
```

```
## [1] 0.1228832
```

```
Aorun.hull <- convhulln(pc.scores[Aorun,][,1:2], output.options=TRUE)$vol
Aorun.hull
```

```
## [1] 4.428162
```

```
Bannykus.hull <- convhulln(pc.scores[Bannykus,][,1:2], output.options=TRUE)$vol
Bannykus.hull
```

```
## [1] 0.341607
```

```
Beipiaosaurus.hull <- convhulln(pc.scores[Beipiaosaurus,][,1:2], output.options=TRUE)$vol
Beipiaosaurus.hull
```

```
## [1] 0.7546681
```

```
Eremotherium.hull <- convhulln(pc.scores[Eremotherium,][,1:2], output.options=TRUE)$vol
Eremotherium.hull
```

```
## [1] 0.8393833
```

```
Erliansaurus.hull <- convhulln(pc.scores[Erliansaurus,][,1:2], output.options=TRUE)$vol
Erliansaurus.hull
```

```
## [1] 2.054281
```

```
Falcarius.hull <- convhulln(pc.scores[Falcarius,][,1:2], output.options=TRUE)$vol
Falcarius.hull
```

```
## [1] 0.5593716
```

```
Guanlong.hull <- convhulln(pc.scores[Guanlong,][,1:2], output.options=TRUE)$vol
Guanlong.hull
```

```
## [1] 2.524644
```

```
Haplocheirus.hull <- convhulln(pc.scores[Haplocheirus,][,1:2], output.options=TRUE)$vol
Haplocheirus.hull
```

```
## [1] 0.2348031
```

```
Linhenykus.hull <- convhulln(pc.scores[Linhenykus,][,1:2], output.options=TRUE)$vol
Linhenykus.hull
```

```
## [1] 6.246133e-06
```

```
Manis.hull <- convhulln(pc.scores[Manis,][,1:2], output.options=TRUE)$vol
Manis.hull
```

```
## [1] 0.09410633
```

```
Mononykus.hull <- convhulln(pc.scores[Mononykus,][,1:2], output.options=TRUE)$vol
Mononykus.hull
```

```
## [1] 2.042926e-06
```

```
Nothronychus.hull <- convhulln(pc.scores[Nothronychus,][,1:2], output.options=TRUE)$vol
Nothronychus.hull
```

```
## [1] 0.6635903
```

```
Puma.hull <- convhulln(pc.scores[Puma,][,1:2], output.options=TRUE)$vol
Puma.hull
```

```
## [1] 2.492232
```

```
Shishugounykus.hull <- convhulln(pc.scores[Shishugounykus,][,1:2], output.options=TRUE)$vol
Shishugounykus.hull
```

```
## [1] 1.070581
```

```
Tamandua.hull <- convhulln(pc.scores[Tamandua,][,1:2], output.options=TRUE)$vol
Tamandua.hull
```

```
## [1] 1.208223
```

```
Therizinosaurus.hull <- convhulln(pc.scores[Therizinosaurus,][,1:2], output.options=TRUE)$vol
Therizinosaurus.hull
```

```
## [1] 1.539722
```

```
Tugulusaurus.hull <- convhulln(pc.scores[Tugulusaurus,][,1:2], output.options=TRUE)$vol
Tugulusaurus.hull
```

```
## [1] 2.01754
```

```
plot(pc.scores[,1], pc.scores[,2], cex.main=1, ylab="PC2", xlab="PC1", cex.lab=1.2, pch=19, col= "black", cex=1.2)
abline(h=0, lty=2,col="gray60")
abline(v=0, lty=2, col="gray60")

# custom function for making a convex hull
Plot_ConvexHull<-function(xcoord, ycoord, lcolor){
  hpts <- chull(x = xcoord, y = ycoord)
  hpts <- c(hpts, hpts[1])
  lines(xcoord[hpts], ycoord[hpts], col = lcolor)}

#test plotting hulls around groups

Plot_ConvexHull(xcoord=pc.scores[Tugulusaurus,][,1], ycoord= pc.scores[Tugulusaurus,][,2], lcolor="#68228B")

Plot_ConvexHull(xcoord=pc.scores[Nothronychus,][,1], ycoord= pc.scores[Nothronychus,][,2], lcolor="blue")

Plot_ConvexHull(xcoord=pc.scores[Haplocheirus,][,1], ycoord= pc.scores[Haplocheirus,][,2], lcolor="red")

# can also plot separate points e.g.
points(pc.scores[Haplocheirus,][,1], pc.scores[Haplocheirus,][,2], pch=25, bg="red", col="black",cex=1.5)
```

```
#compile list of hull areas
hull_area_list <- c(Allosaurus.hull, Alxasaurus.hull, Aorun.hull, Bannykus.hull, Beipiaosaurus.hull, Eremotherium.hull,Erliansaurus.hull, Falcarius.hull, Guanlong.hull, Haplocheirus.hull, Linhenykus.hull,Manis.hull, Mononykus.hull, Nothronychus.hull,Puma.hull, Shishugounykus.hull, Tamandua.hull, Therizinosaurus.hull, Tugulusaurus.hull)


Species<-c("Allosaurus","Alxasaurus","Aorun","Bannykus","Beipiaosaurus","Eremontherium", "Erliansaurus", "Falcarius",
            "Guanlong","Haplocheirus","Linhenykus", "Manis","Mononykus","Nothronychus","Puma", "Shishugounykus",
            "Tamandua","Therizinosaurus","Tugulusaurus")

Clade<-c("Non_maniraptoran","Therizinosauria","Alvarezsaurioidea","Alvarezsaurioidea","Therizinosauria","Mammals", "Therizinosauria", "Therizinosauria","Non_maniraptoran","Alvarezsaurioidea","Alvarezsaurioidea","Mammals","Alvarezsaurioidea","Therizinosauria","Mammals", "Alvarezsaurioidea","Mammals","Therizinosauria","Alvarezsaurioidea")


hull_area_list<-data.frame(Species,hull_area_list,Clade)
hull_area_list
```

```
##            Species hull_area_list             Clade
## 1       Allosaurus   6.180542e+00  Non_maniraptoran
## 2       Alxasaurus   1.228832e-01   Therizinosauria
## 3            Aorun   4.428162e+00 Alvarezsaurioidea
## 4         Bannykus   3.416070e-01 Alvarezsaurioidea
## 5    Beipiaosaurus   7.546681e-01   Therizinosauria
## 6    Eremontherium   8.393833e-01           Mammals
## 7     Erliansaurus   2.054281e+00   Therizinosauria
## 8        Falcarius   5.593716e-01   Therizinosauria
## 9         Guanlong   2.524644e+00  Non_maniraptoran
## 10    Haplocheirus   2.348031e-01 Alvarezsaurioidea
## 11      Linhenykus   6.246133e-06 Alvarezsaurioidea
## 12           Manis   9.410633e-02           Mammals
## 13       Mononykus   2.042926e-06 Alvarezsaurioidea
## 14    Nothronychus   6.635903e-01   Therizinosauria
## 15            Puma   2.492232e+00           Mammals
## 16  Shishugounykus   1.070581e+00 Alvarezsaurioidea
## 17        Tamandua   1.208223e+00           Mammals
## 18 Therizinosaurus   1.539722e+00   Therizinosauria
## 19    Tugulusaurus   2.017540e+00 Alvarezsaurioidea
```

# 4.Caculating the centroid

## 4.1 Coordinates

```
ind.coord <- Claw.pca$x
ind.coord
```

```
##                            PC1        PC2          PC3         PC4
## Allosaurus.p       -3.34322735  1.5024415 -0.283485671 -0.34270920
## Alxasaurus.p       -4.25547189  3.8567721  0.968591611  0.08721399
## Aorun.p            -2.53687163  0.1436204 -0.570672273  0.73341391
## Bannykus.p          0.95231302 -2.6743476 -1.315046636  0.82448412
## Beipiaosaurus.p     3.81786982 -4.0764147  0.663037687 -0.51422791
## Eremotherium.p     -0.86870155 -1.4597797 -0.248024309 -1.75369054
## Erliansaurus.p     -1.31118643 -2.7903685 -0.344203520 -0.37516780
## Falcarius.p        -0.05572564 -1.9047215 -0.416316361 -0.23129536
## Guanlong.p         -2.45530339  0.2594165  0.018335690 -0.35313360
## Haplocheirus.p     -1.30671697 -1.4332262 -0.213730587  0.47816196
## Linhenykus.p       -4.41812298  4.2021227  0.265993563  1.60316911
## Manis.p            -2.90212515 -0.3406726 -1.499151122 -0.59876132
## Mononykus.p        -3.56290532  1.4871996 -1.755561286  1.16844639
## Nothronychus.p      1.75663273 -3.5751840 -0.299306302  0.24297926
## Puma.p             -3.33456905  1.8648867  0.569785594 -0.18090112
## Shishugounykus.p   -3.94190947  3.1544965  1.694840912 -0.05927715
## Tamandua.p          3.45214163 -6.2439935  3.616944678  1.07636588
## Therizinosaurus.p  21.89583870  6.1195515 -1.392877650 -0.28444559
## Tugulusaurus.p     -3.13483590  2.0373422  2.160661465 -0.24668631
## Allosaurus.sd       2.40864451 -2.2084409  0.040838848 -0.64252013
## Alxasaurus.sd      -0.95532526 -3.3378570 -0.158130021 -1.41236044
## Aorun.sd            2.48152554 -2.3874001 -0.009313851 -0.28450104
## Bannykus.sd         4.78836331 -3.8180150 -0.238886670 -0.97482489
## Beipiaosaurus.sd    4.87982360 -3.9494467  0.097597024 -0.86851566
## Eremotherium.sd    -0.30507397 -1.1973785 -1.177209968 -1.72176884
## Erliansaurus.sd     0.13827691 -2.3141258 -0.325772888  0.21834034
## Falcarius.sd        4.22618045 -3.9608883  0.266866241 -0.70222579
## Guanlong.sd        -0.37376791  0.4249703  0.029826995  0.04118170
## Haplocheirus.sd     2.11634198 -1.4224292 -0.702595873 -0.48661276
## Linhenykus.sd      -4.41812506  4.2020874  0.265921136  1.60319849
## Manis.sd           -1.85871510 -2.9528135 -1.125617339 -1.70383349
## Mononykus.sd       -3.56290888  1.4872153 -1.755588351  1.16844423
## Nothronychus.sd     0.31039204  0.9309279 -0.507208412  1.37820611
## Puma.sd            -2.13270643  2.1343638  1.170282819 -0.09216460
## Shishugounykus.sd  -1.15998242  0.7198907 -0.519918949  1.43754927
## Tamandua.sd         9.74696208 -4.0015429  3.733625456  2.04401399
## Therizinosaurus.sd 21.26729943  7.3602389 -2.738420516 -1.89605637
## Tugulusaurus.sd    -2.15120564  1.8696094  2.298429270  0.41183879
## Allosaurus.hp      -2.04750450 -1.4825623 -0.314651076 -0.39195941
## Alxasaurus.hp      -3.79771123  2.9332823  0.359079842  0.29295689
## Aorun.hp           -2.74668706 -1.5153310 -1.568007885  0.49990871
## Bannykus.hp        -2.08910621 -1.9456923 -1.703866342  0.31565575
## Beipiaosaurus.hp   -0.91745173 -3.2212909 -0.772218892 -0.32273401
## Eremotherium.hp    -3.23549043  0.4168465 -1.691245211 -1.09242193
## Erliansaurus.hp    -3.41412410 -0.6467804 -1.552548924 -0.36663212
## Falcarius.hp       -2.62972535 -0.9299619 -1.627619305 -0.53516110
## Guanlong.hp        -4.10304303  2.5541163 -0.525680668 -0.11686757
## Haplocheirus.hp    -2.66357265 -1.5746950 -0.525976516 -0.08639338
## Linhenykus.hp      -4.16218631  2.5353954 -1.360361427  0.98195190
## Manis.hp           -4.23849061  3.1852543 -0.170677713  1.09097847
## Mononykus.hp       -4.03102550  2.4064425 -1.849324781  1.76856272
## Nothronychus.hp     1.15428179 -2.6160904 -0.458269361  0.99387454
## Puma.hp            -4.50500872  5.7497375  3.737623318 -1.93428326
## Shishugounykus.hp  -4.55826939  2.9242360  0.133154579  0.36633316
## Tamandua.hp         2.12800856 -6.3318209  3.458629305  0.44916975
## Therizinosaurus.hp 20.77900846  3.4247211  1.803375138  2.03562987
## Tugulusaurus.hp    -4.81502435  6.4260867  4.364045481 -2.73989661
##                              PC5          PC6          PC7          PC8
## Allosaurus.p       -0.0803834667  0.048666140 -0.277900283  0.231839785
## Alxasaurus.p       -0.0007094108 -0.242251885 -0.121083159 -0.038016410
## Aorun.p            -0.1208205340 -0.017315659  0.107766425  0.280608743
## Bannykus.p          1.0238962142  0.023122944  0.201095925 -0.247819176
## Beipiaosaurus.p    -0.3393671392  0.202394490  0.051158895 -0.028703088
## Eremotherium.p     -0.0220844138  0.072215270 -0.696725768 -0.176682732
## Erliansaurus.p      0.2245772333  0.557721656 -0.062968096  0.252244092
## Falcarius.p        -0.8847587740  0.361634774 -0.138734364 -0.306011593
## Guanlong.p          0.7625990566 -0.661855950 -0.063855977  0.365891232
## Haplocheirus.p      0.6625423566  0.153966407  0.360014305  0.317276519
## Linhenykus.p       -0.9302204415  0.158293518  0.022043176 -0.762216386
## Manis.p             0.1977846251  0.927361339  0.279380797 -0.134959664
## Mononykus.p         0.0841033824  0.401892091  0.679228994  0.064613330
## Nothronychus.p      0.2481384147 -0.248385397  0.342740106 -0.493822518
## Puma.p             -0.0824510942 -0.683605493 -0.611024404 -0.167857492
## Shishugounykus.p   -0.9689452817  0.396706682 -0.498471551  0.693902624
## Tamandua.p         -0.7719227094 -0.450450258  0.207845175 -0.022760826
## Therizinosaurus.p  -0.1252645009 -0.006730212  0.002182203 -0.292593321
## Tugulusaurus.p     -0.5938853580  1.101313525  1.008229369  0.119627576
## Allosaurus.sd       0.1782898879 -0.059174895  0.017084358  0.125008016
## Alxasaurus.sd       0.2461749306  0.233004308  0.033077102  0.097277485
## Aorun.sd           -0.8504325778 -0.128456751 -0.116684049 -0.489252939
## Bannykus.sd        -1.1978091922 -0.307488970 -0.428504016 -0.837022819
## Beipiaosaurus.sd    0.1784973935  0.636133373 -0.019253346  0.308175320
## Eremotherium.sd     0.8086968064  0.362955395 -0.349093968  0.143764613
## Erliansaurus.sd     0.6470187491  0.681518406 -0.102675730 -0.107019473
## Falcarius.sd       -0.6268617955 -0.090938393  0.076648193 -0.196294542
## Guanlong.sd         0.5936843086 -0.456990902 -0.260687497 -0.032207684
## Haplocheirus.sd     0.4509015460  0.661717244 -0.185121524  0.013800037
## Linhenykus.sd      -0.9301801793  0.158228272  0.022054811 -0.762297434
## Manis.sd           -0.5426757292  1.313586864 -0.448316274 -0.304076426
## Mononykus.sd        0.0841451223  0.401850088  0.679267416  0.064591716
## Nothronychus.sd    -1.2066208556 -0.482597229 -0.825455574  0.218751740
## Puma.sd             0.0445390674 -0.861326931 -0.344818381  0.003140411
## Shishugounykus.sd   0.1793816372 -0.598133758 -0.273556374 -0.487788346
## Tamandua.sd         0.3477662892 -0.203629124  0.193114260  0.546024420
## Therizinosaurus.sd -1.6074589952 -0.802991439  1.202597630  0.536805852
## Tugulusaurus.sd    -0.3573124918  1.496698100  0.626419695  0.068499417
## Allosaurus.hp       0.0069825754 -0.627857606  0.115681109  0.049170289
## Alxasaurus.hp      -0.6042905163  0.064893641 -1.023265630  1.003609537
## Aorun.hp            0.6599319091 -0.485074377  0.662683898  0.276360406
## Bannykus.hp         0.1743484719 -0.340597984  0.123143654  0.135144775
## Beipiaosaurus.hp    0.0807309840 -0.670469851  0.560303892 -0.078866392
## Eremotherium.hp     1.0002076379 -0.155152211 -0.447527168  0.450362558
## Erliansaurus.hp     0.2711722696  0.035405197  0.331605912  0.018586682
## Falcarius.hp       -0.5984572731  0.027868243 -1.192762480 -0.232431085
## Guanlong.hp         0.7566261588 -0.911633614 -0.432805274  0.169044428
## Haplocheirus.hp     0.3246062666 -0.265203378  0.555877908  0.401990033
## Linhenykus.hp       0.6341281442 -0.333432994  0.432940841 -0.104239079
## Manis.hp           -0.8475848956  0.924570697  0.043271384  0.295503614
## Mononykus.hp        0.3283751208 -0.459357932  0.551241357 -0.185401914
## Nothronychus.hp     0.8958048157 -0.245749953  0.249410885 -0.404438762
## Puma.hp             0.5642585309  0.505567365  0.876896975 -0.297057641
## Shishugounykus.hp  -0.9343167207 -0.193226392 -0.978457649  0.225653562
## Tamandua.hp        -0.9684068158 -0.945366897  0.403756860  0.333880437
## Therizinosaurus.hp  2.2055375482  1.007307825 -1.226159351 -0.115840782
## Tugulusaurus.hp     1.3277737077 -0.981147418  0.107144378 -0.505470725
##                             PC9         PC10        PC11         PC12
## Allosaurus.p        0.320855540 -0.193564568 -0.04566667 -0.021932364
## Alxasaurus.p        0.077330038 -0.166548843  0.12023527 -0.002297907
## Aorun.p             0.117662539  0.394039651 -0.27092321  0.020151372
## Bannykus.p          0.251471272  0.361412874  0.12074083 -0.309025863
## Beipiaosaurus.p    -0.089317623 -0.257396019 -0.33595310  0.199567189
## Eremotherium.p      0.255397901 -0.272723477  0.06770060 -0.081176664
## Erliansaurus.p     -0.427411988  0.060176259  0.04571854 -0.181961090
## Falcarius.p         0.300842877 -0.183756686 -0.24048464  0.125395216
## Guanlong.p          0.131238487  0.156416436 -0.09361790  0.121696562
## Haplocheirus.p     -0.482788618  0.055935727 -0.04853404 -0.125246761
## Linhenykus.p       -0.673217748  0.159122712 -0.08651743  0.020241199
## Manis.p            -0.213878182  0.187945061 -0.13211877 -0.050257637
## Mononykus.p         0.190340785 -0.098228653 -0.05834908 -0.005736390
## Nothronychus.p      0.185682540 -0.490212590  0.16498162 -0.219814561
## Puma.p              0.120206007 -0.139535239  0.07911995  0.059834351
## Shishugounykus.p    0.318895479  0.092167986 -0.25114720 -0.214006825
## Tamandua.p          0.221509439  0.482900859  0.34314155 -0.070690686
## Therizinosaurus.p   0.513885771  0.689999929  0.09475903  0.459384579
## Tugulusaurus.p      0.198857639 -0.266498759  0.03256698  0.184016139
## Allosaurus.sd       0.191606666  0.086241963 -0.10323004 -0.074898771
## Alxasaurus.sd      -0.206491774  0.008692859  0.15304303 -0.030468337
## Aorun.sd            0.368112847  0.230961672 -0.21983096 -0.134621705
## Bannykus.sd         0.172213877 -0.336466982 -0.22020380  0.048986562
## Beipiaosaurus.sd   -0.393984077  0.018400452 -0.21286152 -0.200255813
## Eremotherium.sd    -0.302500345 -0.059963497  0.45758681  0.138439720
## Erliansaurus.sd     0.169661487  0.599398247  0.16728552 -0.143730942
## Falcarius.sd       -0.051499070  0.094369751  0.06226042 -0.082277175
## Guanlong.sd         0.123636468  0.110021869  0.05401313 -0.020309484
## Haplocheirus.sd    -0.161011259  0.271596831 -0.10609852  0.279903036
## Linhenykus.sd      -0.673266210  0.159132884 -0.08649266  0.020303215
## Manis.sd           -0.166588783 -0.199143375  0.17332437  0.064388097
## Mononykus.sd        0.190299390 -0.098155787 -0.05838756 -0.005734138
## Nothronychus.sd    -0.441177088  0.059743812  0.20398902 -0.132506633
## Puma.sd             0.111844626 -0.149022140  0.14338674  0.110904051
## Shishugounykus.sd  -0.022961022 -0.101872199  0.31088195  0.043611280
## Tamandua.sd        -0.504266423 -0.180924776  0.04368938  0.399488545
## Therizinosaurus.sd -0.389894739 -0.235895816  0.15488401 -0.331552648
## Tugulusaurus.sd     0.338854124  0.060950783  0.27661988 -0.163707597
## Allosaurus.hp      -0.063969611 -0.161345692 -0.02319633  0.199389753
## Alxasaurus.hp       0.376366647 -0.029708803 -0.04496276 -0.077755744
## Aorun.hp           -0.071381040 -0.103744107 -0.06132330  0.118024470
## Bannykus.hp        -0.131207162 -0.088690861 -0.09806358  0.096889428
## Beipiaosaurus.hp    0.448665892  0.026951549 -0.34317386  0.009206499
## Eremotherium.hp    -0.152060029  0.140250203  0.19019376  0.048965230
## Erliansaurus.hp     0.056215511  0.089785382 -0.22818143 -0.007439098
## Falcarius.hp       -0.199617796  0.047510620  0.12164776  0.139714374
## Guanlong.hp         0.194002100 -0.132360785  0.17165541  0.039298977
## Haplocheirus.hp    -0.350386294  0.025815347 -0.20338540  0.264858902
## Linhenykus.hp       0.045399444 -0.020148317 -0.06161175 -0.035990127
## Manis.hp            0.006595240 -0.047563325  0.10287631  0.028950011
## Mononykus.hp        0.221365652 -0.249181549  0.02192124 -0.064381093
## Nothronychus.hp     0.127830620 -0.129382206  0.21934737 -0.134566205
## Puma.hp             0.220875651 -0.187273416  0.20841097  0.209217638
## Shishugounykus.hp   0.020215414  0.072555462 -0.11702330  0.017541737
## Tamandua.hp         0.016689140 -0.003234623  0.05236715 -0.105569799
## Therizinosaurus.hp  0.003082724 -0.473592433 -0.28737375 -0.190223368
## Tugulusaurus.hp    -0.438832950  0.313638344 -0.31963605 -0.250232706
##                           PC13         PC14          PC15         PC16
## Allosaurus.p        0.10938645 -0.150595772 -0.0573629342 -0.039946941
## Alxasaurus.p        0.13014705  0.064566287  0.0354309763 -0.016669414
## Aorun.p            -0.08082073 -0.156161352  0.0555528244 -0.019717246
## Bannykus.p         -0.08500040 -0.155994204 -0.0153088967 -0.113004156
## Beipiaosaurus.p     0.05736425  0.033451787  0.0027172508  0.023125208
## Eremotherium.p      0.08322284 -0.190639056 -0.1847676015  0.002559593
## Erliansaurus.p     -0.07373892  0.021504676 -0.1092085856  0.008676733
## Falcarius.p         0.12428602 -0.027372299 -0.0225119148  0.074766514
## Guanlong.p         -0.13087444 -0.192887927 -0.0883559950 -0.021317720
## Haplocheirus.p     -0.14029515  0.281705584 -0.1198150104 -0.050454751
## Linhenykus.p        0.02943073 -0.002269575  0.0598868403  0.047479242
## Manis.p             0.05209250 -0.149214275 -0.0199728000  0.081321240
## Mononykus.p        -0.02161497 -0.137208190  0.0450995154 -0.002322071
## Nothronychus.p      0.36861353  0.071326588  0.1314113227 -0.072581530
## Puma.p             -0.15217950 -0.032485041 -0.0379217150 -0.062558341
## Shishugounykus.p    0.43833068  0.135294346 -0.0286599001  0.036417847
## Tamandua.p          0.04828163 -0.215166306  0.0225205984  0.174581411
## Therizinosaurus.p   0.20380363  0.069617050 -0.0231409014  0.034525255
## Tugulusaurus.p     -0.11385152  0.066917771 -0.0435229764 -0.018122600
## Allosaurus.sd      -0.05301759 -0.177998730  0.0130439610  0.090624055
## Alxasaurus.sd       0.07702712 -0.006808337  0.2671911968  0.021873503
## Aorun.sd           -0.08867484  0.162137448  0.1038939326 -0.070047399
## Bannykus.sd        -0.15508172 -0.038277176 -0.1483160839 -0.079641319
## Beipiaosaurus.sd   -0.21302969  0.036382380 -0.0251518307  0.149065268
## Eremotherium.sd     0.32835207 -0.089788648  0.3264258406 -0.069750321
## Erliansaurus.sd    -0.08469250  0.049757845 -0.1726201308 -0.079744992
## Falcarius.sd       -0.01247030  0.203536699  0.1476144495 -0.238045651
## Guanlong.sd        -0.07699771  0.133683503 -0.0622374140  0.167080315
## Haplocheirus.sd     0.04447058  0.128889875  0.0030925941 -0.071698991
## Linhenykus.sd       0.02930159 -0.002377017  0.0599419542  0.047429258
## Manis.sd            0.15098903 -0.113646749 -0.1764187217  0.105419816
## Mononykus.sd       -0.02157213 -0.137113898  0.0450584550 -0.002320364
## Nothronychus.sd    -0.13807214 -0.162183114 -0.1008871416 -0.195836522
## Puma.sd            -0.17472510  0.108082582  0.0109097767  0.064358636
## Shishugounykus.sd   0.01731880  0.110177234 -0.0118054199  0.156962965
## Tamandua.sd         0.24938339 -0.153246202 -0.1567192303 -0.161859378
## Therizinosaurus.sd -0.07370417 -0.039977732 -0.0591481585  0.020311020
## Tugulusaurus.sd    -0.05465593  0.020529926 -0.0715119439 -0.040074685
## Allosaurus.hp      -0.02708907 -0.007276475  0.0001331352  0.070434391
## Alxasaurus.hp       0.12498781  0.153773619 -0.0684264468 -0.005716353
## Aorun.hp            0.04318925  0.089067432 -0.0404720445  0.045940796
## Bannykus.hp         0.04182751 -0.099130378 -0.0860208414  0.017275086
## Beipiaosaurus.hp   -0.05736776  0.141877010  0.1125757282 -0.066445182
## Eremotherium.hp    -0.16058491  0.109809993  0.0852522728  0.054201146
## Erliansaurus.hp    -0.03540138 -0.091236083  0.1442739540 -0.043089671
## Falcarius.hp       -0.20361364  0.091263440  0.0138397441  0.013099619
## Guanlong.hp        -0.07534909 -0.023191332 -0.0062323973  0.009253618
## Haplocheirus.hp    -0.01438662  0.126225897 -0.0620266064  0.006603227
## Linhenykus.hp       0.11321864 -0.112318904  0.0219738815 -0.040270643
## Manis.hp            0.03684827  0.163504902  0.1359920699  0.020235594
## Mononykus.hp        0.08909380 -0.040368257 -0.0108226388  0.037359642
## Nothronychus.hp     0.11675077  0.298286162 -0.2749764795 -0.002950107
## Puma.hp            -0.45427638 -0.038390393  0.0221553637 -0.063083882
## Shishugounykus.hp  -0.14978518 -0.077888488  0.1101635059 -0.070589398
## Tamandua.hp        -0.13001937  0.016131391  0.2084374288  0.112639754
## Therizinosaurus.hp -0.18438123 -0.037469097  0.1719593324  0.043418496
## Tugulusaurus.hp     0.32960612 -0.028820422 -0.0722051443 -0.019179626
##                            PC17         PC18          PC19          PC20
## Allosaurus.p        0.044990442 -0.045148668 -0.0204655742  0.0060985902
## Alxasaurus.p        0.017833168 -0.010859171  0.0170464640 -0.0009530141
## Aorun.p            -0.048662318 -0.103330519  0.0322027112  0.0176981366
## Bannykus.p         -0.140877069  0.025481889 -0.1000631917 -0.0700836420
## Beipiaosaurus.p    -0.185371574  0.033011507  0.0973180013 -0.0720619612
## Eremotherium.p     -0.042009761 -0.113297634 -0.0121342883 -0.0332025488
## Erliansaurus.p      0.051348707  0.090966901 -0.0093099801  0.0574245569
## Falcarius.p        -0.033273942  0.069408011  0.0720125336 -0.0455357208
## Guanlong.p          0.029917232  0.001559148 -0.0073474126 -0.0334919846
## Haplocheirus.p      0.108861973 -0.040626645 -0.0202955782  0.0599492594
## Linhenykus.p       -0.018717663 -0.021482128 -0.0280688996 -0.0149087029
## Manis.p             0.061089058  0.028204546  0.0352922190  0.1041541196
## Mononykus.p         0.002832537 -0.013745755 -0.0069011749 -0.0179034407
## Nothronychus.p      0.074441067  0.052541069 -0.1047569708  0.0140909397
## Puma.p              0.011764513 -0.001822747 -0.0386359281  0.0069054785
## Shishugounykus.p   -0.006020648  0.005925455 -0.0095848993 -0.0282432027
## Tamandua.p          0.059412573  0.041798138  0.0536877739 -0.1072426197
## Therizinosaurus.p   0.080800333 -0.041617702 -0.0698262967  0.0149405131
## Tugulusaurus.p      0.033019236  0.004716875  0.0007509656 -0.0301963562
## Allosaurus.sd       0.082606782 -0.079160603 -0.0447604633  0.1001508467
## Alxasaurus.sd      -0.001108646 -0.110863738  0.0623560309  0.0875570516
## Aorun.sd           -0.076086061  0.032576324 -0.0323070345  0.0658504084
## Bannykus.sd         0.034469682 -0.015588057  0.0590536056  0.0218282271
## Beipiaosaurus.sd   -0.073120625 -0.084752458 -0.1437747088 -0.0514175422
## Eremotherium.sd    -0.083520969  0.014143989  0.0138020124  0.0098463425
## Erliansaurus.sd    -0.030486754  0.142291288  0.0764830909  0.0216695413
## Falcarius.sd        0.152164429 -0.050062330 -0.0100666293 -0.1478253355
## Guanlong.sd        -0.056929868 -0.044033694 -0.0214028000  0.0277560705
## Haplocheirus.sd    -0.004811256 -0.047816755  0.1388752675  0.0422186572
## Linhenykus.sd      -0.018723544 -0.021464548 -0.0280690126 -0.0149029653
## Manis.sd            0.063411190  0.027486521 -0.0695610446  0.0063541067
## Mononykus.sd        0.002886239 -0.013663078 -0.0069965513 -0.0178028774
## Nothronychus.sd     0.049249293 -0.074285820  0.0680861871  0.0157948637
## Puma.sd            -0.010261469  0.010402283 -0.0186635519  0.0220972205
## Shishugounykus.sd  -0.017461047 -0.002089981 -0.0206880866  0.0272811494
## Tamandua.sd        -0.116186816  0.011327966 -0.0909364842  0.0697542868
## Therizinosaurus.sd -0.072138593  0.053632549  0.0404650537  0.0012311010
## Tugulusaurus.sd    -0.009724961 -0.024924747  0.0055211630  0.0081744882
## Allosaurus.hp       0.038180508 -0.061843591  0.0150792672 -0.0294981282
## Alxasaurus.hp       0.004517803  0.027357517 -0.0033666790  0.0135160281
## Aorun.hp            0.048660400  0.020968910  0.0557281460 -0.0258518516
## Bannykus.hp         0.079684645  0.096751415 -0.0067351645 -0.0261713344
## Beipiaosaurus.hp   -0.052534781  0.034350017 -0.0842504406  0.0839180116
## Eremotherium.hp    -0.049300410 -0.030517834  0.0090333190 -0.1134700993
## Erliansaurus.hp     0.027461874 -0.009579947  0.0227435709 -0.0291868954
## Falcarius.hp       -0.031790417  0.132103498 -0.0334331640  0.0034845962
## Guanlong.hp         0.011883189  0.072233516  0.0208750795  0.0330128132
## Haplocheirus.hp     0.075832908  0.041287620 -0.0673664404 -0.0811921909
## Linhenykus.hp       0.002408639  0.065621812  0.0560241892  0.0269285488
## Manis.hp           -0.045087797  0.010182431 -0.0064308351  0.0026513587
## Mononykus.hp       -0.010712689 -0.037897198  0.0210688406 -0.0181325963
## Nothronychus.hp    -0.077755660 -0.128332363  0.0794520034  0.0005151404
## Puma.hp            -0.023553504  0.016669352 -0.0117637905  0.0111677086
## Shishugounykus.hp  -0.024790850 -0.015732372 -0.0463772056  0.0048763618
## Tamandua.hp         0.034026147  0.022201701  0.0516128875  0.0686013929
## Therizinosaurus.hp  0.071952484  0.034137345  0.0509634709 -0.0297733746
## Tugulusaurus.hp     0.005312641  0.025200492  0.0188064271 -0.0184495309
##                             PC21          PC22          PC23          PC24
## Allosaurus.p        0.0367968840  0.0133667412 -0.0318069109  0.0218937120
## Alxasaurus.p        0.0007444127  0.0101766940 -0.0110465601  0.0135246966
## Aorun.p             0.0190576860  0.0009808852  0.0021340477  0.0105605457
## Bannykus.p          0.0054620217 -0.0032003555 -0.0250634251 -0.0129954378
## Beipiaosaurus.p    -0.1328784961 -0.0323385474 -0.0414622152  0.0275790455
## Eremotherium.p     -0.0075103341 -0.0823761485  0.0034820837 -0.0007426719
## Erliansaurus.p     -0.0463408604 -0.0328376125 -0.0580997904  0.0352928692
## Falcarius.p         0.0327337723  0.0037192445 -0.0010008004 -0.1081677422
## Guanlong.p          0.0124784976 -0.0380966786  0.0233328618  0.0395947313
## Haplocheirus.p     -0.0557183466  0.0352525741  0.0240248946 -0.0060526498
## Linhenykus.p        0.0110744493 -0.0212165553  0.0032059817  0.0026317188
## Manis.p            -0.0117578754  0.0633312658 -0.0321867830  0.0053649428
## Mononykus.p        -0.0095813093  0.0203546187  0.0207887594  0.0153615327
## Nothronychus.p     -0.0377660159 -0.0358789205  0.0322523088  0.0716613407
## Puma.p             -0.0128755539 -0.0008887828  0.0311442139 -0.0274947322
## Shishugounykus.p    0.0227516734  0.0134995938  0.0098104058  0.0064410480
## Tamandua.p          0.0323689044  0.0494287910 -0.0023609567  0.0500353550
## Therizinosaurus.p  -0.1123434662 -0.0074374127 -0.0202029372 -0.0132872753
## Tugulusaurus.p      0.0006013535 -0.0155923039 -0.0060360726  0.0216520064
## Allosaurus.sd       0.0487823813  0.0264871460 -0.0592212493 -0.0146160138
## Alxasaurus.sd       0.0105090295 -0.0091144883  0.0215975056 -0.0558529888
## Aorun.sd            0.0328359303 -0.0400355946 -0.0680832849  0.0464088310
## Bannykus.sd         0.0077687293  0.0862095200  0.0767804271  0.0163376659
## Beipiaosaurus.sd   -0.0841828030  0.0498880381  0.0711565021  0.0077289236
## Eremotherium.sd    -0.0002979911  0.0140988922  0.0041034580 -0.0155030949
## Erliansaurus.sd     0.0252044725 -0.0126261327  0.0685231057 -0.0178170018
## Falcarius.sd        0.0227769292  0.0275435664  0.0129121728 -0.0324996711
## Guanlong.sd         0.0591665648 -0.0682763867  0.0611508832 -0.0352824352
## Haplocheirus.sd     0.0645806904 -0.0459219808  0.0717663315  0.0945493872
## Linhenykus.sd       0.0110573433 -0.0212062003  0.0032106867  0.0026392637
## Manis.sd            0.0255262533 -0.0363795049 -0.0143721252  0.0133658444
## Mononykus.sd       -0.0096371103  0.0203897855  0.0209664385  0.0154148330
## Nothronychus.sd    -0.0518176955 -0.0738724510 -0.0462934296 -0.0184552737
## Puma.sd             0.0164522755  0.0132817846  0.0006643622  0.0328252292
## Shishugounykus.sd   0.0203659406 -0.0163239941  0.0532700751 -0.0051142433
## Tamandua.sd         0.0522511597  0.0479945417  0.0165185473 -0.0173887872
## Therizinosaurus.sd  0.0692390918  0.0034579398  0.0136841303  0.0083374258
## Tugulusaurus.sd     0.0072069176 -0.0129525477 -0.0136524032 -0.0447512464
## Allosaurus.hp       0.0307754855  0.0384330766 -0.0528649792  0.0135064226
## Alxasaurus.hp      -0.0291955932  0.0060966437  0.0300332229 -0.0011843611
## Aorun.hp           -0.0114300764  0.0180575827 -0.0326344569 -0.0113448373
## Bannykus.hp        -0.0722730115 -0.0422362888  0.0368462907 -0.0587676831
## Beipiaosaurus.hp    0.0405313404 -0.0208417871 -0.0143053223 -0.0179265556
## Eremotherium.hp     0.0020201532  0.0116198058 -0.0252957812  0.0255596759
## Erliansaurus.hp     0.0046202382  0.0105073249  0.0324379027 -0.0256178548
## Falcarius.hp        0.0279733571  0.0660690900 -0.0545965705 -0.0087767382
## Guanlong.hp        -0.0324739743  0.0301625929  0.0051612722  0.0022533525
## Haplocheirus.hp     0.1029887331 -0.0714033790 -0.0391992478 -0.0143548531
## Linhenykus.hp      -0.0439729748  0.0200009174  0.0010175792  0.0150231178
## Manis.hp            0.0017559865  0.0236845616 -0.0026749105 -0.0027713171
## Mononykus.hp       -0.0055399351 -0.0073855632  0.0060171774  0.0006088582
## Nothronychus.hp    -0.0049177549  0.0501637172 -0.0725162375 -0.0146223530
## Puma.hp            -0.0284288308 -0.0065731622 -0.0017946589 -0.0043987452
## Shishugounykus.hp  -0.0206756159  0.0353186509  0.0023198581  0.0103296391
## Tamandua.hp        -0.0666900109 -0.0559411262 -0.0024146780 -0.0205657866
## Therizinosaurus.hp  0.0417457386 -0.0172482344 -0.0239667905 -0.0062826874
## Tugulusaurus.hp    -0.0118987609  0.0186265534 -0.0071609098 -0.0138469767
##                             PC25          PC26          PC27          PC28
## Allosaurus.p        5.642818e-03  0.0050204919  0.0184211125  0.0099410571
## Alxasaurus.p        3.105046e-02 -0.0156716863  0.0078327196 -0.0001596976
## Aorun.p            -1.758109e-02 -0.0156602188 -0.0409235156  0.0473135840
## Bannykus.p         -7.759030e-02  0.0140516869  0.0497897248 -0.0215654362
## Beipiaosaurus.p    -1.822781e-02 -0.0236169068  0.0299787026  0.0196625642
## Eremotherium.p      1.824196e-02  0.0321357145  0.0322132329  0.0400244627
## Erliansaurus.p     -2.464524e-02 -0.0055782158 -0.0468307113  0.0023094393
## Falcarius.p        -2.020771e-02 -0.0481177897  0.0012866541 -0.0190025335
## Guanlong.p          3.562318e-05 -0.0085049565 -0.0382268677  0.0131088158
## Haplocheirus.p     -5.710142e-02 -0.0118442125  0.0569797500  0.0559296615
## Linhenykus.p       -1.987380e-02  0.0025563785 -0.0184029300 -0.0024027247
## Manis.p             3.444219e-02  0.0321345886  0.0226636416 -0.0185709946
## Mononykus.p         4.611982e-03  0.0276874639 -0.0102025980 -0.0285697070
## Nothronychus.p     -4.262579e-02 -0.0707282619 -0.0192639351 -0.0186944821
## Puma.p             -2.078125e-03  0.0109985896 -0.0008746969 -0.0055082244
## Shishugounykus.p    9.731275e-04  0.0041321066  0.0113056532 -0.0020311370
## Tamandua.p         -5.814542e-03 -0.0284442842  0.0203898315  0.0247537767
## Therizinosaurus.p  -1.492655e-02  0.0076660272  0.0024324773 -0.0016594017
## Tugulusaurus.p     -1.385068e-02  0.0001904058  0.0055191946 -0.0096589774
## Allosaurus.sd      -3.220285e-02 -0.0380084972  0.0042240394 -0.0287502110
## Alxasaurus.sd      -1.520232e-02 -0.0324335741 -0.0002571789  0.0465752797
## Aorun.sd            6.275468e-02  0.0219589749 -0.0403268746  0.0266575531
## Bannykus.sd        -7.712960e-02  0.0432219308 -0.0300034877 -0.0018718280
## Beipiaosaurus.sd    7.360556e-02 -0.0231413628 -0.0022794749 -0.0159451626
## Eremotherium.sd    -2.144085e-02  0.0217867460 -0.0224772550  0.0017043107
## Erliansaurus.sd     1.771031e-02 -0.0146665620 -0.0205431746  0.0145207174
## Falcarius.sd        6.698844e-02  0.0159743665  0.0093610276 -0.0066237146
## Guanlong.sd        -9.441349e-03 -0.0323791334  0.0160552660 -0.0594201148
## Haplocheirus.sd     1.501339e-02 -0.0180451763  0.0380870591 -0.0370776256
## Linhenykus.sd      -1.987639e-02  0.0025525738 -0.0184025692 -0.0024001882
## Manis.sd            1.624369e-02  0.0363583924  0.0090890951 -0.0033914830
## Mononykus.sd        4.722801e-03  0.0274682852 -0.0099505531 -0.0284037614
## Nothronychus.sd     2.132790e-02 -0.0349942604  0.0171567118 -0.0483013649
## Puma.sd            -5.227843e-03 -0.0395735633  0.0080578868  0.0024733757
## Shishugounykus.sd   2.974123e-02  0.0424792546 -0.0000378332  0.0403706927
## Tamandua.sd         3.062981e-02  0.0112232577 -0.0093588771 -0.0065205502
## Therizinosaurus.sd -2.351453e-03 -0.0054561798  0.0012270160  0.0115233913
## Tugulusaurus.sd     1.130823e-02 -0.0204404270 -0.0278626948  0.0196599991
## Allosaurus.hp       2.143775e-02 -0.0396793369 -0.0221861943  0.0024861141
## Alxasaurus.hp      -2.163263e-02  0.0265777838 -0.0270603369 -0.0148807928
## Aorun.hp            2.685957e-03 -0.0118569779 -0.0206773687 -0.0298539004
## Bannykus.hp         2.696605e-02 -0.0289790260 -0.0424556632  0.0233295203
## Beipiaosaurus.hp    2.696319e-02 -0.0081768908  0.0113229773 -0.0012654003
## Eremotherium.hp    -1.361555e-02  0.0297821364 -0.0427679232 -0.0219871419
## Erliansaurus.hp    -6.172008e-03  0.0150701395  0.0037984459 -0.0096102468
## Falcarius.hp        3.007014e-02 -0.0233501677  0.0361446873 -0.0067993685
## Guanlong.hp         1.272504e-04  0.0136884970  0.0147039774  0.0114402080
## Haplocheirus.hp    -4.769253e-02  0.0397478934  0.0216085416  0.0303717856
## Linhenykus.hp       4.209785e-02  0.0126491408  0.0426803110  0.0123186315
## Manis.hp           -2.400974e-02  0.0027004552  0.0185054387  0.0027346467
## Mononykus.hp        4.808222e-02 -0.0052378820  0.0220479420  0.0245753555
## Nothronychus.hp    -8.568848e-03  0.0211682548 -0.0304419165 -0.0110597651
## Puma.hp             4.317493e-03 -0.0094107909  0.0013490336 -0.0067869253
## Shishugounykus.hp  -2.767416e-02 -0.0152146896  0.0027191798  0.0234781525
## Tamandua.hp        -6.807072e-03  0.0912459232  0.0108369085 -0.0343213576
## Therizinosaurus.hp  2.101478e-03  0.0092368178 -0.0043715863  0.0098453615
## Tugulusaurus.hp     3.674653e-03  0.0077467534 -0.0016020226 -0.0140142372
##                             PC29          PC30          PC31          PC32
## Allosaurus.p       -0.0309438765 -0.0313284451  0.0077000854 -0.0296198479
## Alxasaurus.p       -0.0088164773 -0.0131197326  0.0045094048  0.0059425737
## Aorun.p            -0.0114471358  0.0169134526 -0.0136594092  0.0033824538
## Bannykus.p         -0.0035640623 -0.0177266027 -0.0028903514  0.0019343084
## Beipiaosaurus.p    -0.0197661055 -0.0081519140 -0.0203134258  0.0212559168
## Eremotherium.p     -0.0046586573  0.0269936242 -0.0004358998  0.0095398569
## Erliansaurus.p     -0.0299681842 -0.0251699103 -0.0162831587 -0.0200408785
## Falcarius.p         0.0354555448  0.0144458886  0.0205367197 -0.0268730533
## Guanlong.p          0.0107380139 -0.0140129433 -0.0158299787 -0.0116868280
## Haplocheirus.p      0.0090176653 -0.0119864647  0.0357888410  0.0075494984
## Linhenykus.p        0.0005648327 -0.0087649870 -0.0080982781  0.0139875215
## Manis.p            -0.0027264021  0.0117409591 -0.0096078484 -0.0029189705
## Mononykus.p        -0.0140617871  0.0068899394 -0.0012120558 -0.0082887878
## Nothronychus.p     -0.0056885633  0.0443791729 -0.0080593522 -0.0139096040
## Puma.p              0.0124453425  0.0253588256 -0.0005645877  0.0266963822
## Shishugounykus.p    0.0059200172 -0.0031942532  0.0122728951  0.0009418265
## Tamandua.p          0.0139657887 -0.0119053220  0.0040328728 -0.0052439387
## Therizinosaurus.p  -0.0096349144 -0.0030627203  0.0021348992 -0.0108410363
## Tugulusaurus.p      0.0227651887 -0.0114723306  0.0146632659  0.0113743936
## Allosaurus.sd       0.0419990734  0.0264796421 -0.0001010705  0.0504760672
## Alxasaurus.sd      -0.0447713063 -0.0011610947 -0.0134230723 -0.0133799639
## Aorun.sd           -0.0017498842  0.0248053913  0.0653716212  0.0011462488
## Bannykus.sd        -0.0130830364 -0.0309662484  0.0263076917 -0.0099619640
## Beipiaosaurus.sd    0.0331767018  0.0070065574  0.0148420780 -0.0248620125
## Eremotherium.sd     0.0459876045 -0.0221063533  0.0296957236 -0.0002919214
## Erliansaurus.sd    -0.0017429711  0.0150316371  0.0024487262  0.0278146774
## Falcarius.sd       -0.0296195490 -0.0032664713 -0.0248588999  0.0244761003
## Guanlong.sd        -0.0802456818 -0.0103122634  0.0233774391  0.0042750766
## Haplocheirus.sd     0.0295405918  0.0067242575 -0.0109416961 -0.0106833199
## Linhenykus.sd       0.0005688609 -0.0087596155 -0.0080994754  0.0139751719
## Manis.sd           -0.0054011476 -0.0206624085 -0.0132555614  0.0291709112
## Mononykus.sd       -0.0139480506  0.0071100523 -0.0014052302 -0.0084613376
## Nothronychus.sd     0.0356231085 -0.0166585915  0.0196012666 -0.0234123406
## Puma.sd             0.0125191970 -0.0086827807 -0.0156781634  0.0175784267
## Shishugounykus.sd   0.0354451561 -0.0026435753 -0.0231734992 -0.0274308911
## Tamandua.sd        -0.0232407342  0.0081178632  0.0054609436  0.0067736327
## Therizinosaurus.sd  0.0008732107  0.0068095772 -0.0118065340  0.0054421715
## Tugulusaurus.sd    -0.0120163101 -0.0132670949 -0.0055008681 -0.0236444030
## Allosaurus.hp      -0.0223431434 -0.0150844479  0.0037753073  0.0040228152
## Alxasaurus.hp       0.0068241401 -0.0002469539 -0.0308328953  0.0049610339
## Aorun.hp           -0.0147368311  0.0043420149  0.0169890350  0.0032644315
## Bannykus.hp         0.0124046947 -0.0055624278  0.0020258407  0.0208079876
## Beipiaosaurus.hp    0.0385502745 -0.0749033957 -0.0383698487  0.0006047555
## Eremotherium.hp    -0.0027457705 -0.0082947391  0.0230919649  0.0139958493
## Erliansaurus.hp     0.0081053702  0.0202931739 -0.0233761730 -0.0089582337
## Falcarius.hp       -0.0249876963  0.0205838431 -0.0202549285 -0.0259737596
## Guanlong.hp         0.0021494579 -0.0021269171  0.0168645567  0.0006541375
## Haplocheirus.hp     0.0119390955  0.0356360739  0.0074752300 -0.0287646272
## Linhenykus.hp      -0.0183462764  0.0176515281  0.0049029416  0.0058730185
## Manis.hp           -0.0001050664  0.0014998395  0.0078254903  0.0179333504
## Mononykus.hp        0.0024131033 -0.0210773884  0.0242471322  0.0095019911
## Nothronychus.hp     0.0256654024  0.0272634993 -0.0332249493 -0.0061120476
## Puma.hp             0.0043735314  0.0179679005 -0.0047246644 -0.0083290636
## Shishugounykus.hp  -0.0070789535  0.0306012560 -0.0128491649 -0.0053728641
## Tamandua.hp        -0.0076133741  0.0127322872 -0.0047954566 -0.0022666944
## Therizinosaurus.hp  0.0031021297 -0.0080322132 -0.0027939525  0.0024585070
## Tugulusaurus.hp     0.0029188509 -0.0036676503  0.0004784771 -0.0104827043
##                             PC33          PC34          PC35          PC36
## Allosaurus.p        0.0284838331 -0.0285567261  1.127436e-02 -6.450317e-03
## Alxasaurus.p        0.0144269473 -0.0108404439 -5.005705e-03  1.827113e-02
## Aorun.p            -0.0195882722 -0.0103132898 -1.350059e-03 -1.836084e-02
## Bannykus.p          0.0031117830  0.0116414263  3.492142e-03  1.040759e-02
## Beipiaosaurus.p     0.0172545258 -0.0008280636 -2.097676e-02 -1.519112e-02
## Eremotherium.p     -0.0194684627  0.0015824926  1.918385e-02  3.200412e-03
## Erliansaurus.p     -0.0118349608 -0.0064044870 -4.075455e-04  3.601845e-02
## Falcarius.p        -0.0150345569 -0.0148154308 -1.431289e-02  2.398833e-03
## Guanlong.p          0.0103198847  0.0312351254 -3.711597e-02 -4.494176e-03
## Haplocheirus.p     -0.0229790960 -0.0137055605 -2.128089e-02 -7.892678e-03
## Linhenykus.p        0.0117082085  0.0033380735  6.106519e-03 -3.821720e-03
## Manis.p            -0.0130614181  0.0354237699 -2.193660e-03 -1.256442e-02
## Mononykus.p        -0.0160661867 -0.0140565625 -8.888951e-03 -7.728182e-03
## Nothronychus.p     -0.0018886578 -0.0026222225  4.805787e-05 -1.128397e-02
## Puma.p             -0.0105305059  0.0167487773  1.699289e-02  1.566156e-02
## Shishugounykus.p   -0.0133480729  0.0072919812 -1.034952e-02  1.184207e-02
## Tamandua.p         -0.0061690884  0.0160570327  8.080570e-03  7.267164e-03
## Therizinosaurus.p  -0.0043542666 -0.0033197366  5.859454e-03  2.752411e-03
## Tugulusaurus.p     -0.0219576959  0.0152899590 -3.074481e-03  1.010839e-02
## Allosaurus.sd       0.0380204783 -0.0146601295 -2.535403e-02  5.698664e-03
## Alxasaurus.sd       0.0026382565  0.0255374717  5.628205e-03  1.036011e-02
## Aorun.sd           -0.0017655558  0.0135057121 -7.026693e-03 -1.248232e-05
## Bannykus.sd         0.0142329081  0.0071474838  4.121481e-03  2.073609e-03
## Beipiaosaurus.sd    0.0187848780  0.0025835600  1.730689e-02 -2.907799e-03
## Eremotherium.sd    -0.0039501529  0.0034251039 -3.458517e-03 -1.109823e-02
## Erliansaurus.sd     0.0012643602 -0.0234446210  1.286009e-02 -1.403147e-02
## Falcarius.sd        0.0067001265  0.0041125262 -1.978415e-02 -6.171739e-03
## Guanlong.sd        -0.0020492652  0.0113617016 -1.448892e-02 -2.092331e-03
## Haplocheirus.sd     0.0132338924 -0.0094392144  1.008676e-02  1.669928e-02
## Linhenykus.sd       0.0117090319  0.0033372131  6.106519e-03 -3.818495e-03
## Manis.sd           -0.0103099682 -0.0070595752 -3.874564e-03 -1.226807e-02
## Mononykus.sd       -0.0162521167 -0.0141928293 -8.793845e-03 -7.737480e-03
## Nothronychus.sd    -0.0078362573  0.0061445084 -1.377944e-03 -1.258646e-02
## Puma.sd            -0.0360470017 -0.0054337683  1.910665e-02 -2.653357e-02
## Shishugounykus.sd   0.0020883456 -0.0071677923 -3.652345e-02  2.057839e-03
## Tamandua.sd        -0.0080333471 -0.0033212278 -4.650783e-03  5.608634e-03
## Therizinosaurus.sd -0.0003684639 -0.0004943154 -1.509692e-03  1.249365e-03
## Tugulusaurus.sd     0.0250136168 -0.0091444110  5.145968e-03 -1.806519e-02
## Allosaurus.hp      -0.0063747743 -0.0201001268  1.951036e-02 -5.110369e-04
## Alxasaurus.hp       0.0121355860  0.0201981996 -5.523860e-04 -1.035619e-02
## Aorun.hp           -0.0043020645  0.0314104425  1.482953e-02 -1.221056e-02
## Bannykus.hp         0.0107795924 -0.0022914240  1.090523e-02  2.016088e-02
## Beipiaosaurus.hp   -0.0084622731  0.0000098691  1.308959e-02 -6.879674e-04
## Eremotherium.hp    -0.0061264865 -0.0235061286 -9.386621e-03  9.088107e-03
## Erliansaurus.hp    -0.0164245294 -0.0066332926  1.030289e-02  1.163405e-02
## Falcarius.hp       -0.0054314803 -0.0028056162 -9.703248e-03  1.377053e-03
## Guanlong.hp         0.0319070220  0.0049114670  1.290660e-02 -1.677271e-02
## Haplocheirus.hp     0.0221683232  0.0054369560  1.423283e-02 -9.657571e-03
## Linhenykus.hp       0.0177612531 -0.0010437104  3.014690e-06  5.981971e-03
## Manis.hp            0.0152453295  0.0063523862  2.065592e-02  8.226734e-03
## Mononykus.hp       -0.0037649555  0.0014124392 -3.810335e-03  1.578858e-02
## Nothronychus.hp     0.0017887767  0.0051728452  6.027633e-03  7.729366e-03
## Puma.hp             0.0045259256  0.0053513229 -9.164408e-03  7.754581e-03
## Shishugounykus.hp  -0.0073519329 -0.0091488148 -2.491610e-03  1.390689e-02
## Tamandua.hp         0.0001805210 -0.0246429117  6.461525e-03 -1.338951e-03
## Therizinosaurus.hp -0.0064791263  0.0044455676  5.804503e-03 -1.903119e-03
## Tugulusaurus.hp    -0.0078724136 -0.0104729810  7.775934e-04 -4.774870e-03
##                             PC37          PC38          PC39          PC40
## Allosaurus.p        0.0015210254  0.0109858542  1.681679e-02 -0.0098256986
## Alxasaurus.p        0.0073564166 -0.0152227665 -4.473132e-03  0.0171651699
## Aorun.p             0.0099368087  0.0131603270 -2.388917e-03  0.0046990344
## Bannykus.p         -0.0046332002  0.0201842946 -1.198131e-02  0.0056672025
## Beipiaosaurus.p    -0.0080795016 -0.0063011032 -2.721147e-03 -0.0077245770
## Eremotherium.p     -0.0107235931 -0.0094097868  1.062051e-02 -0.0034004466
## Erliansaurus.p     -0.0114443271 -0.0014107118  1.741173e-02  0.0036506210
## Falcarius.p         0.0112272360  0.0119154988  1.726055e-02  0.0058322000
## Guanlong.p          0.0091771850 -0.0031072062  1.108547e-02  0.0143759203
## Haplocheirus.p      0.0084445049 -0.0083752025  4.000698e-03 -0.0007085811
## Linhenykus.p        0.0023398259 -0.0013483815  8.452484e-03 -0.0027329371
## Manis.p             0.0046081731  0.0231629995  8.708630e-03 -0.0176282829
## Mononykus.p        -0.0089965524 -0.0221128549 -3.034338e-03 -0.0016529467
## Nothronychus.p      0.0084301347  0.0079080922 -2.566113e-03 -0.0071658018
## Puma.p             -0.0121071050 -0.0127471053  3.471213e-03 -0.0146514849
## Shishugounykus.p   -0.0112001697  0.0052148761 -1.430916e-02 -0.0027112907
## Tamandua.p          0.0087853519 -0.0147360791  2.371475e-03 -0.0054231055
## Therizinosaurus.p  -0.0014654096 -0.0010401834 -2.388105e-05  0.0015064497
## Tugulusaurus.p      0.0071760169  0.0010716725  4.084271e-03 -0.0071742803
## Allosaurus.sd      -0.0172189273 -0.0063279942 -2.689860e-03 -0.0008688276
## Alxasaurus.sd      -0.0016281265 -0.0022020413 -1.638461e-02  0.0003777225
## Aorun.sd            0.0053684556 -0.0013887251 -4.645442e-03  0.0038366245
## Bannykus.sd        -0.0069926623  0.0007441549 -1.151615e-02  0.0022600554
## Beipiaosaurus.sd    0.0016241576 -0.0018606968  3.139596e-03  0.0052425395
## Eremotherium.sd    -0.0028860768 -0.0122320728  1.555691e-02 -0.0006191340
## Erliansaurus.sd    -0.0007391998 -0.0078243179  1.468861e-03 -0.0061755735
## Falcarius.sd       -0.0042310746  0.0097124784  1.362176e-02  0.0009054272
## Guanlong.sd         0.0137292953 -0.0070620166  1.046674e-02 -0.0041735886
## Haplocheirus.sd    -0.0119684603  0.0089543436  1.802165e-03  0.0019774698
## Linhenykus.sd       0.0023407777 -0.0013466352  8.458165e-03 -0.0027252692
## Manis.sd            0.0175942741  0.0016825732 -1.257889e-02  0.0187696497
## Mononykus.sd       -0.0092693123 -0.0220638455 -3.182170e-03 -0.0017049240
## Nothronychus.sd     0.0074338415 -0.0025280719 -1.704365e-02 -0.0028826488
## Puma.sd            -0.0178936948  0.0180119233  1.709381e-03  0.0152837143
## Shishugounykus.sd  -0.0144663783  0.0097505245 -1.638618e-02 -0.0076191827
## Tamandua.sd         0.0015640879  0.0062728031 -1.376754e-03  0.0005262447
## Therizinosaurus.sd  0.0021893895  0.0001860945  2.191323e-03 -0.0009856353
## Tugulusaurus.sd    -0.0240303187  0.0005413950 -3.733911e-03  0.0010468609
## Allosaurus.hp       0.0162686590  0.0006643274 -1.585047e-02 -0.0118209102
## Alxasaurus.hp       0.0128487442 -0.0016024736 -3.712453e-03 -0.0066476896
## Aorun.hp           -0.0367951206 -0.0020569702 -2.226241e-03  0.0115938095
## Bannykus.hp         0.0001543497  0.0115956933 -1.004910e-02 -0.0038025370
## Beipiaosaurus.hp    0.0058275521 -0.0141167302  2.247239e-03 -0.0084847966
## Eremotherium.hp     0.0040209991  0.0158071498 -9.363240e-03 -0.0129432845
## Erliansaurus.hp     0.0173360678 -0.0045530870 -8.724972e-03  0.0104272458
## Falcarius.hp       -0.0104555887 -0.0117018596 -7.243492e-03  0.0002853863
## Guanlong.hp         0.0128575588  0.0033110904  1.168381e-02  0.0038652622
## Haplocheirus.hp    -0.0019190700 -0.0066868987 -4.288476e-03 -0.0031922485
## Linhenykus.hp       0.0179966537 -0.0024489638 -6.591996e-03  0.0042399325
## Manis.hp            0.0089682825  0.0023982168 -4.482402e-03  0.0098745075
## Mononykus.hp       -0.0148123519  0.0147245184  1.004619e-02  0.0016239578
## Nothronychus.hp     0.0187460997 -0.0056601504  9.596783e-03  0.0036499667
## Puma.hp             0.0103360971  0.0072660369 -6.840972e-03 -0.0029863677
## Shishugounykus.hp  -0.0067170331  0.0013444683  1.771745e-02  0.0026482967
## Tamandua.hp        -0.0010623944  0.0088070713  5.382340e-03  0.0059196363
## Therizinosaurus.hp  0.0027191291 -0.0028565382 -2.355018e-03 -0.0004268180
## Tugulusaurus.hp    -0.0071915019 -0.0030470078 -6.608076e-03  0.0016079611
##                             PC41          PC42          PC43          PC44
## Allosaurus.p        1.084731e-02  0.0048645909 -7.940298e-03 -3.540765e-03
## Alxasaurus.p       -5.108702e-03 -0.0138824625 -8.910246e-03  1.073355e-03
## Aorun.p             9.054972e-03 -0.0064534645  1.742969e-03  1.058581e-02
## Bannykus.p         -3.875028e-05 -0.0051551209  1.820798e-03  2.000245e-03
## Beipiaosaurus.p     5.277330e-03 -0.0031141679 -3.676076e-03 -6.740794e-04
## Eremotherium.p     -1.928994e-02  0.0089843269  2.549426e-03  4.831736e-03
## Erliansaurus.p     -5.829224e-03  0.0001599354  2.784756e-04  9.154319e-03
## Falcarius.p        -9.401392e-03 -0.0048079153  5.167959e-05  3.552913e-03
## Guanlong.p          8.346964e-05  0.0032413666  2.928458e-04 -5.031369e-03
## Haplocheirus.p     -2.670376e-03 -0.0051766889 -2.420855e-03  3.454561e-04
## Linhenykus.p       -3.946931e-03  0.0011141396  8.608954e-05 -8.680034e-04
## Manis.p            -3.514064e-03 -0.0056836795 -5.401805e-03  2.535859e-04
## Mononykus.p        -6.969672e-04 -0.0038497671  6.238213e-03  2.663778e-03
## Nothronychus.p     -1.111752e-04  0.0003782620  5.478552e-04 -1.550191e-03
## Puma.p              1.481626e-02 -0.0184571782 -1.113740e-02  2.209170e-03
## Shishugounykus.p    1.281954e-02  0.0072745917 -1.019601e-03  6.463654e-03
## Tamandua.p         -1.846657e-03 -0.0028533382 -1.564336e-03  8.430675e-04
## Therizinosaurus.p  -9.117144e-04  0.0008412247  8.291077e-04 -1.325471e-03
## Tugulusaurus.p      6.126063e-03  0.0099657153  3.164893e-03 -9.290639e-03
## Allosaurus.sd      -4.863764e-03  0.0042202465  1.706333e-03 -2.717775e-04
## Alxasaurus.sd       2.003798e-04  0.0004152458  7.022923e-03 -7.000918e-03
## Aorun.sd           -1.430968e-03 -0.0044669652 -1.413512e-03 -1.327243e-03
## Bannykus.sd        -4.288777e-03  0.0028902488 -2.355594e-04 -3.989080e-04
## Beipiaosaurus.sd    1.079307e-03  0.0009903793  3.022480e-04 -1.045369e-04
## Eremotherium.sd     3.023059e-03  0.0007377906 -2.343386e-03  2.381312e-03
## Erliansaurus.sd     3.128892e-03  0.0112504856  3.507047e-03 -4.936225e-03
## Falcarius.sd       -1.987541e-03  0.0011801293  2.147165e-03  6.246514e-03
## Guanlong.sd         4.052347e-03  0.0022547339 -4.275041e-03  1.868238e-03
## Haplocheirus.sd     9.577127e-04 -0.0085090169  2.613715e-03  2.278332e-03
## Linhenykus.sd      -3.941503e-03  0.0011181408  7.890431e-05 -8.713519e-04
## Manis.sd            1.346082e-02 -0.0040995043  7.931940e-04 -2.560081e-03
## Mononykus.sd       -4.939082e-04 -0.0038914372  6.311190e-03  2.692343e-03
## Nothronychus.sd     2.164167e-03  0.0049183286  5.823330e-04  1.252878e-04
## Puma.sd            -9.238251e-03 -0.0015710767 -5.833716e-03 -2.246535e-03
## Shishugounykus.sd   2.048561e-03  0.0086443421 -4.854748e-03  6.016491e-03
## Tamandua.sd        -4.927249e-03  0.0003798109  3.078870e-06 -1.214641e-03
## Therizinosaurus.sd  2.139191e-03 -0.0017038029 -1.195587e-03 -3.743176e-05
## Tugulusaurus.sd    -4.365543e-03 -0.0095121045 -9.203901e-03 -4.291064e-03
## Allosaurus.hp      -2.386950e-03  0.0007296395  5.310800e-03  4.422065e-03
## Alxasaurus.hp      -1.885394e-02 -0.0092009170  6.164341e-03 -4.207737e-03
## Aorun.hp            3.457352e-03  0.0115357875 -5.563237e-03  2.450761e-03
## Bannykus.hp         5.045180e-03 -0.0027905321  7.352170e-04  9.851828e-04
## Beipiaosaurus.hp   -1.990646e-03  0.0048486333 -7.215640e-04  1.174107e-03
## Eremotherium.hp    -9.390305e-03  0.0005406645 -5.396810e-03 -6.466867e-03
## Erliansaurus.hp     8.873150e-04  0.0025030610 -1.211673e-02 -7.239955e-03
## Falcarius.hp        1.215519e-03 -0.0016224178  7.908643e-03 -4.824807e-03
## Guanlong.hp         8.723552e-03 -0.0020419783  1.040429e-02  7.608644e-03
## Haplocheirus.hp    -3.460736e-04 -0.0039097298  7.929658e-04 -1.104870e-03
## Linhenykus.hp      -1.299477e-02  0.0103666400 -1.180011e-02  3.298330e-04
## Manis.hp           -2.362509e-03  0.0094635574  4.396735e-03  7.700710e-03
## Mononykus.hp        5.604953e-04 -0.0086624234  1.519352e-02 -1.125552e-02
## Nothronychus.hp     9.793285e-03  0.0018433594  8.338908e-04 -2.326261e-03
## Puma.hp            -1.909729e-04  0.0031842354  3.717059e-03  7.983713e-03
## Shishugounykus.hp   9.998882e-03  0.0109784864 -1.176055e-03 -7.286528e-03
## Tamandua.hp         3.555657e-03 -0.0008969915  2.757291e-03 -3.230329e-03
## Therizinosaurus.hp  2.158488e-03  0.0009860932  4.522977e-04  5.352554e-05
## Tugulusaurus.hp     7.444573e-04 -0.0004915122  6.863034e-03 -2.810043e-03
##                             PC45          PC46          PC47          PC48
## Allosaurus.p        6.624542e-03 -6.525368e-03 -6.096825e-04 -0.0015574121
## Alxasaurus.p        3.903093e-03 -5.479834e-03  1.293516e-04  0.0011076212
## Aorun.p             1.840229e-03  3.590639e-03 -6.673128e-03 -0.0020387324
## Bannykus.p          4.283193e-03 -1.131931e-03  2.866066e-03 -0.0006119127
## Beipiaosaurus.p    -1.868381e-03 -1.491033e-03  3.583864e-04  0.0012698687
## Eremotherium.p     -2.113237e-03 -2.893137e-05  1.255879e-04  0.0003870840
## Erliansaurus.p      1.011953e-03  2.365029e-03 -5.950427e-04  0.0005263996
## Falcarius.p         6.243131e-03 -2.060305e-04 -7.638733e-04  0.0013734821
## Guanlong.p          9.082590e-04 -3.159623e-03  2.197198e-04 -0.0015523949
## Haplocheirus.p     -1.552863e-03 -1.169299e-03  6.878114e-04 -0.0006698116
## Linhenykus.p        2.055674e-03  9.428677e-04  1.859308e-03 -0.0010142143
## Manis.p            -4.013802e-03 -4.028277e-03  1.090574e-03  0.0014420370
## Mononykus.p         2.194458e-03 -1.669281e-03  2.647714e-03  0.0011921524
## Nothronychus.p     -1.335376e-03  1.960228e-04 -2.639509e-03  0.0003568192
## Puma.p              5.713947e-03  4.811244e-04 -3.846346e-03 -0.0003260397
## Shishugounykus.p   -9.759933e-04  2.943972e-03  4.796850e-03 -0.0031015776
## Tamandua.p         -2.813028e-03 -9.323126e-05 -8.929414e-04  0.0010658563
## Therizinosaurus.p   6.340769e-04  1.821720e-04 -3.330767e-04 -0.0001017420
## Tugulusaurus.p      9.504945e-03  4.572704e-03 -4.261080e-04  0.0011630569
## Allosaurus.sd      -2.973339e-04  1.055581e-03 -1.553903e-04 -0.0000217908
## Alxasaurus.sd       9.930819e-03 -6.713735e-04  1.833680e-03  0.0017086353
## Aorun.sd            2.786447e-04 -1.718711e-03  2.573253e-03  0.0012307736
## Bannykus.sd        -2.977794e-03  9.277690e-04 -1.047933e-03 -0.0010913913
## Beipiaosaurus.sd    3.731217e-03  4.360014e-04 -6.461020e-04 -0.0009178709
## Eremotherium.sd    -4.158799e-03  5.202754e-04  1.021875e-03 -0.0041609965
## Erliansaurus.sd     3.523750e-03 -4.288332e-03 -2.344165e-04 -0.0007176123
## Falcarius.sd        8.229388e-04  6.510969e-04  4.998822e-04  0.0004450297
## Guanlong.sd        -6.283668e-03  1.286486e-03  7.883498e-05 -0.0003779101
## Haplocheirus.sd    -4.772922e-04  1.540236e-03  1.189047e-03  0.0003429699
## Linhenykus.sd       2.054743e-03  9.395452e-04  1.855795e-03 -0.0010112711
## Manis.sd           -2.869396e-04  1.772287e-03 -1.756805e-03  0.0009659593
## Mononykus.sd        2.359969e-03 -1.627365e-03  2.538049e-03  0.0012377750
## Nothronychus.sd    -1.149579e-03 -5.085350e-04 -1.177964e-03  0.0009966163
## Puma.sd             2.766890e-03 -1.317029e-03  4.804211e-03 -0.0008354926
## Shishugounykus.sd   1.732435e-03 -1.487900e-03 -8.824439e-04  0.0023254153
## Tamandua.sd        -8.033859e-04  3.887676e-04 -2.648903e-04  0.0017089169
## Therizinosaurus.sd  6.641672e-06 -1.652692e-04  1.236388e-04  0.0001291019
## Tugulusaurus.sd    -7.583748e-03  5.941506e-03  3.396286e-04  0.0010004554
## Allosaurus.hp      -2.342753e-03 -2.732385e-04  3.825028e-03 -0.0019013375
## Alxasaurus.hp       1.035269e-03 -7.124773e-04 -6.638220e-04 -0.0013761703
## Aorun.hp            1.500422e-03 -9.882242e-04 -3.206981e-03  0.0003537375
## Bannykus.hp        -3.821997e-03 -2.460204e-03  4.345480e-03 -0.0009618883
## Beipiaosaurus.hp   -1.791880e-03  1.503109e-03 -2.355850e-03  0.0012657692
## Eremotherium.hp     1.120697e-03  2.143819e-03 -3.030431e-03  0.0030128067
## Erliansaurus.hp    -1.001761e-02  9.010674e-04  1.576897e-03  0.0016113834
## Falcarius.hp       -6.503162e-04  2.082614e-03 -2.480880e-03 -0.0036354831
## Guanlong.hp         6.970586e-04  7.216084e-03  3.495766e-03  0.0058231533
## Haplocheirus.hp    -1.342653e-03 -2.557739e-04  2.193691e-04 -0.0004796775
## Linhenykus.hp       5.795758e-03  7.803524e-03 -1.447699e-03 -0.0034822539
## Manis.hp           -5.017118e-03 -7.346016e-03 -6.227954e-03  0.0022919451
## Mononykus.hp       -7.494565e-03  7.831271e-04 -3.948127e-03 -0.0022108982
## Nothronychus.hp    -7.159069e-04 -4.202219e-04  1.976428e-03 -0.0012494929
## Puma.hp            -4.321931e-03 -2.746116e-03  5.359272e-04 -0.0030747956
## Shishugounykus.hp  -5.858606e-03  2.441113e-04  2.230240e-03  0.0030861608
## Tamandua.hp         8.643687e-04 -5.576946e-04 -3.845119e-04 -0.0025276493
## Therizinosaurus.hp -3.339097e-04 -2.247945e-05 -9.035045e-05 -0.0004494744
## Tugulusaurus.hp    -7.386566e-04 -8.617376e-04 -3.062142e-03  0.0020363120
##                             PC49          PC50
## Allosaurus.p       -1.336220e-03  1.351776e-10
## Alxasaurus.p        1.618256e-03 -2.903305e-10
## Aorun.p             6.138220e-04 -5.228046e-11
## Bannykus.p         -6.673481e-04 -1.219333e-10
## Beipiaosaurus.p    -1.160663e-04  7.958918e-11
## Eremotherium.p     -1.490209e-04  9.106028e-12
## Erliansaurus.p     -2.850655e-04  1.387477e-10
## Falcarius.p        -4.022568e-04 -5.660601e-11
## Guanlong.p          1.520281e-04  2.194726e-10
## Haplocheirus.p     -1.128245e-03 -4.468290e-11
## Linhenykus.p       -6.968842e-04 -7.705725e-11
## Manis.p             1.440323e-03 -4.357839e-11
## Mononykus.p         4.656267e-04  2.708504e-11
## Nothronychus.p      2.274928e-04 -1.201670e-11
## Puma.p             -6.103335e-04  1.511509e-10
## Shishugounykus.p    6.537423e-04  1.064969e-10
## Tamandua.p         -1.027848e-04 -4.596501e-11
## Therizinosaurus.p   7.532446e-05  2.847034e-11
## Tugulusaurus.p      8.334879e-05  8.005648e-11
## Allosaurus.sd      -1.422184e-04  1.017121e-10
## Alxasaurus.sd       3.006645e-04  4.061149e-11
## Aorun.sd           -1.070909e-03  3.780137e-11
## Bannykus.sd         1.191478e-03  1.623887e-11
## Beipiaosaurus.sd    2.399374e-04 -1.217092e-11
## Eremotherium.sd     1.471791e-04 -3.331405e-11
## Erliansaurus.sd     1.624447e-04 -5.680395e-11
## Falcarius.sd        8.474676e-05  4.083817e-11
## Guanlong.sd         6.177882e-04  1.169755e-11
## Haplocheirus.sd    -5.614436e-04 -7.312023e-11
## Linhenykus.sd      -7.023780e-04  6.316214e-11
## Manis.sd           -4.146137e-04 -2.024283e-10
## Mononykus.sd        6.202422e-04  7.894367e-11
## Nothronychus.sd     6.270342e-04  3.527648e-11
## Puma.sd             1.251735e-03  2.643854e-10
## Shishugounykus.sd  -9.791903e-04 -3.097843e-11
## Tamandua.sd        -3.319724e-04  4.505967e-11
## Therizinosaurus.sd -2.548016e-04 -3.919398e-11
## Tugulusaurus.sd     1.653352e-04  3.757361e-11
## Allosaurus.hp      -1.501002e-03 -1.321065e-10
## Alxasaurus.hp      -1.852985e-03 -2.019616e-10
## Aorun.hp           -1.963465e-03 -3.522125e-10
## Bannykus.hp         1.185678e-03  4.012250e-11
## Beipiaosaurus.hp    8.621091e-04 -1.296784e-10
## Eremotherium.hp     1.026888e-03  1.194815e-11
## Erliansaurus.hp    -2.650454e-03  3.021414e-10
## Falcarius.hp       -9.792542e-05  1.689631e-10
## Guanlong.hp        -5.580301e-04  1.144352e-10
## Haplocheirus.hp     1.833036e-03 -2.648962e-11
## Linhenykus.hp       9.649174e-04 -3.666953e-11
## Manis.hp            5.521725e-04  3.426840e-10
## Mononykus.hp       -1.501764e-04  5.811199e-12
## Nothronychus.hp     8.690390e-04  4.627033e-11
## Puma.hp            -5.657101e-04 -1.862599e-10
## Shishugounykus.hp   1.009151e-03 -4.972793e-10
## Tamandua.hp         1.464790e-04 -3.756623e-11
## Therizinosaurus.hp  1.108389e-04 -4.141892e-11
## Tugulusaurus.hp    -7.359950e-06  5.307529e-11
```

```
Coord.p<-ind.coord[1:19, 1:2]
Coord.s<-ind.coord[20:38, 1:2]
Coord.hp<-ind.coord[39:57, 1:2]
Coord<-ind.coord[1:57, 1:2]
Coord.p
```

```
##                           PC1        PC2
## Allosaurus.p      -3.34322735  1.5024415
## Alxasaurus.p      -4.25547189  3.8567721
## Aorun.p           -2.53687163  0.1436204
## Bannykus.p         0.95231302 -2.6743476
## Beipiaosaurus.p    3.81786982 -4.0764147
## Eremotherium.p    -0.86870155 -1.4597797
## Erliansaurus.p    -1.31118643 -2.7903685
## Falcarius.p       -0.05572564 -1.9047215
## Guanlong.p        -2.45530339  0.2594165
## Haplocheirus.p    -1.30671697 -1.4332262
## Linhenykus.p      -4.41812298  4.2021227
## Manis.p           -2.90212515 -0.3406726
## Mononykus.p       -3.56290532  1.4871996
## Nothronychus.p     1.75663273 -3.5751840
## Puma.p            -3.33456905  1.8648867
## Shishugounykus.p  -3.94190947  3.1544965
## Tamandua.p         3.45214163 -6.2439935
## Therizinosaurus.p 21.89583870  6.1195515
## Tugulusaurus.p    -3.13483590  2.0373422
```

```
Coord.s
```

```
##                           PC1        PC2
## Allosaurus.sd       2.4086445 -2.2084409
## Alxasaurus.sd      -0.9553253 -3.3378570
## Aorun.sd            2.4815255 -2.3874001
## Bannykus.sd         4.7883633 -3.8180150
## Beipiaosaurus.sd    4.8798236 -3.9494467
## Eremotherium.sd    -0.3050740 -1.1973785
## Erliansaurus.sd     0.1382769 -2.3141258
## Falcarius.sd        4.2261804 -3.9608883
## Guanlong.sd        -0.3737679  0.4249703
## Haplocheirus.sd     2.1163420 -1.4224292
## Linhenykus.sd      -4.4181251  4.2020874
## Manis.sd           -1.8587151 -2.9528135
## Mononykus.sd       -3.5629089  1.4872153
## Nothronychus.sd     0.3103920  0.9309279
## Puma.sd            -2.1327064  2.1343638
## Shishugounykus.sd  -1.1599824  0.7198907
## Tamandua.sd         9.7469621 -4.0015429
## Therizinosaurus.sd 21.2672994  7.3602389
## Tugulusaurus.sd    -2.1512056  1.8696094
```

```
Coord.hp
```

```
##                           PC1        PC2
## Allosaurus.hp      -2.0475045 -1.4825623
## Alxasaurus.hp      -3.7977112  2.9332823
## Aorun.hp           -2.7466871 -1.5153310
## Bannykus.hp        -2.0891062 -1.9456923
## Beipiaosaurus.hp   -0.9174517 -3.2212909
## Eremotherium.hp    -3.2354904  0.4168465
## Erliansaurus.hp    -3.4141241 -0.6467804
## Falcarius.hp       -2.6297254 -0.9299619
## Guanlong.hp        -4.1030430  2.5541163
## Haplocheirus.hp    -2.6635726 -1.5746950
## Linhenykus.hp      -4.1621863  2.5353954
## Manis.hp           -4.2384906  3.1852543
## Mononykus.hp       -4.0310255  2.4064425
## Nothronychus.hp     1.1542818 -2.6160904
## Puma.hp            -4.5050087  5.7497375
## Shishugounykus.hp  -4.5582694  2.9242360
## Tamandua.hp         2.1280086 -6.3318209
## Therizinosaurus.hp 20.7790085  3.4247211
## Tugulusaurus.hp    -4.8150244  6.4260867
```

```
Coord
```

```
##                            PC1        PC2
## Allosaurus.p       -3.34322735  1.5024415
## Alxasaurus.p       -4.25547189  3.8567721
## Aorun.p            -2.53687163  0.1436204
## Bannykus.p          0.95231302 -2.6743476
## Beipiaosaurus.p     3.81786982 -4.0764147
## Eremotherium.p     -0.86870155 -1.4597797
## Erliansaurus.p     -1.31118643 -2.7903685
## Falcarius.p        -0.05572564 -1.9047215
## Guanlong.p         -2.45530339  0.2594165
## Haplocheirus.p     -1.30671697 -1.4332262
## Linhenykus.p       -4.41812298  4.2021227
## Manis.p            -2.90212515 -0.3406726
## Mononykus.p        -3.56290532  1.4871996
## Nothronychus.p      1.75663273 -3.5751840
## Puma.p             -3.33456905  1.8648867
## Shishugounykus.p   -3.94190947  3.1544965
## Tamandua.p          3.45214163 -6.2439935
## Therizinosaurus.p  21.89583870  6.1195515
## Tugulusaurus.p     -3.13483590  2.0373422
## Allosaurus.sd       2.40864451 -2.2084409
## Alxasaurus.sd      -0.95532526 -3.3378570
## Aorun.sd            2.48152554 -2.3874001
## Bannykus.sd         4.78836331 -3.8180150
## Beipiaosaurus.sd    4.87982360 -3.9494467
## Eremotherium.sd    -0.30507397 -1.1973785
## Erliansaurus.sd     0.13827691 -2.3141258
## Falcarius.sd        4.22618045 -3.9608883
## Guanlong.sd        -0.37376791  0.4249703
## Haplocheirus.sd     2.11634198 -1.4224292
## Linhenykus.sd      -4.41812506  4.2020874
## Manis.sd           -1.85871510 -2.9528135
## Mononykus.sd       -3.56290888  1.4872153
## Nothronychus.sd     0.31039204  0.9309279
## Puma.sd            -2.13270643  2.1343638
## Shishugounykus.sd  -1.15998242  0.7198907
## Tamandua.sd         9.74696208 -4.0015429
## Therizinosaurus.sd 21.26729943  7.3602389
## Tugulusaurus.sd    -2.15120564  1.8696094
## Allosaurus.hp      -2.04750450 -1.4825623
## Alxasaurus.hp      -3.79771123  2.9332823
## Aorun.hp           -2.74668706 -1.5153310
## Bannykus.hp        -2.08910621 -1.9456923
## Beipiaosaurus.hp   -0.91745173 -3.2212909
## Eremotherium.hp    -3.23549043  0.4168465
## Erliansaurus.hp    -3.41412410 -0.6467804
## Falcarius.hp       -2.62972535 -0.9299619
## Guanlong.hp        -4.10304303  2.5541163
## Haplocheirus.hp    -2.66357265 -1.5746950
## Linhenykus.hp      -4.16218631  2.5353954
## Manis.hp           -4.23849061  3.1852543
## Mononykus.hp       -4.03102550  2.4064425
## Nothronychus.hp     1.15428179 -2.6160904
## Puma.hp            -4.50500872  5.7497375
## Shishugounykus.hp  -4.55826939  2.9242360
## Tamandua.hp         2.12800856 -6.3318209
## Therizinosaurus.hp 20.77900846  3.4247211
## Tugulusaurus.hp    -4.81502435  6.4260867
```

```
options(scipen = 10000)
Coord.centroid<-Coord[1:19, 1:2]
Coord.centroid
```

```
##                           PC1        PC2
## Allosaurus.p      -3.34322735  1.5024415
## Alxasaurus.p      -4.25547189  3.8567721
## Aorun.p           -2.53687163  0.1436204
## Bannykus.p         0.95231302 -2.6743476
## Beipiaosaurus.p    3.81786982 -4.0764147
## Eremotherium.p    -0.86870155 -1.4597797
## Erliansaurus.p    -1.31118643 -2.7903685
## Falcarius.p       -0.05572564 -1.9047215
## Guanlong.p        -2.45530339  0.2594165
## Haplocheirus.p    -1.30671697 -1.4332262
## Linhenykus.p      -4.41812298  4.2021227
## Manis.p           -2.90212515 -0.3406726
## Mononykus.p       -3.56290532  1.4871996
## Nothronychus.p     1.75663273 -3.5751840
## Puma.p            -3.33456905  1.8648867
## Shishugounykus.p  -3.94190947  3.1544965
## Tamandua.p         3.45214163 -6.2439935
## Therizinosaurus.p 21.89583870  6.1195515
## Tugulusaurus.p    -3.13483590  2.0373422
```

```
row.names(Coord.centroid)<-Claw.group[1:19]
Coord.centroid
```

```
##                         PC1        PC2
## Allosaurus      -3.34322735  1.5024415
## Alxasaurus      -4.25547189  3.8567721
## Aorun           -2.53687163  0.1436204
## Bannykus         0.95231302 -2.6743476
## Beipiaosaurus    3.81786982 -4.0764147
## Eremotherium    -0.86870155 -1.4597797
## Erliansaurus    -1.31118643 -2.7903685
## Falcarius       -0.05572564 -1.9047215
## Guanlong        -2.45530339  0.2594165
## Haplocheirus    -1.30671697 -1.4332262
## Linhenykus      -4.41812298  4.2021227
## Manis           -2.90212515 -0.3406726
## Mononykus       -3.56290532  1.4871996
## Nothronychus     1.75663273 -3.5751840
## Puma            -3.33456905  1.8648867
## Shishugounykus  -3.94190947  3.1544965
## Tamandua         3.45214163 -6.2439935
## Therizinosaurus 21.89583870  6.1195515
## Tugulusaurus    -3.13483590  2.0373422
```

## 4.2 Coordinates of individuals

```
##Allosaurus###
N=1
xa<-Coord.p[N,1]
ya<-Coord.p[N,2]
xb<-Coord.s[N,1]
yb<-Coord.s[N,2]
xc<-Coord.hp[N,1]
yc<-Coord.hp[N,2]

x<-(xa+xb+xc)/3
y<-(ya+yb+yc)/3
Coord.centroid[1,1]=x
Coord.centroid[1,2]=y
Coord.centroid
```

```
##                         PC1        PC2
## Allosaurus      -0.99402911 -0.7295206
## Alxasaurus      -4.25547189  3.8567721
## Aorun           -2.53687163  0.1436204
## Bannykus         0.95231302 -2.6743476
## Beipiaosaurus    3.81786982 -4.0764147
## Eremotherium    -0.86870155 -1.4597797
## Erliansaurus    -1.31118643 -2.7903685
## Falcarius       -0.05572564 -1.9047215
## Guanlong        -2.45530339  0.2594165
## Haplocheirus    -1.30671697 -1.4332262
## Linhenykus      -4.41812298  4.2021227
## Manis           -2.90212515 -0.3406726
## Mononykus       -3.56290532  1.4871996
## Nothronychus     1.75663273 -3.5751840
## Puma            -3.33456905  1.8648867
## Shishugounykus  -3.94190947  3.1544965
## Tamandua         3.45214163 -6.2439935
## Therizinosaurus 21.89583870  6.1195515
## Tugulusaurus    -3.13483590  2.0373422
```

```
##Alxasaurus###
N=2
xa<-Coord.p[N,1]
ya<-Coord.p[N,2]
xb<-Coord.s[N,1]
yb<-Coord.s[N,2]
xc<-Coord.hp[N,1]
yc<-Coord.hp[N,2]

x<-(xa+xb+xc)/3
y<-(ya+yb+yc)/3
Coord.centroid[2,1]=x
Coord.centroid[2,2]=y
Coord.centroid
```

```
##                         PC1        PC2
## Allosaurus      -0.99402911 -0.7295206
## Alxasaurus      -3.00283613  1.1507325
## Aorun           -2.53687163  0.1436204
## Bannykus         0.95231302 -2.6743476
## Beipiaosaurus    3.81786982 -4.0764147
## Eremotherium    -0.86870155 -1.4597797
## Erliansaurus    -1.31118643 -2.7903685
## Falcarius       -0.05572564 -1.9047215
## Guanlong        -2.45530339  0.2594165
## Haplocheirus    -1.30671697 -1.4332262
## Linhenykus      -4.41812298  4.2021227
## Manis           -2.90212515 -0.3406726
## Mononykus       -3.56290532  1.4871996
## Nothronychus     1.75663273 -3.5751840
## Puma            -3.33456905  1.8648867
## Shishugounykus  -3.94190947  3.1544965
## Tamandua         3.45214163 -6.2439935
## Therizinosaurus 21.89583870  6.1195515
## Tugulusaurus    -3.13483590  2.0373422
```

```
##Aorun###
N=3
xa<-Coord.p[N,1]
ya<-Coord.p[N,2]
xb<-Coord.s[N,1]
yb<-Coord.s[N,2]
xc<-Coord.hp[N,1]
yc<-Coord.hp[N,2]

x<-(xa+xb+xc)/3
y<-(ya+yb+yc)/3
Coord.centroid[3,1]=x
Coord.centroid[3,2]=y
Coord.centroid
```

```
##                         PC1        PC2
## Allosaurus      -0.99402911 -0.7295206
## Alxasaurus      -3.00283613  1.1507325
## Aorun           -0.93401105 -1.2530369
## Bannykus         0.95231302 -2.6743476
## Beipiaosaurus    3.81786982 -4.0764147
## Eremotherium    -0.86870155 -1.4597797
## Erliansaurus    -1.31118643 -2.7903685
## Falcarius       -0.05572564 -1.9047215
## Guanlong        -2.45530339  0.2594165
## Haplocheirus    -1.30671697 -1.4332262
## Linhenykus      -4.41812298  4.2021227
## Manis           -2.90212515 -0.3406726
## Mononykus       -3.56290532  1.4871996
## Nothronychus     1.75663273 -3.5751840
## Puma            -3.33456905  1.8648867
## Shishugounykus  -3.94190947  3.1544965
## Tamandua         3.45214163 -6.2439935
## Therizinosaurus 21.89583870  6.1195515
## Tugulusaurus    -3.13483590  2.0373422
```

```
##Bannykus###
N=4
xa<-Coord.p[N,1]
ya<-Coord.p[N,2]
xb<-Coord.s[N,1]
yb<-Coord.s[N,2]
xc<-Coord.hp[N,1]
yc<-Coord.hp[N,2]

x<-(xa+xb+xc)/3
y<-(ya+yb+yc)/3
Coord.centroid[4,1]=x
Coord.centroid[4,2]=y
Coord.centroid
```

```
##                         PC1        PC2
## Allosaurus      -0.99402911 -0.7295206
## Alxasaurus      -3.00283613  1.1507325
## Aorun           -0.93401105 -1.2530369
## Bannykus         1.21719004 -2.8126850
## Beipiaosaurus    3.81786982 -4.0764147
## Eremotherium    -0.86870155 -1.4597797
## Erliansaurus    -1.31118643 -2.7903685
## Falcarius       -0.05572564 -1.9047215
## Guanlong        -2.45530339  0.2594165
## Haplocheirus    -1.30671697 -1.4332262
## Linhenykus      -4.41812298  4.2021227
## Manis           -2.90212515 -0.3406726
## Mononykus       -3.56290532  1.4871996
## Nothronychus     1.75663273 -3.5751840
## Puma            -3.33456905  1.8648867
## Shishugounykus  -3.94190947  3.1544965
## Tamandua         3.45214163 -6.2439935
## Therizinosaurus 21.89583870  6.1195515
## Tugulusaurus    -3.13483590  2.0373422
```

```
##Beipiaosaurus###
N=5
xa<-Coord.p[N,1]
ya<-Coord.p[N,2]
xb<-Coord.s[N,1]
yb<-Coord.s[N,2]
xc<-Coord.hp[N,1]
yc<-Coord.hp[N,2]

x<-(xa+xb+xc)/3
y<-(ya+yb+yc)/3
Coord.centroid[5,1]=x
Coord.centroid[5,2]=y
Coord.centroid
```

```
##                         PC1        PC2
## Allosaurus      -0.99402911 -0.7295206
## Alxasaurus      -3.00283613  1.1507325
## Aorun           -0.93401105 -1.2530369
## Bannykus         1.21719004 -2.8126850
## Beipiaosaurus    2.59341390 -3.7490508
## Eremotherium    -0.86870155 -1.4597797
## Erliansaurus    -1.31118643 -2.7903685
## Falcarius       -0.05572564 -1.9047215
## Guanlong        -2.45530339  0.2594165
## Haplocheirus    -1.30671697 -1.4332262
## Linhenykus      -4.41812298  4.2021227
## Manis           -2.90212515 -0.3406726
## Mononykus       -3.56290532  1.4871996
## Nothronychus     1.75663273 -3.5751840
## Puma            -3.33456905  1.8648867
## Shishugounykus  -3.94190947  3.1544965
## Tamandua         3.45214163 -6.2439935
## Therizinosaurus 21.89583870  6.1195515
## Tugulusaurus    -3.13483590  2.0373422
```

```
##Eremontherium###
N=6
xa<-Coord.p[N,1]
ya<-Coord.p[N,2]
xb<-Coord.s[N,1]
yb<-Coord.s[N,2]
xc<-Coord.hp[N,1]
yc<-Coord.hp[N,2]

x<-(xa+xb+xc)/3
y<-(ya+yb+yc)/3
Coord.centroid[6,1]=x
Coord.centroid[6,2]=y
Coord.centroid
```

```
##                         PC1        PC2
## Allosaurus      -0.99402911 -0.7295206
## Alxasaurus      -3.00283613  1.1507325
## Aorun           -0.93401105 -1.2530369
## Bannykus         1.21719004 -2.8126850
## Beipiaosaurus    2.59341390 -3.7490508
## Eremotherium    -1.46975532 -0.7467706
## Erliansaurus    -1.31118643 -2.7903685
## Falcarius       -0.05572564 -1.9047215
## Guanlong        -2.45530339  0.2594165
## Haplocheirus    -1.30671697 -1.4332262
## Linhenykus      -4.41812298  4.2021227
## Manis           -2.90212515 -0.3406726
## Mononykus       -3.56290532  1.4871996
## Nothronychus     1.75663273 -3.5751840
## Puma            -3.33456905  1.8648867
## Shishugounykus  -3.94190947  3.1544965
## Tamandua         3.45214163 -6.2439935
## Therizinosaurus 21.89583870  6.1195515
## Tugulusaurus    -3.13483590  2.0373422
```

```
##Erliansaurus###
N=7
xa<-Coord.p[N,1]
ya<-Coord.p[N,2]
xb<-Coord.s[N,1]
yb<-Coord.s[N,2]
xc<-Coord.hp[N,1]
yc<-Coord.hp[N,2]

x<-(xa+xb+xc)/3
y<-(ya+yb+yc)/3
Coord.centroid[7,1]=x
Coord.centroid[7,2]=y
Coord.centroid
```

```
##                         PC1        PC2
## Allosaurus      -0.99402911 -0.7295206
## Alxasaurus      -3.00283613  1.1507325
## Aorun           -0.93401105 -1.2530369
## Bannykus         1.21719004 -2.8126850
## Beipiaosaurus    2.59341390 -3.7490508
## Eremotherium    -1.46975532 -0.7467706
## Erliansaurus    -1.52901121 -1.9170916
## Falcarius       -0.05572564 -1.9047215
## Guanlong        -2.45530339  0.2594165
## Haplocheirus    -1.30671697 -1.4332262
## Linhenykus      -4.41812298  4.2021227
## Manis           -2.90212515 -0.3406726
## Mononykus       -3.56290532  1.4871996
## Nothronychus     1.75663273 -3.5751840
## Puma            -3.33456905  1.8648867
## Shishugounykus  -3.94190947  3.1544965
## Tamandua         3.45214163 -6.2439935
## Therizinosaurus 21.89583870  6.1195515
## Tugulusaurus    -3.13483590  2.0373422
```

```
##Falcarius###
N=8
xa<-Coord.p[N,1]
ya<-Coord.p[N,2]
xb<-Coord.s[N,1]
yb<-Coord.s[N,2]
xc<-Coord.hp[N,1]
yc<-Coord.hp[N,2]

x<-(xa+xb+xc)/3
y<-(ya+yb+yc)/3
Coord.centroid[8,1]=x
Coord.centroid[8,2]=y
Coord.centroid
```

```
##                        PC1        PC2
## Allosaurus      -0.9940291 -0.7295206
## Alxasaurus      -3.0028361  1.1507325
## Aorun           -0.9340111 -1.2530369
## Bannykus         1.2171900 -2.8126850
## Beipiaosaurus    2.5934139 -3.7490508
## Eremotherium    -1.4697553 -0.7467706
## Erliansaurus    -1.5290112 -1.9170916
## Falcarius        0.5135765 -2.2651906
## Guanlong        -2.4553034  0.2594165
## Haplocheirus    -1.3067170 -1.4332262
## Linhenykus      -4.4181230  4.2021227
## Manis           -2.9021252 -0.3406726
## Mononykus       -3.5629053  1.4871996
## Nothronychus     1.7566327 -3.5751840
## Puma            -3.3345691  1.8648867
## Shishugounykus  -3.9419095  3.1544965
## Tamandua         3.4521416 -6.2439935
## Therizinosaurus 21.8958387  6.1195515
## Tugulusaurus    -3.1348359  2.0373422
```

```
##Guanlong###

N=9
xa<-Coord.p[N,1]
ya<-Coord.p[N,2]
xb<-Coord.s[N,1]
yb<-Coord.s[N,2]
xc<-Coord.hp[N,1]
yc<-Coord.hp[N,2]

x<-(xa+xb+xc)/3
y<-(ya+yb+yc)/3
Coord.centroid[9,1]=x
Coord.centroid[9,2]=y
Coord.centroid
```

```
##                        PC1        PC2
## Allosaurus      -0.9940291 -0.7295206
## Alxasaurus      -3.0028361  1.1507325
## Aorun           -0.9340111 -1.2530369
## Bannykus         1.2171900 -2.8126850
## Beipiaosaurus    2.5934139 -3.7490508
## Eremotherium    -1.4697553 -0.7467706
## Erliansaurus    -1.5290112 -1.9170916
## Falcarius        0.5135765 -2.2651906
## Guanlong        -2.3107048  1.0795010
## Haplocheirus    -1.3067170 -1.4332262
## Linhenykus      -4.4181230  4.2021227
## Manis           -2.9021252 -0.3406726
## Mononykus       -3.5629053  1.4871996
## Nothronychus     1.7566327 -3.5751840
## Puma            -3.3345691  1.8648867
## Shishugounykus  -3.9419095  3.1544965
## Tamandua         3.4521416 -6.2439935
## Therizinosaurus 21.8958387  6.1195515
## Tugulusaurus    -3.1348359  2.0373422
```

```
##Haplocheirus###
N=10
xa<-Coord.p[N,1]
ya<-Coord.p[N,2]
xb<-Coord.s[N,1]
yb<-Coord.s[N,2]
xc<-Coord.hp[N,1]
yc<-Coord.hp[N,2]

x<-(xa+xb+xc)/3
y<-(ya+yb+yc)/3
Coord.centroid[10,1]=x
Coord.centroid[10,2]=y
Coord.centroid
```

```
##                        PC1        PC2
## Allosaurus      -0.9940291 -0.7295206
## Alxasaurus      -3.0028361  1.1507325
## Aorun           -0.9340111 -1.2530369
## Bannykus         1.2171900 -2.8126850
## Beipiaosaurus    2.5934139 -3.7490508
## Eremotherium    -1.4697553 -0.7467706
## Erliansaurus    -1.5290112 -1.9170916
## Falcarius        0.5135765 -2.2651906
## Guanlong        -2.3107048  1.0795010
## Haplocheirus    -0.6179825 -1.4767835
## Linhenykus      -4.4181230  4.2021227
## Manis           -2.9021252 -0.3406726
## Mononykus       -3.5629053  1.4871996
## Nothronychus     1.7566327 -3.5751840
## Puma            -3.3345691  1.8648867
## Shishugounykus  -3.9419095  3.1544965
## Tamandua         3.4521416 -6.2439935
## Therizinosaurus 21.8958387  6.1195515
## Tugulusaurus    -3.1348359  2.0373422
```

```
##Linhenykus###
N=11
xa<-Coord.p[N,1]
ya<-Coord.p[N,2]
xb<-Coord.s[N,1]
yb<-Coord.s[N,2]
xc<-Coord.hp[N,1]
yc<-Coord.hp[N,2]

x<-(xa+xb+xc)/3
y<-(ya+yb+yc)/3
Coord.centroid[11,1]=x
Coord.centroid[11,2]=y
Coord.centroid
```

```
##                        PC1        PC2
## Allosaurus      -0.9940291 -0.7295206
## Alxasaurus      -3.0028361  1.1507325
## Aorun           -0.9340111 -1.2530369
## Bannykus         1.2171900 -2.8126850
## Beipiaosaurus    2.5934139 -3.7490508
## Eremotherium    -1.4697553 -0.7467706
## Erliansaurus    -1.5290112 -1.9170916
## Falcarius        0.5135765 -2.2651906
## Guanlong        -2.3107048  1.0795010
## Haplocheirus    -0.6179825 -1.4767835
## Linhenykus      -4.3328114  3.6465351
## Manis           -2.9021252 -0.3406726
## Mononykus       -3.5629053  1.4871996
## Nothronychus     1.7566327 -3.5751840
## Puma            -3.3345691  1.8648867
## Shishugounykus  -3.9419095  3.1544965
## Tamandua         3.4521416 -6.2439935
## Therizinosaurus 21.8958387  6.1195515
## Tugulusaurus    -3.1348359  2.0373422
```

```
##Manis###

N=12
xa<-Coord.p[N,1]
ya<-Coord.p[N,2]
xb<-Coord.s[N,1]
yb<-Coord.s[N,2]
xc<-Coord.hp[N,1]
yc<-Coord.hp[N,2]

x<-(xa+xb+xc)/3
y<-(ya+yb+yc)/3
Coord.centroid[12,1]=x
Coord.centroid[12,2]=y
Coord.centroid
```

```
##                        PC1        PC2
## Allosaurus      -0.9940291 -0.7295206
## Alxasaurus      -3.0028361  1.1507325
## Aorun           -0.9340111 -1.2530369
## Bannykus         1.2171900 -2.8126850
## Beipiaosaurus    2.5934139 -3.7490508
## Eremotherium    -1.4697553 -0.7467706
## Erliansaurus    -1.5290112 -1.9170916
## Falcarius        0.5135765 -2.2651906
## Guanlong        -2.3107048  1.0795010
## Haplocheirus    -0.6179825 -1.4767835
## Linhenykus      -4.3328114  3.6465351
## Manis           -2.9997770 -0.0360773
## Mononykus       -3.5629053  1.4871996
## Nothronychus     1.7566327 -3.5751840
## Puma            -3.3345691  1.8648867
## Shishugounykus  -3.9419095  3.1544965
## Tamandua         3.4521416 -6.2439935
## Therizinosaurus 21.8958387  6.1195515
## Tugulusaurus    -3.1348359  2.0373422
```

```
##Mononykus###

N=13
xa<-Coord.p[N,1]
ya<-Coord.p[N,2]
xb<-Coord.s[N,1]
yb<-Coord.s[N,2]
xc<-Coord.hp[N,1]
yc<-Coord.hp[N,2]

x<-(xa+xb+xc)/3
y<-(ya+yb+yc)/3
Coord.centroid[13,1]=x
Coord.centroid[13,2]=y
Coord.centroid
```

```
##                        PC1        PC2
## Allosaurus      -0.9940291 -0.7295206
## Alxasaurus      -3.0028361  1.1507325
## Aorun           -0.9340111 -1.2530369
## Bannykus         1.2171900 -2.8126850
## Beipiaosaurus    2.5934139 -3.7490508
## Eremotherium    -1.4697553 -0.7467706
## Erliansaurus    -1.5290112 -1.9170916
## Falcarius        0.5135765 -2.2651906
## Guanlong        -2.3107048  1.0795010
## Haplocheirus    -0.6179825 -1.4767835
## Linhenykus      -4.3328114  3.6465351
## Manis           -2.9997770 -0.0360773
## Mononykus       -3.7189466  1.7936191
## Nothronychus     1.7566327 -3.5751840
## Puma            -3.3345691  1.8648867
## Shishugounykus  -3.9419095  3.1544965
## Tamandua         3.4521416 -6.2439935
## Therizinosaurus 21.8958387  6.1195515
## Tugulusaurus    -3.1348359  2.0373422
```

```
##Nothronychus###

N=14
xa<-Coord.p[N,1]
ya<-Coord.p[N,2]
xb<-Coord.s[N,1]
yb<-Coord.s[N,2]
xc<-Coord.hp[N,1]
yc<-Coord.hp[N,2]

x<-(xa+xb+xc)/3
y<-(ya+yb+yc)/3
Coord.centroid[14,1]=x
Coord.centroid[14,2]=y
Coord.centroid
```

```
##                        PC1        PC2
## Allosaurus      -0.9940291 -0.7295206
## Alxasaurus      -3.0028361  1.1507325
## Aorun           -0.9340111 -1.2530369
## Bannykus         1.2171900 -2.8126850
## Beipiaosaurus    2.5934139 -3.7490508
## Eremotherium    -1.4697553 -0.7467706
## Erliansaurus    -1.5290112 -1.9170916
## Falcarius        0.5135765 -2.2651906
## Guanlong        -2.3107048  1.0795010
## Haplocheirus    -0.6179825 -1.4767835
## Linhenykus      -4.3328114  3.6465351
## Manis           -2.9997770 -0.0360773
## Mononykus       -3.7189466  1.7936191
## Nothronychus     1.0737689 -1.7534488
## Puma            -3.3345691  1.8648867
## Shishugounykus  -3.9419095  3.1544965
## Tamandua         3.4521416 -6.2439935
## Therizinosaurus 21.8958387  6.1195515
## Tugulusaurus    -3.1348359  2.0373422
```

```
#Puma###


N=15
xa<-Coord.p[N,1]
ya<-Coord.p[N,2]
xb<-Coord.s[N,1]
yb<-Coord.s[N,2]
xc<-Coord.hp[N,1]
yc<-Coord.hp[N,2]

x<-(xa+xb+xc)/3
y<-(ya+yb+yc)/3
Coord.centroid[15,1]=x
Coord.centroid[15,2]=y
Coord.centroid
```

```
##                        PC1        PC2
## Allosaurus      -0.9940291 -0.7295206
## Alxasaurus      -3.0028361  1.1507325
## Aorun           -0.9340111 -1.2530369
## Bannykus         1.2171900 -2.8126850
## Beipiaosaurus    2.5934139 -3.7490508
## Eremotherium    -1.4697553 -0.7467706
## Erliansaurus    -1.5290112 -1.9170916
## Falcarius        0.5135765 -2.2651906
## Guanlong        -2.3107048  1.0795010
## Haplocheirus    -0.6179825 -1.4767835
## Linhenykus      -4.3328114  3.6465351
## Manis           -2.9997770 -0.0360773
## Mononykus       -3.7189466  1.7936191
## Nothronychus     1.0737689 -1.7534488
## Puma            -3.3240947  3.2496627
## Shishugounykus  -3.9419095  3.1544965
## Tamandua         3.4521416 -6.2439935
## Therizinosaurus 21.8958387  6.1195515
## Tugulusaurus    -3.1348359  2.0373422
```

```
###Shishugounykus###

N=16
xa<-Coord.p[N,1]
ya<-Coord.p[N,2]
xb<-Coord.s[N,1]
yb<-Coord.s[N,2]
xc<-Coord.hp[N,1]
yc<-Coord.hp[N,2]

x<-(xa+xb+xc)/3
y<-(ya+yb+yc)/3
Coord.centroid[16,1]=x
Coord.centroid[16,2]=y
Coord.centroid
```

```
##                        PC1        PC2
## Allosaurus      -0.9940291 -0.7295206
## Alxasaurus      -3.0028361  1.1507325
## Aorun           -0.9340111 -1.2530369
## Bannykus         1.2171900 -2.8126850
## Beipiaosaurus    2.5934139 -3.7490508
## Eremotherium    -1.4697553 -0.7467706
## Erliansaurus    -1.5290112 -1.9170916
## Falcarius        0.5135765 -2.2651906
## Guanlong        -2.3107048  1.0795010
## Haplocheirus    -0.6179825 -1.4767835
## Linhenykus      -4.3328114  3.6465351
## Manis           -2.9997770 -0.0360773
## Mononykus       -3.7189466  1.7936191
## Nothronychus     1.0737689 -1.7534488
## Puma            -3.3240947  3.2496627
## Shishugounykus  -3.2200538  2.2662077
## Tamandua         3.4521416 -6.2439935
## Therizinosaurus 21.8958387  6.1195515
## Tugulusaurus    -3.1348359  2.0373422
```

```
#Tamandua###

N=17
xa<-Coord.p[N,1]
ya<-Coord.p[N,2]
xb<-Coord.s[N,1]
yb<-Coord.s[N,2]
xc<-Coord.hp[N,1]
yc<-Coord.hp[N,2]

x<-(xa+xb+xc)/3
y<-(ya+yb+yc)/3
Coord.centroid[17,1]=x
Coord.centroid[17,2]=y
Coord.centroid
```

```
##                        PC1        PC2
## Allosaurus      -0.9940291 -0.7295206
## Alxasaurus      -3.0028361  1.1507325
## Aorun           -0.9340111 -1.2530369
## Bannykus         1.2171900 -2.8126850
## Beipiaosaurus    2.5934139 -3.7490508
## Eremotherium    -1.4697553 -0.7467706
## Erliansaurus    -1.5290112 -1.9170916
## Falcarius        0.5135765 -2.2651906
## Guanlong        -2.3107048  1.0795010
## Haplocheirus    -0.6179825 -1.4767835
## Linhenykus      -4.3328114  3.6465351
## Manis           -2.9997770 -0.0360773
## Mononykus       -3.7189466  1.7936191
## Nothronychus     1.0737689 -1.7534488
## Puma            -3.3240947  3.2496627
## Shishugounykus  -3.2200538  2.2662077
## Tamandua         5.1090374 -5.5257858
## Therizinosaurus 21.8958387  6.1195515
## Tugulusaurus    -3.1348359  2.0373422
```

```
##Therizinosaurus###
N=18
xa<-Coord.p[N,1]
ya<-Coord.p[N,2]
xb<-Coord.s[N,1]
yb<-Coord.s[N,2]
xc<-Coord.hp[N,1]
yc<-Coord.hp[N,2]

x<-(xa+xb+xc)/3
y<-(ya+yb+yc)/3
Coord.centroid[18,1]=x
Coord.centroid[18,2]=y
Coord.centroid
```

```
##                        PC1        PC2
## Allosaurus      -0.9940291 -0.7295206
## Alxasaurus      -3.0028361  1.1507325
## Aorun           -0.9340111 -1.2530369
## Bannykus         1.2171900 -2.8126850
## Beipiaosaurus    2.5934139 -3.7490508
## Eremotherium    -1.4697553 -0.7467706
## Erliansaurus    -1.5290112 -1.9170916
## Falcarius        0.5135765 -2.2651906
## Guanlong        -2.3107048  1.0795010
## Haplocheirus    -0.6179825 -1.4767835
## Linhenykus      -4.3328114  3.6465351
## Manis           -2.9997770 -0.0360773
## Mononykus       -3.7189466  1.7936191
## Nothronychus     1.0737689 -1.7534488
## Puma            -3.3240947  3.2496627
## Shishugounykus  -3.2200538  2.2662077
## Tamandua         5.1090374 -5.5257858
## Therizinosaurus 21.3140489  5.6348372
## Tugulusaurus    -3.1348359  2.0373422
```

```
##Tugulusaurus###
N=19
xa<-Coord.p[N,1]
ya<-Coord.p[N,2]
xb<-Coord.s[N,1]
yb<-Coord.s[N,2]
xc<-Coord.hp[N,1]
yc<-Coord.hp[N,2]

x<-(xa+xb+xc)/3
y<-(ya+yb+yc)/3
Coord.centroid[19,1]=x
Coord.centroid[19,2]=y
Coord.centroid
```

```
##                        PC1        PC2
## Allosaurus      -0.9940291 -0.7295206
## Alxasaurus      -3.0028361  1.1507325
## Aorun           -0.9340111 -1.2530369
## Bannykus         1.2171900 -2.8126850
## Beipiaosaurus    2.5934139 -3.7490508
## Eremotherium    -1.4697553 -0.7467706
## Erliansaurus    -1.5290112 -1.9170916
## Falcarius        0.5135765 -2.2651906
## Guanlong        -2.3107048  1.0795010
## Haplocheirus    -0.6179825 -1.4767835
## Linhenykus      -4.3328114  3.6465351
## Manis           -2.9997770 -0.0360773
## Mononykus       -3.7189466  1.7936191
## Nothronychus     1.0737689 -1.7534488
## Puma            -3.3240947  3.2496627
## Shishugounykus  -3.2200538  2.2662077
## Tamandua         5.1090374 -5.5257858
## Therizinosaurus 21.3140489  5.6348372
## Tugulusaurus    -3.3670220  3.4443461
```

```
###save 

write.csv(Coord.centroid,'Coord.centroid.csv')
```

## 4.3 Plots of Coordinates

```
MWAM<-read.csv("MWAN.csv",header=T,row.names =1)
MWAM
```

```
##                  Piercing Scratch.digging Hook.and.pull.digging
## Allosaurus      11.686928       23.128360             14.653577
## Alxasaurus       8.543933       16.579043             10.288983
## Aorun           13.042667       23.893906             13.574143
## Bannykus        12.948863       23.598193             13.555904
## Beipiaosaurus   23.685686       27.006217             16.715786
## Eremotherium    17.174796       18.761361             13.132937
## Erliansaurus    15.580538       17.286285             12.199441
## Falcarius       17.394739       29.062512             13.872308
## Guanlong        12.756350       15.978561              9.792356
## Haplocheirous   15.075024       21.342703             13.216308
## Linhenykus       8.483857        8.483858              9.986201
## Manis                  NA              NA                    NA
## Mononykus       11.792620       16.631616             10.755348
## Nothronychus    20.947966       24.459247             19.025633
## Puma                   NA              NA                    NA
## Shishugounykus   9.035069       14.644445              8.888612
## Tamandua        22.426868       31.931135             20.644218
## Therizinosaurus 68.471554       82.217589             49.836773
## Tugulusaurus    10.160913       11.501567              4.660503
##                             Clade      Type    mya
## Allosaurus       NonManirtaptoran  arboreal -155.0
## Alxasaurus        Therizinosauria  arboreal -113.0
## Aorun           Alvarezsaurioidea fossorial -166.1
## Bannykus        Alvarezsaurioidea  arboreal -125.0
## Beipiaosaurus     Therizinosauria  arboreal -125.0
## Eremotherium              Mammals fossorial   -4.9
## Erliansaurus      Therizinosauria  arboreal  -85.0
## Falcarius         Therizinosauria  arboreal -129.0
## Guanlong         NonManirtaptoran  arboreal -160.0
## Haplocheirous   Alvarezsaurioidea fossorial -166.1
## Linhenykus      Alvarezsaurioidea fossorial  -83.6
## Manis                     Mammals fossorial    0.0
## Mononykus       Alvarezsaurioidea fossorial  -72.1
## Nothronychus      Therizinosauria  arboreal  -92.0
## Puma                      Mammals  arboreal    0.0
## Shishugounykus  Alvarezsaurioidea fossorial -166.1
## Tamandua                  Mammals fossorial    0.0
## Therizinosaurus   Therizinosauria  arboreal  -72.1
## Tugulusaurus    Alvarezsaurioidea fossorial -139.8
```

```
Mya<-MWAM[,6]
Clade<-MWAM[,4]
hull_area_list<-read.csv("hull_area_list.csv",header=T,row.names =1)
Hullarea<-hull_area_list[,2]
df.Coord.centroid<- data.frame(Coord.centroid,Mya,Clade,Hullarea)
df.Coord.centroid
```

```
##                        PC1        PC2    Mya             Clade   Hullarea
## Allosaurus      -0.9940291 -0.7295206 -155.0  NonManirtaptoran 6.18054203
## Alxasaurus      -3.0028361  1.1507325 -113.0   Therizinosauria 0.12288320
## Aorun           -0.9340111 -1.2530369 -166.1 Alvarezsaurioidea 4.42816221
## Bannykus         1.2171900 -2.8126850 -125.0 Alvarezsaurioidea 0.34160699
## Beipiaosaurus    2.5934139 -3.7490508 -125.0   Therizinosauria 0.75466812
## Eremotherium    -1.4697553 -0.7467706   -4.9           Mammals 0.83938332
## Erliansaurus    -1.5290112 -1.9170916  -85.0   Therizinosauria 2.05428058
## Falcarius        0.5135765 -2.2651906 -129.0   Therizinosauria 0.55937158
## Guanlong        -2.3107048  1.0795010 -160.0  NonManirtaptoran 2.52464428
## Haplocheirus    -0.6179825 -1.4767835 -166.1 Alvarezsaurioidea 0.23480308
## Linhenykus      -4.3328114  3.6465351  -83.6 Alvarezsaurioidea 0.00000625
## Manis           -2.9997770 -0.0360773    0.0           Mammals 0.09410633
## Mononykus       -3.7189466  1.7936191  -72.1 Alvarezsaurioidea 0.00000204
## Nothronychus     1.0737689 -1.7534488  -92.0   Therizinosauria 0.66359031
## Puma            -3.3240947  3.2496627    0.0           Mammals 2.49223187
## Shishugounykus  -3.2200538  2.2662077 -166.1 Alvarezsaurioidea 1.07058064
## Tamandua         5.1090374 -5.5257858    0.0           Mammals 1.20822268
## Therizinosaurus 21.3140489  5.6348372  -72.1   Therizinosauria 1.53972195
## Tugulusaurus    -3.3670220  3.4443461 -139.8 Alvarezsaurioidea 2.01753963
```

```
##Plot of centroid and hull areas
g<-ggplot(data=df.Coord.centroid, aes(x=PC1, y=PC2, size=Hullarea,colour=Clade))+geom_point()
g<-g+geom_text_repel(aes(label = rownames(df.Coord.centroid)), size = 3)
g
ggsave(file='the centroids of each functional triangle.svg', plot=g,units="mm", width=300, height=300)
ggsave(file='the centroids of each functional triangle.pdf', plot=g,units="mm", width=300, height=300)

##Plot of centroid and hull areas+ hist
g1<-ggplot(data=df.Coord.centroid, aes(x=PC1, y=PC2, size=Hullarea,colour=Clade))+geom_point(alpha=0.5)+scale_size(range = c(1, 24), name="Population (M)")
g1<-g1+geom_text_repel(aes(label = rownames(df.Coord.centroid)), size = 3)+theme(legend.position="none")
g1<-ggMarginal(g1, type="histogram", fill = "purple",alpha=0.5)
g1
```

```
ggsave(file='the centroids.svg', plot=g1,units="mm", width=300, height=300)
ggsave(file='the centroids.pdf', plot=g1,units="mm", width=300, height=300)
```

# 5.Caculating the coordinates of vertex

## 5.1 Coordinates of individual vertexes

```
ind.coord <- Claw.pca$x
ind.coord
```

```
##                            PC1        PC2          PC3         PC4
## Allosaurus.p       -3.34322735  1.5024415 -0.283485671 -0.34270920
## Alxasaurus.p       -4.25547189  3.8567721  0.968591611  0.08721399
## Aorun.p            -2.53687163  0.1436204 -0.570672273  0.73341391
## Bannykus.p          0.95231302 -2.6743476 -1.315046636  0.82448412
## Beipiaosaurus.p     3.81786982 -4.0764147  0.663037687 -0.51422791
## Eremotherium.p     -0.86870155 -1.4597797 -0.248024309 -1.75369054
## Erliansaurus.p     -1.31118643 -2.7903685 -0.344203520 -0.37516780
## Falcarius.p        -0.05572564 -1.9047215 -0.416316361 -0.23129536
## Guanlong.p         -2.45530339  0.2594165  0.018335690 -0.35313360
## Haplocheirus.p     -1.30671697 -1.4332262 -0.213730587  0.47816196
## Linhenykus.p       -4.41812298  4.2021227  0.265993563  1.60316911
## Manis.p            -2.90212515 -0.3406726 -1.499151122 -0.59876132
## Mononykus.p        -3.56290532  1.4871996 -1.755561286  1.16844639
## Nothronychus.p      1.75663273 -3.5751840 -0.299306302  0.24297926
## Puma.p             -3.33456905  1.8648867  0.569785594 -0.18090112
## Shishugounykus.p   -3.94190947  3.1544965  1.694840912 -0.05927715
## Tamandua.p          3.45214163 -6.2439935  3.616944678  1.07636588
## Therizinosaurus.p  21.89583870  6.1195515 -1.392877650 -0.28444559
## Tugulusaurus.p     -3.13483590  2.0373422  2.160661465 -0.24668631
## Allosaurus.sd       2.40864451 -2.2084409  0.040838848 -0.64252013
## Alxasaurus.sd      -0.95532526 -3.3378570 -0.158130021 -1.41236044
## Aorun.sd            2.48152554 -2.3874001 -0.009313851 -0.28450104
## Bannykus.sd         4.78836331 -3.8180150 -0.238886670 -0.97482489
## Beipiaosaurus.sd    4.87982360 -3.9494467  0.097597024 -0.86851566
## Eremotherium.sd    -0.30507397 -1.1973785 -1.177209968 -1.72176884
## Erliansaurus.sd     0.13827691 -2.3141258 -0.325772888  0.21834034
## Falcarius.sd        4.22618045 -3.9608883  0.266866241 -0.70222579
## Guanlong.sd        -0.37376791  0.4249703  0.029826995  0.04118170
## Haplocheirus.sd     2.11634198 -1.4224292 -0.702595873 -0.48661276
## Linhenykus.sd      -4.41812506  4.2020874  0.265921136  1.60319849
## Manis.sd           -1.85871510 -2.9528135 -1.125617339 -1.70383349
## Mononykus.sd       -3.56290888  1.4872153 -1.755588351  1.16844423
## Nothronychus.sd     0.31039204  0.9309279 -0.507208412  1.37820611
## Puma.sd            -2.13270643  2.1343638  1.170282819 -0.09216460
## Shishugounykus.sd  -1.15998242  0.7198907 -0.519918949  1.43754927
## Tamandua.sd         9.74696208 -4.0015429  3.733625456  2.04401399
## Therizinosaurus.sd 21.26729943  7.3602389 -2.738420516 -1.89605637
## Tugulusaurus.sd    -2.15120564  1.8696094  2.298429270  0.41183879
## Allosaurus.hp      -2.04750450 -1.4825623 -0.314651076 -0.39195941
## Alxasaurus.hp      -3.79771123  2.9332823  0.359079842  0.29295689
## Aorun.hp           -2.74668706 -1.5153310 -1.568007885  0.49990871
## Bannykus.hp        -2.08910621 -1.9456923 -1.703866342  0.31565575
## Beipiaosaurus.hp   -0.91745173 -3.2212909 -0.772218892 -0.32273401
## Eremotherium.hp    -3.23549043  0.4168465 -1.691245211 -1.09242193
## Erliansaurus.hp    -3.41412410 -0.6467804 -1.552548924 -0.36663212
## Falcarius.hp       -2.62972535 -0.9299619 -1.627619305 -0.53516110
## Guanlong.hp        -4.10304303  2.5541163 -0.525680668 -0.11686757
## Haplocheirus.hp    -2.66357265 -1.5746950 -0.525976516 -0.08639338
## Linhenykus.hp      -4.16218631  2.5353954 -1.360361427  0.98195190
## Manis.hp           -4.23849061  3.1852543 -0.170677713  1.09097847
## Mononykus.hp       -4.03102550  2.4064425 -1.849324781  1.76856272
## Nothronychus.hp     1.15428179 -2.6160904 -0.458269361  0.99387454
## Puma.hp            -4.50500872  5.7497375  3.737623318 -1.93428326
## Shishugounykus.hp  -4.55826939  2.9242360  0.133154579  0.36633316
## Tamandua.hp         2.12800856 -6.3318209  3.458629305  0.44916975
## Therizinosaurus.hp 20.77900846  3.4247211  1.803375138  2.03562987
## Tugulusaurus.hp    -4.81502435  6.4260867  4.364045481 -2.73989661
##                              PC5          PC6          PC7          PC8
## Allosaurus.p       -0.0803834667  0.048666140 -0.277900283  0.231839785
## Alxasaurus.p       -0.0007094108 -0.242251885 -0.121083159 -0.038016410
## Aorun.p            -0.1208205340 -0.017315659  0.107766425  0.280608743
## Bannykus.p          1.0238962142  0.023122944  0.201095925 -0.247819176
## Beipiaosaurus.p    -0.3393671392  0.202394490  0.051158895 -0.028703088
## Eremotherium.p     -0.0220844138  0.072215270 -0.696725768 -0.176682732
## Erliansaurus.p      0.2245772333  0.557721656 -0.062968096  0.252244092
## Falcarius.p        -0.8847587740  0.361634774 -0.138734364 -0.306011593
## Guanlong.p          0.7625990566 -0.661855950 -0.063855977  0.365891232
## Haplocheirus.p      0.6625423566  0.153966407  0.360014305  0.317276519
## Linhenykus.p       -0.9302204415  0.158293518  0.022043176 -0.762216386
## Manis.p             0.1977846251  0.927361339  0.279380797 -0.134959664
## Mononykus.p         0.0841033824  0.401892091  0.679228994  0.064613330
## Nothronychus.p      0.2481384147 -0.248385397  0.342740106 -0.493822518
## Puma.p             -0.0824510942 -0.683605493 -0.611024404 -0.167857492
## Shishugounykus.p   -0.9689452817  0.396706682 -0.498471551  0.693902624
## Tamandua.p         -0.7719227094 -0.450450258  0.207845175 -0.022760826
## Therizinosaurus.p  -0.1252645009 -0.006730212  0.002182203 -0.292593321
## Tugulusaurus.p     -0.5938853580  1.101313525  1.008229369  0.119627576
## Allosaurus.sd       0.1782898879 -0.059174895  0.017084358  0.125008016
## Alxasaurus.sd       0.2461749306  0.233004308  0.033077102  0.097277485
## Aorun.sd           -0.8504325778 -0.128456751 -0.116684049 -0.489252939
## Bannykus.sd        -1.1978091922 -0.307488970 -0.428504016 -0.837022819
## Beipiaosaurus.sd    0.1784973935  0.636133373 -0.019253346  0.308175320
## Eremotherium.sd     0.8086968064  0.362955395 -0.349093968  0.143764613
## Erliansaurus.sd     0.6470187491  0.681518406 -0.102675730 -0.107019473
## Falcarius.sd       -0.6268617955 -0.090938393  0.076648193 -0.196294542
## Guanlong.sd         0.5936843086 -0.456990902 -0.260687497 -0.032207684
## Haplocheirus.sd     0.4509015460  0.661717244 -0.185121524  0.013800037
## Linhenykus.sd      -0.9301801793  0.158228272  0.022054811 -0.762297434
## Manis.sd           -0.5426757292  1.313586864 -0.448316274 -0.304076426
## Mononykus.sd        0.0841451223  0.401850088  0.679267416  0.064591716
## Nothronychus.sd    -1.2066208556 -0.482597229 -0.825455574  0.218751740
## Puma.sd             0.0445390674 -0.861326931 -0.344818381  0.003140411
## Shishugounykus.sd   0.1793816372 -0.598133758 -0.273556374 -0.487788346
## Tamandua.sd         0.3477662892 -0.203629124  0.193114260  0.546024420
## Therizinosaurus.sd -1.6074589952 -0.802991439  1.202597630  0.536805852
## Tugulusaurus.sd    -0.3573124918  1.496698100  0.626419695  0.068499417
## Allosaurus.hp       0.0069825754 -0.627857606  0.115681109  0.049170289
## Alxasaurus.hp      -0.6042905163  0.064893641 -1.023265630  1.003609537
## Aorun.hp            0.6599319091 -0.485074377  0.662683898  0.276360406
## Bannykus.hp         0.1743484719 -0.340597984  0.123143654  0.135144775
## Beipiaosaurus.hp    0.0807309840 -0.670469851  0.560303892 -0.078866392
## Eremotherium.hp     1.0002076379 -0.155152211 -0.447527168  0.450362558
## Erliansaurus.hp     0.2711722696  0.035405197  0.331605912  0.018586682
## Falcarius.hp       -0.5984572731  0.027868243 -1.192762480 -0.232431085
## Guanlong.hp         0.7566261588 -0.911633614 -0.432805274  0.169044428
## Haplocheirus.hp     0.3246062666 -0.265203378  0.555877908  0.401990033
## Linhenykus.hp       0.6341281442 -0.333432994  0.432940841 -0.104239079
## Manis.hp           -0.8475848956  0.924570697  0.043271384  0.295503614
## Mononykus.hp        0.3283751208 -0.459357932  0.551241357 -0.185401914
## Nothronychus.hp     0.8958048157 -0.245749953  0.249410885 -0.404438762
## Puma.hp             0.5642585309  0.505567365  0.876896975 -0.297057641
## Shishugounykus.hp  -0.9343167207 -0.193226392 -0.978457649  0.225653562
## Tamandua.hp        -0.9684068158 -0.945366897  0.403756860  0.333880437
## Therizinosaurus.hp  2.2055375482  1.007307825 -1.226159351 -0.115840782
## Tugulusaurus.hp     1.3277737077 -0.981147418  0.107144378 -0.505470725
##                             PC9         PC10        PC11         PC12
## Allosaurus.p        0.320855540 -0.193564568 -0.04566667 -0.021932364
## Alxasaurus.p        0.077330038 -0.166548843  0.12023527 -0.002297907
## Aorun.p             0.117662539  0.394039651 -0.27092321  0.020151372
## Bannykus.p          0.251471272  0.361412874  0.12074083 -0.309025863
## Beipiaosaurus.p    -0.089317623 -0.257396019 -0.33595310  0.199567189
## Eremotherium.p      0.255397901 -0.272723477  0.06770060 -0.081176664
## Erliansaurus.p     -0.427411988  0.060176259  0.04571854 -0.181961090
## Falcarius.p         0.300842877 -0.183756686 -0.24048464  0.125395216
## Guanlong.p          0.131238487  0.156416436 -0.09361790  0.121696562
## Haplocheirus.p     -0.482788618  0.055935727 -0.04853404 -0.125246761
## Linhenykus.p       -0.673217748  0.159122712 -0.08651743  0.020241199
## Manis.p            -0.213878182  0.187945061 -0.13211877 -0.050257637
## Mononykus.p         0.190340785 -0.098228653 -0.05834908 -0.005736390
## Nothronychus.p      0.185682540 -0.490212590  0.16498162 -0.219814561
## Puma.p              0.120206007 -0.139535239  0.07911995  0.059834351
## Shishugounykus.p    0.318895479  0.092167986 -0.25114720 -0.214006825
## Tamandua.p          0.221509439  0.482900859  0.34314155 -0.070690686
## Therizinosaurus.p   0.513885771  0.689999929  0.09475903  0.459384579
## Tugulusaurus.p      0.198857639 -0.266498759  0.03256698  0.184016139
## Allosaurus.sd       0.191606666  0.086241963 -0.10323004 -0.074898771
## Alxasaurus.sd      -0.206491774  0.008692859  0.15304303 -0.030468337
## Aorun.sd            0.368112847  0.230961672 -0.21983096 -0.134621705
## Bannykus.sd         0.172213877 -0.336466982 -0.22020380  0.048986562
## Beipiaosaurus.sd   -0.393984077  0.018400452 -0.21286152 -0.200255813
## Eremotherium.sd    -0.302500345 -0.059963497  0.45758681  0.138439720
## Erliansaurus.sd     0.169661487  0.599398247  0.16728552 -0.143730942
## Falcarius.sd       -0.051499070  0.094369751  0.06226042 -0.082277175
## Guanlong.sd         0.123636468  0.110021869  0.05401313 -0.020309484
## Haplocheirus.sd    -0.161011259  0.271596831 -0.10609852  0.279903036
## Linhenykus.sd      -0.673266210  0.159132884 -0.08649266  0.020303215
## Manis.sd           -0.166588783 -0.199143375  0.17332437  0.064388097
## Mononykus.sd        0.190299390 -0.098155787 -0.05838756 -0.005734138
## Nothronychus.sd    -0.441177088  0.059743812  0.20398902 -0.132506633
## Puma.sd             0.111844626 -0.149022140  0.14338674  0.110904051
## Shishugounykus.sd  -0.022961022 -0.101872199  0.31088195  0.043611280
## Tamandua.sd        -0.504266423 -0.180924776  0.04368938  0.399488545
## Therizinosaurus.sd -0.389894739 -0.235895816  0.15488401 -0.331552648
## Tugulusaurus.sd     0.338854124  0.060950783  0.27661988 -0.163707597
## Allosaurus.hp      -0.063969611 -0.161345692 -0.02319633  0.199389753
## Alxasaurus.hp       0.376366647 -0.029708803 -0.04496276 -0.077755744
## Aorun.hp           -0.071381040 -0.103744107 -0.06132330  0.118024470
## Bannykus.hp        -0.131207162 -0.088690861 -0.09806358  0.096889428
## Beipiaosaurus.hp    0.448665892  0.026951549 -0.34317386  0.009206499
## Eremotherium.hp    -0.152060029  0.140250203  0.19019376  0.048965230
## Erliansaurus.hp     0.056215511  0.089785382 -0.22818143 -0.007439098
## Falcarius.hp       -0.199617796  0.047510620  0.12164776  0.139714374
## Guanlong.hp         0.194002100 -0.132360785  0.17165541  0.039298977
## Haplocheirus.hp    -0.350386294  0.025815347 -0.20338540  0.264858902
## Linhenykus.hp       0.045399444 -0.020148317 -0.06161175 -0.035990127
## Manis.hp            0.006595240 -0.047563325  0.10287631  0.028950011
## Mononykus.hp        0.221365652 -0.249181549  0.02192124 -0.064381093
## Nothronychus.hp     0.127830620 -0.129382206  0.21934737 -0.134566205
## Puma.hp             0.220875651 -0.187273416  0.20841097  0.209217638
## Shishugounykus.hp   0.020215414  0.072555462 -0.11702330  0.017541737
## Tamandua.hp         0.016689140 -0.003234623  0.05236715 -0.105569799
## Therizinosaurus.hp  0.003082724 -0.473592433 -0.28737375 -0.190223368
## Tugulusaurus.hp    -0.438832950  0.313638344 -0.31963605 -0.250232706
##                           PC13         PC14          PC15         PC16
## Allosaurus.p        0.10938645 -0.150595772 -0.0573629342 -0.039946941
## Alxasaurus.p        0.13014705  0.064566287  0.0354309763 -0.016669414
## Aorun.p            -0.08082073 -0.156161352  0.0555528244 -0.019717246
## Bannykus.p         -0.08500040 -0.155994204 -0.0153088967 -0.113004156
## Beipiaosaurus.p     0.05736425  0.033451787  0.0027172508  0.023125208
## Eremotherium.p      0.08322284 -0.190639056 -0.1847676015  0.002559593
## Erliansaurus.p     -0.07373892  0.021504676 -0.1092085856  0.008676733
## Falcarius.p         0.12428602 -0.027372299 -0.0225119148  0.074766514
## Guanlong.p         -0.13087444 -0.192887927 -0.0883559950 -0.021317720
## Haplocheirus.p     -0.14029515  0.281705584 -0.1198150104 -0.050454751
## Linhenykus.p        0.02943073 -0.002269575  0.0598868403  0.047479242
## Manis.p             0.05209250 -0.149214275 -0.0199728000  0.081321240
## Mononykus.p        -0.02161497 -0.137208190  0.0450995154 -0.002322071
## Nothronychus.p      0.36861353  0.071326588  0.1314113227 -0.072581530
## Puma.p             -0.15217950 -0.032485041 -0.0379217150 -0.062558341
## Shishugounykus.p    0.43833068  0.135294346 -0.0286599001  0.036417847
## Tamandua.p          0.04828163 -0.215166306  0.0225205984  0.174581411
## Therizinosaurus.p   0.20380363  0.069617050 -0.0231409014  0.034525255
## Tugulusaurus.p     -0.11385152  0.066917771 -0.0435229764 -0.018122600
## Allosaurus.sd      -0.05301759 -0.177998730  0.0130439610  0.090624055
## Alxasaurus.sd       0.07702712 -0.006808337  0.2671911968  0.021873503
## Aorun.sd           -0.08867484  0.162137448  0.1038939326 -0.070047399
## Bannykus.sd        -0.15508172 -0.038277176 -0.1483160839 -0.079641319
## Beipiaosaurus.sd   -0.21302969  0.036382380 -0.0251518307  0.149065268
## Eremotherium.sd     0.32835207 -0.089788648  0.3264258406 -0.069750321
## Erliansaurus.sd    -0.08469250  0.049757845 -0.1726201308 -0.079744992
## Falcarius.sd       -0.01247030  0.203536699  0.1476144495 -0.238045651
## Guanlong.sd        -0.07699771  0.133683503 -0.0622374140  0.167080315
## Haplocheirus.sd     0.04447058  0.128889875  0.0030925941 -0.071698991
## Linhenykus.sd       0.02930159 -0.002377017  0.0599419542  0.047429258
## Manis.sd            0.15098903 -0.113646749 -0.1764187217  0.105419816
## Mononykus.sd       -0.02157213 -0.137113898  0.0450584550 -0.002320364
## Nothronychus.sd    -0.13807214 -0.162183114 -0.1008871416 -0.195836522
## Puma.sd            -0.17472510  0.108082582  0.0109097767  0.064358636
## Shishugounykus.sd   0.01731880  0.110177234 -0.0118054199  0.156962965
## Tamandua.sd         0.24938339 -0.153246202 -0.1567192303 -0.161859378
## Therizinosaurus.sd -0.07370417 -0.039977732 -0.0591481585  0.020311020
## Tugulusaurus.sd    -0.05465593  0.020529926 -0.0715119439 -0.040074685
## Allosaurus.hp      -0.02708907 -0.007276475  0.0001331352  0.070434391
## Alxasaurus.hp       0.12498781  0.153773619 -0.0684264468 -0.005716353
## Aorun.hp            0.04318925  0.089067432 -0.0404720445  0.045940796
## Bannykus.hp         0.04182751 -0.099130378 -0.0860208414  0.017275086
## Beipiaosaurus.hp   -0.05736776  0.141877010  0.1125757282 -0.066445182
## Eremotherium.hp    -0.16058491  0.109809993  0.0852522728  0.054201146
## Erliansaurus.hp    -0.03540138 -0.091236083  0.1442739540 -0.043089671
## Falcarius.hp       -0.20361364  0.091263440  0.0138397441  0.013099619
## Guanlong.hp        -0.07534909 -0.023191332 -0.0062323973  0.009253618
## Haplocheirus.hp    -0.01438662  0.126225897 -0.0620266064  0.006603227
## Linhenykus.hp       0.11321864 -0.112318904  0.0219738815 -0.040270643
## Manis.hp            0.03684827  0.163504902  0.1359920699  0.020235594
## Mononykus.hp        0.08909380 -0.040368257 -0.0108226388  0.037359642
## Nothronychus.hp     0.11675077  0.298286162 -0.2749764795 -0.002950107
## Puma.hp            -0.45427638 -0.038390393  0.0221553637 -0.063083882
## Shishugounykus.hp  -0.14978518 -0.077888488  0.1101635059 -0.070589398
## Tamandua.hp        -0.13001937  0.016131391  0.2084374288  0.112639754
## Therizinosaurus.hp -0.18438123 -0.037469097  0.1719593324  0.043418496
## Tugulusaurus.hp     0.32960612 -0.028820422 -0.0722051443 -0.019179626
##                            PC17         PC18          PC19          PC20
## Allosaurus.p        0.044990442 -0.045148668 -0.0204655742  0.0060985902
## Alxasaurus.p        0.017833168 -0.010859171  0.0170464640 -0.0009530141
## Aorun.p            -0.048662318 -0.103330519  0.0322027112  0.0176981366
## Bannykus.p         -0.140877069  0.025481889 -0.1000631917 -0.0700836420
## Beipiaosaurus.p    -0.185371574  0.033011507  0.0973180013 -0.0720619612
## Eremotherium.p     -0.042009761 -0.113297634 -0.0121342883 -0.0332025488
## Erliansaurus.p      0.051348707  0.090966901 -0.0093099801  0.0574245569
## Falcarius.p        -0.033273942  0.069408011  0.0720125336 -0.0455357208
## Guanlong.p          0.029917232  0.001559148 -0.0073474126 -0.0334919846
## Haplocheirus.p      0.108861973 -0.040626645 -0.0202955782  0.0599492594
## Linhenykus.p       -0.018717663 -0.021482128 -0.0280688996 -0.0149087029
## Manis.p             0.061089058  0.028204546  0.0352922190  0.1041541196
## Mononykus.p         0.002832537 -0.013745755 -0.0069011749 -0.0179034407
## Nothronychus.p      0.074441067  0.052541069 -0.1047569708  0.0140909397
## Puma.p              0.011764513 -0.001822747 -0.0386359281  0.0069054785
## Shishugounykus.p   -0.006020648  0.005925455 -0.0095848993 -0.0282432027
## Tamandua.p          0.059412573  0.041798138  0.0536877739 -0.1072426197
## Therizinosaurus.p   0.080800333 -0.041617702 -0.0698262967  0.0149405131
## Tugulusaurus.p      0.033019236  0.004716875  0.0007509656 -0.0301963562
## Allosaurus.sd       0.082606782 -0.079160603 -0.0447604633  0.1001508467
## Alxasaurus.sd      -0.001108646 -0.110863738  0.0623560309  0.0875570516
## Aorun.sd           -0.076086061  0.032576324 -0.0323070345  0.0658504084
## Bannykus.sd         0.034469682 -0.015588057  0.0590536056  0.0218282271
## Beipiaosaurus.sd   -0.073120625 -0.084752458 -0.1437747088 -0.0514175422
## Eremotherium.sd    -0.083520969  0.014143989  0.0138020124  0.0098463425
## Erliansaurus.sd    -0.030486754  0.142291288  0.0764830909  0.0216695413
## Falcarius.sd        0.152164429 -0.050062330 -0.0100666293 -0.1478253355
## Guanlong.sd        -0.056929868 -0.044033694 -0.0214028000  0.0277560705
## Haplocheirus.sd    -0.004811256 -0.047816755  0.1388752675  0.0422186572
## Linhenykus.sd      -0.018723544 -0.021464548 -0.0280690126 -0.0149029653
## Manis.sd            0.063411190  0.027486521 -0.0695610446  0.0063541067
## Mononykus.sd        0.002886239 -0.013663078 -0.0069965513 -0.0178028774
## Nothronychus.sd     0.049249293 -0.074285820  0.0680861871  0.0157948637
## Puma.sd            -0.010261469  0.010402283 -0.0186635519  0.0220972205
## Shishugounykus.sd  -0.017461047 -0.002089981 -0.0206880866  0.0272811494
## Tamandua.sd        -0.116186816  0.011327966 -0.0909364842  0.0697542868
## Therizinosaurus.sd -0.072138593  0.053632549  0.0404650537  0.0012311010
## Tugulusaurus.sd    -0.009724961 -0.024924747  0.0055211630  0.0081744882
## Allosaurus.hp       0.038180508 -0.061843591  0.0150792672 -0.0294981282
## Alxasaurus.hp       0.004517803  0.027357517 -0.0033666790  0.0135160281
## Aorun.hp            0.048660400  0.020968910  0.0557281460 -0.0258518516
## Bannykus.hp         0.079684645  0.096751415 -0.0067351645 -0.0261713344
## Beipiaosaurus.hp   -0.052534781  0.034350017 -0.0842504406  0.0839180116
## Eremotherium.hp    -0.049300410 -0.030517834  0.0090333190 -0.1134700993
## Erliansaurus.hp     0.027461874 -0.009579947  0.0227435709 -0.0291868954
## Falcarius.hp       -0.031790417  0.132103498 -0.0334331640  0.0034845962
## Guanlong.hp         0.011883189  0.072233516  0.0208750795  0.0330128132
## Haplocheirus.hp     0.075832908  0.041287620 -0.0673664404 -0.0811921909
## Linhenykus.hp       0.002408639  0.065621812  0.0560241892  0.0269285488
## Manis.hp           -0.045087797  0.010182431 -0.0064308351  0.0026513587
## Mononykus.hp       -0.010712689 -0.037897198  0.0210688406 -0.0181325963
## Nothronychus.hp    -0.077755660 -0.128332363  0.0794520034  0.0005151404
## Puma.hp            -0.023553504  0.016669352 -0.0117637905  0.0111677086
## Shishugounykus.hp  -0.024790850 -0.015732372 -0.0463772056  0.0048763618
## Tamandua.hp         0.034026147  0.022201701  0.0516128875  0.0686013929
## Therizinosaurus.hp  0.071952484  0.034137345  0.0509634709 -0.0297733746
## Tugulusaurus.hp     0.005312641  0.025200492  0.0188064271 -0.0184495309
##                             PC21          PC22          PC23          PC24
## Allosaurus.p        0.0367968840  0.0133667412 -0.0318069109  0.0218937120
## Alxasaurus.p        0.0007444127  0.0101766940 -0.0110465601  0.0135246966
## Aorun.p             0.0190576860  0.0009808852  0.0021340477  0.0105605457
## Bannykus.p          0.0054620217 -0.0032003555 -0.0250634251 -0.0129954378
## Beipiaosaurus.p    -0.1328784961 -0.0323385474 -0.0414622152  0.0275790455
## Eremotherium.p     -0.0075103341 -0.0823761485  0.0034820837 -0.0007426719
## Erliansaurus.p     -0.0463408604 -0.0328376125 -0.0580997904  0.0352928692
## Falcarius.p         0.0327337723  0.0037192445 -0.0010008004 -0.1081677422
## Guanlong.p          0.0124784976 -0.0380966786  0.0233328618  0.0395947313
## Haplocheirus.p     -0.0557183466  0.0352525741  0.0240248946 -0.0060526498
## Linhenykus.p        0.0110744493 -0.0212165553  0.0032059817  0.0026317188
## Manis.p            -0.0117578754  0.0633312658 -0.0321867830  0.0053649428
## Mononykus.p        -0.0095813093  0.0203546187  0.0207887594  0.0153615327
## Nothronychus.p     -0.0377660159 -0.0358789205  0.0322523088  0.0716613407
## Puma.p             -0.0128755539 -0.0008887828  0.0311442139 -0.0274947322
## Shishugounykus.p    0.0227516734  0.0134995938  0.0098104058  0.0064410480
## Tamandua.p          0.0323689044  0.0494287910 -0.0023609567  0.0500353550
## Therizinosaurus.p  -0.1123434662 -0.0074374127 -0.0202029372 -0.0132872753
## Tugulusaurus.p      0.0006013535 -0.0155923039 -0.0060360726  0.0216520064
## Allosaurus.sd       0.0487823813  0.0264871460 -0.0592212493 -0.0146160138
## Alxasaurus.sd       0.0105090295 -0.0091144883  0.0215975056 -0.0558529888
## Aorun.sd            0.0328359303 -0.0400355946 -0.0680832849  0.0464088310
## Bannykus.sd         0.0077687293  0.0862095200  0.0767804271  0.0163376659
## Beipiaosaurus.sd   -0.0841828030  0.0498880381  0.0711565021  0.0077289236
## Eremotherium.sd    -0.0002979911  0.0140988922  0.0041034580 -0.0155030949
## Erliansaurus.sd     0.0252044725 -0.0126261327  0.0685231057 -0.0178170018
## Falcarius.sd        0.0227769292  0.0275435664  0.0129121728 -0.0324996711
## Guanlong.sd         0.0591665648 -0.0682763867  0.0611508832 -0.0352824352
## Haplocheirus.sd     0.0645806904 -0.0459219808  0.0717663315  0.0945493872
## Linhenykus.sd       0.0110573433 -0.0212062003  0.0032106867  0.0026392637
## Manis.sd            0.0255262533 -0.0363795049 -0.0143721252  0.0133658444
## Mononykus.sd       -0.0096371103  0.0203897855  0.0209664385  0.0154148330
## Nothronychus.sd    -0.0518176955 -0.0738724510 -0.0462934296 -0.0184552737
## Puma.sd             0.0164522755  0.0132817846  0.0006643622  0.0328252292
## Shishugounykus.sd   0.0203659406 -0.0163239941  0.0532700751 -0.0051142433
## Tamandua.sd         0.0522511597  0.0479945417  0.0165185473 -0.0173887872
## Therizinosaurus.sd  0.0692390918  0.0034579398  0.0136841303  0.0083374258
## Tugulusaurus.sd     0.0072069176 -0.0129525477 -0.0136524032 -0.0447512464
## Allosaurus.hp       0.0307754855  0.0384330766 -0.0528649792  0.0135064226
## Alxasaurus.hp      -0.0291955932  0.0060966437  0.0300332229 -0.0011843611
## Aorun.hp           -0.0114300764  0.0180575827 -0.0326344569 -0.0113448373
## Bannykus.hp        -0.0722730115 -0.0422362888  0.0368462907 -0.0587676831
## Beipiaosaurus.hp    0.0405313404 -0.0208417871 -0.0143053223 -0.0179265556
## Eremotherium.hp     0.0020201532  0.0116198058 -0.0252957812  0.0255596759
## Erliansaurus.hp     0.0046202382  0.0105073249  0.0324379027 -0.0256178548
## Falcarius.hp        0.0279733571  0.0660690900 -0.0545965705 -0.0087767382
## Guanlong.hp        -0.0324739743  0.0301625929  0.0051612722  0.0022533525
## Haplocheirus.hp     0.1029887331 -0.0714033790 -0.0391992478 -0.0143548531
## Linhenykus.hp      -0.0439729748  0.0200009174  0.0010175792  0.0150231178
## Manis.hp            0.0017559865  0.0236845616 -0.0026749105 -0.0027713171
## Mononykus.hp       -0.0055399351 -0.0073855632  0.0060171774  0.0006088582
## Nothronychus.hp    -0.0049177549  0.0501637172 -0.0725162375 -0.0146223530
## Puma.hp            -0.0284288308 -0.0065731622 -0.0017946589 -0.0043987452
## Shishugounykus.hp  -0.0206756159  0.0353186509  0.0023198581  0.0103296391
## Tamandua.hp        -0.0666900109 -0.0559411262 -0.0024146780 -0.0205657866
## Therizinosaurus.hp  0.0417457386 -0.0172482344 -0.0239667905 -0.0062826874
## Tugulusaurus.hp    -0.0118987609  0.0186265534 -0.0071609098 -0.0138469767
##                              PC25          PC26          PC27          PC28
## Allosaurus.p        0.00564281831  0.0050204919  0.0184211125  0.0099410571
## Alxasaurus.p        0.03105046223 -0.0156716863  0.0078327196 -0.0001596976
## Aorun.p            -0.01758108535 -0.0156602188 -0.0409235156  0.0473135840
## Bannykus.p         -0.07759029899  0.0140516869  0.0497897248 -0.0215654362
## Beipiaosaurus.p    -0.01822781071 -0.0236169068  0.0299787026  0.0196625642
## Eremotherium.p      0.01824195655  0.0321357145  0.0322132329  0.0400244627
## Erliansaurus.p     -0.02464523872 -0.0055782158 -0.0468307113  0.0023094393
## Falcarius.p        -0.02020770765 -0.0481177897  0.0012866541 -0.0190025335
## Guanlong.p          0.00003562318 -0.0085049565 -0.0382268677  0.0131088158
## Haplocheirus.p     -0.05710141577 -0.0118442125  0.0569797500  0.0559296615
## Linhenykus.p       -0.01987379651  0.0025563785 -0.0184029300 -0.0024027247
## Manis.p             0.03444219431  0.0321345886  0.0226636416 -0.0185709946
## Mononykus.p         0.00461198159  0.0276874639 -0.0102025980 -0.0285697070
## Nothronychus.p     -0.04262578741 -0.0707282619 -0.0192639351 -0.0186944821
## Puma.p             -0.00207812545  0.0109985896 -0.0008746969 -0.0055082244
## Shishugounykus.p    0.00097312752  0.0041321066  0.0113056532 -0.0020311370
## Tamandua.p         -0.00581454207 -0.0284442842  0.0203898315  0.0247537767
## Therizinosaurus.p  -0.01492654697  0.0076660272  0.0024324773 -0.0016594017
## Tugulusaurus.p     -0.01385067943  0.0001904058  0.0055191946 -0.0096589774
## Allosaurus.sd      -0.03220284537 -0.0380084972  0.0042240394 -0.0287502110
## Alxasaurus.sd      -0.01520231976 -0.0324335741 -0.0002571789  0.0465752797
## Aorun.sd            0.06275467804  0.0219589749 -0.0403268746  0.0266575531
## Bannykus.sd        -0.07712960253  0.0432219308 -0.0300034877 -0.0018718280
## Beipiaosaurus.sd    0.07360556479 -0.0231413628 -0.0022794749 -0.0159451626
## Eremotherium.sd    -0.02144085032  0.0217867460 -0.0224772550  0.0017043107
## Erliansaurus.sd     0.01771030911 -0.0146665620 -0.0205431746  0.0145207174
## Falcarius.sd        0.06698843561  0.0159743665  0.0093610276 -0.0066237146
## Guanlong.sd        -0.00944134862 -0.0323791334  0.0160552660 -0.0594201148
## Haplocheirus.sd     0.01501339258 -0.0180451763  0.0380870591 -0.0370776256
## Linhenykus.sd      -0.01987639045  0.0025525738 -0.0184025692 -0.0024001882
## Manis.sd            0.01624368664  0.0363583924  0.0090890951 -0.0033914830
## Mononykus.sd        0.00472280103  0.0274682852 -0.0099505531 -0.0284037614
## Nothronychus.sd     0.02132790090 -0.0349942604  0.0171567118 -0.0483013649
## Puma.sd            -0.00522784294 -0.0395735633  0.0080578868  0.0024733757
## Shishugounykus.sd   0.02974122616  0.0424792546 -0.0000378332  0.0403706927
## Tamandua.sd         0.03062981227  0.0112232577 -0.0093588771 -0.0065205502
## Therizinosaurus.sd -0.00235145349 -0.0054561798  0.0012270160  0.0115233913
## Tugulusaurus.sd     0.01130823472 -0.0204404270 -0.0278626948  0.0196599991
## Allosaurus.hp       0.02143774556 -0.0396793369 -0.0221861943  0.0024861141
## Alxasaurus.hp      -0.02163263450  0.0265777838 -0.0270603369 -0.0148807928
## Aorun.hp            0.00268595732 -0.0118569779 -0.0206773687 -0.0298539004
## Bannykus.hp         0.02696604876 -0.0289790260 -0.0424556632  0.0233295203
## Beipiaosaurus.hp    0.02696318873 -0.0081768908  0.0113229773 -0.0012654003
## Eremotherium.hp    -0.01361554906  0.0297821364 -0.0427679232 -0.0219871419
## Erliansaurus.hp    -0.00617200820  0.0150701395  0.0037984459 -0.0096102468
## Falcarius.hp        0.03007014347 -0.0233501677  0.0361446873 -0.0067993685
## Guanlong.hp         0.00012725037  0.0136884970  0.0147039774  0.0114402080
## Haplocheirus.hp    -0.04769253374  0.0397478934  0.0216085416  0.0303717856
## Linhenykus.hp       0.04209784952  0.0126491408  0.0426803110  0.0123186315
## Manis.hp           -0.02400974396  0.0027004552  0.0185054387  0.0027346467
## Mononykus.hp        0.04808222302 -0.0052378820  0.0220479420  0.0245753555
## Nothronychus.hp    -0.00856884841  0.0211682548 -0.0304419165 -0.0110597651
## Puma.hp             0.00431749294 -0.0094107909  0.0013490336 -0.0067869253
## Shishugounykus.hp  -0.02767415741 -0.0152146896  0.0027191798  0.0234781525
## Tamandua.hp        -0.00680707212  0.0912459232  0.0108369085 -0.0343213576
## Therizinosaurus.hp  0.00210147796  0.0092368178 -0.0043715863  0.0098453615
## Tugulusaurus.hp     0.00367465274  0.0077467534 -0.0016020226 -0.0140142372
##                             PC29          PC30          PC31          PC32
## Allosaurus.p       -0.0309438765 -0.0313284451  0.0077000854 -0.0296198479
## Alxasaurus.p       -0.0088164773 -0.0131197326  0.0045094048  0.0059425737
## Aorun.p            -0.0114471358  0.0169134526 -0.0136594092  0.0033824538
## Bannykus.p         -0.0035640623 -0.0177266027 -0.0028903514  0.0019343084
## Beipiaosaurus.p    -0.0197661055 -0.0081519140 -0.0203134258  0.0212559168
## Eremotherium.p     -0.0046586573  0.0269936242 -0.0004358998  0.0095398569
## Erliansaurus.p     -0.0299681842 -0.0251699103 -0.0162831587 -0.0200408785
## Falcarius.p         0.0354555448  0.0144458886  0.0205367197 -0.0268730533
## Guanlong.p          0.0107380139 -0.0140129433 -0.0158299787 -0.0116868280
## Haplocheirus.p      0.0090176653 -0.0119864647  0.0357888410  0.0075494984
## Linhenykus.p        0.0005648327 -0.0087649870 -0.0080982781  0.0139875215
## Manis.p            -0.0027264021  0.0117409591 -0.0096078484 -0.0029189705
## Mononykus.p        -0.0140617871  0.0068899394 -0.0012120558 -0.0082887878
## Nothronychus.p     -0.0056885633  0.0443791729 -0.0080593522 -0.0139096040
## Puma.p              0.0124453425  0.0253588256 -0.0005645877  0.0266963822
## Shishugounykus.p    0.0059200172 -0.0031942532  0.0122728951  0.0009418265
## Tamandua.p          0.0139657887 -0.0119053220  0.0040328728 -0.0052439387
## Therizinosaurus.p  -0.0096349144 -0.0030627203  0.0021348992 -0.0108410363
## Tugulusaurus.p      0.0227651887 -0.0114723306  0.0146632659  0.0113743936
## Allosaurus.sd       0.0419990734  0.0264796421 -0.0001010705  0.0504760672
## Alxasaurus.sd      -0.0447713063 -0.0011610947 -0.0134230723 -0.0133799639
## Aorun.sd           -0.0017498842  0.0248053913  0.0653716212  0.0011462488
## Bannykus.sd        -0.0130830364 -0.0309662484  0.0263076917 -0.0099619640
## Beipiaosaurus.sd    0.0331767018  0.0070065574  0.0148420780 -0.0248620125
## Eremotherium.sd     0.0459876045 -0.0221063533  0.0296957236 -0.0002919214
## Erliansaurus.sd    -0.0017429711  0.0150316371  0.0024487262  0.0278146774
## Falcarius.sd       -0.0296195490 -0.0032664713 -0.0248588999  0.0244761003
## Guanlong.sd        -0.0802456818 -0.0103122634  0.0233774391  0.0042750766
## Haplocheirus.sd     0.0295405918  0.0067242575 -0.0109416961 -0.0106833199
## Linhenykus.sd       0.0005688609 -0.0087596155 -0.0080994754  0.0139751719
## Manis.sd           -0.0054011476 -0.0206624085 -0.0132555614  0.0291709112
## Mononykus.sd       -0.0139480506  0.0071100523 -0.0014052302 -0.0084613376
## Nothronychus.sd     0.0356231085 -0.0166585915  0.0196012666 -0.0234123406
## Puma.sd             0.0125191970 -0.0086827807 -0.0156781634  0.0175784267
## Shishugounykus.sd   0.0354451561 -0.0026435753 -0.0231734992 -0.0274308911
## Tamandua.sd        -0.0232407342  0.0081178632  0.0054609436  0.0067736327
## Therizinosaurus.sd  0.0008732107  0.0068095772 -0.0118065340  0.0054421715
## Tugulusaurus.sd    -0.0120163101 -0.0132670949 -0.0055008681 -0.0236444030
## Allosaurus.hp      -0.0223431434 -0.0150844479  0.0037753073  0.0040228152
## Alxasaurus.hp       0.0068241401 -0.0002469539 -0.0308328953  0.0049610339
## Aorun.hp           -0.0147368311  0.0043420149  0.0169890350  0.0032644315
## Bannykus.hp         0.0124046947 -0.0055624278  0.0020258407  0.0208079876
## Beipiaosaurus.hp    0.0385502745 -0.0749033957 -0.0383698487  0.0006047555
## Eremotherium.hp    -0.0027457705 -0.0082947391  0.0230919649  0.0139958493
## Erliansaurus.hp     0.0081053702  0.0202931739 -0.0233761730 -0.0089582337
## Falcarius.hp       -0.0249876963  0.0205838431 -0.0202549285 -0.0259737596
## Guanlong.hp         0.0021494579 -0.0021269171  0.0168645567  0.0006541375
## Haplocheirus.hp     0.0119390955  0.0356360739  0.0074752300 -0.0287646272
## Linhenykus.hp      -0.0183462764  0.0176515281  0.0049029416  0.0058730185
## Manis.hp           -0.0001050664  0.0014998395  0.0078254903  0.0179333504
## Mononykus.hp        0.0024131033 -0.0210773884  0.0242471322  0.0095019911
## Nothronychus.hp     0.0256654024  0.0272634993 -0.0332249493 -0.0061120476
## Puma.hp             0.0043735314  0.0179679005 -0.0047246644 -0.0083290636
## Shishugounykus.hp  -0.0070789535  0.0306012560 -0.0128491649 -0.0053728641
## Tamandua.hp        -0.0076133741  0.0127322872 -0.0047954566 -0.0022666944
## Therizinosaurus.hp  0.0031021297 -0.0080322132 -0.0027939525  0.0024585070
## Tugulusaurus.hp     0.0029188509 -0.0036676503  0.0004784771 -0.0104827043
##                             PC33          PC34           PC35           PC36
## Allosaurus.p        0.0284838331 -0.0285567261  0.01127436409 -0.00645031729
## Alxasaurus.p        0.0144269473 -0.0108404439 -0.00500570529  0.01827112561
## Aorun.p            -0.0195882722 -0.0103132898 -0.00135005899 -0.01836084185
## Bannykus.p          0.0031117830  0.0116414263  0.00349214216  0.01040758974
## Beipiaosaurus.p     0.0172545258 -0.0008280636 -0.02097676291 -0.01519112284
## Eremotherium.p     -0.0194684627  0.0015824926  0.01918384515  0.00320041152
## Erliansaurus.p     -0.0118349608 -0.0064044870 -0.00040754548  0.03601845069
## Falcarius.p        -0.0150345569 -0.0148154308 -0.01431288947  0.00239883275
## Guanlong.p          0.0103198847  0.0312351254 -0.03711596500 -0.00449417622
## Haplocheirus.p     -0.0229790960 -0.0137055605 -0.02128088905 -0.00789267834
## Linhenykus.p        0.0117082085  0.0033380735  0.00610651940 -0.00382172049
## Manis.p            -0.0130614181  0.0354237699 -0.00219366024 -0.01256442245
## Mononykus.p        -0.0160661867 -0.0140565625 -0.00888895115 -0.00772818178
## Nothronychus.p     -0.0018886578 -0.0026222225  0.00004805787 -0.01128396750
## Puma.p             -0.0105305059  0.0167487773  0.01699288552  0.01566156299
## Shishugounykus.p   -0.0133480729  0.0072919812 -0.01034952143  0.01184207348
## Tamandua.p         -0.0061690884  0.0160570327  0.00808056962  0.00726716406
## Therizinosaurus.p  -0.0043542666 -0.0033197366  0.00585945361  0.00275241139
## Tugulusaurus.p     -0.0219576959  0.0152899590 -0.00307448069  0.01010839382
## Allosaurus.sd       0.0380204783 -0.0146601295 -0.02535402586  0.00569866361
## Alxasaurus.sd       0.0026382565  0.0255374717  0.00562820450  0.01036010503
## Aorun.sd           -0.0017655558  0.0135057121 -0.00702669274 -0.00001248232
## Bannykus.sd         0.0142329081  0.0071474838  0.00412148072  0.00207360929
## Beipiaosaurus.sd    0.0187848780  0.0025835600  0.01730688571 -0.00290779894
## Eremotherium.sd    -0.0039501529  0.0034251039 -0.00345851671 -0.01109822992
## Erliansaurus.sd     0.0012643602 -0.0234446210  0.01286009097 -0.01403147217
## Falcarius.sd        0.0067001265  0.0041125262 -0.01978415328 -0.00617173930
## Guanlong.sd        -0.0020492652  0.0113617016 -0.01448891661 -0.00209233082
## Haplocheirus.sd     0.0132338924 -0.0094392144  0.01008675632  0.01669927640
## Linhenykus.sd       0.0117090319  0.0033372131  0.00610651915 -0.00381849485
## Manis.sd           -0.0103099682 -0.0070595752 -0.00387456447 -0.01226806816
## Mononykus.sd       -0.0162521167 -0.0141928293 -0.00879384501 -0.00773748030
## Nothronychus.sd    -0.0078362573  0.0061445084 -0.00137794387 -0.01258645849
## Puma.sd            -0.0360470017 -0.0054337683  0.01910664838 -0.02653356812
## Shishugounykus.sd   0.0020883456 -0.0071677923 -0.03652344764  0.00205783857
## Tamandua.sd        -0.0080333471 -0.0033212278 -0.00465078326  0.00560863368
## Therizinosaurus.sd -0.0003684639 -0.0004943154 -0.00150969223  0.00124936505
## Tugulusaurus.sd     0.0250136168 -0.0091444110  0.00514596819 -0.01806518983
## Allosaurus.hp      -0.0063747743 -0.0201001268  0.01951035570 -0.00051103688
## Alxasaurus.hp       0.0121355860  0.0201981996 -0.00055238600 -0.01035618569
## Aorun.hp           -0.0043020645  0.0314104425  0.01482953463 -0.01221056274
## Bannykus.hp         0.0107795924 -0.0022914240  0.01090522979  0.02016088015
## Beipiaosaurus.hp   -0.0084622731  0.0000098691  0.01308959351 -0.00068796744
## Eremotherium.hp    -0.0061264865 -0.0235061286 -0.00938662064  0.00908810714
## Erliansaurus.hp    -0.0164245294 -0.0066332926  0.01030289112  0.01163404665
## Falcarius.hp       -0.0054314803 -0.0028056162 -0.00970324808  0.00137705329
## Guanlong.hp         0.0319070220  0.0049114670  0.01290660170 -0.01677271311
## Haplocheirus.hp     0.0221683232  0.0054369560  0.01423282840 -0.00965757150
## Linhenykus.hp       0.0177612531 -0.0010437104  0.00000301469  0.00598197065
## Manis.hp            0.0152453295  0.0063523862  0.02065592318  0.00822673439
## Mononykus.hp       -0.0037649555  0.0014124392 -0.00381033528  0.01578857957
## Nothronychus.hp     0.0017887767  0.0051728452  0.00602763340  0.00772936623
## Puma.hp             0.0045259256  0.0053513229 -0.00916440816  0.00775458055
## Shishugounykus.hp  -0.0073519329 -0.0091488148 -0.00249160969  0.01390689260
## Tamandua.hp         0.0001805210 -0.0246429117  0.00646152510 -0.00133895056
## Therizinosaurus.hp -0.0064791263  0.0044455676  0.00580450324 -0.00190311945
## Tugulusaurus.hp    -0.0078724136 -0.0104729810  0.00077759341 -0.00477486958
##                             PC37          PC38           PC39          PC40
## Allosaurus.p        0.0015210254  0.0109858542  0.01681678756 -0.0098256986
## Alxasaurus.p        0.0073564166 -0.0152227665 -0.00447313174  0.0171651699
## Aorun.p             0.0099368087  0.0131603270 -0.00238891728  0.0046990344
## Bannykus.p         -0.0046332002  0.0201842946 -0.01198130967  0.0056672025
## Beipiaosaurus.p    -0.0080795016 -0.0063011032 -0.00272114698 -0.0077245770
## Eremotherium.p     -0.0107235931 -0.0094097868  0.01062050798 -0.0034004466
## Erliansaurus.p     -0.0114443271 -0.0014107118  0.01741173030  0.0036506210
## Falcarius.p         0.0112272360  0.0119154988  0.01726055460  0.0058322000
## Guanlong.p          0.0091771850 -0.0031072062  0.01108546810  0.0143759203
## Haplocheirus.p      0.0084445049 -0.0083752025  0.00400069815 -0.0007085811
## Linhenykus.p        0.0023398259 -0.0013483815  0.00845248447 -0.0027329371
## Manis.p             0.0046081731  0.0231629995  0.00870862989 -0.0176282829
## Mononykus.p        -0.0089965524 -0.0221128549 -0.00303433762 -0.0016529467
## Nothronychus.p      0.0084301347  0.0079080922 -0.00256611255 -0.0071658018
## Puma.p             -0.0121071050 -0.0127471053  0.00347121313 -0.0146514849
## Shishugounykus.p   -0.0112001697  0.0052148761 -0.01430915984 -0.0027112907
## Tamandua.p          0.0087853519 -0.0147360791  0.00237147546 -0.0054231055
## Therizinosaurus.p  -0.0014654096 -0.0010401834 -0.00002388105  0.0015064497
## Tugulusaurus.p      0.0071760169  0.0010716725  0.00408427054 -0.0071742803
## Allosaurus.sd      -0.0172189273 -0.0063279942 -0.00268985962 -0.0008688276
## Alxasaurus.sd      -0.0016281265 -0.0022020413 -0.01638461304  0.0003777225
## Aorun.sd            0.0053684556 -0.0013887251 -0.00464544154  0.0038366245
## Bannykus.sd        -0.0069926623  0.0007441549 -0.01151614984  0.0022600554
## Beipiaosaurus.sd    0.0016241576 -0.0018606968  0.00313959569  0.0052425395
## Eremotherium.sd    -0.0028860768 -0.0122320728  0.01555691072 -0.0006191340
## Erliansaurus.sd    -0.0007391998 -0.0078243179  0.00146886119 -0.0061755735
## Falcarius.sd       -0.0042310746  0.0097124784  0.01362176086  0.0009054272
## Guanlong.sd         0.0137292953 -0.0070620166  0.01046673867 -0.0041735886
## Haplocheirus.sd    -0.0119684603  0.0089543436  0.00180216533  0.0019774698
## Linhenykus.sd       0.0023407777 -0.0013466352  0.00845816483 -0.0027252692
## Manis.sd            0.0175942741  0.0016825732 -0.01257888655  0.0187696497
## Mononykus.sd       -0.0092693123 -0.0220638455 -0.00318217004 -0.0017049240
## Nothronychus.sd     0.0074338415 -0.0025280719 -0.01704365494 -0.0028826488
## Puma.sd            -0.0178936948  0.0180119233  0.00170938075  0.0152837143
## Shishugounykus.sd  -0.0144663783  0.0097505245 -0.01638617860 -0.0076191827
## Tamandua.sd         0.0015640879  0.0062728031 -0.00137675427  0.0005262447
## Therizinosaurus.sd  0.0021893895  0.0001860945  0.00219132314 -0.0009856353
## Tugulusaurus.sd    -0.0240303187  0.0005413950 -0.00373391056  0.0010468609
## Allosaurus.hp       0.0162686590  0.0006643274 -0.01585047316 -0.0118209102
## Alxasaurus.hp       0.0128487442 -0.0016024736 -0.00371245345 -0.0066476896
## Aorun.hp           -0.0367951206 -0.0020569702 -0.00222624110  0.0115938095
## Bannykus.hp         0.0001543497  0.0115956933 -0.01004910168 -0.0038025370
## Beipiaosaurus.hp    0.0058275521 -0.0141167302  0.00224723936 -0.0084847966
## Eremotherium.hp     0.0040209991  0.0158071498 -0.00936324026 -0.0129432845
## Erliansaurus.hp     0.0173360678 -0.0045530870 -0.00872497163  0.0104272458
## Falcarius.hp       -0.0104555887 -0.0117018596 -0.00724349246  0.0002853863
## Guanlong.hp         0.0128575588  0.0033110904  0.01168381045  0.0038652622
## Haplocheirus.hp    -0.0019190700 -0.0066868987 -0.00428847596 -0.0031922485
## Linhenykus.hp       0.0179966537 -0.0024489638 -0.00659199561  0.0042399325
## Manis.hp            0.0089682825  0.0023982168 -0.00448240215  0.0098745075
## Mononykus.hp       -0.0148123519  0.0147245184  0.01004618883  0.0016239578
## Nothronychus.hp     0.0187460997 -0.0056601504  0.00959678264  0.0036499667
## Puma.hp             0.0103360971  0.0072660369 -0.00684097197 -0.0029863677
## Shishugounykus.hp  -0.0067170331  0.0013444683  0.01771744637  0.0026482967
## Tamandua.hp        -0.0010623944  0.0088070713  0.00538234032  0.0059196363
## Therizinosaurus.hp  0.0027191291 -0.0028565382 -0.00235501788 -0.0004268180
## Tugulusaurus.hp    -0.0071915019 -0.0030470078 -0.00660807627  0.0016079611
##                              PC41          PC42           PC43           PC44
## Allosaurus.p        0.01084731055  0.0048645909 -0.00794029757 -0.00354076464
## Alxasaurus.p       -0.00510870241 -0.0138824625 -0.00891024623  0.00107335491
## Aorun.p             0.00905497173 -0.0064534645  0.00174296931  0.01058580614
## Bannykus.p         -0.00003875028 -0.0051551209  0.00182079757  0.00200024528
## Beipiaosaurus.p     0.00527733044 -0.0031141679 -0.00367607552 -0.00067407935
## Eremotherium.p     -0.01928994222  0.0089843269  0.00254942641  0.00483173568
## Erliansaurus.p     -0.00582922444  0.0001599354  0.00027847564  0.00915431939
## Falcarius.p        -0.00940139223 -0.0048079153  0.00005167959  0.00355291303
## Guanlong.p          0.00008346964  0.0032413666  0.00029284583 -0.00503136910
## Haplocheirus.p     -0.00267037557 -0.0051766889 -0.00242085481  0.00034545612
## Linhenykus.p       -0.00394693104  0.0011141396  0.00008608954 -0.00086800341
## Manis.p            -0.00351406440 -0.0056836795 -0.00540180537  0.00025358585
## Mononykus.p        -0.00069696721 -0.0038497671  0.00623821349  0.00266377849
## Nothronychus.p     -0.00011117524  0.0003782620  0.00054785518 -0.00155019122
## Puma.p              0.01481626190 -0.0184571782 -0.01113740169  0.00220917037
## Shishugounykus.p    0.01281954356  0.0072745917 -0.00101960130  0.00646365439
## Tamandua.p         -0.00184665713 -0.0028533382 -0.00156433555  0.00084306752
## Therizinosaurus.p  -0.00091171444  0.0008412247  0.00082910769 -0.00132547112
## Tugulusaurus.p      0.00612606322  0.0099657153  0.00316489339 -0.00929063895
## Allosaurus.sd      -0.00486376418  0.0042202465  0.00170633312 -0.00027177749
## Alxasaurus.sd       0.00020037975  0.0004152458  0.00702292310 -0.00700091797
## Aorun.sd           -0.00143096787 -0.0044669652 -0.00141351237 -0.00132724302
## Bannykus.sd        -0.00428877742  0.0028902488 -0.00023555945 -0.00039890804
## Beipiaosaurus.sd    0.00107930738  0.0009903793  0.00030224801 -0.00010453685
## Eremotherium.sd     0.00302305944  0.0007377906 -0.00234338580  0.00238131225
## Erliansaurus.sd     0.00312889215  0.0112504856  0.00350704699 -0.00493622529
## Falcarius.sd       -0.00198754097  0.0011801293  0.00214716475  0.00624651392
## Guanlong.sd         0.00405234702  0.0022547339 -0.00427504099  0.00186823819
## Haplocheirus.sd     0.00095771269 -0.0085090169  0.00261371509  0.00227833195
## Linhenykus.sd      -0.00394150318  0.0011181408  0.00007890431 -0.00087135189
## Manis.sd            0.01346081712 -0.0040995043  0.00079319402 -0.00256008058
## Mononykus.sd       -0.00049390817 -0.0038914372  0.00631119017  0.00269234327
## Nothronychus.sd     0.00216416747  0.0049183286  0.00058233300  0.00012528785
## Puma.sd            -0.00923825063 -0.0015710767 -0.00583371600 -0.00224653539
## Shishugounykus.sd   0.00204856108  0.0086443421 -0.00485474828  0.00601649127
## Tamandua.sd        -0.00492724910  0.0003798109  0.00000307887 -0.00121464094
## Therizinosaurus.sd  0.00213919083 -0.0017038029 -0.00119558737 -0.00003743176
## Tugulusaurus.sd    -0.00436554282 -0.0095121045 -0.00920390138 -0.00429106442
## Allosaurus.hp      -0.00238694985  0.0007296395  0.00531080029  0.00442206480
## Alxasaurus.hp      -0.01885394053 -0.0092009170  0.00616434135 -0.00420773738
## Aorun.hp            0.00345735161  0.0115357875 -0.00556323670  0.00245076099
## Bannykus.hp         0.00504517952 -0.0027905321  0.00073521699  0.00098518282
## Beipiaosaurus.hp   -0.00199064615  0.0048486333 -0.00072156401  0.00117410664
## Eremotherium.hp    -0.00939030549  0.0005406645 -0.00539681038 -0.00646686675
## Erliansaurus.hp     0.00088731497  0.0025030610 -0.01211673121 -0.00723995478
## Falcarius.hp        0.00121551903 -0.0016224178  0.00790864334 -0.00482480655
## Guanlong.hp         0.00872355225 -0.0020419783  0.01040429328  0.00760864398
## Haplocheirus.hp    -0.00034607357 -0.0039097298  0.00079296579 -0.00110486997
## Linhenykus.hp      -0.01299477025  0.0103666400 -0.01180010892  0.00032983300
## Manis.hp           -0.00236250918  0.0094635574  0.00439673508  0.00770070999
## Mononykus.hp        0.00056049525 -0.0086624234  0.01519352207 -0.01125551991
## Nothronychus.hp     0.00979328515  0.0018433594  0.00083389080 -0.00232626064
## Puma.hp            -0.00019097287  0.0031842354  0.00371705851  0.00798371285
## Shishugounykus.hp   0.00999888208  0.0109784864 -0.00117605464 -0.00728652759
## Tamandua.hp         0.00355565737 -0.0008969915  0.00275729122 -0.00323032884
## Therizinosaurus.hp  0.00215848832  0.0009860932  0.00045229771  0.00005352554
## Tugulusaurus.hp     0.00074445735 -0.0004915122  0.00686303403 -0.00281004265
##                               PC45           PC46           PC47          PC48
## Allosaurus.p        0.006624542271 -0.00652536827 -0.00060968251 -0.0015574121
## Alxasaurus.p        0.003903092518 -0.00547983373  0.00012935161  0.0011076212
## Aorun.p             0.001840228855  0.00359063872 -0.00667312806 -0.0020387324
## Bannykus.p          0.004283193437 -0.00113193078  0.00286606601 -0.0006119127
## Beipiaosaurus.p    -0.001868381319 -0.00149103307  0.00035838637  0.0012698687
## Eremotherium.p     -0.002113236858 -0.00002893137  0.00012558794  0.0003870840
## Erliansaurus.p      0.001011953020  0.00236502901 -0.00059504270  0.0005263996
## Falcarius.p         0.006243130554 -0.00020603049 -0.00076387331  0.0013734821
## Guanlong.p          0.000908258998 -0.00315962299  0.00021971980 -0.0015523949
## Haplocheirus.p     -0.001552862971 -0.00116929940  0.00068781135 -0.0006698116
## Linhenykus.p        0.002055674097  0.00094286765  0.00185930802 -0.0010142143
## Manis.p            -0.004013801981 -0.00402827657  0.00109057374  0.0014420370
## Mononykus.p         0.002194458409 -0.00166928084  0.00264771443  0.0011921524
## Nothronychus.p     -0.001335375788  0.00019602283 -0.00263950866  0.0003568192
## Puma.p              0.005713947409  0.00048112444 -0.00384634632 -0.0003260397
## Shishugounykus.p   -0.000975993298  0.00294397182  0.00479684986 -0.0031015776
## Tamandua.p         -0.002813028382 -0.00009323126 -0.00089294144  0.0010658563
## Therizinosaurus.p   0.000634076909  0.00018217203 -0.00033307675 -0.0001017420
## Tugulusaurus.p      0.009504944761  0.00457270409 -0.00042610803  0.0011630569
## Allosaurus.sd      -0.000297333926  0.00105558070 -0.00015539026 -0.0000217908
## Alxasaurus.sd       0.009930818896 -0.00067137352  0.00183368044  0.0017086353
## Aorun.sd            0.000278644660 -0.00171871062  0.00257325282  0.0012307736
## Bannykus.sd        -0.002977794042  0.00092776903 -0.00104793295 -0.0010913913
## Beipiaosaurus.sd    0.003731217086  0.00043600138 -0.00064610199 -0.0009178709
## Eremotherium.sd    -0.004158798701  0.00052027544  0.00102187545 -0.0041609965
## Erliansaurus.sd     0.003523750494 -0.00428833158 -0.00023441654 -0.0007176123
## Falcarius.sd        0.000822938763  0.00065109687  0.00049988216  0.0004450297
## Guanlong.sd        -0.006283668177  0.00128648648  0.00007883498 -0.0003779101
## Haplocheirus.sd    -0.000477292164  0.00154023577  0.00118904726  0.0003429699
## Linhenykus.sd       0.002054743403  0.00093954521  0.00185579528 -0.0010112711
## Manis.sd           -0.000286939551  0.00177228733 -0.00175680491  0.0009659593
## Mononykus.sd        0.002359968893 -0.00162736545  0.00253804874  0.0012377750
## Nothronychus.sd    -0.001149579183 -0.00050853502 -0.00117796382  0.0009966163
## Puma.sd             0.002766889862 -0.00131702939  0.00480421050 -0.0008354926
## Shishugounykus.sd   0.001732435276 -0.00148789966 -0.00088244390  0.0023254153
## Tamandua.sd        -0.000803385900  0.00038876758 -0.00026489026  0.0017089169
## Therizinosaurus.sd  0.000006641672 -0.00016526925  0.00012363884  0.0001291019
## Tugulusaurus.sd    -0.007583747669  0.00594150584  0.00033962864  0.0010004554
## Allosaurus.hp      -0.002342752852 -0.00027323849  0.00382502839 -0.0019013375
## Alxasaurus.hp       0.001035268646 -0.00071247734 -0.00066382204 -0.0013761703
## Aorun.hp            0.001500421560 -0.00098822420 -0.00320698074  0.0003537375
## Bannykus.hp        -0.003821996929 -0.00246020421  0.00434547999 -0.0009618883
## Beipiaosaurus.hp   -0.001791880008  0.00150310870 -0.00235585010  0.0012657692
## Eremotherium.hp     0.001120696668  0.00214381867 -0.00303043092  0.0030128067
## Erliansaurus.hp    -0.010017609411  0.00090106737  0.00157689723  0.0016113834
## Falcarius.hp       -0.000650316214  0.00208261424 -0.00248087977 -0.0036354831
## Guanlong.hp         0.000697058636  0.00721608352  0.00349576556  0.0058231533
## Haplocheirus.hp    -0.001342652927 -0.00025577388  0.00021936914 -0.0004796775
## Linhenykus.hp       0.005795757953  0.00780352388 -0.00144769894 -0.0034822539
## Manis.hp           -0.005017118129 -0.00734601578 -0.00622795436  0.0022919451
## Mononykus.hp       -0.007494564964  0.00078312715 -0.00394812654 -0.0022108982
## Nothronychus.hp    -0.000715906908 -0.00042022189  0.00197642845 -0.0012494929
## Puma.hp            -0.004321931344 -0.00274611636  0.00053592719 -0.0030747956
## Shishugounykus.hp  -0.005858606477  0.00024411134  0.00223023977  0.0030861608
## Tamandua.hp         0.000864368721 -0.00055769461 -0.00038451190 -0.0025276493
## Therizinosaurus.hp -0.000333909713 -0.00002247945 -0.00009035045 -0.0004494744
## Tugulusaurus.hp    -0.000738656642 -0.00086173760 -0.00306214181  0.0020363120
##                              PC49                  PC50
## Allosaurus.p       -0.00133621966  0.000000000135177577
## Alxasaurus.p        0.00161825630 -0.000000000290330524
## Aorun.p             0.00061382198 -0.000000000052280458
## Bannykus.p         -0.00066734813 -0.000000000121933255
## Beipiaosaurus.p    -0.00011606628  0.000000000079589182
## Eremotherium.p     -0.00014902085  0.000000000009106028
## Erliansaurus.p     -0.00028506548  0.000000000138747708
## Falcarius.p        -0.00040225684 -0.000000000056606012
## Guanlong.p          0.00015202812  0.000000000219472648
## Haplocheirus.p     -0.00112824541 -0.000000000044682896
## Linhenykus.p       -0.00069688425 -0.000000000077057249
## Manis.p             0.00144032322 -0.000000000043578391
## Mononykus.p         0.00046562666  0.000000000027085043
## Nothronychus.p      0.00022749284 -0.000000000012016700
## Puma.p             -0.00061033348  0.000000000151150925
## Shishugounykus.p    0.00065374229  0.000000000106496936
## Tamandua.p         -0.00010278479 -0.000000000045965010
## Therizinosaurus.p   0.00007532446  0.000000000028470337
## Tugulusaurus.p      0.00008334879  0.000000000080056475
## Allosaurus.sd      -0.00014221839  0.000000000101712125
## Alxasaurus.sd       0.00030066452  0.000000000040611486
## Aorun.sd           -0.00107090855  0.000000000037801373
## Bannykus.sd         0.00119147837  0.000000000016238871
## Beipiaosaurus.sd    0.00023993736 -0.000000000012170917
## Eremotherium.sd     0.00014717912 -0.000000000033314049
## Erliansaurus.sd     0.00016244468 -0.000000000056803950
## Falcarius.sd        0.00008474676  0.000000000040838166
## Guanlong.sd         0.00061778825  0.000000000011697546
## Haplocheirus.sd    -0.00056144361 -0.000000000073120230
## Linhenykus.sd      -0.00070237795  0.000000000063162142
## Manis.sd           -0.00041461365 -0.000000000202428282
## Mononykus.sd        0.00062024216  0.000000000078943671
## Nothronychus.sd     0.00062703422  0.000000000035276476
## Puma.sd             0.00125173490  0.000000000264385444
## Shishugounykus.sd  -0.00097919035 -0.000000000030978428
## Tamandua.sd        -0.00033197235  0.000000000045059671
## Therizinosaurus.sd -0.00025480157 -0.000000000039193981
## Tugulusaurus.sd     0.00016533523  0.000000000037573611
## Allosaurus.hp      -0.00150100151 -0.000000000132106492
## Alxasaurus.hp      -0.00185298466 -0.000000000201961559
## Aorun.hp           -0.00196346515 -0.000000000352212495
## Bannykus.hp         0.00118567826  0.000000000040122503
## Beipiaosaurus.hp    0.00086210907 -0.000000000129678365
## Eremotherium.hp     0.00102688794  0.000000000011948151
## Erliansaurus.hp    -0.00265045427  0.000000000302141381
## Falcarius.hp       -0.00009792542  0.000000000168963135
## Guanlong.hp        -0.00055803005  0.000000000114435197
## Haplocheirus.hp     0.00183303587 -0.000000000026489616
## Linhenykus.hp       0.00096491743 -0.000000000036669529
## Manis.hp            0.00055217252  0.000000000342683978
## Mononykus.hp       -0.00015017636  0.000000000005811199
## Nothronychus.hp     0.00086903897  0.000000000046270335
## Puma.hp            -0.00056571005 -0.000000000186259924
## Shishugounykus.hp   0.00100915078 -0.000000000497279343
## Tamandua.hp         0.00014647904 -0.000000000037566228
## Therizinosaurus.hp  0.00011083891 -0.000000000041418924
## Tugulusaurus.hp    -0.00000735995  0.000000000053075294
```

```
Coord.p<-ind.coord[1:19, 1:2]
Coord.s<-ind.coord[20:38, 1:2]
Coord.hp<-ind.coord[39:57, 1:2]
Coord<-ind.coord[1:57, 1:2]
Coord.p
```

```
##                           PC1        PC2
## Allosaurus.p      -3.34322735  1.5024415
## Alxasaurus.p      -4.25547189  3.8567721
## Aorun.p           -2.53687163  0.1436204
## Bannykus.p         0.95231302 -2.6743476
## Beipiaosaurus.p    3.81786982 -4.0764147
## Eremotherium.p    -0.86870155 -1.4597797
## Erliansaurus.p    -1.31118643 -2.7903685
## Falcarius.p       -0.05572564 -1.9047215
## Guanlong.p        -2.45530339  0.2594165
## Haplocheirus.p    -1.30671697 -1.4332262
## Linhenykus.p      -4.41812298  4.2021227
## Manis.p           -2.90212515 -0.3406726
## Mononykus.p       -3.56290532  1.4871996
## Nothronychus.p     1.75663273 -3.5751840
## Puma.p            -3.33456905  1.8648867
## Shishugounykus.p  -3.94190947  3.1544965
## Tamandua.p         3.45214163 -6.2439935
## Therizinosaurus.p 21.89583870  6.1195515
## Tugulusaurus.p    -3.13483590  2.0373422
```

```
Coord.s
```

```
##                           PC1        PC2
## Allosaurus.sd       2.4086445 -2.2084409
## Alxasaurus.sd      -0.9553253 -3.3378570
## Aorun.sd            2.4815255 -2.3874001
## Bannykus.sd         4.7883633 -3.8180150
## Beipiaosaurus.sd    4.8798236 -3.9494467
## Eremotherium.sd    -0.3050740 -1.1973785
## Erliansaurus.sd     0.1382769 -2.3141258
## Falcarius.sd        4.2261804 -3.9608883
## Guanlong.sd        -0.3737679  0.4249703
## Haplocheirus.sd     2.1163420 -1.4224292
## Linhenykus.sd      -4.4181251  4.2020874
## Manis.sd           -1.8587151 -2.9528135
## Mononykus.sd       -3.5629089  1.4872153
## Nothronychus.sd     0.3103920  0.9309279
## Puma.sd            -2.1327064  2.1343638
## Shishugounykus.sd  -1.1599824  0.7198907
## Tamandua.sd         9.7469621 -4.0015429
## Therizinosaurus.sd 21.2672994  7.3602389
## Tugulusaurus.sd    -2.1512056  1.8696094
```

```
Coord.hp
```

```
##                           PC1        PC2
## Allosaurus.hp      -2.0475045 -1.4825623
## Alxasaurus.hp      -3.7977112  2.9332823
## Aorun.hp           -2.7466871 -1.5153310
## Bannykus.hp        -2.0891062 -1.9456923
## Beipiaosaurus.hp   -0.9174517 -3.2212909
## Eremotherium.hp    -3.2354904  0.4168465
## Erliansaurus.hp    -3.4141241 -0.6467804
## Falcarius.hp       -2.6297254 -0.9299619
## Guanlong.hp        -4.1030430  2.5541163
## Haplocheirus.hp    -2.6635726 -1.5746950
## Linhenykus.hp      -4.1621863  2.5353954
## Manis.hp           -4.2384906  3.1852543
## Mononykus.hp       -4.0310255  2.4064425
## Nothronychus.hp     1.1542818 -2.6160904
## Puma.hp            -4.5050087  5.7497375
## Shishugounykus.hp  -4.5582694  2.9242360
## Tamandua.hp         2.1280086 -6.3318209
## Therizinosaurus.hp 20.7790085  3.4247211
## Tugulusaurus.hp    -4.8150244  6.4260867
```

```
Coord
```

```
##                            PC1        PC2
## Allosaurus.p       -3.34322735  1.5024415
## Alxasaurus.p       -4.25547189  3.8567721
## Aorun.p            -2.53687163  0.1436204
## Bannykus.p          0.95231302 -2.6743476
## Beipiaosaurus.p     3.81786982 -4.0764147
## Eremotherium.p     -0.86870155 -1.4597797
## Erliansaurus.p     -1.31118643 -2.7903685
## Falcarius.p        -0.05572564 -1.9047215
## Guanlong.p         -2.45530339  0.2594165
## Haplocheirus.p     -1.30671697 -1.4332262
## Linhenykus.p       -4.41812298  4.2021227
## Manis.p            -2.90212515 -0.3406726
## Mononykus.p        -3.56290532  1.4871996
## Nothronychus.p      1.75663273 -3.5751840
## Puma.p             -3.33456905  1.8648867
## Shishugounykus.p   -3.94190947  3.1544965
## Tamandua.p          3.45214163 -6.2439935
## Therizinosaurus.p  21.89583870  6.1195515
## Tugulusaurus.p     -3.13483590  2.0373422
## Allosaurus.sd       2.40864451 -2.2084409
## Alxasaurus.sd      -0.95532526 -3.3378570
## Aorun.sd            2.48152554 -2.3874001
## Bannykus.sd         4.78836331 -3.8180150
## Beipiaosaurus.sd    4.87982360 -3.9494467
## Eremotherium.sd    -0.30507397 -1.1973785
## Erliansaurus.sd     0.13827691 -2.3141258
## Falcarius.sd        4.22618045 -3.9608883
## Guanlong.sd        -0.37376791  0.4249703
## Haplocheirus.sd     2.11634198 -1.4224292
## Linhenykus.sd      -4.41812506  4.2020874
## Manis.sd           -1.85871510 -2.9528135
## Mononykus.sd       -3.56290888  1.4872153
## Nothronychus.sd     0.31039204  0.9309279
## Puma.sd            -2.13270643  2.1343638
## Shishugounykus.sd  -1.15998242  0.7198907
## Tamandua.sd         9.74696208 -4.0015429
## Therizinosaurus.sd 21.26729943  7.3602389
## Tugulusaurus.sd    -2.15120564  1.8696094
## Allosaurus.hp      -2.04750450 -1.4825623
## Alxasaurus.hp      -3.79771123  2.9332823
## Aorun.hp           -2.74668706 -1.5153310
## Bannykus.hp        -2.08910621 -1.9456923
## Beipiaosaurus.hp   -0.91745173 -3.2212909
## Eremotherium.hp    -3.23549043  0.4168465
## Erliansaurus.hp    -3.41412410 -0.6467804
## Falcarius.hp       -2.62972535 -0.9299619
## Guanlong.hp        -4.10304303  2.5541163
## Haplocheirus.hp    -2.66357265 -1.5746950
## Linhenykus.hp      -4.16218631  2.5353954
## Manis.hp           -4.23849061  3.1852543
## Mononykus.hp       -4.03102550  2.4064425
## Nothronychus.hp     1.15428179 -2.6160904
## Puma.hp            -4.50500872  5.7497375
## Shishugounykus.hp  -4.55826939  2.9242360
## Tamandua.hp         2.12800856 -6.3318209
## Therizinosaurus.hp 20.77900846  3.4247211
## Tugulusaurus.hp    -4.81502435  6.4260867
```

```
Coord<-cbind(Coord,row.names(Coord),Claw.group)
Coord<-data.frame(Coord)
Coord
```

```
##                                    PC1                PC2                 V3
## Allosaurus.p         -3.34322735187353   1.50244147382803       Allosaurus.p
## Alxasaurus.p           -4.255471889542   3.85677207688787       Alxasaurus.p
## Aorun.p              -2.53687163143769   0.14362043287757            Aorun.p
## Bannykus.p           0.952313016835642  -2.67434759313146         Bannykus.p
## Beipiaosaurus.p       3.81786981997023  -4.07641470020687    Beipiaosaurus.p
## Eremotherium.p      -0.868701548683492  -1.45977973617072     Eremotherium.p
## Erliansaurus.p       -1.31118643370482  -2.79036851806435     Erliansaurus.p
## Falcarius.p        -0.0557256402293965   -1.9047215336694        Falcarius.p
## Guanlong.p           -2.45530338937449  0.259416512016431         Guanlong.p
## Haplocheirus.p       -1.30671696825264  -1.43322620068637     Haplocheirus.p
## Linhenykus.p         -4.41812298074612   4.20212267224958       Linhenykus.p
## Manis.p               -2.9021251511579 -0.340672628586861            Manis.p
## Mononykus.p            -3.562905318611    1.4871995663131        Mononykus.p
## Nothronychus.p        1.75663273158633  -3.57518402087914     Nothronychus.p
## Puma.p                -3.3345690524545   1.86488667349052             Puma.p
## Shishugounykus.p      -3.9419094696946   3.15449645443408   Shishugounykus.p
## Tamandua.p            3.45214163320569  -6.24399354832337         Tamandua.p
## Therizinosaurus.p     21.8958386997326   6.11955147074652  Therizinosaurus.p
## Tugulusaurus.p       -3.13483589912855   2.03734216220988     Tugulusaurus.p
## Allosaurus.sd         2.40864451371362  -2.20844093055381      Allosaurus.sd
## Alxasaurus.sd       -0.955325259848304  -3.33785698412482      Alxasaurus.sd
## Aorun.sd               2.4815255391396  -2.38740014395244           Aorun.sd
## Bannykus.sd           4.78836330717256  -3.81801504226093        Bannykus.sd
## Beipiaosaurus.sd      4.87982360122715  -3.94944670390194   Beipiaosaurus.sd
## Eremotherium.sd     -0.305073969266404  -1.19737849355226    Eremotherium.sd
## Erliansaurus.sd      0.138276905508952  -2.31412581619549    Erliansaurus.sd
## Falcarius.sd          4.22618044567215  -3.96088826569217       Falcarius.sd
## Guanlong.sd         -0.373767907754908  0.424970332171403        Guanlong.sd
## Haplocheirus.sd       2.11634198315462   -1.4224292185737    Haplocheirus.sd
## Linhenykus.sd        -4.41812505884409   4.20208739538641      Linhenykus.sd
## Manis.sd             -1.85871509723941  -2.95281353580997           Manis.sd
## Mononykus.sd         -3.56290887529811   1.48721527875529       Mononykus.sd
## Nothronychus.sd      0.310392043143303  0.930927926314909    Nothronychus.sd
## Puma.sd              -2.13270643417702   2.13436383743563            Puma.sd
## Shishugounykus.sd    -1.15998241864241  0.719890696770926  Shishugounykus.sd
## Tamandua.sd           9.74696208477258  -4.00154290601293        Tamandua.sd
## Therizinosaurus.sd    21.2672994253161    7.3602388713676 Therizinosaurus.sd
## Tugulusaurus.sd      -2.15120564013809   1.86960940052722    Tugulusaurus.sd
## Allosaurus.hp        -2.04750450307139  -1.48256229294346      Allosaurus.hp
## Alxasaurus.hp        -3.79771122630939   2.93328226878909      Alxasaurus.hp
## Aorun.hp             -2.74668706422921  -1.51533101279831           Aorun.hp
## Bannykus.hp          -2.08910621167036  -1.94569234311952        Bannykus.hp
## Beipiaosaurus.hp    -0.917451731003115  -3.22129089496012   Beipiaosaurus.hp
## Eremotherium.hp      -3.23549043148814  0.416846487127476    Eremotherium.hp
## Erliansaurus.hp      -3.41412409537733 -0.646780357013774    Erliansaurus.hp
## Falcarius.hp         -2.62972535448004 -0.929961852106356       Falcarius.hp
## Guanlong.hp          -4.10304302514343   2.55411627131565        Guanlong.hp
## Haplocheirus.hp      -2.66357264842416  -1.57469501772789    Haplocheirus.hp
## Linhenykus.hp        -4.16218630963397   2.53539535845806      Linhenykus.hp
## Manis.hp             -4.23849061442556   3.18525425780466           Manis.hp
## Mononykus.hp         -4.03102550118825   2.40644250432042       Mononykus.hp
## Nothronychus.hp       1.15428179092534  -2.61609044287505    Nothronychus.hp
## Puma.hp              -4.50500872366101   5.74973749884311            Puma.hp
## Shishugounykus.hp    -4.55826938783683   2.92423596945238  Shishugounykus.hp
## Tamandua.hp           2.12800856452825  -6.33182094455303        Tamandua.hp
## Therizinosaurus.hp    20.7790084618885   3.42472114627147 Therizinosaurus.hp
## Tugulusaurus.hp      -4.81502435345154   6.42608668228123    Tugulusaurus.hp
##                         Claw.group
## Allosaurus.p            Allosaurus
## Alxasaurus.p            Alxasaurus
## Aorun.p                      Aorun
## Bannykus.p                Bannykus
## Beipiaosaurus.p      Beipiaosaurus
## Eremotherium.p        Eremotherium
## Erliansaurus.p        Erliansaurus
## Falcarius.p              Falcarius
## Guanlong.p                Guanlong
## Haplocheirus.p        Haplocheirus
## Linhenykus.p            Linhenykus
## Manis.p                      Manis
## Mononykus.p              Mononykus
## Nothronychus.p        Nothronychus
## Puma.p                        Puma
## Shishugounykus.p    Shishugounykus
## Tamandua.p                Tamandua
## Therizinosaurus.p  Therizinosaurus
## Tugulusaurus.p        Tugulusaurus
## Allosaurus.sd           Allosaurus
## Alxasaurus.sd           Alxasaurus
## Aorun.sd                     Aorun
## Bannykus.sd               Bannykus
## Beipiaosaurus.sd     Beipiaosaurus
## Eremotherium.sd       Eremotherium
## Erliansaurus.sd       Erliansaurus
## Falcarius.sd             Falcarius
## Guanlong.sd               Guanlong
## Haplocheirus.sd       Haplocheirus
## Linhenykus.sd           Linhenykus
## Manis.sd                     Manis
## Mononykus.sd             Mononykus
## Nothronychus.sd       Nothronychus
## Puma.sd                       Puma
## Shishugounykus.sd   Shishugounykus
## Tamandua.sd               Tamandua
## Therizinosaurus.sd Therizinosaurus
## Tugulusaurus.sd       Tugulusaurus
## Allosaurus.hp           Allosaurus
## Alxasaurus.hp           Alxasaurus
## Aorun.hp                     Aorun
## Bannykus.hp               Bannykus
## Beipiaosaurus.hp     Beipiaosaurus
## Eremotherium.hp       Eremotherium
## Erliansaurus.hp       Erliansaurus
## Falcarius.hp             Falcarius
## Guanlong.hp               Guanlong
## Haplocheirus.hp       Haplocheirus
## Linhenykus.hp           Linhenykus
## Manis.hp                     Manis
## Mononykus.hp             Mononykus
## Nothronychus.hp       Nothronychus
## Puma.hp                       Puma
## Shishugounykus.hp   Shishugounykus
## Tamandua.hp               Tamandua
## Therizinosaurus.hp Therizinosaurus
## Tugulusaurus.hp       Tugulusaurus
```

```
write.csv(Coord,'Coord.data.csv')
```

## 5.2 PC1 divergence in Piercing, Scratch-digging and Hook pull

```
Coord.pc1<-read.csv('Coord.data.csv', row.names = 1)
Coord.pc1
```

```
##                            PC1        PC2                 V3      Claw.group
## Allosaurus.p       -3.34322735  1.5024415       Allosaurus.p      Allosaurus
## Alxasaurus.p       -4.25547189  3.8567721       Alxasaurus.p      Alxasaurus
## Aorun.p            -2.53687163  0.1436204            Aorun.p           Aorun
## Bannykus.p          0.95231302 -2.6743476         Bannykus.p        Bannykus
## Beipiaosaurus.p     3.81786982 -4.0764147    Beipiaosaurus.p   Beipiaosaurus
## Eremotherium.p     -0.86870155 -1.4597797     Eremotherium.p    Eremotherium
## Erliansaurus.p     -1.31118643 -2.7903685     Erliansaurus.p    Erliansaurus
## Falcarius.p        -0.05572564 -1.9047215        Falcarius.p       Falcarius
## Guanlong.p         -2.45530339  0.2594165         Guanlong.p        Guanlong
## Haplocheirus.p     -1.30671697 -1.4332262     Haplocheirus.p    Haplocheirus
## Linhenykus.p       -4.41812298  4.2021227       Linhenykus.p      Linhenykus
## Manis.p            -2.90212515 -0.3406726            Manis.p           Manis
## Mononykus.p        -3.56290532  1.4871996        Mononykus.p       Mononykus
## Nothronychus.p      1.75663273 -3.5751840     Nothronychus.p    Nothronychus
## Puma.p             -3.33456905  1.8648867             Puma.p            Puma
## Shishugounykus.p   -3.94190947  3.1544965   Shishugounykus.p  Shishugounykus
## Tamandua.p          3.45214163 -6.2439935         Tamandua.p        Tamandua
## Therizinosaurus.p  21.89583870  6.1195515  Therizinosaurus.p Therizinosaurus
## Tugulusaurus.p     -3.13483590  2.0373422     Tugulusaurus.p    Tugulusaurus
## Allosaurus.sd       2.40864451 -2.2084409      Allosaurus.sd      Allosaurus
## Alxasaurus.sd      -0.95532526 -3.3378570      Alxasaurus.sd      Alxasaurus
## Aorun.sd            2.48152554 -2.3874001           Aorun.sd           Aorun
## Bannykus.sd         4.78836331 -3.8180150        Bannykus.sd        Bannykus
## Beipiaosaurus.sd    4.87982360 -3.9494467   Beipiaosaurus.sd   Beipiaosaurus
## Eremotherium.sd    -0.30507397 -1.1973785    Eremotherium.sd    Eremotherium
## Erliansaurus.sd     0.13827691 -2.3141258    Erliansaurus.sd    Erliansaurus
## Falcarius.sd        4.22618045 -3.9608883       Falcarius.sd       Falcarius
## Guanlong.sd        -0.37376791  0.4249703        Guanlong.sd        Guanlong
## Haplocheirus.sd     2.11634198 -1.4224292    Haplocheirus.sd    Haplocheirus
## Linhenykus.sd      -4.41812506  4.2020874      Linhenykus.sd      Linhenykus
## Manis.sd           -1.85871510 -2.9528135           Manis.sd           Manis
## Mononykus.sd       -3.56290888  1.4872153       Mononykus.sd       Mononykus
## Nothronychus.sd     0.31039204  0.9309279    Nothronychus.sd    Nothronychus
## Puma.sd            -2.13270643  2.1343638            Puma.sd            Puma
## Shishugounykus.sd  -1.15998242  0.7198907  Shishugounykus.sd  Shishugounykus
## Tamandua.sd         9.74696208 -4.0015429        Tamandua.sd        Tamandua
## Therizinosaurus.sd 21.26729943  7.3602389 Therizinosaurus.sd Therizinosaurus
## Tugulusaurus.sd    -2.15120564  1.8696094    Tugulusaurus.sd    Tugulusaurus
## Allosaurus.hp      -2.04750450 -1.4825623      Allosaurus.hp      Allosaurus
## Alxasaurus.hp      -3.79771123  2.9332823      Alxasaurus.hp      Alxasaurus
## Aorun.hp           -2.74668706 -1.5153310           Aorun.hp           Aorun
## Bannykus.hp        -2.08910621 -1.9456923        Bannykus.hp        Bannykus
## Beipiaosaurus.hp   -0.91745173 -3.2212909   Beipiaosaurus.hp   Beipiaosaurus
## Eremotherium.hp    -3.23549043  0.4168465    Eremotherium.hp    Eremotherium
## Erliansaurus.hp    -3.41412410 -0.6467804    Erliansaurus.hp    Erliansaurus
## Falcarius.hp       -2.62972535 -0.9299619       Falcarius.hp       Falcarius
## Guanlong.hp        -4.10304303  2.5541163        Guanlong.hp        Guanlong
## Haplocheirus.hp    -2.66357265 -1.5746950    Haplocheirus.hp    Haplocheirus
## Linhenykus.hp      -4.16218631  2.5353954      Linhenykus.hp      Linhenykus
## Manis.hp           -4.23849061  3.1852543           Manis.hp           Manis
## Mononykus.hp       -4.03102550  2.4064425       Mononykus.hp       Mononykus
## Nothronychus.hp     1.15428179 -2.6160904    Nothronychus.hp    Nothronychus
## Puma.hp            -4.50500872  5.7497375            Puma.hp            Puma
## Shishugounykus.hp  -4.55826939  2.9242360  Shishugounykus.hp  Shishugounykus
## Tamandua.hp         2.12800856 -6.3318209        Tamandua.hp        Tamandua
## Therizinosaurus.hp 20.77900846  3.4247211 Therizinosaurus.hp Therizinosaurus
## Tugulusaurus.hp    -4.81502435  6.4260867    Tugulusaurus.hp    Tugulusaurus
```

```
h<-ggplot(Coord.pc1, aes(x=V3,y=PC1))
h<-h+ geom_histogram(stat='identity',binwidth = 0.05,aes(fill=Claw.group))
h<-h+ coord_flip()                 
h
```

```
ggsave(file='PC1 divergence in P, S and HP.svg', plot=h,units="mm", width=300, height=300)
ggsave(file='PC1 divergence in P, S and HP.pdf', plot=h,units="mm", width=300, height=300)
```

## 5.3 PC2 divergence in Piercing, Scratch-digging and Hook pull

```
Coord.pc2<-read.csv('Coord.data.csv', row.names = 1)
Coord.pc2
```

```
##                            PC1        PC2                 V3      Claw.group
## Allosaurus.p       -3.34322735  1.5024415       Allosaurus.p      Allosaurus
## Alxasaurus.p       -4.25547189  3.8567721       Alxasaurus.p      Alxasaurus
## Aorun.p            -2.53687163  0.1436204            Aorun.p           Aorun
## Bannykus.p          0.95231302 -2.6743476         Bannykus.p        Bannykus
## Beipiaosaurus.p     3.81786982 -4.0764147    Beipiaosaurus.p   Beipiaosaurus
## Eremotherium.p     -0.86870155 -1.4597797     Eremotherium.p    Eremotherium
## Erliansaurus.p     -1.31118643 -2.7903685     Erliansaurus.p    Erliansaurus
## Falcarius.p        -0.05572564 -1.9047215        Falcarius.p       Falcarius
## Guanlong.p         -2.45530339  0.2594165         Guanlong.p        Guanlong
## Haplocheirus.p     -1.30671697 -1.4332262     Haplocheirus.p    Haplocheirus
## Linhenykus.p       -4.41812298  4.2021227       Linhenykus.p      Linhenykus
## Manis.p            -2.90212515 -0.3406726            Manis.p           Manis
## Mononykus.p        -3.56290532  1.4871996        Mononykus.p       Mononykus
## Nothronychus.p      1.75663273 -3.5751840     Nothronychus.p    Nothronychus
## Puma.p             -3.33456905  1.8648867             Puma.p            Puma
## Shishugounykus.p   -3.94190947  3.1544965   Shishugounykus.p  Shishugounykus
## Tamandua.p          3.45214163 -6.2439935         Tamandua.p        Tamandua
## Therizinosaurus.p  21.89583870  6.1195515  Therizinosaurus.p Therizinosaurus
## Tugulusaurus.p     -3.13483590  2.0373422     Tugulusaurus.p    Tugulusaurus
## Allosaurus.sd       2.40864451 -2.2084409      Allosaurus.sd      Allosaurus
## Alxasaurus.sd      -0.95532526 -3.3378570      Alxasaurus.sd      Alxasaurus
## Aorun.sd            2.48152554 -2.3874001           Aorun.sd           Aorun
## Bannykus.sd         4.78836331 -3.8180150        Bannykus.sd        Bannykus
## Beipiaosaurus.sd    4.87982360 -3.9494467   Beipiaosaurus.sd   Beipiaosaurus
## Eremotherium.sd    -0.30507397 -1.1973785    Eremotherium.sd    Eremotherium
## Erliansaurus.sd     0.13827691 -2.3141258    Erliansaurus.sd    Erliansaurus
## Falcarius.sd        4.22618045 -3.9608883       Falcarius.sd       Falcarius
## Guanlong.sd        -0.37376791  0.4249703        Guanlong.sd        Guanlong
## Haplocheirus.sd     2.11634198 -1.4224292    Haplocheirus.sd    Haplocheirus
## Linhenykus.sd      -4.41812506  4.2020874      Linhenykus.sd      Linhenykus
## Manis.sd           -1.85871510 -2.9528135           Manis.sd           Manis
## Mononykus.sd       -3.56290888  1.4872153       Mononykus.sd       Mononykus
## Nothronychus.sd     0.31039204  0.9309279    Nothronychus.sd    Nothronychus
## Puma.sd            -2.13270643  2.1343638            Puma.sd            Puma
## Shishugounykus.sd  -1.15998242  0.7198907  Shishugounykus.sd  Shishugounykus
## Tamandua.sd         9.74696208 -4.0015429        Tamandua.sd        Tamandua
## Therizinosaurus.sd 21.26729943  7.3602389 Therizinosaurus.sd Therizinosaurus
## Tugulusaurus.sd    -2.15120564  1.8696094    Tugulusaurus.sd    Tugulusaurus
## Allosaurus.hp      -2.04750450 -1.4825623      Allosaurus.hp      Allosaurus
## Alxasaurus.hp      -3.79771123  2.9332823      Alxasaurus.hp      Alxasaurus
## Aorun.hp           -2.74668706 -1.5153310           Aorun.hp           Aorun
## Bannykus.hp        -2.08910621 -1.9456923        Bannykus.hp        Bannykus
## Beipiaosaurus.hp   -0.91745173 -3.2212909   Beipiaosaurus.hp   Beipiaosaurus
## Eremotherium.hp    -3.23549043  0.4168465    Eremotherium.hp    Eremotherium
## Erliansaurus.hp    -3.41412410 -0.6467804    Erliansaurus.hp    Erliansaurus
## Falcarius.hp       -2.62972535 -0.9299619       Falcarius.hp       Falcarius
## Guanlong.hp        -4.10304303  2.5541163        Guanlong.hp        Guanlong
## Haplocheirus.hp    -2.66357265 -1.5746950    Haplocheirus.hp    Haplocheirus
## Linhenykus.hp      -4.16218631  2.5353954      Linhenykus.hp      Linhenykus
## Manis.hp           -4.23849061  3.1852543           Manis.hp           Manis
## Mononykus.hp       -4.03102550  2.4064425       Mononykus.hp       Mononykus
## Nothronychus.hp     1.15428179 -2.6160904    Nothronychus.hp    Nothronychus
## Puma.hp            -4.50500872  5.7497375            Puma.hp            Puma
## Shishugounykus.hp  -4.55826939  2.9242360  Shishugounykus.hp  Shishugounykus
## Tamandua.hp         2.12800856 -6.3318209        Tamandua.hp        Tamandua
## Therizinosaurus.hp 20.77900846  3.4247211 Therizinosaurus.hp Therizinosaurus
## Tugulusaurus.hp    -4.81502435  6.4260867    Tugulusaurus.hp    Tugulusaurus
```

```
h<-ggplot(Coord.pc2, aes(x=V3,y=PC2))
h<-h+ geom_histogram(stat='identity',binwidth = 0.05,aes(fill=Claw.group))
h<-h+ coord_flip()                 
h
```

```
ggsave(file='PC2 divergence in P, S and HP.svg', plot=h,units="mm", width=300, height=300)
ggsave(file='PC2 divergence in P, S and HP.pdf', plot=h,units="mm", width=300, height=300)
```

## 5.4 divergence from Scratch-digging to Piercing

```
##divergence from Scratch-digging to Piercing.##
Coord.p
```

```
##                           PC1        PC2
## Allosaurus.p      -3.34322735  1.5024415
## Alxasaurus.p      -4.25547189  3.8567721
## Aorun.p           -2.53687163  0.1436204
## Bannykus.p         0.95231302 -2.6743476
## Beipiaosaurus.p    3.81786982 -4.0764147
## Eremotherium.p    -0.86870155 -1.4597797
## Erliansaurus.p    -1.31118643 -2.7903685
## Falcarius.p       -0.05572564 -1.9047215
## Guanlong.p        -2.45530339  0.2594165
## Haplocheirus.p    -1.30671697 -1.4332262
## Linhenykus.p      -4.41812298  4.2021227
## Manis.p           -2.90212515 -0.3406726
## Mononykus.p       -3.56290532  1.4871996
## Nothronychus.p     1.75663273 -3.5751840
## Puma.p            -3.33456905  1.8648867
## Shishugounykus.p  -3.94190947  3.1544965
## Tamandua.p         3.45214163 -6.2439935
## Therizinosaurus.p 21.89583870  6.1195515
## Tugulusaurus.p    -3.13483590  2.0373422
```

```
Coord.s
```

```
##                           PC1        PC2
## Allosaurus.sd       2.4086445 -2.2084409
## Alxasaurus.sd      -0.9553253 -3.3378570
## Aorun.sd            2.4815255 -2.3874001
## Bannykus.sd         4.7883633 -3.8180150
## Beipiaosaurus.sd    4.8798236 -3.9494467
## Eremotherium.sd    -0.3050740 -1.1973785
## Erliansaurus.sd     0.1382769 -2.3141258
## Falcarius.sd        4.2261804 -3.9608883
## Guanlong.sd        -0.3737679  0.4249703
## Haplocheirus.sd     2.1163420 -1.4224292
## Linhenykus.sd      -4.4181251  4.2020874
## Manis.sd           -1.8587151 -2.9528135
## Mononykus.sd       -3.5629089  1.4872153
## Nothronychus.sd     0.3103920  0.9309279
## Puma.sd            -2.1327064  2.1343638
## Shishugounykus.sd  -1.1599824  0.7198907
## Tamandua.sd         9.7469621 -4.0015429
## Therizinosaurus.sd 21.2672994  7.3602389
## Tugulusaurus.sd    -2.1512056  1.8696094
```

```
Coord.hp
```

```
##                           PC1        PC2
## Allosaurus.hp      -2.0475045 -1.4825623
## Alxasaurus.hp      -3.7977112  2.9332823
## Aorun.hp           -2.7466871 -1.5153310
## Bannykus.hp        -2.0891062 -1.9456923
## Beipiaosaurus.hp   -0.9174517 -3.2212909
## Eremotherium.hp    -3.2354904  0.4168465
## Erliansaurus.hp    -3.4141241 -0.6467804
## Falcarius.hp       -2.6297254 -0.9299619
## Guanlong.hp        -4.1030430  2.5541163
## Haplocheirus.hp    -2.6635726 -1.5746950
## Linhenykus.hp      -4.1621863  2.5353954
## Manis.hp           -4.2384906  3.1852543
## Mononykus.hp       -4.0310255  2.4064425
## Nothronychus.hp     1.1542818 -2.6160904
## Puma.hp            -4.5050087  5.7497375
## Shishugounykus.hp  -4.5582694  2.9242360
## Tamandua.hp         2.1280086 -6.3318209
## Therizinosaurus.hp 20.7790085  3.4247211
## Tugulusaurus.hp    -4.8150244  6.4260867
```

```
Coord.s.p<-cbind(Coord.s,Coord.p)
Coord.s.p<-cbind(Coord.s.p,Coord.s.p[,1]-Coord.s.p[,3],Coord.s.p[,2]-Coord.s.p[,4])
Coord.s.p
```

```
##                           PC1        PC2         PC1        PC2                
## Allosaurus.sd       2.4086445 -2.2084409 -3.34322735  1.5024415  5.751871865587
## Alxasaurus.sd      -0.9553253 -3.3378570 -4.25547189  3.8567721  3.300146629694
## Aorun.sd            2.4815255 -2.3874001 -2.53687163  0.1436204  5.018397170577
## Bannykus.sd         4.7883633 -3.8180150  0.95231302 -2.6743476  3.836050290337
## Beipiaosaurus.sd    4.8798236 -3.9494467  3.81786982 -4.0764147  1.061953781257
## Eremotherium.sd    -0.3050740 -1.1973785 -0.86870155 -1.4597797  0.563627579417
## Erliansaurus.sd     0.1382769 -2.3141258 -1.31118643 -2.7903685  1.449463339214
## Falcarius.sd        4.2261804 -3.9608883 -0.05572564 -1.9047215  4.281906085902
## Guanlong.sd        -0.3737679  0.4249703 -2.45530339  0.2594165  2.081535481620
## Haplocheirus.sd     2.1163420 -1.4224292 -1.30671697 -1.4332262  3.423058951407
## Linhenykus.sd      -4.4181251  4.2020874 -4.41812298  4.2021227 -0.000002078098
## Manis.sd           -1.8587151 -2.9528135 -2.90212515 -0.3406726  1.043410053918
## Mononykus.sd       -3.5629089  1.4872153 -3.56290532  1.4871996 -0.000003556687
## Nothronychus.sd     0.3103920  0.9309279  1.75663273 -3.5751840 -1.446240688443
## Puma.sd            -2.1327064  2.1343638 -3.33456905  1.8648867  1.201862618277
## Shishugounykus.sd  -1.1599824  0.7198907 -3.94190947  3.1544965  2.781927051052
## Tamandua.sd         9.7469621 -4.0015429  3.45214163 -6.2439935  6.294820451567
## Therizinosaurus.sd 21.2672994  7.3602389 21.89583870  6.1195515 -0.628539274416
## Tugulusaurus.sd    -2.1512056  1.8696094 -3.13483590  2.0373422  0.983630258990
##                                  
## Allosaurus.sd      -3.71088240438
## Alxasaurus.sd      -7.19462906101
## Aorun.sd           -2.53102057683
## Bannykus.sd        -1.14366744913
## Beipiaosaurus.sd    0.12696799630
## Eremotherium.sd     0.26240124262
## Erliansaurus.sd     0.47624270187
## Falcarius.sd       -2.05616673202
## Guanlong.sd         0.16555382015
## Haplocheirus.sd     0.01079698211
## Linhenykus.sd      -0.00003527686
## Manis.sd           -2.61214090722
## Mononykus.sd        0.00001571244
## Nothronychus.sd     4.50611194719
## Puma.sd             0.26947716395
## Shishugounykus.sd  -2.43460575766
## Tamandua.sd         2.24245064231
## Therizinosaurus.sd  1.24068740062
## Tugulusaurus.sd    -0.16773276168
```

```
colnames(Coord.s.p)<-c('PC1.s','PC2.s','PC1.p','PC2.p','PC1.s-PC1.p','PC2.s-PC2.p')
rownames(Coord.s.p)<-c(Claw.group[1:19])
Coord.s.p
```

```
##                      PC1.s      PC2.s       PC1.p      PC2.p     PC1.s-PC1.p
## Allosaurus       2.4086445 -2.2084409 -3.34322735  1.5024415  5.751871865587
## Alxasaurus      -0.9553253 -3.3378570 -4.25547189  3.8567721  3.300146629694
## Aorun            2.4815255 -2.3874001 -2.53687163  0.1436204  5.018397170577
## Bannykus         4.7883633 -3.8180150  0.95231302 -2.6743476  3.836050290337
## Beipiaosaurus    4.8798236 -3.9494467  3.81786982 -4.0764147  1.061953781257
## Eremotherium    -0.3050740 -1.1973785 -0.86870155 -1.4597797  0.563627579417
## Erliansaurus     0.1382769 -2.3141258 -1.31118643 -2.7903685  1.449463339214
## Falcarius        4.2261804 -3.9608883 -0.05572564 -1.9047215  4.281906085902
## Guanlong        -0.3737679  0.4249703 -2.45530339  0.2594165  2.081535481620
## Haplocheirus     2.1163420 -1.4224292 -1.30671697 -1.4332262  3.423058951407
## Linhenykus      -4.4181251  4.2020874 -4.41812298  4.2021227 -0.000002078098
## Manis           -1.8587151 -2.9528135 -2.90212515 -0.3406726  1.043410053918
## Mononykus       -3.5629089  1.4872153 -3.56290532  1.4871996 -0.000003556687
## Nothronychus     0.3103920  0.9309279  1.75663273 -3.5751840 -1.446240688443
## Puma            -2.1327064  2.1343638 -3.33456905  1.8648867  1.201862618277
## Shishugounykus  -1.1599824  0.7198907 -3.94190947  3.1544965  2.781927051052
## Tamandua         9.7469621 -4.0015429  3.45214163 -6.2439935  6.294820451567
## Therizinosaurus 21.2672994  7.3602389 21.89583870  6.1195515 -0.628539274416
## Tugulusaurus    -2.1512056  1.8696094 -3.13483590  2.0373422  0.983630258990
##                    PC2.s-PC2.p
## Allosaurus      -3.71088240438
## Alxasaurus      -7.19462906101
## Aorun           -2.53102057683
## Bannykus        -1.14366744913
## Beipiaosaurus    0.12696799630
## Eremotherium     0.26240124262
## Erliansaurus     0.47624270187
## Falcarius       -2.05616673202
## Guanlong         0.16555382015
## Haplocheirus     0.01079698211
## Linhenykus      -0.00003527686
## Manis           -2.61214090722
## Mononykus        0.00001571244
## Nothronychus     4.50611194719
## Puma             0.26947716395
## Shishugounykus  -2.43460575766
## Tamandua         2.24245064231
## Therizinosaurus  1.24068740062
## Tugulusaurus    -0.16773276168
```

```
Coord.s.p<-data.frame(Coord.s.p)
Coord.s.p
```

```
##                      PC1.s      PC2.s       PC1.p      PC2.p     PC1.s.PC1.p
## Allosaurus       2.4086445 -2.2084409 -3.34322735  1.5024415  5.751871865587
## Alxasaurus      -0.9553253 -3.3378570 -4.25547189  3.8567721  3.300146629694
## Aorun            2.4815255 -2.3874001 -2.53687163  0.1436204  5.018397170577
## Bannykus         4.7883633 -3.8180150  0.95231302 -2.6743476  3.836050290337
## Beipiaosaurus    4.8798236 -3.9494467  3.81786982 -4.0764147  1.061953781257
## Eremotherium    -0.3050740 -1.1973785 -0.86870155 -1.4597797  0.563627579417
## Erliansaurus     0.1382769 -2.3141258 -1.31118643 -2.7903685  1.449463339214
## Falcarius        4.2261804 -3.9608883 -0.05572564 -1.9047215  4.281906085902
## Guanlong        -0.3737679  0.4249703 -2.45530339  0.2594165  2.081535481620
## Haplocheirus     2.1163420 -1.4224292 -1.30671697 -1.4332262  3.423058951407
## Linhenykus      -4.4181251  4.2020874 -4.41812298  4.2021227 -0.000002078098
## Manis           -1.8587151 -2.9528135 -2.90212515 -0.3406726  1.043410053918
## Mononykus       -3.5629089  1.4872153 -3.56290532  1.4871996 -0.000003556687
## Nothronychus     0.3103920  0.9309279  1.75663273 -3.5751840 -1.446240688443
## Puma            -2.1327064  2.1343638 -3.33456905  1.8648867  1.201862618277
## Shishugounykus  -1.1599824  0.7198907 -3.94190947  3.1544965  2.781927051052
## Tamandua         9.7469621 -4.0015429  3.45214163 -6.2439935  6.294820451567
## Therizinosaurus 21.2672994  7.3602389 21.89583870  6.1195515 -0.628539274416
## Tugulusaurus    -2.1512056  1.8696094 -3.13483590  2.0373422  0.983630258990
##                    PC2.s.PC2.p
## Allosaurus      -3.71088240438
## Alxasaurus      -7.19462906101
## Aorun           -2.53102057683
## Bannykus        -1.14366744913
## Beipiaosaurus    0.12696799630
## Eremotherium     0.26240124262
## Erliansaurus     0.47624270187
## Falcarius       -2.05616673202
## Guanlong         0.16555382015
## Haplocheirus     0.01079698211
## Linhenykus      -0.00003527686
## Manis           -2.61214090722
## Mononykus        0.00001571244
## Nothronychus     4.50611194719
## Puma             0.26947716395
## Shishugounykus  -2.43460575766
## Tamandua         2.24245064231
## Therizinosaurus  1.24068740062
## Tugulusaurus    -0.16773276168
```

```
#PC1 divergence from Scratch-digging to Piercing.#
h1<-ggplot(Coord.s.p, aes(x=row.names(Coord.s.p),y=PC1.s-PC1.p))
h1<-h1+ geom_histogram(stat='identity',binwidth = 0.05)
h1<-h1+ coord_flip()                 
h1
```

```
ggsave(file='PC1 divergence from Scratch to Piercing.svg', plot=h1,units="mm", width=300, height=300)
ggsave(file='PC1 divergence from Scratch to Piercing.pdf', plot=h1,units="mm", width=300, height=300)

#PC2 divergence from Scratch-digging to Piercing.#
h2<-ggplot(Coord.s.p, aes(x=row.names(Coord.s.p),y=PC2.s-PC2.p))
h2<-h2+ geom_histogram(stat='identity',binwidth = 0.05)
h2<-h2+ coord_flip()                 
h2
```

```
ggsave(file='PC2 divergence from Scratch to Piercing.svg', plot=h2,units="mm", width=300, height=300)
ggsave(file='PC2 divergence from Scratch to Piercing.pdf', plot=h2,units="mm", width=300, height=300)
```

## 5.5 divergence from HookandPull to Piercing.

```
###divergence from HookandPull to Piercing.###

Coord.hp.p<-cbind(Coord.hp,Coord.p)
Coord.hp.p<-cbind(Coord.hp.p,Coord.hp.p[,1]-Coord.hp.p[,3],Coord.hp.p[,2]-Coord.hp.p[,4])
Coord.hp.p
```

```
##                           PC1        PC2         PC1        PC2           
## Allosaurus.hp      -2.0475045 -1.4825623 -3.34322735  1.5024415  1.2957228
## Alxasaurus.hp      -3.7977112  2.9332823 -4.25547189  3.8567721  0.4577607
## Aorun.hp           -2.7466871 -1.5153310 -2.53687163  0.1436204 -0.2098154
## Bannykus.hp        -2.0891062 -1.9456923  0.95231302 -2.6743476 -3.0414192
## Beipiaosaurus.hp   -0.9174517 -3.2212909  3.81786982 -4.0764147 -4.7353216
## Eremotherium.hp    -3.2354904  0.4168465 -0.86870155 -1.4597797 -2.3667889
## Erliansaurus.hp    -3.4141241 -0.6467804 -1.31118643 -2.7903685 -2.1029377
## Falcarius.hp       -2.6297254 -0.9299619 -0.05572564 -1.9047215 -2.5739997
## Guanlong.hp        -4.1030430  2.5541163 -2.45530339  0.2594165 -1.6477396
## Haplocheirus.hp    -2.6635726 -1.5746950 -1.30671697 -1.4332262 -1.3568557
## Linhenykus.hp      -4.1621863  2.5353954 -4.41812298  4.2021227  0.2559367
## Manis.hp           -4.2384906  3.1852543 -2.90212515 -0.3406726 -1.3363655
## Mononykus.hp       -4.0310255  2.4064425 -3.56290532  1.4871996 -0.4681202
## Nothronychus.hp     1.1542818 -2.6160904  1.75663273 -3.5751840 -0.6023509
## Puma.hp            -4.5050087  5.7497375 -3.33456905  1.8648867 -1.1704397
## Shishugounykus.hp  -4.5582694  2.9242360 -3.94190947  3.1544965 -0.6163599
## Tamandua.hp         2.1280086 -6.3318209  3.45214163 -6.2439935 -1.3241331
## Therizinosaurus.hp 20.7790085  3.4247211 21.89583870  6.1195515 -1.1168302
## Tugulusaurus.hp    -4.8150244  6.4260867 -3.13483590  2.0373422 -1.6801885
##                              
## Allosaurus.hp      -2.9850038
## Alxasaurus.hp      -0.9234898
## Aorun.hp           -1.6589514
## Bannykus.hp         0.7286553
## Beipiaosaurus.hp    0.8551238
## Eremotherium.hp     1.8766262
## Erliansaurus.hp     2.1435882
## Falcarius.hp        0.9747597
## Guanlong.hp         2.2946998
## Haplocheirus.hp    -0.1414688
## Linhenykus.hp      -1.6667273
## Manis.hp            3.5259269
## Mononykus.hp        0.9192429
## Nothronychus.hp     0.9590936
## Puma.hp             3.8848508
## Shishugounykus.hp  -0.2302605
## Tamandua.hp        -0.0878274
## Therizinosaurus.hp -2.6948303
## Tugulusaurus.hp     4.3887445
```

```
colnames(Coord.hp.p)<-c('PC1.hp','PC2.hp','PC1.p','PC2.p','PC1.hp-PC1.p','PC2.hp-PC2.p')
rownames(Coord.hp.p)<-c(Claw.group[1:19])
Coord.hp.p
```

```
##                     PC1.hp     PC2.hp       PC1.p      PC2.p PC1.hp-PC1.p
## Allosaurus      -2.0475045 -1.4825623 -3.34322735  1.5024415    1.2957228
## Alxasaurus      -3.7977112  2.9332823 -4.25547189  3.8567721    0.4577607
## Aorun           -2.7466871 -1.5153310 -2.53687163  0.1436204   -0.2098154
## Bannykus        -2.0891062 -1.9456923  0.95231302 -2.6743476   -3.0414192
## Beipiaosaurus   -0.9174517 -3.2212909  3.81786982 -4.0764147   -4.7353216
## Eremotherium    -3.2354904  0.4168465 -0.86870155 -1.4597797   -2.3667889
## Erliansaurus    -3.4141241 -0.6467804 -1.31118643 -2.7903685   -2.1029377
## Falcarius       -2.6297254 -0.9299619 -0.05572564 -1.9047215   -2.5739997
## Guanlong        -4.1030430  2.5541163 -2.45530339  0.2594165   -1.6477396
## Haplocheirus    -2.6635726 -1.5746950 -1.30671697 -1.4332262   -1.3568557
## Linhenykus      -4.1621863  2.5353954 -4.41812298  4.2021227    0.2559367
## Manis           -4.2384906  3.1852543 -2.90212515 -0.3406726   -1.3363655
## Mononykus       -4.0310255  2.4064425 -3.56290532  1.4871996   -0.4681202
## Nothronychus     1.1542818 -2.6160904  1.75663273 -3.5751840   -0.6023509
## Puma            -4.5050087  5.7497375 -3.33456905  1.8648867   -1.1704397
## Shishugounykus  -4.5582694  2.9242360 -3.94190947  3.1544965   -0.6163599
## Tamandua         2.1280086 -6.3318209  3.45214163 -6.2439935   -1.3241331
## Therizinosaurus 20.7790085  3.4247211 21.89583870  6.1195515   -1.1168302
## Tugulusaurus    -4.8150244  6.4260867 -3.13483590  2.0373422   -1.6801885
##                 PC2.hp-PC2.p
## Allosaurus        -2.9850038
## Alxasaurus        -0.9234898
## Aorun             -1.6589514
## Bannykus           0.7286553
## Beipiaosaurus      0.8551238
## Eremotherium       1.8766262
## Erliansaurus       2.1435882
## Falcarius          0.9747597
## Guanlong           2.2946998
## Haplocheirus      -0.1414688
## Linhenykus        -1.6667273
## Manis              3.5259269
## Mononykus          0.9192429
## Nothronychus       0.9590936
## Puma               3.8848508
## Shishugounykus    -0.2302605
## Tamandua          -0.0878274
## Therizinosaurus   -2.6948303
## Tugulusaurus       4.3887445
```

```
Coord.hp.p<-data.frame(Coord.hp.p)

#PC1 divergence from HookandPull to Piercing.#
h3<-ggplot(Coord.hp.p, aes(x=row.names(Coord.hp.p),y=PC1.hp-PC1.p))
h3<-h3+ geom_histogram(stat='identity',binwidth = 0.05)
h3<-h3+ coord_flip()                 
h3
```

```
ggsave(file='PC1 divergence from HookandPull to Piercing.svg', plot=h3,units="mm", width=300, height=300)
ggsave(file='PC1 divergence from HookandPull to Piercing.pdf', plot=h3,units="mm", width=300, height=300)

#PC2 divergence from HookandPull to Piercing.#
h4<-ggplot(Coord.hp.p, aes(x=row.names(Coord.hp.p),y=PC2.hp-PC2.p))
h4<-h4+ geom_histogram(stat='identity',binwidth = 0.05)
h4<-h4+ coord_flip()                 
h4
```

```
ggsave(file='PC2 divergence from HookandPull to Piercing.svg', plot=h4,units="mm", width=300, height=300)
ggsave(file='PC2 divergence from HookandPull to Piercing.pdf', plot=h4,units="mm", width=300, height=300)
```

## 5.6 combined figure

```
##combine figure
myplot<-grid.arrange(h1,h2,h3,h4, ncol=2, nrow=2)
```

```
myplot
```

```
## TableGrob (2 x 2) "arrange": 4 grobs
##   z     cells    name           grob
## 1 1 (1-1,1-1) arrange gtable[layout]
## 2 2 (1-1,2-2) arrange gtable[layout]
## 3 3 (2-2,1-1) arrange gtable[layout]
## 4 4 (2-2,2-2) arrange gtable[layout]
```

```
ggsave(file='PC1PC2 divergence combine figure.svg', plot=myplot,units="mm", width=400, height=400)
ggsave(file='PC1PC2 divergence combine figure.pdf', plot=myplot,units="mm", width=400, height=400)
```

## 5.6 combine figure Ridgelineplot by Genera

```
##combine figure Ridgelineplot by Genera##

RidgelineplotPC1<-ggplot(Coord.pc1, aes(x = PC1, y = Claw.group, fill = Claw.group)) +
                   geom_density_ridges(alpha=0.6, stat="binline", bins=30) +
                      theme_ridges() + 
                     theme(axis.text.y = element_text( face = "italic"))+
                   theme(legend.position = "none")+
                   xlab('Coordinates on FPC1')+
                   ylab('Genera')
RidgelineplotPC1
```

```
RidgelineplotPC2<-ggplot(Coord.pc1, aes(x = PC2, y = Claw.group, fill = Claw.group)) +
  geom_density_ridges(alpha=0.6, stat="binline", bins=30) +
  theme_ridges() + 
  theme(axis.text.y = element_text( face = "italic"))+
  theme(legend.position = "none")+
  xlab('Coordinates on FPC2')+
  ylab('Genera')
RidgelineplotPC2
```

```
##combine figure Ridgelineplot by clade##
Group<-read.csv('Group data.csv',header = F)
Coord<-cbind(Coord.pc1,Group$V4)
colnames(Coord)<-c("PC1", "PC2", "Function",  "Genera", "Clade" )
Coord
```

```
##                            PC1        PC2           Function          Genera
## Allosaurus.p       -3.34322735  1.5024415       Allosaurus.p      Allosaurus
## Alxasaurus.p       -4.25547189  3.8567721       Alxasaurus.p      Alxasaurus
## Aorun.p            -2.53687163  0.1436204            Aorun.p           Aorun
## Bannykus.p          0.95231302 -2.6743476         Bannykus.p        Bannykus
## Beipiaosaurus.p     3.81786982 -4.0764147    Beipiaosaurus.p   Beipiaosaurus
## Eremotherium.p     -0.86870155 -1.4597797     Eremotherium.p    Eremotherium
## Erliansaurus.p     -1.31118643 -2.7903685     Erliansaurus.p    Erliansaurus
## Falcarius.p        -0.05572564 -1.9047215        Falcarius.p       Falcarius
## Guanlong.p         -2.45530339  0.2594165         Guanlong.p        Guanlong
## Haplocheirus.p     -1.30671697 -1.4332262     Haplocheirus.p    Haplocheirus
## Linhenykus.p       -4.41812298  4.2021227       Linhenykus.p      Linhenykus
## Manis.p            -2.90212515 -0.3406726            Manis.p           Manis
## Mononykus.p        -3.56290532  1.4871996        Mononykus.p       Mononykus
## Nothronychus.p      1.75663273 -3.5751840     Nothronychus.p    Nothronychus
## Puma.p             -3.33456905  1.8648867             Puma.p            Puma
## Shishugounykus.p   -3.94190947  3.1544965   Shishugounykus.p  Shishugounykus
## Tamandua.p          3.45214163 -6.2439935         Tamandua.p        Tamandua
## Therizinosaurus.p  21.89583870  6.1195515  Therizinosaurus.p Therizinosaurus
## Tugulusaurus.p     -3.13483590  2.0373422     Tugulusaurus.p    Tugulusaurus
## Allosaurus.sd       2.40864451 -2.2084409      Allosaurus.sd      Allosaurus
## Alxasaurus.sd      -0.95532526 -3.3378570      Alxasaurus.sd      Alxasaurus
## Aorun.sd            2.48152554 -2.3874001           Aorun.sd           Aorun
## Bannykus.sd         4.78836331 -3.8180150        Bannykus.sd        Bannykus
## Beipiaosaurus.sd    4.87982360 -3.9494467   Beipiaosaurus.sd   Beipiaosaurus
## Eremotherium.sd    -0.30507397 -1.1973785    Eremotherium.sd    Eremotherium
## Erliansaurus.sd     0.13827691 -2.3141258    Erliansaurus.sd    Erliansaurus
## Falcarius.sd        4.22618045 -3.9608883       Falcarius.sd       Falcarius
## Guanlong.sd        -0.37376791  0.4249703        Guanlong.sd        Guanlong
## Haplocheirus.sd     2.11634198 -1.4224292    Haplocheirus.sd    Haplocheirus
## Linhenykus.sd      -4.41812506  4.2020874      Linhenykus.sd      Linhenykus
## Manis.sd           -1.85871510 -2.9528135           Manis.sd           Manis
## Mononykus.sd       -3.56290888  1.4872153       Mononykus.sd       Mononykus
## Nothronychus.sd     0.31039204  0.9309279    Nothronychus.sd    Nothronychus
## Puma.sd            -2.13270643  2.1343638            Puma.sd            Puma
## Shishugounykus.sd  -1.15998242  0.7198907  Shishugounykus.sd  Shishugounykus
## Tamandua.sd         9.74696208 -4.0015429        Tamandua.sd        Tamandua
## Therizinosaurus.sd 21.26729943  7.3602389 Therizinosaurus.sd Therizinosaurus
## Tugulusaurus.sd    -2.15120564  1.8696094    Tugulusaurus.sd    Tugulusaurus
## Allosaurus.hp      -2.04750450 -1.4825623      Allosaurus.hp      Allosaurus
## Alxasaurus.hp      -3.79771123  2.9332823      Alxasaurus.hp      Alxasaurus
## Aorun.hp           -2.74668706 -1.5153310           Aorun.hp           Aorun
## Bannykus.hp        -2.08910621 -1.9456923        Bannykus.hp        Bannykus
## Beipiaosaurus.hp   -0.91745173 -3.2212909   Beipiaosaurus.hp   Beipiaosaurus
## Eremotherium.hp    -3.23549043  0.4168465    Eremotherium.hp    Eremotherium
## Erliansaurus.hp    -3.41412410 -0.6467804    Erliansaurus.hp    Erliansaurus
## Falcarius.hp       -2.62972535 -0.9299619       Falcarius.hp       Falcarius
## Guanlong.hp        -4.10304303  2.5541163        Guanlong.hp        Guanlong
## Haplocheirus.hp    -2.66357265 -1.5746950    Haplocheirus.hp    Haplocheirus
## Linhenykus.hp      -4.16218631  2.5353954      Linhenykus.hp      Linhenykus
## Manis.hp           -4.23849061  3.1852543           Manis.hp           Manis
## Mononykus.hp       -4.03102550  2.4064425       Mononykus.hp       Mononykus
## Nothronychus.hp     1.15428179 -2.6160904    Nothronychus.hp    Nothronychus
## Puma.hp            -4.50500872  5.7497375            Puma.hp            Puma
## Shishugounykus.hp  -4.55826939  2.9242360  Shishugounykus.hp  Shishugounykus
## Tamandua.hp         2.12800856 -6.3318209        Tamandua.hp        Tamandua
## Therizinosaurus.hp 20.77900846  3.4247211 Therizinosaurus.hp Therizinosaurus
## Tugulusaurus.hp    -4.81502435  6.4260867    Tugulusaurus.hp    Tugulusaurus
##                                Clade
## Allosaurus.p        Non-maniraptoran
## Alxasaurus.p         Therizinosauria
## Aorun.p            Alvarezsaurioidea
## Bannykus.p         Alvarezsaurioidea
## Beipiaosaurus.p      Therizinosauria
## Eremotherium.p               Mammals
## Erliansaurus.p       Therizinosauria
## Falcarius.p          Therizinosauria
## Guanlong.p          Non-maniraptoran
## Haplocheirus.p     Alvarezsaurioidea
## Linhenykus.p       Alvarezsaurioidea
## Manis.p                      Mammals
## Mononykus.p        Alvarezsaurioidea
## Nothronychus.p       Therizinosauria
## Puma.p                       Mammals
## Shishugounykus.p   Alvarezsaurioidea
## Tamandua.p                   Mammals
## Therizinosaurus.p    Therizinosauria
## Tugulusaurus.p     Alvarezsaurioidea
## Allosaurus.sd       Non-maniraptoran
## Alxasaurus.sd        Therizinosauria
## Aorun.sd           Alvarezsaurioidea
## Bannykus.sd        Alvarezsaurioidea
## Beipiaosaurus.sd     Therizinosauria
## Eremotherium.sd              Mammals
## Erliansaurus.sd      Therizinosauria
## Falcarius.sd         Therizinosauria
## Guanlong.sd         Non-maniraptoran
## Haplocheirus.sd    Alvarezsaurioidea
## Linhenykus.sd      Alvarezsaurioidea
## Manis.sd                     Mammals
## Mononykus.sd       Alvarezsaurioidea
## Nothronychus.sd      Therizinosauria
## Puma.sd                      Mammals
## Shishugounykus.sd  Alvarezsaurioidea
## Tamandua.sd                  Mammals
## Therizinosaurus.sd   Therizinosauria
## Tugulusaurus.sd    Alvarezsaurioidea
## Allosaurus.hp       Non-maniraptoran
## Alxasaurus.hp        Therizinosauria
## Aorun.hp           Alvarezsaurioidea
## Bannykus.hp        Alvarezsaurioidea
## Beipiaosaurus.hp     Therizinosauria
## Eremotherium.hp              Mammals
## Erliansaurus.hp      Therizinosauria
## Falcarius.hp         Therizinosauria
## Guanlong.hp         Non-maniraptoran
## Haplocheirus.hp    Alvarezsaurioidea
## Linhenykus.hp      Alvarezsaurioidea
## Manis.hp                     Mammals
## Mononykus.hp       Alvarezsaurioidea
## Nothronychus.hp      Therizinosauria
## Puma.hp                      Mammals
## Shishugounykus.hp  Alvarezsaurioidea
## Tamandua.hp                  Mammals
## Therizinosaurus.hp   Therizinosauria
## Tugulusaurus.hp    Alvarezsaurioidea
```

```
Coord$Function[1:19]<-c("piercing")
Coord$Function[20:38]<-c("scratch_digging")
Coord$Function[39:57]<-c("hook_and_pull")
Coord2<-Coord[order(Coord[,5]),]
Coord2
```

```
##                            PC1        PC2        Function          Genera
## Aorun.p            -2.53687163  0.1436204        piercing           Aorun
## Bannykus.p          0.95231302 -2.6743476        piercing        Bannykus
## Haplocheirus.p     -1.30671697 -1.4332262        piercing    Haplocheirus
## Linhenykus.p       -4.41812298  4.2021227        piercing      Linhenykus
## Mononykus.p        -3.56290532  1.4871996        piercing       Mononykus
## Shishugounykus.p   -3.94190947  3.1544965        piercing  Shishugounykus
## Tugulusaurus.p     -3.13483590  2.0373422        piercing    Tugulusaurus
## Aorun.sd            2.48152554 -2.3874001 scratch_digging           Aorun
## Bannykus.sd         4.78836331 -3.8180150 scratch_digging        Bannykus
## Haplocheirus.sd     2.11634198 -1.4224292 scratch_digging    Haplocheirus
## Linhenykus.sd      -4.41812506  4.2020874 scratch_digging      Linhenykus
## Mononykus.sd       -3.56290888  1.4872153 scratch_digging       Mononykus
## Shishugounykus.sd  -1.15998242  0.7198907 scratch_digging  Shishugounykus
## Tugulusaurus.sd    -2.15120564  1.8696094 scratch_digging    Tugulusaurus
## Aorun.hp           -2.74668706 -1.5153310   hook_and_pull           Aorun
## Bannykus.hp        -2.08910621 -1.9456923   hook_and_pull        Bannykus
## Haplocheirus.hp    -2.66357265 -1.5746950   hook_and_pull    Haplocheirus
## Linhenykus.hp      -4.16218631  2.5353954   hook_and_pull      Linhenykus
## Mononykus.hp       -4.03102550  2.4064425   hook_and_pull       Mononykus
## Shishugounykus.hp  -4.55826939  2.9242360   hook_and_pull  Shishugounykus
## Tugulusaurus.hp    -4.81502435  6.4260867   hook_and_pull    Tugulusaurus
## Eremotherium.p     -0.86870155 -1.4597797        piercing    Eremotherium
## Manis.p            -2.90212515 -0.3406726        piercing           Manis
## Puma.p             -3.33456905  1.8648867        piercing            Puma
## Tamandua.p          3.45214163 -6.2439935        piercing        Tamandua
## Eremotherium.sd    -0.30507397 -1.1973785 scratch_digging    Eremotherium
## Manis.sd           -1.85871510 -2.9528135 scratch_digging           Manis
## Puma.sd            -2.13270643  2.1343638 scratch_digging            Puma
## Tamandua.sd         9.74696208 -4.0015429 scratch_digging        Tamandua
## Eremotherium.hp    -3.23549043  0.4168465   hook_and_pull    Eremotherium
## Manis.hp           -4.23849061  3.1852543   hook_and_pull           Manis
## Puma.hp            -4.50500872  5.7497375   hook_and_pull            Puma
## Tamandua.hp         2.12800856 -6.3318209   hook_and_pull        Tamandua
## Allosaurus.p       -3.34322735  1.5024415        piercing      Allosaurus
## Guanlong.p         -2.45530339  0.2594165        piercing        Guanlong
## Allosaurus.sd       2.40864451 -2.2084409 scratch_digging      Allosaurus
## Guanlong.sd        -0.37376791  0.4249703 scratch_digging        Guanlong
## Allosaurus.hp      -2.04750450 -1.4825623   hook_and_pull      Allosaurus
## Guanlong.hp        -4.10304303  2.5541163   hook_and_pull        Guanlong
## Alxasaurus.p       -4.25547189  3.8567721        piercing      Alxasaurus
## Beipiaosaurus.p     3.81786982 -4.0764147        piercing   Beipiaosaurus
## Erliansaurus.p     -1.31118643 -2.7903685        piercing    Erliansaurus
## Falcarius.p        -0.05572564 -1.9047215        piercing       Falcarius
## Nothronychus.p      1.75663273 -3.5751840        piercing    Nothronychus
## Therizinosaurus.p  21.89583870  6.1195515        piercing Therizinosaurus
## Alxasaurus.sd      -0.95532526 -3.3378570 scratch_digging      Alxasaurus
## Beipiaosaurus.sd    4.87982360 -3.9494467 scratch_digging   Beipiaosaurus
## Erliansaurus.sd     0.13827691 -2.3141258 scratch_digging    Erliansaurus
## Falcarius.sd        4.22618045 -3.9608883 scratch_digging       Falcarius
## Nothronychus.sd     0.31039204  0.9309279 scratch_digging    Nothronychus
## Therizinosaurus.sd 21.26729943  7.3602389 scratch_digging Therizinosaurus
## Alxasaurus.hp      -3.79771123  2.9332823   hook_and_pull      Alxasaurus
## Beipiaosaurus.hp   -0.91745173 -3.2212909   hook_and_pull   Beipiaosaurus
## Erliansaurus.hp    -3.41412410 -0.6467804   hook_and_pull    Erliansaurus
## Falcarius.hp       -2.62972535 -0.9299619   hook_and_pull       Falcarius
## Nothronychus.hp     1.15428179 -2.6160904   hook_and_pull    Nothronychus
## Therizinosaurus.hp 20.77900846  3.4247211   hook_and_pull Therizinosaurus
##                                Clade
## Aorun.p            Alvarezsaurioidea
## Bannykus.p         Alvarezsaurioidea
## Haplocheirus.p     Alvarezsaurioidea
## Linhenykus.p       Alvarezsaurioidea
## Mononykus.p        Alvarezsaurioidea
## Shishugounykus.p   Alvarezsaurioidea
## Tugulusaurus.p     Alvarezsaurioidea
## Aorun.sd           Alvarezsaurioidea
## Bannykus.sd        Alvarezsaurioidea
## Haplocheirus.sd    Alvarezsaurioidea
## Linhenykus.sd      Alvarezsaurioidea
## Mononykus.sd       Alvarezsaurioidea
## Shishugounykus.sd  Alvarezsaurioidea
## Tugulusaurus.sd    Alvarezsaurioidea
## Aorun.hp           Alvarezsaurioidea
## Bannykus.hp        Alvarezsaurioidea
## Haplocheirus.hp    Alvarezsaurioidea
## Linhenykus.hp      Alvarezsaurioidea
## Mononykus.hp       Alvarezsaurioidea
## Shishugounykus.hp  Alvarezsaurioidea
## Tugulusaurus.hp    Alvarezsaurioidea
## Eremotherium.p               Mammals
## Manis.p                      Mammals
## Puma.p                       Mammals
## Tamandua.p                   Mammals
## Eremotherium.sd              Mammals
## Manis.sd                     Mammals
## Puma.sd                      Mammals
## Tamandua.sd                  Mammals
## Eremotherium.hp              Mammals
## Manis.hp                     Mammals
## Puma.hp                      Mammals
## Tamandua.hp                  Mammals
## Allosaurus.p        Non-maniraptoran
## Guanlong.p          Non-maniraptoran
## Allosaurus.sd       Non-maniraptoran
## Guanlong.sd         Non-maniraptoran
## Allosaurus.hp       Non-maniraptoran
## Guanlong.hp         Non-maniraptoran
## Alxasaurus.p         Therizinosauria
## Beipiaosaurus.p      Therizinosauria
## Erliansaurus.p       Therizinosauria
## Falcarius.p          Therizinosauria
## Nothronychus.p       Therizinosauria
## Therizinosaurus.p    Therizinosauria
## Alxasaurus.sd        Therizinosauria
## Beipiaosaurus.sd     Therizinosauria
## Erliansaurus.sd      Therizinosauria
## Falcarius.sd         Therizinosauria
## Nothronychus.sd      Therizinosauria
## Therizinosaurus.sd   Therizinosauria
## Alxasaurus.hp        Therizinosauria
## Beipiaosaurus.hp     Therizinosauria
## Erliansaurus.hp      Therizinosauria
## Falcarius.hp         Therizinosauria
## Nothronychus.hp      Therizinosauria
## Therizinosaurus.hp   Therizinosauria
```

```
##reorder genera

Coord2$Genera<- factor(Coord2$Genera, 
                       levels = c('Aorun','Shishugounykus','Haplocheirus', 'Tugulusaurus',
                                  'Bannykus','Linhenykus','Mononykus','Falcarius','Beipiaosaurus',
                                  'Alxasaurus','Nothronychus','Erliansaurus','Therizinosaurus',
                                  'Allosaurus','Guanlong','Eremotherium','Tamandua','Puma','Manis'))
Coord2
```

```
##                            PC1        PC2        Function          Genera
## Aorun.p            -2.53687163  0.1436204        piercing           Aorun
## Bannykus.p          0.95231302 -2.6743476        piercing        Bannykus
## Haplocheirus.p     -1.30671697 -1.4332262        piercing    Haplocheirus
## Linhenykus.p       -4.41812298  4.2021227        piercing      Linhenykus
## Mononykus.p        -3.56290532  1.4871996        piercing       Mononykus
## Shishugounykus.p   -3.94190947  3.1544965        piercing  Shishugounykus
## Tugulusaurus.p     -3.13483590  2.0373422        piercing    Tugulusaurus
## Aorun.sd            2.48152554 -2.3874001 scratch_digging           Aorun
## Bannykus.sd         4.78836331 -3.8180150 scratch_digging        Bannykus
## Haplocheirus.sd     2.11634198 -1.4224292 scratch_digging    Haplocheirus
## Linhenykus.sd      -4.41812506  4.2020874 scratch_digging      Linhenykus
## Mononykus.sd       -3.56290888  1.4872153 scratch_digging       Mononykus
## Shishugounykus.sd  -1.15998242  0.7198907 scratch_digging  Shishugounykus
## Tugulusaurus.sd    -2.15120564  1.8696094 scratch_digging    Tugulusaurus
## Aorun.hp           -2.74668706 -1.5153310   hook_and_pull           Aorun
## Bannykus.hp        -2.08910621 -1.9456923   hook_and_pull        Bannykus
## Haplocheirus.hp    -2.66357265 -1.5746950   hook_and_pull    Haplocheirus
## Linhenykus.hp      -4.16218631  2.5353954   hook_and_pull      Linhenykus
## Mononykus.hp       -4.03102550  2.4064425   hook_and_pull       Mononykus
## Shishugounykus.hp  -4.55826939  2.9242360   hook_and_pull  Shishugounykus
## Tugulusaurus.hp    -4.81502435  6.4260867   hook_and_pull    Tugulusaurus
## Eremotherium.p     -0.86870155 -1.4597797        piercing    Eremotherium
## Manis.p            -2.90212515 -0.3406726        piercing           Manis
## Puma.p             -3.33456905  1.8648867        piercing            Puma
## Tamandua.p          3.45214163 -6.2439935        piercing        Tamandua
## Eremotherium.sd    -0.30507397 -1.1973785 scratch_digging    Eremotherium
## Manis.sd           -1.85871510 -2.9528135 scratch_digging           Manis
## Puma.sd            -2.13270643  2.1343638 scratch_digging            Puma
## Tamandua.sd         9.74696208 -4.0015429 scratch_digging        Tamandua
## Eremotherium.hp    -3.23549043  0.4168465   hook_and_pull    Eremotherium
## Manis.hp           -4.23849061  3.1852543   hook_and_pull           Manis
## Puma.hp            -4.50500872  5.7497375   hook_and_pull            Puma
## Tamandua.hp         2.12800856 -6.3318209   hook_and_pull        Tamandua
## Allosaurus.p       -3.34322735  1.5024415        piercing      Allosaurus
## Guanlong.p         -2.45530339  0.2594165        piercing        Guanlong
## Allosaurus.sd       2.40864451 -2.2084409 scratch_digging      Allosaurus
## Guanlong.sd        -0.37376791  0.4249703 scratch_digging        Guanlong
## Allosaurus.hp      -2.04750450 -1.4825623   hook_and_pull      Allosaurus
## Guanlong.hp        -4.10304303  2.5541163   hook_and_pull        Guanlong
## Alxasaurus.p       -4.25547189  3.8567721        piercing      Alxasaurus
## Beipiaosaurus.p     3.81786982 -4.0764147        piercing   Beipiaosaurus
## Erliansaurus.p     -1.31118643 -2.7903685        piercing    Erliansaurus
## Falcarius.p        -0.05572564 -1.9047215        piercing       Falcarius
## Nothronychus.p      1.75663273 -3.5751840        piercing    Nothronychus
## Therizinosaurus.p  21.89583870  6.1195515        piercing Therizinosaurus
## Alxasaurus.sd      -0.95532526 -3.3378570 scratch_digging      Alxasaurus
## Beipiaosaurus.sd    4.87982360 -3.9494467 scratch_digging   Beipiaosaurus
## Erliansaurus.sd     0.13827691 -2.3141258 scratch_digging    Erliansaurus
## Falcarius.sd        4.22618045 -3.9608883 scratch_digging       Falcarius
## Nothronychus.sd     0.31039204  0.9309279 scratch_digging    Nothronychus
## Therizinosaurus.sd 21.26729943  7.3602389 scratch_digging Therizinosaurus
## Alxasaurus.hp      -3.79771123  2.9332823   hook_and_pull      Alxasaurus
## Beipiaosaurus.hp   -0.91745173 -3.2212909   hook_and_pull   Beipiaosaurus
## Erliansaurus.hp    -3.41412410 -0.6467804   hook_and_pull    Erliansaurus
## Falcarius.hp       -2.62972535 -0.9299619   hook_and_pull       Falcarius
## Nothronychus.hp     1.15428179 -2.6160904   hook_and_pull    Nothronychus
## Therizinosaurus.hp 20.77900846  3.4247211   hook_and_pull Therizinosaurus
##                                Clade
## Aorun.p            Alvarezsaurioidea
## Bannykus.p         Alvarezsaurioidea
## Haplocheirus.p     Alvarezsaurioidea
## Linhenykus.p       Alvarezsaurioidea
## Mononykus.p        Alvarezsaurioidea
## Shishugounykus.p   Alvarezsaurioidea
## Tugulusaurus.p     Alvarezsaurioidea
## Aorun.sd           Alvarezsaurioidea
## Bannykus.sd        Alvarezsaurioidea
## Haplocheirus.sd    Alvarezsaurioidea
## Linhenykus.sd      Alvarezsaurioidea
## Mononykus.sd       Alvarezsaurioidea
## Shishugounykus.sd  Alvarezsaurioidea
## Tugulusaurus.sd    Alvarezsaurioidea
## Aorun.hp           Alvarezsaurioidea
## Bannykus.hp        Alvarezsaurioidea
## Haplocheirus.hp    Alvarezsaurioidea
## Linhenykus.hp      Alvarezsaurioidea
## Mononykus.hp       Alvarezsaurioidea
## Shishugounykus.hp  Alvarezsaurioidea
## Tugulusaurus.hp    Alvarezsaurioidea
## Eremotherium.p               Mammals
## Manis.p                      Mammals
## Puma.p                       Mammals
## Tamandua.p                   Mammals
## Eremotherium.sd              Mammals
## Manis.sd                     Mammals
## Puma.sd                      Mammals
## Tamandua.sd                  Mammals
## Eremotherium.hp              Mammals
## Manis.hp                     Mammals
## Puma.hp                      Mammals
## Tamandua.hp                  Mammals
## Allosaurus.p        Non-maniraptoran
## Guanlong.p          Non-maniraptoran
## Allosaurus.sd       Non-maniraptoran
## Guanlong.sd         Non-maniraptoran
## Allosaurus.hp       Non-maniraptoran
## Guanlong.hp         Non-maniraptoran
## Alxasaurus.p         Therizinosauria
## Beipiaosaurus.p      Therizinosauria
## Erliansaurus.p       Therizinosauria
## Falcarius.p          Therizinosauria
## Nothronychus.p       Therizinosauria
## Therizinosaurus.p    Therizinosauria
## Alxasaurus.sd        Therizinosauria
## Beipiaosaurus.sd     Therizinosauria
## Erliansaurus.sd      Therizinosauria
## Falcarius.sd         Therizinosauria
## Nothronychus.sd      Therizinosauria
## Therizinosaurus.sd   Therizinosauria
## Alxasaurus.hp        Therizinosauria
## Beipiaosaurus.hp     Therizinosauria
## Erliansaurus.hp      Therizinosauria
## Falcarius.hp         Therizinosauria
## Nothronychus.hp      Therizinosauria
## Therizinosaurus.hp   Therizinosauria
```

```
##reordered plot PC1

plotPC1<-ggplot(Coord2, aes(x = PC1, y = Genera, fill = Clade)) +
  geom_density_ridges(alpha=0.8, stat="binline", bins=30,colour='grey') +
  theme_ridges() + 
  theme(axis.text.y = element_text( face = "italic"))+
  theme(legend.position = "none")+
  xlab('Coordinates on FPC1')+
  ylab('Genera')
plotPC1
```

```
ggsave(file='PC1 divergence combine figure by clade.svg', plot=plotPC1,units="mm", width=400, height=200)
ggsave(file='PC1 divergence combine figure by clade.pdf', plot=plotPC1,units="mm", width=400, height=200)

##reordered plot PC2
plotPC2<-ggplot(Coord2, aes(x = PC2, y = Genera, fill = Clade)) +
  geom_density_ridges(alpha=0.8, stat="binline", bins=30,colour='grey') +
  theme_ridges() + 
  theme(axis.text.y = element_text( face = "italic"))+
  theme(legend.position = "none")+
  xlab('Coordinates on FPC2')+
  ylab('Genera')
plotPC2
```

```
ggsave(file='PC2 divergence combine figure by clade.svg', plot=plotPC2,units="mm", width=400, height=200)
ggsave(file='PC2 divergence combine figure by clade.pdf', plot=plotPC2,units="mm", width=400, height=200)

plotPC1<-ggplot(Coord2, aes(x = PC1, y = Genera, fill = Function,colour=Clade)) +
  geom_density_ridges(alpha=0.8, stat="binline", bins=30,size = 1,scale=0.7) +
 ## IF YOU LIKE GREY### scale_fill_grey(start=0.9, end=0.5)+###
  scale_fill_manual(values=c(piercing = "red", scratch_digging = "blue", hook_and_pull = "green" ))+
  theme_ridges() + 
  theme(axis.text.y = element_text( face = "italic"))+
  theme()+
  xlab('Coordinates on FPC1')+
  ylab('Genera')
plotPC1
```

```
ggsave(file='PC1 divergence combine figure by function.svg', plot=plotPC1,units="mm", width=400, height=200)
ggsave(file='PC1 divergence combine figure by function.pdf', plot=plotPC1,units="mm", width=400, height=200)

plotPC2<-ggplot(Coord2, aes(x = PC2, y = Genera, fill = Function,colour=Clade)) +
  geom_density_ridges(alpha=0.8, stat="binline", bins=30,size = 1,scale=0.7) +
  scale_fill_manual(values=c(piercing = "red", scratch_digging = "blue", hook_and_pull = "green" ))+
  theme_ridges() + 
  theme(axis.text.y = element_text( face = "italic"))+
  theme()+
  xlab('Coordinates on FPC2')+
  ylab('Genera')
plotPC2
```

```
ggsave(file='PC2 divergence combine figure by function.svg', plot=plotPC2,units="mm", width=400, height=200)
ggsave(file='PC2 divergence combine figure by function.pdf', plot=plotPC2,units="mm", width=400, height=200)
myplot<-grid.arrange(plotPC1,plotPC2, ncol=1, nrow=2)
```

```
myplot
```

```
## TableGrob (2 x 1) "arrange": 2 grobs
##   z     cells    name           grob
## 1 1 (1-1,1-1) arrange gtable[layout]
## 2 2 (2-2,1-1) arrange gtable[layout]
```

```
ggsave(file='PC1 and 2 divergence by function.svg', plot=myplot,units="mm", width=400, height=400)
ggsave(file='PC1 and 2 divergence by function.pdf', plot=myplot,units="mm", width=400, height=400)
```

# 6.Caculating the distance between vertexes

## 6.1 Caculating between individuals

```
# Coordinates of individuals
#::::::::::::::::::::::::::::::::::
ind.coord <- Claw.pca$x
ind.coord
```

```
##                            PC1        PC2          PC3         PC4
## Allosaurus.p       -3.34322735  1.5024415 -0.283485671 -0.34270920
## Alxasaurus.p       -4.25547189  3.8567721  0.968591611  0.08721399
## Aorun.p            -2.53687163  0.1436204 -0.570672273  0.73341391
## Bannykus.p          0.95231302 -2.6743476 -1.315046636  0.82448412
## Beipiaosaurus.p     3.81786982 -4.0764147  0.663037687 -0.51422791
## Eremotherium.p     -0.86870155 -1.4597797 -0.248024309 -1.75369054
## Erliansaurus.p     -1.31118643 -2.7903685 -0.344203520 -0.37516780
## Falcarius.p        -0.05572564 -1.9047215 -0.416316361 -0.23129536
## Guanlong.p         -2.45530339  0.2594165  0.018335690 -0.35313360
## Haplocheirus.p     -1.30671697 -1.4332262 -0.213730587  0.47816196
## Linhenykus.p       -4.41812298  4.2021227  0.265993563  1.60316911
## Manis.p            -2.90212515 -0.3406726 -1.499151122 -0.59876132
## Mononykus.p        -3.56290532  1.4871996 -1.755561286  1.16844639
## Nothronychus.p      1.75663273 -3.5751840 -0.299306302  0.24297926
## Puma.p             -3.33456905  1.8648867  0.569785594 -0.18090112
## Shishugounykus.p   -3.94190947  3.1544965  1.694840912 -0.05927715
## Tamandua.p          3.45214163 -6.2439935  3.616944678  1.07636588
## Therizinosaurus.p  21.89583870  6.1195515 -1.392877650 -0.28444559
## Tugulusaurus.p     -3.13483590  2.0373422  2.160661465 -0.24668631
## Allosaurus.sd       2.40864451 -2.2084409  0.040838848 -0.64252013
## Alxasaurus.sd      -0.95532526 -3.3378570 -0.158130021 -1.41236044
## Aorun.sd            2.48152554 -2.3874001 -0.009313851 -0.28450104
## Bannykus.sd         4.78836331 -3.8180150 -0.238886670 -0.97482489
## Beipiaosaurus.sd    4.87982360 -3.9494467  0.097597024 -0.86851566
## Eremotherium.sd    -0.30507397 -1.1973785 -1.177209968 -1.72176884
## Erliansaurus.sd     0.13827691 -2.3141258 -0.325772888  0.21834034
## Falcarius.sd        4.22618045 -3.9608883  0.266866241 -0.70222579
## Guanlong.sd        -0.37376791  0.4249703  0.029826995  0.04118170
## Haplocheirus.sd     2.11634198 -1.4224292 -0.702595873 -0.48661276
## Linhenykus.sd      -4.41812506  4.2020874  0.265921136  1.60319849
## Manis.sd           -1.85871510 -2.9528135 -1.125617339 -1.70383349
## Mononykus.sd       -3.56290888  1.4872153 -1.755588351  1.16844423
## Nothronychus.sd     0.31039204  0.9309279 -0.507208412  1.37820611
## Puma.sd            -2.13270643  2.1343638  1.170282819 -0.09216460
## Shishugounykus.sd  -1.15998242  0.7198907 -0.519918949  1.43754927
## Tamandua.sd         9.74696208 -4.0015429  3.733625456  2.04401399
## Therizinosaurus.sd 21.26729943  7.3602389 -2.738420516 -1.89605637
## Tugulusaurus.sd    -2.15120564  1.8696094  2.298429270  0.41183879
## Allosaurus.hp      -2.04750450 -1.4825623 -0.314651076 -0.39195941
## Alxasaurus.hp      -3.79771123  2.9332823  0.359079842  0.29295689
## Aorun.hp           -2.74668706 -1.5153310 -1.568007885  0.49990871
## Bannykus.hp        -2.08910621 -1.9456923 -1.703866342  0.31565575
## Beipiaosaurus.hp   -0.91745173 -3.2212909 -0.772218892 -0.32273401
## Eremotherium.hp    -3.23549043  0.4168465 -1.691245211 -1.09242193
## Erliansaurus.hp    -3.41412410 -0.6467804 -1.552548924 -0.36663212
## Falcarius.hp       -2.62972535 -0.9299619 -1.627619305 -0.53516110
## Guanlong.hp        -4.10304303  2.5541163 -0.525680668 -0.11686757
## Haplocheirus.hp    -2.66357265 -1.5746950 -0.525976516 -0.08639338
## Linhenykus.hp      -4.16218631  2.5353954 -1.360361427  0.98195190
## Manis.hp           -4.23849061  3.1852543 -0.170677713  1.09097847
## Mononykus.hp       -4.03102550  2.4064425 -1.849324781  1.76856272
## Nothronychus.hp     1.15428179 -2.6160904 -0.458269361  0.99387454
## Puma.hp            -4.50500872  5.7497375  3.737623318 -1.93428326
## Shishugounykus.hp  -4.55826939  2.9242360  0.133154579  0.36633316
## Tamandua.hp         2.12800856 -6.3318209  3.458629305  0.44916975
## Therizinosaurus.hp 20.77900846  3.4247211  1.803375138  2.03562987
## Tugulusaurus.hp    -4.81502435  6.4260867  4.364045481 -2.73989661
##                              PC5          PC6          PC7          PC8
## Allosaurus.p       -0.0803834667  0.048666140 -0.277900283  0.231839785
## Alxasaurus.p       -0.0007094108 -0.242251885 -0.121083159 -0.038016410
## Aorun.p            -0.1208205340 -0.017315659  0.107766425  0.280608743
## Bannykus.p          1.0238962142  0.023122944  0.201095925 -0.247819176
## Beipiaosaurus.p    -0.3393671392  0.202394490  0.051158895 -0.028703088
## Eremotherium.p     -0.0220844138  0.072215270 -0.696725768 -0.176682732
## Erliansaurus.p      0.2245772333  0.557721656 -0.062968096  0.252244092
## Falcarius.p        -0.8847587740  0.361634774 -0.138734364 -0.306011593
## Guanlong.p          0.7625990566 -0.661855950 -0.063855977  0.365891232
## Haplocheirus.p      0.6625423566  0.153966407  0.360014305  0.317276519
## Linhenykus.p       -0.9302204415  0.158293518  0.022043176 -0.762216386
## Manis.p             0.1977846251  0.927361339  0.279380797 -0.134959664
## Mononykus.p         0.0841033824  0.401892091  0.679228994  0.064613330
## Nothronychus.p      0.2481384147 -0.248385397  0.342740106 -0.493822518
## Puma.p             -0.0824510942 -0.683605493 -0.611024404 -0.167857492
## Shishugounykus.p   -0.9689452817  0.396706682 -0.498471551  0.693902624
## Tamandua.p         -0.7719227094 -0.450450258  0.207845175 -0.022760826
## Therizinosaurus.p  -0.1252645009 -0.006730212  0.002182203 -0.292593321
## Tugulusaurus.p     -0.5938853580  1.101313525  1.008229369  0.119627576
## Allosaurus.sd       0.1782898879 -0.059174895  0.017084358  0.125008016
## Alxasaurus.sd       0.2461749306  0.233004308  0.033077102  0.097277485
## Aorun.sd           -0.8504325778 -0.128456751 -0.116684049 -0.489252939
## Bannykus.sd        -1.1978091922 -0.307488970 -0.428504016 -0.837022819
## Beipiaosaurus.sd    0.1784973935  0.636133373 -0.019253346  0.308175320
## Eremotherium.sd     0.8086968064  0.362955395 -0.349093968  0.143764613
## Erliansaurus.sd     0.6470187491  0.681518406 -0.102675730 -0.107019473
## Falcarius.sd       -0.6268617955 -0.090938393  0.076648193 -0.196294542
## Guanlong.sd         0.5936843086 -0.456990902 -0.260687497 -0.032207684
## Haplocheirus.sd     0.4509015460  0.661717244 -0.185121524  0.013800037
## Linhenykus.sd      -0.9301801793  0.158228272  0.022054811 -0.762297434
## Manis.sd           -0.5426757292  1.313586864 -0.448316274 -0.304076426
## Mononykus.sd        0.0841451223  0.401850088  0.679267416  0.064591716
## Nothronychus.sd    -1.2066208556 -0.482597229 -0.825455574  0.218751740
## Puma.sd             0.0445390674 -0.861326931 -0.344818381  0.003140411
## Shishugounykus.sd   0.1793816372 -0.598133758 -0.273556374 -0.487788346
## Tamandua.sd         0.3477662892 -0.203629124  0.193114260  0.546024420
## Therizinosaurus.sd -1.6074589952 -0.802991439  1.202597630  0.536805852
## Tugulusaurus.sd    -0.3573124918  1.496698100  0.626419695  0.068499417
## Allosaurus.hp       0.0069825754 -0.627857606  0.115681109  0.049170289
## Alxasaurus.hp      -0.6042905163  0.064893641 -1.023265630  1.003609537
## Aorun.hp            0.6599319091 -0.485074377  0.662683898  0.276360406
## Bannykus.hp         0.1743484719 -0.340597984  0.123143654  0.135144775
## Beipiaosaurus.hp    0.0807309840 -0.670469851  0.560303892 -0.078866392
## Eremotherium.hp     1.0002076379 -0.155152211 -0.447527168  0.450362558
## Erliansaurus.hp     0.2711722696  0.035405197  0.331605912  0.018586682
## Falcarius.hp       -0.5984572731  0.027868243 -1.192762480 -0.232431085
## Guanlong.hp         0.7566261588 -0.911633614 -0.432805274  0.169044428
## Haplocheirus.hp     0.3246062666 -0.265203378  0.555877908  0.401990033
## Linhenykus.hp       0.6341281442 -0.333432994  0.432940841 -0.104239079
## Manis.hp           -0.8475848956  0.924570697  0.043271384  0.295503614
## Mononykus.hp        0.3283751208 -0.459357932  0.551241357 -0.185401914
## Nothronychus.hp     0.8958048157 -0.245749953  0.249410885 -0.404438762
## Puma.hp             0.5642585309  0.505567365  0.876896975 -0.297057641
## Shishugounykus.hp  -0.9343167207 -0.193226392 -0.978457649  0.225653562
## Tamandua.hp        -0.9684068158 -0.945366897  0.403756860  0.333880437
## Therizinosaurus.hp  2.2055375482  1.007307825 -1.226159351 -0.115840782
## Tugulusaurus.hp     1.3277737077 -0.981147418  0.107144378 -0.505470725
##                             PC9         PC10        PC11         PC12
## Allosaurus.p        0.320855540 -0.193564568 -0.04566667 -0.021932364
## Alxasaurus.p        0.077330038 -0.166548843  0.12023527 -0.002297907
## Aorun.p             0.117662539  0.394039651 -0.27092321  0.020151372
## Bannykus.p          0.251471272  0.361412874  0.12074083 -0.309025863
## Beipiaosaurus.p    -0.089317623 -0.257396019 -0.33595310  0.199567189
## Eremotherium.p      0.255397901 -0.272723477  0.06770060 -0.081176664
## Erliansaurus.p     -0.427411988  0.060176259  0.04571854 -0.181961090
## Falcarius.p         0.300842877 -0.183756686 -0.24048464  0.125395216
## Guanlong.p          0.131238487  0.156416436 -0.09361790  0.121696562
## Haplocheirus.p     -0.482788618  0.055935727 -0.04853404 -0.125246761
## Linhenykus.p       -0.673217748  0.159122712 -0.08651743  0.020241199
## Manis.p            -0.213878182  0.187945061 -0.13211877 -0.050257637
## Mononykus.p         0.190340785 -0.098228653 -0.05834908 -0.005736390
## Nothronychus.p      0.185682540 -0.490212590  0.16498162 -0.219814561
## Puma.p              0.120206007 -0.139535239  0.07911995  0.059834351
## Shishugounykus.p    0.318895479  0.092167986 -0.25114720 -0.214006825
## Tamandua.p          0.221509439  0.482900859  0.34314155 -0.070690686
## Therizinosaurus.p   0.513885771  0.689999929  0.09475903  0.459384579
## Tugulusaurus.p      0.198857639 -0.266498759  0.03256698  0.184016139
## Allosaurus.sd       0.191606666  0.086241963 -0.10323004 -0.074898771
## Alxasaurus.sd      -0.206491774  0.008692859  0.15304303 -0.030468337
## Aorun.sd            0.368112847  0.230961672 -0.21983096 -0.134621705
## Bannykus.sd         0.172213877 -0.336466982 -0.22020380  0.048986562
## Beipiaosaurus.sd   -0.393984077  0.018400452 -0.21286152 -0.200255813
## Eremotherium.sd    -0.302500345 -0.059963497  0.45758681  0.138439720
## Erliansaurus.sd     0.169661487  0.599398247  0.16728552 -0.143730942
## Falcarius.sd       -0.051499070  0.094369751  0.06226042 -0.082277175
## Guanlong.sd         0.123636468  0.110021869  0.05401313 -0.020309484
## Haplocheirus.sd    -0.161011259  0.271596831 -0.10609852  0.279903036
## Linhenykus.sd      -0.673266210  0.159132884 -0.08649266  0.020303215
## Manis.sd           -0.166588783 -0.199143375  0.17332437  0.064388097
## Mononykus.sd        0.190299390 -0.098155787 -0.05838756 -0.005734138
## Nothronychus.sd    -0.441177088  0.059743812  0.20398902 -0.132506633
## Puma.sd             0.111844626 -0.149022140  0.14338674  0.110904051
## Shishugounykus.sd  -0.022961022 -0.101872199  0.31088195  0.043611280
## Tamandua.sd        -0.504266423 -0.180924776  0.04368938  0.399488545
## Therizinosaurus.sd -0.389894739 -0.235895816  0.15488401 -0.331552648
## Tugulusaurus.sd     0.338854124  0.060950783  0.27661988 -0.163707597
## Allosaurus.hp      -0.063969611 -0.161345692 -0.02319633  0.199389753
## Alxasaurus.hp       0.376366647 -0.029708803 -0.04496276 -0.077755744
## Aorun.hp           -0.071381040 -0.103744107 -0.06132330  0.118024470
## Bannykus.hp        -0.131207162 -0.088690861 -0.09806358  0.096889428
## Beipiaosaurus.hp    0.448665892  0.026951549 -0.34317386  0.009206499
## Eremotherium.hp    -0.152060029  0.140250203  0.19019376  0.048965230
## Erliansaurus.hp     0.056215511  0.089785382 -0.22818143 -0.007439098
## Falcarius.hp       -0.199617796  0.047510620  0.12164776  0.139714374
## Guanlong.hp         0.194002100 -0.132360785  0.17165541  0.039298977
## Haplocheirus.hp    -0.350386294  0.025815347 -0.20338540  0.264858902
## Linhenykus.hp       0.045399444 -0.020148317 -0.06161175 -0.035990127
## Manis.hp            0.006595240 -0.047563325  0.10287631  0.028950011
## Mononykus.hp        0.221365652 -0.249181549  0.02192124 -0.064381093
## Nothronychus.hp     0.127830620 -0.129382206  0.21934737 -0.134566205
## Puma.hp             0.220875651 -0.187273416  0.20841097  0.209217638
## Shishugounykus.hp   0.020215414  0.072555462 -0.11702330  0.017541737
## Tamandua.hp         0.016689140 -0.003234623  0.05236715 -0.105569799
## Therizinosaurus.hp  0.003082724 -0.473592433 -0.28737375 -0.190223368
## Tugulusaurus.hp    -0.438832950  0.313638344 -0.31963605 -0.250232706
##                           PC13         PC14          PC15         PC16
## Allosaurus.p        0.10938645 -0.150595772 -0.0573629342 -0.039946941
## Alxasaurus.p        0.13014705  0.064566287  0.0354309763 -0.016669414
## Aorun.p            -0.08082073 -0.156161352  0.0555528244 -0.019717246
## Bannykus.p         -0.08500040 -0.155994204 -0.0153088967 -0.113004156
## Beipiaosaurus.p     0.05736425  0.033451787  0.0027172508  0.023125208
## Eremotherium.p      0.08322284 -0.190639056 -0.1847676015  0.002559593
## Erliansaurus.p     -0.07373892  0.021504676 -0.1092085856  0.008676733
## Falcarius.p         0.12428602 -0.027372299 -0.0225119148  0.074766514
## Guanlong.p         -0.13087444 -0.192887927 -0.0883559950 -0.021317720
## Haplocheirus.p     -0.14029515  0.281705584 -0.1198150104 -0.050454751
## Linhenykus.p        0.02943073 -0.002269575  0.0598868403  0.047479242
## Manis.p             0.05209250 -0.149214275 -0.0199728000  0.081321240
## Mononykus.p        -0.02161497 -0.137208190  0.0450995154 -0.002322071
## Nothronychus.p      0.36861353  0.071326588  0.1314113227 -0.072581530
## Puma.p             -0.15217950 -0.032485041 -0.0379217150 -0.062558341
## Shishugounykus.p    0.43833068  0.135294346 -0.0286599001  0.036417847
## Tamandua.p          0.04828163 -0.215166306  0.0225205984  0.174581411
## Therizinosaurus.p   0.20380363  0.069617050 -0.0231409014  0.034525255
## Tugulusaurus.p     -0.11385152  0.066917771 -0.0435229764 -0.018122600
## Allosaurus.sd      -0.05301759 -0.177998730  0.0130439610  0.090624055
## Alxasaurus.sd       0.07702712 -0.006808337  0.2671911968  0.021873503
## Aorun.sd           -0.08867484  0.162137448  0.1038939326 -0.070047399
## Bannykus.sd        -0.15508172 -0.038277176 -0.1483160839 -0.079641319
## Beipiaosaurus.sd   -0.21302969  0.036382380 -0.0251518307  0.149065268
## Eremotherium.sd     0.32835207 -0.089788648  0.3264258406 -0.069750321
## Erliansaurus.sd    -0.08469250  0.049757845 -0.1726201308 -0.079744992
## Falcarius.sd       -0.01247030  0.203536699  0.1476144495 -0.238045651
## Guanlong.sd        -0.07699771  0.133683503 -0.0622374140  0.167080315
## Haplocheirus.sd     0.04447058  0.128889875  0.0030925941 -0.071698991
## Linhenykus.sd       0.02930159 -0.002377017  0.0599419542  0.047429258
## Manis.sd            0.15098903 -0.113646749 -0.1764187217  0.105419816
## Mononykus.sd       -0.02157213 -0.137113898  0.0450584550 -0.002320364
## Nothronychus.sd    -0.13807214 -0.162183114 -0.1008871416 -0.195836522
## Puma.sd            -0.17472510  0.108082582  0.0109097767  0.064358636
## Shishugounykus.sd   0.01731880  0.110177234 -0.0118054199  0.156962965
## Tamandua.sd         0.24938339 -0.153246202 -0.1567192303 -0.161859378
## Therizinosaurus.sd -0.07370417 -0.039977732 -0.0591481585  0.020311020
## Tugulusaurus.sd    -0.05465593  0.020529926 -0.0715119439 -0.040074685
## Allosaurus.hp      -0.02708907 -0.007276475  0.0001331352  0.070434391
## Alxasaurus.hp       0.12498781  0.153773619 -0.0684264468 -0.005716353
## Aorun.hp            0.04318925  0.089067432 -0.0404720445  0.045940796
## Bannykus.hp         0.04182751 -0.099130378 -0.0860208414  0.017275086
## Beipiaosaurus.hp   -0.05736776  0.141877010  0.1125757282 -0.066445182
## Eremotherium.hp    -0.16058491  0.109809993  0.0852522728  0.054201146
## Erliansaurus.hp    -0.03540138 -0.091236083  0.1442739540 -0.043089671
## Falcarius.hp       -0.20361364  0.091263440  0.0138397441  0.013099619
## Guanlong.hp        -0.07534909 -0.023191332 -0.0062323973  0.009253618
## Haplocheirus.hp    -0.01438662  0.126225897 -0.0620266064  0.006603227
## Linhenykus.hp       0.11321864 -0.112318904  0.0219738815 -0.040270643
## Manis.hp            0.03684827  0.163504902  0.1359920699  0.020235594
## Mononykus.hp        0.08909380 -0.040368257 -0.0108226388  0.037359642
## Nothronychus.hp     0.11675077  0.298286162 -0.2749764795 -0.002950107
## Puma.hp            -0.45427638 -0.038390393  0.0221553637 -0.063083882
## Shishugounykus.hp  -0.14978518 -0.077888488  0.1101635059 -0.070589398
## Tamandua.hp        -0.13001937  0.016131391  0.2084374288  0.112639754
## Therizinosaurus.hp -0.18438123 -0.037469097  0.1719593324  0.043418496
## Tugulusaurus.hp     0.32960612 -0.028820422 -0.0722051443 -0.019179626
##                            PC17         PC18          PC19          PC20
## Allosaurus.p        0.044990442 -0.045148668 -0.0204655742  0.0060985902
## Alxasaurus.p        0.017833168 -0.010859171  0.0170464640 -0.0009530141
## Aorun.p            -0.048662318 -0.103330519  0.0322027112  0.0176981366
## Bannykus.p         -0.140877069  0.025481889 -0.1000631917 -0.0700836420
## Beipiaosaurus.p    -0.185371574  0.033011507  0.0973180013 -0.0720619612
## Eremotherium.p     -0.042009761 -0.113297634 -0.0121342883 -0.0332025488
## Erliansaurus.p      0.051348707  0.090966901 -0.0093099801  0.0574245569
## Falcarius.p        -0.033273942  0.069408011  0.0720125336 -0.0455357208
## Guanlong.p          0.029917232  0.001559148 -0.0073474126 -0.0334919846
## Haplocheirus.p      0.108861973 -0.040626645 -0.0202955782  0.0599492594
## Linhenykus.p       -0.018717663 -0.021482128 -0.0280688996 -0.0149087029
## Manis.p             0.061089058  0.028204546  0.0352922190  0.1041541196
## Mononykus.p         0.002832537 -0.013745755 -0.0069011749 -0.0179034407
## Nothronychus.p      0.074441067  0.052541069 -0.1047569708  0.0140909397
## Puma.p              0.011764513 -0.001822747 -0.0386359281  0.0069054785
## Shishugounykus.p   -0.006020648  0.005925455 -0.0095848993 -0.0282432027
## Tamandua.p          0.059412573  0.041798138  0.0536877739 -0.1072426197
## Therizinosaurus.p   0.080800333 -0.041617702 -0.0698262967  0.0149405131
## Tugulusaurus.p      0.033019236  0.004716875  0.0007509656 -0.0301963562
## Allosaurus.sd       0.082606782 -0.079160603 -0.0447604633  0.1001508467
## Alxasaurus.sd      -0.001108646 -0.110863738  0.0623560309  0.0875570516
## Aorun.sd           -0.076086061  0.032576324 -0.0323070345  0.0658504084
## Bannykus.sd         0.034469682 -0.015588057  0.0590536056  0.0218282271
## Beipiaosaurus.sd   -0.073120625 -0.084752458 -0.1437747088 -0.0514175422
## Eremotherium.sd    -0.083520969  0.014143989  0.0138020124  0.0098463425
## Erliansaurus.sd    -0.030486754  0.142291288  0.0764830909  0.0216695413
## Falcarius.sd        0.152164429 -0.050062330 -0.0100666293 -0.1478253355
## Guanlong.sd        -0.056929868 -0.044033694 -0.0214028000  0.0277560705
## Haplocheirus.sd    -0.004811256 -0.047816755  0.1388752675  0.0422186572
## Linhenykus.sd      -0.018723544 -0.021464548 -0.0280690126 -0.0149029653
## Manis.sd            0.063411190  0.027486521 -0.0695610446  0.0063541067
## Mononykus.sd        0.002886239 -0.013663078 -0.0069965513 -0.0178028774
## Nothronychus.sd     0.049249293 -0.074285820  0.0680861871  0.0157948637
## Puma.sd            -0.010261469  0.010402283 -0.0186635519  0.0220972205
## Shishugounykus.sd  -0.017461047 -0.002089981 -0.0206880866  0.0272811494
## Tamandua.sd        -0.116186816  0.011327966 -0.0909364842  0.0697542868
## Therizinosaurus.sd -0.072138593  0.053632549  0.0404650537  0.0012311010
## Tugulusaurus.sd    -0.009724961 -0.024924747  0.0055211630  0.0081744882
## Allosaurus.hp       0.038180508 -0.061843591  0.0150792672 -0.0294981282
## Alxasaurus.hp       0.004517803  0.027357517 -0.0033666790  0.0135160281
## Aorun.hp            0.048660400  0.020968910  0.0557281460 -0.0258518516
## Bannykus.hp         0.079684645  0.096751415 -0.0067351645 -0.0261713344
## Beipiaosaurus.hp   -0.052534781  0.034350017 -0.0842504406  0.0839180116
## Eremotherium.hp    -0.049300410 -0.030517834  0.0090333190 -0.1134700993
## Erliansaurus.hp     0.027461874 -0.009579947  0.0227435709 -0.0291868954
## Falcarius.hp       -0.031790417  0.132103498 -0.0334331640  0.0034845962
## Guanlong.hp         0.011883189  0.072233516  0.0208750795  0.0330128132
## Haplocheirus.hp     0.075832908  0.041287620 -0.0673664404 -0.0811921909
## Linhenykus.hp       0.002408639  0.065621812  0.0560241892  0.0269285488
## Manis.hp           -0.045087797  0.010182431 -0.0064308351  0.0026513587
## Mononykus.hp       -0.010712689 -0.037897198  0.0210688406 -0.0181325963
## Nothronychus.hp    -0.077755660 -0.128332363  0.0794520034  0.0005151404
## Puma.hp            -0.023553504  0.016669352 -0.0117637905  0.0111677086
## Shishugounykus.hp  -0.024790850 -0.015732372 -0.0463772056  0.0048763618
## Tamandua.hp         0.034026147  0.022201701  0.0516128875  0.0686013929
## Therizinosaurus.hp  0.071952484  0.034137345  0.0509634709 -0.0297733746
## Tugulusaurus.hp     0.005312641  0.025200492  0.0188064271 -0.0184495309
##                             PC21          PC22          PC23          PC24
## Allosaurus.p        0.0367968840  0.0133667412 -0.0318069109  0.0218937120
## Alxasaurus.p        0.0007444127  0.0101766940 -0.0110465601  0.0135246966
## Aorun.p             0.0190576860  0.0009808852  0.0021340477  0.0105605457
## Bannykus.p          0.0054620217 -0.0032003555 -0.0250634251 -0.0129954378
## Beipiaosaurus.p    -0.1328784961 -0.0323385474 -0.0414622152  0.0275790455
## Eremotherium.p     -0.0075103341 -0.0823761485  0.0034820837 -0.0007426719
## Erliansaurus.p     -0.0463408604 -0.0328376125 -0.0580997904  0.0352928692
## Falcarius.p         0.0327337723  0.0037192445 -0.0010008004 -0.1081677422
## Guanlong.p          0.0124784976 -0.0380966786  0.0233328618  0.0395947313
## Haplocheirus.p     -0.0557183466  0.0352525741  0.0240248946 -0.0060526498
## Linhenykus.p        0.0110744493 -0.0212165553  0.0032059817  0.0026317188
## Manis.p            -0.0117578754  0.0633312658 -0.0321867830  0.0053649428
## Mononykus.p        -0.0095813093  0.0203546187  0.0207887594  0.0153615327
## Nothronychus.p     -0.0377660159 -0.0358789205  0.0322523088  0.0716613407
## Puma.p             -0.0128755539 -0.0008887828  0.0311442139 -0.0274947322
## Shishugounykus.p    0.0227516734  0.0134995938  0.0098104058  0.0064410480
## Tamandua.p          0.0323689044  0.0494287910 -0.0023609567  0.0500353550
## Therizinosaurus.p  -0.1123434662 -0.0074374127 -0.0202029372 -0.0132872753
## Tugulusaurus.p      0.0006013535 -0.0155923039 -0.0060360726  0.0216520064
## Allosaurus.sd       0.0487823813  0.0264871460 -0.0592212493 -0.0146160138
## Alxasaurus.sd       0.0105090295 -0.0091144883  0.0215975056 -0.0558529888
## Aorun.sd            0.0328359303 -0.0400355946 -0.0680832849  0.0464088310
## Bannykus.sd         0.0077687293  0.0862095200  0.0767804271  0.0163376659
## Beipiaosaurus.sd   -0.0841828030  0.0498880381  0.0711565021  0.0077289236
## Eremotherium.sd    -0.0002979911  0.0140988922  0.0041034580 -0.0155030949
## Erliansaurus.sd     0.0252044725 -0.0126261327  0.0685231057 -0.0178170018
## Falcarius.sd        0.0227769292  0.0275435664  0.0129121728 -0.0324996711
## Guanlong.sd         0.0591665648 -0.0682763867  0.0611508832 -0.0352824352
## Haplocheirus.sd     0.0645806904 -0.0459219808  0.0717663315  0.0945493872
## Linhenykus.sd       0.0110573433 -0.0212062003  0.0032106867  0.0026392637
## Manis.sd            0.0255262533 -0.0363795049 -0.0143721252  0.0133658444
## Mononykus.sd       -0.0096371103  0.0203897855  0.0209664385  0.0154148330
## Nothronychus.sd    -0.0518176955 -0.0738724510 -0.0462934296 -0.0184552737
## Puma.sd             0.0164522755  0.0132817846  0.0006643622  0.0328252292
## Shishugounykus.sd   0.0203659406 -0.0163239941  0.0532700751 -0.0051142433
## Tamandua.sd         0.0522511597  0.0479945417  0.0165185473 -0.0173887872
## Therizinosaurus.sd  0.0692390918  0.0034579398  0.0136841303  0.0083374258
## Tugulusaurus.sd     0.0072069176 -0.0129525477 -0.0136524032 -0.0447512464
## Allosaurus.hp       0.0307754855  0.0384330766 -0.0528649792  0.0135064226
## Alxasaurus.hp      -0.0291955932  0.0060966437  0.0300332229 -0.0011843611
## Aorun.hp           -0.0114300764  0.0180575827 -0.0326344569 -0.0113448373
## Bannykus.hp        -0.0722730115 -0.0422362888  0.0368462907 -0.0587676831
## Beipiaosaurus.hp    0.0405313404 -0.0208417871 -0.0143053223 -0.0179265556
## Eremotherium.hp     0.0020201532  0.0116198058 -0.0252957812  0.0255596759
## Erliansaurus.hp     0.0046202382  0.0105073249  0.0324379027 -0.0256178548
## Falcarius.hp        0.0279733571  0.0660690900 -0.0545965705 -0.0087767382
## Guanlong.hp        -0.0324739743  0.0301625929  0.0051612722  0.0022533525
## Haplocheirus.hp     0.1029887331 -0.0714033790 -0.0391992478 -0.0143548531
## Linhenykus.hp      -0.0439729748  0.0200009174  0.0010175792  0.0150231178
## Manis.hp            0.0017559865  0.0236845616 -0.0026749105 -0.0027713171
## Mononykus.hp       -0.0055399351 -0.0073855632  0.0060171774  0.0006088582
## Nothronychus.hp    -0.0049177549  0.0501637172 -0.0725162375 -0.0146223530
## Puma.hp            -0.0284288308 -0.0065731622 -0.0017946589 -0.0043987452
## Shishugounykus.hp  -0.0206756159  0.0353186509  0.0023198581  0.0103296391
## Tamandua.hp        -0.0666900109 -0.0559411262 -0.0024146780 -0.0205657866
## Therizinosaurus.hp  0.0417457386 -0.0172482344 -0.0239667905 -0.0062826874
## Tugulusaurus.hp    -0.0118987609  0.0186265534 -0.0071609098 -0.0138469767
##                              PC25          PC26          PC27          PC28
## Allosaurus.p        0.00564281831  0.0050204919  0.0184211125  0.0099410571
## Alxasaurus.p        0.03105046223 -0.0156716863  0.0078327196 -0.0001596976
## Aorun.p            -0.01758108535 -0.0156602188 -0.0409235156  0.0473135840
## Bannykus.p         -0.07759029899  0.0140516869  0.0497897248 -0.0215654362
## Beipiaosaurus.p    -0.01822781071 -0.0236169068  0.0299787026  0.0196625642
## Eremotherium.p      0.01824195655  0.0321357145  0.0322132329  0.0400244627
## Erliansaurus.p     -0.02464523872 -0.0055782158 -0.0468307113  0.0023094393
## Falcarius.p        -0.02020770765 -0.0481177897  0.0012866541 -0.0190025335
## Guanlong.p          0.00003562318 -0.0085049565 -0.0382268677  0.0131088158
## Haplocheirus.p     -0.05710141577 -0.0118442125  0.0569797500  0.0559296615
## Linhenykus.p       -0.01987379651  0.0025563785 -0.0184029300 -0.0024027247
## Manis.p             0.03444219431  0.0321345886  0.0226636416 -0.0185709946
## Mononykus.p         0.00461198159  0.0276874639 -0.0102025980 -0.0285697070
## Nothronychus.p     -0.04262578741 -0.0707282619 -0.0192639351 -0.0186944821
## Puma.p             -0.00207812545  0.0109985896 -0.0008746969 -0.0055082244
## Shishugounykus.p    0.00097312752  0.0041321066  0.0113056532 -0.0020311370
## Tamandua.p         -0.00581454207 -0.0284442842  0.0203898315  0.0247537767
## Therizinosaurus.p  -0.01492654697  0.0076660272  0.0024324773 -0.0016594017
## Tugulusaurus.p     -0.01385067943  0.0001904058  0.0055191946 -0.0096589774
## Allosaurus.sd      -0.03220284537 -0.0380084972  0.0042240394 -0.0287502110
## Alxasaurus.sd      -0.01520231976 -0.0324335741 -0.0002571789  0.0465752797
## Aorun.sd            0.06275467804  0.0219589749 -0.0403268746  0.0266575531
## Bannykus.sd        -0.07712960253  0.0432219308 -0.0300034877 -0.0018718280
## Beipiaosaurus.sd    0.07360556479 -0.0231413628 -0.0022794749 -0.0159451626
## Eremotherium.sd    -0.02144085032  0.0217867460 -0.0224772550  0.0017043107
## Erliansaurus.sd     0.01771030911 -0.0146665620 -0.0205431746  0.0145207174
## Falcarius.sd        0.06698843561  0.0159743665  0.0093610276 -0.0066237146
## Guanlong.sd        -0.00944134862 -0.0323791334  0.0160552660 -0.0594201148
## Haplocheirus.sd     0.01501339258 -0.0180451763  0.0380870591 -0.0370776256
## Linhenykus.sd      -0.01987639045  0.0025525738 -0.0184025692 -0.0024001882
## Manis.sd            0.01624368664  0.0363583924  0.0090890951 -0.0033914830
## Mononykus.sd        0.00472280103  0.0274682852 -0.0099505531 -0.0284037614
## Nothronychus.sd     0.02132790090 -0.0349942604  0.0171567118 -0.0483013649
## Puma.sd            -0.00522784294 -0.0395735633  0.0080578868  0.0024733757
## Shishugounykus.sd   0.02974122616  0.0424792546 -0.0000378332  0.0403706927
## Tamandua.sd         0.03062981227  0.0112232577 -0.0093588771 -0.0065205502
## Therizinosaurus.sd -0.00235145349 -0.0054561798  0.0012270160  0.0115233913
## Tugulusaurus.sd     0.01130823472 -0.0204404270 -0.0278626948  0.0196599991
## Allosaurus.hp       0.02143774556 -0.0396793369 -0.0221861943  0.0024861141
## Alxasaurus.hp      -0.02163263450  0.0265777838 -0.0270603369 -0.0148807928
## Aorun.hp            0.00268595732 -0.0118569779 -0.0206773687 -0.0298539004
## Bannykus.hp         0.02696604876 -0.0289790260 -0.0424556632  0.0233295203
## Beipiaosaurus.hp    0.02696318873 -0.0081768908  0.0113229773 -0.0012654003
## Eremotherium.hp    -0.01361554906  0.0297821364 -0.0427679232 -0.0219871419
## Erliansaurus.hp    -0.00617200820  0.0150701395  0.0037984459 -0.0096102468
## Falcarius.hp        0.03007014347 -0.0233501677  0.0361446873 -0.0067993685
## Guanlong.hp         0.00012725037  0.0136884970  0.0147039774  0.0114402080
## Haplocheirus.hp    -0.04769253374  0.0397478934  0.0216085416  0.0303717856
## Linhenykus.hp       0.04209784952  0.0126491408  0.0426803110  0.0123186315
## Manis.hp           -0.02400974396  0.0027004552  0.0185054387  0.0027346467
## Mononykus.hp        0.04808222302 -0.0052378820  0.0220479420  0.0245753555
## Nothronychus.hp    -0.00856884841  0.0211682548 -0.0304419165 -0.0110597651
## Puma.hp             0.00431749294 -0.0094107909  0.0013490336 -0.0067869253
## Shishugounykus.hp  -0.02767415741 -0.0152146896  0.0027191798  0.0234781525
## Tamandua.hp        -0.00680707212  0.0912459232  0.0108369085 -0.0343213576
## Therizinosaurus.hp  0.00210147796  0.0092368178 -0.0043715863  0.0098453615
## Tugulusaurus.hp     0.00367465274  0.0077467534 -0.0016020226 -0.0140142372
##                             PC29          PC30          PC31          PC32
## Allosaurus.p       -0.0309438765 -0.0313284451  0.0077000854 -0.0296198479
## Alxasaurus.p       -0.0088164773 -0.0131197326  0.0045094048  0.0059425737
## Aorun.p            -0.0114471358  0.0169134526 -0.0136594092  0.0033824538
## Bannykus.p         -0.0035640623 -0.0177266027 -0.0028903514  0.0019343084
## Beipiaosaurus.p    -0.0197661055 -0.0081519140 -0.0203134258  0.0212559168
## Eremotherium.p     -0.0046586573  0.0269936242 -0.0004358998  0.0095398569
## Erliansaurus.p     -0.0299681842 -0.0251699103 -0.0162831587 -0.0200408785
## Falcarius.p         0.0354555448  0.0144458886  0.0205367197 -0.0268730533
## Guanlong.p          0.0107380139 -0.0140129433 -0.0158299787 -0.0116868280
## Haplocheirus.p      0.0090176653 -0.0119864647  0.0357888410  0.0075494984
## Linhenykus.p        0.0005648327 -0.0087649870 -0.0080982781  0.0139875215
## Manis.p            -0.0027264021  0.0117409591 -0.0096078484 -0.0029189705
## Mononykus.p        -0.0140617871  0.0068899394 -0.0012120558 -0.0082887878
## Nothronychus.p     -0.0056885633  0.0443791729 -0.0080593522 -0.0139096040
## Puma.p              0.0124453425  0.0253588256 -0.0005645877  0.0266963822
## Shishugounykus.p    0.0059200172 -0.0031942532  0.0122728951  0.0009418265
## Tamandua.p          0.0139657887 -0.0119053220  0.0040328728 -0.0052439387
## Therizinosaurus.p  -0.0096349144 -0.0030627203  0.0021348992 -0.0108410363
## Tugulusaurus.p      0.0227651887 -0.0114723306  0.0146632659  0.0113743936
## Allosaurus.sd       0.0419990734  0.0264796421 -0.0001010705  0.0504760672
## Alxasaurus.sd      -0.0447713063 -0.0011610947 -0.0134230723 -0.0133799639
## Aorun.sd           -0.0017498842  0.0248053913  0.0653716212  0.0011462488
## Bannykus.sd        -0.0130830364 -0.0309662484  0.0263076917 -0.0099619640
## Beipiaosaurus.sd    0.0331767018  0.0070065574  0.0148420780 -0.0248620125
## Eremotherium.sd     0.0459876045 -0.0221063533  0.0296957236 -0.0002919214
## Erliansaurus.sd    -0.0017429711  0.0150316371  0.0024487262  0.0278146774
## Falcarius.sd       -0.0296195490 -0.0032664713 -0.0248588999  0.0244761003
## Guanlong.sd        -0.0802456818 -0.0103122634  0.0233774391  0.0042750766
## Haplocheirus.sd     0.0295405918  0.0067242575 -0.0109416961 -0.0106833199
## Linhenykus.sd       0.0005688609 -0.0087596155 -0.0080994754  0.0139751719
## Manis.sd           -0.0054011476 -0.0206624085 -0.0132555614  0.0291709112
## Mononykus.sd       -0.0139480506  0.0071100523 -0.0014052302 -0.0084613376
## Nothronychus.sd     0.0356231085 -0.0166585915  0.0196012666 -0.0234123406
## Puma.sd             0.0125191970 -0.0086827807 -0.0156781634  0.0175784267
## Shishugounykus.sd   0.0354451561 -0.0026435753 -0.0231734992 -0.0274308911
## Tamandua.sd        -0.0232407342  0.0081178632  0.0054609436  0.0067736327
## Therizinosaurus.sd  0.0008732107  0.0068095772 -0.0118065340  0.0054421715
## Tugulusaurus.sd    -0.0120163101 -0.0132670949 -0.0055008681 -0.0236444030
## Allosaurus.hp      -0.0223431434 -0.0150844479  0.0037753073  0.0040228152
## Alxasaurus.hp       0.0068241401 -0.0002469539 -0.0308328953  0.0049610339
## Aorun.hp           -0.0147368311  0.0043420149  0.0169890350  0.0032644315
## Bannykus.hp         0.0124046947 -0.0055624278  0.0020258407  0.0208079876
## Beipiaosaurus.hp    0.0385502745 -0.0749033957 -0.0383698487  0.0006047555
## Eremotherium.hp    -0.0027457705 -0.0082947391  0.0230919649  0.0139958493
## Erliansaurus.hp     0.0081053702  0.0202931739 -0.0233761730 -0.0089582337
## Falcarius.hp       -0.0249876963  0.0205838431 -0.0202549285 -0.0259737596
## Guanlong.hp         0.0021494579 -0.0021269171  0.0168645567  0.0006541375
## Haplocheirus.hp     0.0119390955  0.0356360739  0.0074752300 -0.0287646272
## Linhenykus.hp      -0.0183462764  0.0176515281  0.0049029416  0.0058730185
## Manis.hp           -0.0001050664  0.0014998395  0.0078254903  0.0179333504
## Mononykus.hp        0.0024131033 -0.0210773884  0.0242471322  0.0095019911
## Nothronychus.hp     0.0256654024  0.0272634993 -0.0332249493 -0.0061120476
## Puma.hp             0.0043735314  0.0179679005 -0.0047246644 -0.0083290636
## Shishugounykus.hp  -0.0070789535  0.0306012560 -0.0128491649 -0.0053728641
## Tamandua.hp        -0.0076133741  0.0127322872 -0.0047954566 -0.0022666944
## Therizinosaurus.hp  0.0031021297 -0.0080322132 -0.0027939525  0.0024585070
## Tugulusaurus.hp     0.0029188509 -0.0036676503  0.0004784771 -0.0104827043
##                             PC33          PC34           PC35           PC36
## Allosaurus.p        0.0284838331 -0.0285567261  0.01127436409 -0.00645031729
## Alxasaurus.p        0.0144269473 -0.0108404439 -0.00500570529  0.01827112561
## Aorun.p            -0.0195882722 -0.0103132898 -0.00135005899 -0.01836084185
## Bannykus.p          0.0031117830  0.0116414263  0.00349214216  0.01040758974
## Beipiaosaurus.p     0.0172545258 -0.0008280636 -0.02097676291 -0.01519112284
## Eremotherium.p     -0.0194684627  0.0015824926  0.01918384515  0.00320041152
## Erliansaurus.p     -0.0118349608 -0.0064044870 -0.00040754548  0.03601845069
## Falcarius.p        -0.0150345569 -0.0148154308 -0.01431288947  0.00239883275
## Guanlong.p          0.0103198847  0.0312351254 -0.03711596500 -0.00449417622
## Haplocheirus.p     -0.0229790960 -0.0137055605 -0.02128088905 -0.00789267834
## Linhenykus.p        0.0117082085  0.0033380735  0.00610651940 -0.00382172049
## Manis.p            -0.0130614181  0.0354237699 -0.00219366024 -0.01256442245
## Mononykus.p        -0.0160661867 -0.0140565625 -0.00888895115 -0.00772818178
## Nothronychus.p     -0.0018886578 -0.0026222225  0.00004805787 -0.01128396750
## Puma.p             -0.0105305059  0.0167487773  0.01699288552  0.01566156299
## Shishugounykus.p   -0.0133480729  0.0072919812 -0.01034952143  0.01184207348
## Tamandua.p         -0.0061690884  0.0160570327  0.00808056962  0.00726716406
## Therizinosaurus.p  -0.0043542666 -0.0033197366  0.00585945361  0.00275241139
## Tugulusaurus.p     -0.0219576959  0.0152899590 -0.00307448069  0.01010839382
## Allosaurus.sd       0.0380204783 -0.0146601295 -0.02535402586  0.00569866361
## Alxasaurus.sd       0.0026382565  0.0255374717  0.00562820450  0.01036010503
## Aorun.sd           -0.0017655558  0.0135057121 -0.00702669274 -0.00001248232
## Bannykus.sd         0.0142329081  0.0071474838  0.00412148072  0.00207360929
## Beipiaosaurus.sd    0.0187848780  0.0025835600  0.01730688571 -0.00290779894
## Eremotherium.sd    -0.0039501529  0.0034251039 -0.00345851671 -0.01109822992
## Erliansaurus.sd     0.0012643602 -0.0234446210  0.01286009097 -0.01403147217
## Falcarius.sd        0.0067001265  0.0041125262 -0.01978415328 -0.00617173930
## Guanlong.sd        -0.0020492652  0.0113617016 -0.01448891661 -0.00209233082
## Haplocheirus.sd     0.0132338924 -0.0094392144  0.01008675632  0.01669927640
## Linhenykus.sd       0.0117090319  0.0033372131  0.00610651915 -0.00381849485
## Manis.sd           -0.0103099682 -0.0070595752 -0.00387456447 -0.01226806816
## Mononykus.sd       -0.0162521167 -0.0141928293 -0.00879384501 -0.00773748030
## Nothronychus.sd    -0.0078362573  0.0061445084 -0.00137794387 -0.01258645849
## Puma.sd            -0.0360470017 -0.0054337683  0.01910664838 -0.02653356812
## Shishugounykus.sd   0.0020883456 -0.0071677923 -0.03652344764  0.00205783857
## Tamandua.sd        -0.0080333471 -0.0033212278 -0.00465078326  0.00560863368
## Therizinosaurus.sd -0.0003684639 -0.0004943154 -0.00150969223  0.00124936505
## Tugulusaurus.sd     0.0250136168 -0.0091444110  0.00514596819 -0.01806518983
## Allosaurus.hp      -0.0063747743 -0.0201001268  0.01951035570 -0.00051103688
## Alxasaurus.hp       0.0121355860  0.0201981996 -0.00055238600 -0.01035618569
## Aorun.hp           -0.0043020645  0.0314104425  0.01482953463 -0.01221056274
## Bannykus.hp         0.0107795924 -0.0022914240  0.01090522979  0.02016088015
## Beipiaosaurus.hp   -0.0084622731  0.0000098691  0.01308959351 -0.00068796744
## Eremotherium.hp    -0.0061264865 -0.0235061286 -0.00938662064  0.00908810714
## Erliansaurus.hp    -0.0164245294 -0.0066332926  0.01030289112  0.01163404665
## Falcarius.hp       -0.0054314803 -0.0028056162 -0.00970324808  0.00137705329
## Guanlong.hp         0.0319070220  0.0049114670  0.01290660170 -0.01677271311
## Haplocheirus.hp     0.0221683232  0.0054369560  0.01423282840 -0.00965757150
## Linhenykus.hp       0.0177612531 -0.0010437104  0.00000301469  0.00598197065
## Manis.hp            0.0152453295  0.0063523862  0.02065592318  0.00822673439
## Mononykus.hp       -0.0037649555  0.0014124392 -0.00381033528  0.01578857957
## Nothronychus.hp     0.0017887767  0.0051728452  0.00602763340  0.00772936623
## Puma.hp             0.0045259256  0.0053513229 -0.00916440816  0.00775458055
## Shishugounykus.hp  -0.0073519329 -0.0091488148 -0.00249160969  0.01390689260
## Tamandua.hp         0.0001805210 -0.0246429117  0.00646152510 -0.00133895056
## Therizinosaurus.hp -0.0064791263  0.0044455676  0.00580450324 -0.00190311945
## Tugulusaurus.hp    -0.0078724136 -0.0104729810  0.00077759341 -0.00477486958
##                             PC37          PC38           PC39          PC40
## Allosaurus.p        0.0015210254  0.0109858542  0.01681678756 -0.0098256986
## Alxasaurus.p        0.0073564166 -0.0152227665 -0.00447313174  0.0171651699
## Aorun.p             0.0099368087  0.0131603270 -0.00238891728  0.0046990344
## Bannykus.p         -0.0046332002  0.0201842946 -0.01198130967  0.0056672025
## Beipiaosaurus.p    -0.0080795016 -0.0063011032 -0.00272114698 -0.0077245770
## Eremotherium.p     -0.0107235931 -0.0094097868  0.01062050798 -0.0034004466
## Erliansaurus.p     -0.0114443271 -0.0014107118  0.01741173030  0.0036506210
## Falcarius.p         0.0112272360  0.0119154988  0.01726055460  0.0058322000
## Guanlong.p          0.0091771850 -0.0031072062  0.01108546810  0.0143759203
## Haplocheirus.p      0.0084445049 -0.0083752025  0.00400069815 -0.0007085811
## Linhenykus.p        0.0023398259 -0.0013483815  0.00845248447 -0.0027329371
## Manis.p             0.0046081731  0.0231629995  0.00870862989 -0.0176282829
## Mononykus.p        -0.0089965524 -0.0221128549 -0.00303433762 -0.0016529467
## Nothronychus.p      0.0084301347  0.0079080922 -0.00256611255 -0.0071658018
## Puma.p             -0.0121071050 -0.0127471053  0.00347121313 -0.0146514849
## Shishugounykus.p   -0.0112001697  0.0052148761 -0.01430915984 -0.0027112907
## Tamandua.p          0.0087853519 -0.0147360791  0.00237147546 -0.0054231055
## Therizinosaurus.p  -0.0014654096 -0.0010401834 -0.00002388105  0.0015064497
## Tugulusaurus.p      0.0071760169  0.0010716725  0.00408427054 -0.0071742803
## Allosaurus.sd      -0.0172189273 -0.0063279942 -0.00268985962 -0.0008688276
## Alxasaurus.sd      -0.0016281265 -0.0022020413 -0.01638461304  0.0003777225
## Aorun.sd            0.0053684556 -0.0013887251 -0.00464544154  0.0038366245
## Bannykus.sd        -0.0069926623  0.0007441549 -0.01151614984  0.0022600554
## Beipiaosaurus.sd    0.0016241576 -0.0018606968  0.00313959569  0.0052425395
## Eremotherium.sd    -0.0028860768 -0.0122320728  0.01555691072 -0.0006191340
## Erliansaurus.sd    -0.0007391998 -0.0078243179  0.00146886119 -0.0061755735
## Falcarius.sd       -0.0042310746  0.0097124784  0.01362176086  0.0009054272
## Guanlong.sd         0.0137292953 -0.0070620166  0.01046673867 -0.0041735886
## Haplocheirus.sd    -0.0119684603  0.0089543436  0.00180216533  0.0019774698
## Linhenykus.sd       0.0023407777 -0.0013466352  0.00845816483 -0.0027252692
## Manis.sd            0.0175942741  0.0016825732 -0.01257888655  0.0187696497
## Mononykus.sd       -0.0092693123 -0.0220638455 -0.00318217004 -0.0017049240
## Nothronychus.sd     0.0074338415 -0.0025280719 -0.01704365494 -0.0028826488
## Puma.sd            -0.0178936948  0.0180119233  0.00170938075  0.0152837143
## Shishugounykus.sd  -0.0144663783  0.0097505245 -0.01638617860 -0.0076191827
## Tamandua.sd         0.0015640879  0.0062728031 -0.00137675427  0.0005262447
## Therizinosaurus.sd  0.0021893895  0.0001860945  0.00219132314 -0.0009856353
## Tugulusaurus.sd    -0.0240303187  0.0005413950 -0.00373391056  0.0010468609
## Allosaurus.hp       0.0162686590  0.0006643274 -0.01585047316 -0.0118209102
## Alxasaurus.hp       0.0128487442 -0.0016024736 -0.00371245345 -0.0066476896
## Aorun.hp           -0.0367951206 -0.0020569702 -0.00222624110  0.0115938095
## Bannykus.hp         0.0001543497  0.0115956933 -0.01004910168 -0.0038025370
## Beipiaosaurus.hp    0.0058275521 -0.0141167302  0.00224723936 -0.0084847966
## Eremotherium.hp     0.0040209991  0.0158071498 -0.00936324026 -0.0129432845
## Erliansaurus.hp     0.0173360678 -0.0045530870 -0.00872497163  0.0104272458
## Falcarius.hp       -0.0104555887 -0.0117018596 -0.00724349246  0.0002853863
## Guanlong.hp         0.0128575588  0.0033110904  0.01168381045  0.0038652622
## Haplocheirus.hp    -0.0019190700 -0.0066868987 -0.00428847596 -0.0031922485
## Linhenykus.hp       0.0179966537 -0.0024489638 -0.00659199561  0.0042399325
## Manis.hp            0.0089682825  0.0023982168 -0.00448240215  0.0098745075
## Mononykus.hp       -0.0148123519  0.0147245184  0.01004618883  0.0016239578
## Nothronychus.hp     0.0187460997 -0.0056601504  0.00959678264  0.0036499667
## Puma.hp             0.0103360971  0.0072660369 -0.00684097197 -0.0029863677
## Shishugounykus.hp  -0.0067170331  0.0013444683  0.01771744637  0.0026482967
## Tamandua.hp        -0.0010623944  0.0088070713  0.00538234032  0.0059196363
## Therizinosaurus.hp  0.0027191291 -0.0028565382 -0.00235501788 -0.0004268180
## Tugulusaurus.hp    -0.0071915019 -0.0030470078 -0.00660807627  0.0016079611
##                              PC41          PC42           PC43           PC44
## Allosaurus.p        0.01084731055  0.0048645909 -0.00794029757 -0.00354076464
## Alxasaurus.p       -0.00510870241 -0.0138824625 -0.00891024623  0.00107335491
## Aorun.p             0.00905497173 -0.0064534645  0.00174296931  0.01058580614
## Bannykus.p         -0.00003875028 -0.0051551209  0.00182079757  0.00200024528
## Beipiaosaurus.p     0.00527733044 -0.0031141679 -0.00367607552 -0.00067407935
## Eremotherium.p     -0.01928994222  0.0089843269  0.00254942641  0.00483173568
## Erliansaurus.p     -0.00582922444  0.0001599354  0.00027847564  0.00915431939
## Falcarius.p        -0.00940139223 -0.0048079153  0.00005167959  0.00355291303
## Guanlong.p          0.00008346964  0.0032413666  0.00029284583 -0.00503136910
## Haplocheirus.p     -0.00267037557 -0.0051766889 -0.00242085481  0.00034545612
## Linhenykus.p       -0.00394693104  0.0011141396  0.00008608954 -0.00086800341
## Manis.p            -0.00351406440 -0.0056836795 -0.00540180537  0.00025358585
## Mononykus.p        -0.00069696721 -0.0038497671  0.00623821349  0.00266377849
## Nothronychus.p     -0.00011117524  0.0003782620  0.00054785518 -0.00155019122
## Puma.p              0.01481626190 -0.0184571782 -0.01113740169  0.00220917037
## Shishugounykus.p    0.01281954356  0.0072745917 -0.00101960130  0.00646365439
## Tamandua.p         -0.00184665713 -0.0028533382 -0.00156433555  0.00084306752
## Therizinosaurus.p  -0.00091171444  0.0008412247  0.00082910769 -0.00132547112
## Tugulusaurus.p      0.00612606322  0.0099657153  0.00316489339 -0.00929063895
## Allosaurus.sd      -0.00486376418  0.0042202465  0.00170633312 -0.00027177749
## Alxasaurus.sd       0.00020037975  0.0004152458  0.00702292310 -0.00700091797
## Aorun.sd           -0.00143096787 -0.0044669652 -0.00141351237 -0.00132724302
## Bannykus.sd        -0.00428877742  0.0028902488 -0.00023555945 -0.00039890804
## Beipiaosaurus.sd    0.00107930738  0.0009903793  0.00030224801 -0.00010453685
## Eremotherium.sd     0.00302305944  0.0007377906 -0.00234338580  0.00238131225
## Erliansaurus.sd     0.00312889215  0.0112504856  0.00350704699 -0.00493622529
## Falcarius.sd       -0.00198754097  0.0011801293  0.00214716475  0.00624651392
## Guanlong.sd         0.00405234702  0.0022547339 -0.00427504099  0.00186823819
## Haplocheirus.sd     0.00095771269 -0.0085090169  0.00261371509  0.00227833195
## Linhenykus.sd      -0.00394150318  0.0011181408  0.00007890431 -0.00087135189
## Manis.sd            0.01346081712 -0.0040995043  0.00079319402 -0.00256008058
## Mononykus.sd       -0.00049390817 -0.0038914372  0.00631119017  0.00269234327
## Nothronychus.sd     0.00216416747  0.0049183286  0.00058233300  0.00012528785
## Puma.sd            -0.00923825063 -0.0015710767 -0.00583371600 -0.00224653539
## Shishugounykus.sd   0.00204856108  0.0086443421 -0.00485474828  0.00601649127
## Tamandua.sd        -0.00492724910  0.0003798109  0.00000307887 -0.00121464094
## Therizinosaurus.sd  0.00213919083 -0.0017038029 -0.00119558737 -0.00003743176
## Tugulusaurus.sd    -0.00436554282 -0.0095121045 -0.00920390138 -0.00429106442
## Allosaurus.hp      -0.00238694985  0.0007296395  0.00531080029  0.00442206480
## Alxasaurus.hp      -0.01885394053 -0.0092009170  0.00616434135 -0.00420773738
## Aorun.hp            0.00345735161  0.0115357875 -0.00556323670  0.00245076099
## Bannykus.hp         0.00504517952 -0.0027905321  0.00073521699  0.00098518282
## Beipiaosaurus.hp   -0.00199064615  0.0048486333 -0.00072156401  0.00117410664
## Eremotherium.hp    -0.00939030549  0.0005406645 -0.00539681038 -0.00646686675
## Erliansaurus.hp     0.00088731497  0.0025030610 -0.01211673121 -0.00723995478
## Falcarius.hp        0.00121551903 -0.0016224178  0.00790864334 -0.00482480655
## Guanlong.hp         0.00872355225 -0.0020419783  0.01040429328  0.00760864398
## Haplocheirus.hp    -0.00034607357 -0.0039097298  0.00079296579 -0.00110486997
## Linhenykus.hp      -0.01299477025  0.0103666400 -0.01180010892  0.00032983300
## Manis.hp           -0.00236250918  0.0094635574  0.00439673508  0.00770070999
## Mononykus.hp        0.00056049525 -0.0086624234  0.01519352207 -0.01125551991
## Nothronychus.hp     0.00979328515  0.0018433594  0.00083389080 -0.00232626064
## Puma.hp            -0.00019097287  0.0031842354  0.00371705851  0.00798371285
## Shishugounykus.hp   0.00999888208  0.0109784864 -0.00117605464 -0.00728652759
## Tamandua.hp         0.00355565737 -0.0008969915  0.00275729122 -0.00323032884
## Therizinosaurus.hp  0.00215848832  0.0009860932  0.00045229771  0.00005352554
## Tugulusaurus.hp     0.00074445735 -0.0004915122  0.00686303403 -0.00281004265
##                               PC45           PC46           PC47          PC48
## Allosaurus.p        0.006624542271 -0.00652536827 -0.00060968251 -0.0015574121
## Alxasaurus.p        0.003903092518 -0.00547983373  0.00012935161  0.0011076212
## Aorun.p             0.001840228855  0.00359063872 -0.00667312806 -0.0020387324
## Bannykus.p          0.004283193437 -0.00113193078  0.00286606601 -0.0006119127
## Beipiaosaurus.p    -0.001868381319 -0.00149103307  0.00035838637  0.0012698687
## Eremotherium.p     -0.002113236858 -0.00002893137  0.00012558794  0.0003870840
## Erliansaurus.p      0.001011953020  0.00236502901 -0.00059504270  0.0005263996
## Falcarius.p         0.006243130554 -0.00020603049 -0.00076387331  0.0013734821
## Guanlong.p          0.000908258998 -0.00315962299  0.00021971980 -0.0015523949
## Haplocheirus.p     -0.001552862971 -0.00116929940  0.00068781135 -0.0006698116
## Linhenykus.p        0.002055674097  0.00094286765  0.00185930802 -0.0010142143
## Manis.p            -0.004013801981 -0.00402827657  0.00109057374  0.0014420370
## Mononykus.p         0.002194458409 -0.00166928084  0.00264771443  0.0011921524
## Nothronychus.p     -0.001335375788  0.00019602283 -0.00263950866  0.0003568192
## Puma.p              0.005713947409  0.00048112444 -0.00384634632 -0.0003260397
## Shishugounykus.p   -0.000975993298  0.00294397182  0.00479684986 -0.0031015776
## Tamandua.p         -0.002813028382 -0.00009323126 -0.00089294144  0.0010658563
## Therizinosaurus.p   0.000634076909  0.00018217203 -0.00033307675 -0.0001017420
## Tugulusaurus.p      0.009504944761  0.00457270409 -0.00042610803  0.0011630569
## Allosaurus.sd      -0.000297333926  0.00105558070 -0.00015539026 -0.0000217908
## Alxasaurus.sd       0.009930818896 -0.00067137352  0.00183368044  0.0017086353
## Aorun.sd            0.000278644660 -0.00171871062  0.00257325282  0.0012307736
## Bannykus.sd        -0.002977794042  0.00092776903 -0.00104793295 -0.0010913913
## Beipiaosaurus.sd    0.003731217086  0.00043600138 -0.00064610199 -0.0009178709
## Eremotherium.sd    -0.004158798701  0.00052027544  0.00102187545 -0.0041609965
## Erliansaurus.sd     0.003523750494 -0.00428833158 -0.00023441654 -0.0007176123
## Falcarius.sd        0.000822938763  0.00065109687  0.00049988216  0.0004450297
## Guanlong.sd        -0.006283668177  0.00128648648  0.00007883498 -0.0003779101
## Haplocheirus.sd    -0.000477292164  0.00154023577  0.00118904726  0.0003429699
## Linhenykus.sd       0.002054743403  0.00093954521  0.00185579528 -0.0010112711
## Manis.sd           -0.000286939551  0.00177228733 -0.00175680491  0.0009659593
## Mononykus.sd        0.002359968893 -0.00162736545  0.00253804874  0.0012377750
## Nothronychus.sd    -0.001149579183 -0.00050853502 -0.00117796382  0.0009966163
## Puma.sd             0.002766889862 -0.00131702939  0.00480421050 -0.0008354926
## Shishugounykus.sd   0.001732435276 -0.00148789966 -0.00088244390  0.0023254153
## Tamandua.sd        -0.000803385900  0.00038876758 -0.00026489026  0.0017089169
## Therizinosaurus.sd  0.000006641672 -0.00016526925  0.00012363884  0.0001291019
## Tugulusaurus.sd    -0.007583747669  0.00594150584  0.00033962864  0.0010004554
## Allosaurus.hp      -0.002342752852 -0.00027323849  0.00382502839 -0.0019013375
## Alxasaurus.hp       0.001035268646 -0.00071247734 -0.00066382204 -0.0013761703
## Aorun.hp            0.001500421560 -0.00098822420 -0.00320698074  0.0003537375
## Bannykus.hp        -0.003821996929 -0.00246020421  0.00434547999 -0.0009618883
## Beipiaosaurus.hp   -0.001791880008  0.00150310870 -0.00235585010  0.0012657692
## Eremotherium.hp     0.001120696668  0.00214381867 -0.00303043092  0.0030128067
## Erliansaurus.hp    -0.010017609411  0.00090106737  0.00157689723  0.0016113834
## Falcarius.hp       -0.000650316214  0.00208261424 -0.00248087977 -0.0036354831
## Guanlong.hp         0.000697058636  0.00721608352  0.00349576556  0.0058231533
## Haplocheirus.hp    -0.001342652927 -0.00025577388  0.00021936914 -0.0004796775
## Linhenykus.hp       0.005795757953  0.00780352388 -0.00144769894 -0.0034822539
## Manis.hp           -0.005017118129 -0.00734601578 -0.00622795436  0.0022919451
## Mononykus.hp       -0.007494564964  0.00078312715 -0.00394812654 -0.0022108982
## Nothronychus.hp    -0.000715906908 -0.00042022189  0.00197642845 -0.0012494929
## Puma.hp            -0.004321931344 -0.00274611636  0.00053592719 -0.0030747956
## Shishugounykus.hp  -0.005858606477  0.00024411134  0.00223023977  0.0030861608
## Tamandua.hp         0.000864368721 -0.00055769461 -0.00038451190 -0.0025276493
## Therizinosaurus.hp -0.000333909713 -0.00002247945 -0.00009035045 -0.0004494744
## Tugulusaurus.hp    -0.000738656642 -0.00086173760 -0.00306214181  0.0020363120
##                              PC49                  PC50
## Allosaurus.p       -0.00133621966  0.000000000135177577
## Alxasaurus.p        0.00161825630 -0.000000000290330524
## Aorun.p             0.00061382198 -0.000000000052280458
## Bannykus.p         -0.00066734813 -0.000000000121933255
## Beipiaosaurus.p    -0.00011606628  0.000000000079589182
## Eremotherium.p     -0.00014902085  0.000000000009106028
## Erliansaurus.p     -0.00028506548  0.000000000138747708
## Falcarius.p        -0.00040225684 -0.000000000056606012
## Guanlong.p          0.00015202812  0.000000000219472648
## Haplocheirus.p     -0.00112824541 -0.000000000044682896
## Linhenykus.p       -0.00069688425 -0.000000000077057249
## Manis.p             0.00144032322 -0.000000000043578391
## Mononykus.p         0.00046562666  0.000000000027085043
## Nothronychus.p      0.00022749284 -0.000000000012016700
## Puma.p             -0.00061033348  0.000000000151150925
## Shishugounykus.p    0.00065374229  0.000000000106496936
## Tamandua.p         -0.00010278479 -0.000000000045965010
## Therizinosaurus.p   0.00007532446  0.000000000028470337
## Tugulusaurus.p      0.00008334879  0.000000000080056475
## Allosaurus.sd      -0.00014221839  0.000000000101712125
## Alxasaurus.sd       0.00030066452  0.000000000040611486
## Aorun.sd           -0.00107090855  0.000000000037801373
## Bannykus.sd         0.00119147837  0.000000000016238871
## Beipiaosaurus.sd    0.00023993736 -0.000000000012170917
## Eremotherium.sd     0.00014717912 -0.000000000033314049
## Erliansaurus.sd     0.00016244468 -0.000000000056803950
## Falcarius.sd        0.00008474676  0.000000000040838166
## Guanlong.sd         0.00061778825  0.000000000011697546
## Haplocheirus.sd    -0.00056144361 -0.000000000073120230
## Linhenykus.sd      -0.00070237795  0.000000000063162142
## Manis.sd           -0.00041461365 -0.000000000202428282
## Mononykus.sd        0.00062024216  0.000000000078943671
## Nothronychus.sd     0.00062703422  0.000000000035276476
## Puma.sd             0.00125173490  0.000000000264385444
## Shishugounykus.sd  -0.00097919035 -0.000000000030978428
## Tamandua.sd        -0.00033197235  0.000000000045059671
## Therizinosaurus.sd -0.00025480157 -0.000000000039193981
## Tugulusaurus.sd     0.00016533523  0.000000000037573611
## Allosaurus.hp      -0.00150100151 -0.000000000132106492
## Alxasaurus.hp      -0.00185298466 -0.000000000201961559
## Aorun.hp           -0.00196346515 -0.000000000352212495
## Bannykus.hp         0.00118567826  0.000000000040122503
## Beipiaosaurus.hp    0.00086210907 -0.000000000129678365
## Eremotherium.hp     0.00102688794  0.000000000011948151
## Erliansaurus.hp    -0.00265045427  0.000000000302141381
## Falcarius.hp       -0.00009792542  0.000000000168963135
## Guanlong.hp        -0.00055803005  0.000000000114435197
## Haplocheirus.hp     0.00183303587 -0.000000000026489616
## Linhenykus.hp       0.00096491743 -0.000000000036669529
## Manis.hp            0.00055217252  0.000000000342683978
## Mononykus.hp       -0.00015017636  0.000000000005811199
## Nothronychus.hp     0.00086903897  0.000000000046270335
## Puma.hp            -0.00056571005 -0.000000000186259924
## Shishugounykus.hp   0.00100915078 -0.000000000497279343
## Tamandua.hp         0.00014647904 -0.000000000037566228
## Therizinosaurus.hp  0.00011083891 -0.000000000041418924
## Tugulusaurus.hp    -0.00000735995  0.000000000053075294
```

```
Coord.p<-ind.coord[1:19, 1:2]
Coord.s<-ind.coord[20:38, 1:2]
Coord.hp<-ind.coord[39:57, 1:2]
Coord<-ind.coord[1:57, 1:2]
Coord.p
```

```
##                           PC1        PC2
## Allosaurus.p      -3.34322735  1.5024415
## Alxasaurus.p      -4.25547189  3.8567721
## Aorun.p           -2.53687163  0.1436204
## Bannykus.p         0.95231302 -2.6743476
## Beipiaosaurus.p    3.81786982 -4.0764147
## Eremotherium.p    -0.86870155 -1.4597797
## Erliansaurus.p    -1.31118643 -2.7903685
## Falcarius.p       -0.05572564 -1.9047215
## Guanlong.p        -2.45530339  0.2594165
## Haplocheirus.p    -1.30671697 -1.4332262
## Linhenykus.p      -4.41812298  4.2021227
## Manis.p           -2.90212515 -0.3406726
## Mononykus.p       -3.56290532  1.4871996
## Nothronychus.p     1.75663273 -3.5751840
## Puma.p            -3.33456905  1.8648867
## Shishugounykus.p  -3.94190947  3.1544965
## Tamandua.p         3.45214163 -6.2439935
## Therizinosaurus.p 21.89583870  6.1195515
## Tugulusaurus.p    -3.13483590  2.0373422
```

```
Coord.s
```

```
##                           PC1        PC2
## Allosaurus.sd       2.4086445 -2.2084409
## Alxasaurus.sd      -0.9553253 -3.3378570
## Aorun.sd            2.4815255 -2.3874001
## Bannykus.sd         4.7883633 -3.8180150
## Beipiaosaurus.sd    4.8798236 -3.9494467
## Eremotherium.sd    -0.3050740 -1.1973785
## Erliansaurus.sd     0.1382769 -2.3141258
## Falcarius.sd        4.2261804 -3.9608883
## Guanlong.sd        -0.3737679  0.4249703
## Haplocheirus.sd     2.1163420 -1.4224292
## Linhenykus.sd      -4.4181251  4.2020874
## Manis.sd           -1.8587151 -2.9528135
## Mononykus.sd       -3.5629089  1.4872153
## Nothronychus.sd     0.3103920  0.9309279
## Puma.sd            -2.1327064  2.1343638
## Shishugounykus.sd  -1.1599824  0.7198907
## Tamandua.sd         9.7469621 -4.0015429
## Therizinosaurus.sd 21.2672994  7.3602389
## Tugulusaurus.sd    -2.1512056  1.8696094
```

```
Coord.hp
```

```
##                           PC1        PC2
## Allosaurus.hp      -2.0475045 -1.4825623
## Alxasaurus.hp      -3.7977112  2.9332823
## Aorun.hp           -2.7466871 -1.5153310
## Bannykus.hp        -2.0891062 -1.9456923
## Beipiaosaurus.hp   -0.9174517 -3.2212909
## Eremotherium.hp    -3.2354904  0.4168465
## Erliansaurus.hp    -3.4141241 -0.6467804
## Falcarius.hp       -2.6297254 -0.9299619
## Guanlong.hp        -4.1030430  2.5541163
## Haplocheirus.hp    -2.6635726 -1.5746950
## Linhenykus.hp      -4.1621863  2.5353954
## Manis.hp           -4.2384906  3.1852543
## Mononykus.hp       -4.0310255  2.4064425
## Nothronychus.hp     1.1542818 -2.6160904
## Puma.hp            -4.5050087  5.7497375
## Shishugounykus.hp  -4.5582694  2.9242360
## Tamandua.hp         2.1280086 -6.3318209
## Therizinosaurus.hp 20.7790085  3.4247211
## Tugulusaurus.hp    -4.8150244  6.4260867
```

```
Coord
```

```
##                            PC1        PC2
## Allosaurus.p       -3.34322735  1.5024415
## Alxasaurus.p       -4.25547189  3.8567721
## Aorun.p            -2.53687163  0.1436204
## Bannykus.p          0.95231302 -2.6743476
## Beipiaosaurus.p     3.81786982 -4.0764147
## Eremotherium.p     -0.86870155 -1.4597797
## Erliansaurus.p     -1.31118643 -2.7903685
## Falcarius.p        -0.05572564 -1.9047215
## Guanlong.p         -2.45530339  0.2594165
## Haplocheirus.p     -1.30671697 -1.4332262
## Linhenykus.p       -4.41812298  4.2021227
## Manis.p            -2.90212515 -0.3406726
## Mononykus.p        -3.56290532  1.4871996
## Nothronychus.p      1.75663273 -3.5751840
## Puma.p             -3.33456905  1.8648867
## Shishugounykus.p   -3.94190947  3.1544965
## Tamandua.p          3.45214163 -6.2439935
## Therizinosaurus.p  21.89583870  6.1195515
## Tugulusaurus.p     -3.13483590  2.0373422
## Allosaurus.sd       2.40864451 -2.2084409
## Alxasaurus.sd      -0.95532526 -3.3378570
## Aorun.sd            2.48152554 -2.3874001
## Bannykus.sd         4.78836331 -3.8180150
## Beipiaosaurus.sd    4.87982360 -3.9494467
## Eremotherium.sd    -0.30507397 -1.1973785
## Erliansaurus.sd     0.13827691 -2.3141258
## Falcarius.sd        4.22618045 -3.9608883
## Guanlong.sd        -0.37376791  0.4249703
## Haplocheirus.sd     2.11634198 -1.4224292
## Linhenykus.sd      -4.41812506  4.2020874
## Manis.sd           -1.85871510 -2.9528135
## Mononykus.sd       -3.56290888  1.4872153
## Nothronychus.sd     0.31039204  0.9309279
## Puma.sd            -2.13270643  2.1343638
## Shishugounykus.sd  -1.15998242  0.7198907
## Tamandua.sd         9.74696208 -4.0015429
## Therizinosaurus.sd 21.26729943  7.3602389
## Tugulusaurus.sd    -2.15120564  1.8696094
## Allosaurus.hp      -2.04750450 -1.4825623
## Alxasaurus.hp      -3.79771123  2.9332823
## Aorun.hp           -2.74668706 -1.5153310
## Bannykus.hp        -2.08910621 -1.9456923
## Beipiaosaurus.hp   -0.91745173 -3.2212909
## Eremotherium.hp    -3.23549043  0.4168465
## Erliansaurus.hp    -3.41412410 -0.6467804
## Falcarius.hp       -2.62972535 -0.9299619
## Guanlong.hp        -4.10304303  2.5541163
## Haplocheirus.hp    -2.66357265 -1.5746950
## Linhenykus.hp      -4.16218631  2.5353954
## Manis.hp           -4.23849061  3.1852543
## Mononykus.hp       -4.03102550  2.4064425
## Nothronychus.hp     1.15428179 -2.6160904
## Puma.hp            -4.50500872  5.7497375
## Shishugounykus.hp  -4.55826939  2.9242360
## Tamandua.hp         2.12800856 -6.3318209
## Therizinosaurus.hp 20.77900846  3.4247211
## Tugulusaurus.hp    -4.81502435  6.4260867
```

```
options(scipen = 10000)

##Allosaurus###
N=1
x<-Coord.p[N,1]
y<-Coord.p[N,2]
xa<-Coord.s[N,1]
ya<-Coord.s[N,2]
xb<-Coord.hp[N,1]
yb<-Coord.hp[N,2]

dS1<-sqrt((x-xa)^2+(y-ya)^2)
dHP1<-sqrt((x-xb)^2+(y-yb)^2)
dTD1<-sqrt((xa-xb)^2+(ya-yb)^2)
  
##Alxasaurus###
N=2
x<-Coord.p[N,1]
y<-Coord.p[N,2]
xa<-Coord.s[N,1]
ya<-Coord.s[N,2]
xb<-Coord.hp[N,1]
yb<-Coord.hp[N,2]

dS2<-sqrt((x-xa)^2+(y-ya)^2)
dHP2<-sqrt((x-xb)^2+(y-yb)^2)
dTD2<-sqrt((xa-xb)^2+(ya-yb)^2)

##Aorun###
N=3
x<-Coord.p[N,1]
y<-Coord.p[N,2]
xa<-Coord.s[N,1]
ya<-Coord.s[N,2]
xb<-Coord.hp[N,1]
yb<-Coord.hp[N,2]

dS3<-sqrt((x-xa)^2+(y-ya)^2)
dHP3<-sqrt((x-xb)^2+(y-yb)^2)
dTD3<-sqrt((xa-xb)^2+(ya-yb)^2)
##Bannykus###
N=4
x<-Coord.p[N,1]
y<-Coord.p[N,2]
xa<-Coord.s[N,1]
ya<-Coord.s[N,2]
xb<-Coord.hp[N,1]
yb<-Coord.hp[N,2]

dS4<-sqrt((x-xa)^2+(y-ya)^2)
dHP4<-sqrt((x-xb)^2+(y-yb)^2)
dTD4<-sqrt((xa-xb)^2+(ya-yb)^2)

##Beipiaosaurus###
N=5
x<-Coord.p[N,1]
y<-Coord.p[N,2]
xa<-Coord.s[N,1]
ya<-Coord.s[N,2]
xb<-Coord.hp[N,1]
yb<-Coord.hp[N,2]

dS5<-sqrt((x-xa)^2+(y-ya)^2)
dHP5<-sqrt((x-xb)^2+(y-yb)^2)
dTD5<-sqrt((xa-xb)^2+(ya-yb)^2)

##Eremontherium###
N=6
x<-Coord.p[N,1]
y<-Coord.p[N,2]
xa<-Coord.s[N,1]
ya<-Coord.s[N,2]
xb<-Coord.hp[N,1]
yb<-Coord.hp[N,2]

dS6<-sqrt((x-xa)^2+(y-ya)^2)
dHP6<-sqrt((x-xb)^2+(y-yb)^2)
dTD6<-sqrt((xa-xb)^2+(ya-yb)^2)

##Erliansaurus###
N=7
x<-Coord.p[N,1]
y<-Coord.p[N,2]
xa<-Coord.s[N,1]
ya<-Coord.s[N,2]
xb<-Coord.hp[N,1]
yb<-Coord.hp[N,2]

dS7<-sqrt((x-xa)^2+(y-ya)^2)
dHP7<-sqrt((x-xb)^2+(y-yb)^2)
dTD7<-sqrt((xa-xb)^2+(ya-yb)^2)

##Falcarius###
N=8
x<-Coord.p[N,1]
y<-Coord.p[N,2]
xa<-Coord.s[N,1]
ya<-Coord.s[N,2]
xb<-Coord.hp[N,1]
yb<-Coord.hp[N,2]

dS8<-sqrt((x-xa)^2+(y-ya)^2)
dHP8<-sqrt((x-xb)^2+(y-yb)^2)
dTD8<-sqrt((xa-xb)^2+(ya-yb)^2)

##Guanlong###

N=9
x<-Coord.p[N,1]
y<-Coord.p[N,2]
xa<-Coord.s[N,1]
ya<-Coord.s[N,2]
xb<-Coord.hp[N,1]
yb<-Coord.hp[N,2]

dS9<-sqrt((x-xa)^2+(y-ya)^2)
dHP9<-sqrt((x-xb)^2+(y-yb)^2)
dTD9<-sqrt((xa-xb)^2+(ya-yb)^2)
##Haplocheirus###
N=10
x<-Coord.p[N,1]
y<-Coord.p[N,2]
xa<-Coord.s[N,1]
ya<-Coord.s[N,2]
xb<-Coord.hp[N,1]
yb<-Coord.hp[N,2]

dS10<-sqrt((x-xa)^2+(y-ya)^2)
dHP10<-sqrt((x-xb)^2+(y-yb)^2)
dTD10<-sqrt((xa-xb)^2+(ya-yb)^2)

##Linhenykus###
N=11
x<-Coord.p[N,1]
y<-Coord.p[N,2]
xa<-Coord.s[N,1]
ya<-Coord.s[N,2]
xb<-Coord.hp[N,1]
yb<-Coord.hp[N,2]

dS11<-sqrt((x-xa)^2+(y-ya)^2)
dHP11<-sqrt((x-xb)^2+(y-yb)^2)
dTD11<-sqrt((xa-xb)^2+(ya-yb)^2)
##Manis###

N=12
x<-Coord.p[N,1]
y<-Coord.p[N,2]
xa<-Coord.s[N,1]
ya<-Coord.s[N,2]
xb<-Coord.hp[N,1]
yb<-Coord.hp[N,2]

dS12<-sqrt((x-xa)^2+(y-ya)^2)
dHP12<-sqrt((x-xb)^2+(y-yb)^2)
dTD12<-sqrt((xa-xb)^2+(ya-yb)^2)
##Mononykus###

N=13
x<-Coord.p[N,1]
y<-Coord.p[N,2]
xa<-Coord.s[N,1]
ya<-Coord.s[N,2]
xb<-Coord.hp[N,1]
yb<-Coord.hp[N,2]

dS13<-sqrt((x-xa)^2+(y-ya)^2)
dHP13<-sqrt((x-xb)^2+(y-yb)^2)
dTD13<-sqrt((xa-xb)^2+(ya-yb)^2)
##Nothronychus###

N=14
x<-Coord.p[N,1]
y<-Coord.p[N,2]
xa<-Coord.s[N,1]
ya<-Coord.s[N,2]
xb<-Coord.hp[N,1]
yb<-Coord.hp[N,2]

dS14<-sqrt((x-xa)^2+(y-ya)^2)
dHP14<-sqrt((x-xb)^2+(y-yb)^2)
dTD14<-sqrt((xa-xb)^2+(ya-yb)^2)
##Puma###

N=15
x<-Coord.p[N,1]
y<-Coord.p[N,2]
xa<-Coord.s[N,1]
ya<-Coord.s[N,2]
xb<-Coord.hp[N,1]
yb<-Coord.hp[N,2]

dS15<-sqrt((x-xa)^2+(y-ya)^2)
dHP15<-sqrt((x-xb)^2+(y-yb)^2)
dTD15<-sqrt((xa-xb)^2+(ya-yb)^2)

##Shishugounykus###
N=16
x<-Coord.p[N,1]
y<-Coord.p[N,2]
xa<-Coord.s[N,1]
ya<-Coord.s[N,2]
xb<-Coord.hp[N,1]
yb<-Coord.hp[N,2]

dS16<-sqrt((x-xa)^2+(y-ya)^2)
dHP16<-sqrt((x-xb)^2+(y-yb)^2)
dTD16<-sqrt((xa-xb)^2+(ya-yb)^2)

##Tamandua###
N=17
x<-Coord.p[N,1]
y<-Coord.p[N,2]
xa<-Coord.s[N,1]
ya<-Coord.s[N,2]
xb<-Coord.hp[N,1]
yb<-Coord.hp[N,2]

dS17<-sqrt((x-xa)^2+(y-ya)^2)
dHP17<-sqrt((x-xb)^2+(y-yb)^2)
dTD17<-sqrt((xa-xb)^2+(ya-yb)^2)
##Therizinosaurus###
N=18
x<-Coord.p[N,1]
y<-Coord.p[N,2]
xa<-Coord.s[N,1]
ya<-Coord.s[N,2]
xb<-Coord.hp[N,1]
yb<-Coord.hp[N,2]

dS18<-sqrt((x-xa)^2+(y-ya)^2)
dHP18<-sqrt((x-xb)^2+(y-yb)^2)
dTD18<-sqrt((xa-xb)^2+(ya-yb)^2)
##Tugulusaurus###
N=19
x<-Coord.p[N,1]
y<-Coord.p[N,2]
xa<-Coord.s[N,1]
ya<-Coord.s[N,2]
xb<-Coord.hp[N,1]
yb<-Coord.hp[N,2]

dS19<-sqrt((x-xa)^2+(y-ya)^2)
dHP19<-sqrt((x-xb)^2+(y-yb)^2)
dTD19<-sqrt((xa-xb)^2+(ya-yb)^2)
```

## 6.2 made dataset

```
matrix.distance.Scratchdigging<-c(Allosaurus=dS1, Alxasaurus=dS2,Aorun=dS3,
                             Bannykus=dS4,Beipiaosaurus=dS5,Eremotherium=dS6,Erliansaurus=dS7,
                             Falcarius=dS8,Guanlong=-dS9,Haplocheirus=dS10,
                             Linhenykus=dS11,Manis=dS12,Mononykus=dS13,Nothronychus=dS14,Puma=-dS15,
                             Shishugounykus=dS16,Tamandua=dS17,Therizinosaurus=dS18,
                             Tugulusaurus=-dS19)
write.csv(matrix.distance.Scratchdigging, file = 'matrix.distance.Scratch.csv')

matrix.distance.HookandPull<-c(Allosaurus=dHP1, Alxasaurus=dHP2,Aorun=dHP3,
                               Bannykus=-dHP4,Beipiaosaurus=-dHP5,Eremotherium=-dHP6,Erliansaurus=-dHP7,
                               Falcarius=-dHP8,Guanlong=-dHP9,Haplocheirus=-dHP10,
                               Linhenykus=dHP11,Manis=-dHP12,Mononykus=dHP13,Nothronychus=-dHP14,Puma=-dHP15,
                               Shishugounykus=dHP16,Tamandua=-dHP17,Therizinosaurus=-dHP18,
                               Tugulusaurus=-dHP19)
write.csv(matrix.distance.HookandPull, file = 'matrix.distance.HookandPull.csv')

matrix.distance.twodistance<-c(Allosaurus=dTD1, Alxasaurus=dTD2,Aorun=dTD3,
                               Bannykus=dTD4,Beipiaosaurus=dTD5,Eremotherium=dTD6,Erliansaurus=dTD7,
                               Falcarius=dTD8,Guanlong=-dTD9,Haplocheirus=dTD10,
                               Linhenykus=-dTD11,Manis=dTD12,Mononykus=-dTD13,Nothronychus=dTD14,Puma=dTD15,
                               Shishugounykus=dTD16,Tamandua=dTD17,Therizinosaurus=dTD18,
                               Tugulusaurus=dTD19)
write.csv(matrix.distance.twodistance, file = 'matrix.distance.twodistance.csv')
```

## 6.3 Make distance dataset

```
options(scipen = 10000)
distance.HookandPull<-read.csv("matrix.distance.HookandPull.csv",header=T,row.names =1)
distance.Scratchdigging<-read.csv("matrix.distance.Scratch.csv",header=T,row.names =1)
distance.twodistance<-read.csv("matrix.distance.twodistance.csv",header=T,row.names =1)
MWAM<-read.csv("MWAN.csv",header=T,row.names =1)
HP<-distance.HookandPull[,1]
S<-distance.Scratchdigging[,1]
DBD<-distance.twodistance[,1]
Mya<-MWAM[,6]
Clade<-MWAM[,4]
Genus<-row.names(distance.HookandPull)
df.distance<- data.frame(HP,S,DBD,Mya,Clade,Genus)
row.names(df.distance)<-row.names(distance.HookandPull)
df.distance
```

```
##                         HP              S       DBD    Mya             Clade
## Allosaurus       3.2540967  6.84504771183  4.514882 -155.0  NonManirtaptoran
## Alxasaurus       1.0307173  7.91540618686  6.885227 -113.0   Therizinosauria
## Aorun            1.6721670  5.62053158714  5.300444 -166.1 Alvarezsaurioidea
## Bannykus        -3.1274861  4.00290607736  7.127775 -125.0 Alvarezsaurioidea
## Beipiaosaurus   -4.8119130  1.06951704316  5.842826 -125.0   Therizinosauria
## Eremotherium    -3.0204992  0.62171573923  3.345604   -4.9           Mammals
## Erliansaurus    -3.0028848  1.52569691709  3.924232  -85.0   Therizinosauria
## Falcarius       -2.7523864  4.75000435351  7.495996 -129.0   Therizinosauria
## Guanlong        -2.8250120 -2.08810872050 -4.294270 -160.0  NonManirtaptoran
## Haplocheirus    -1.3642107  3.42307597924  4.782339 -166.1 Alvarezsaurioidea
## Linhenykus       1.6862632  0.00003533802 -1.686229  -83.6 Alvarezsaurioidea
## Manis           -3.7706807  2.81282503185  6.583252    0.0           Mammals
## Mononykus        1.0315736  0.00001610996 -1.031558  -72.1 Alvarezsaurioidea
## Nothronychus    -1.1325578  4.73251064548  3.646024  -92.0   Therizinosauria
## Puma            -4.0573384 -1.23170276248  4.324205    0.0           Mammals
## Shishugounykus   0.6579661  3.69681258825  4.050616 -166.1 Alvarezsaurioidea
## Tamandua        -1.3270426  6.68231617036  7.967349    0.0           Mammals
## Therizinosaurus -2.9170911  1.39081517303  3.965694  -72.1   Therizinosauria
## Tugulusaurus    -4.6993735 -0.99782902631  5.278012 -139.8 Alvarezsaurioidea
##                           Genus
## Allosaurus           Allosaurus
## Alxasaurus           Alxasaurus
## Aorun                     Aorun
## Bannykus               Bannykus
## Beipiaosaurus     Beipiaosaurus
## Eremotherium       Eremotherium
## Erliansaurus       Erliansaurus
## Falcarius             Falcarius
## Guanlong               Guanlong
## Haplocheirus       Haplocheirus
## Linhenykus           Linhenykus
## Manis                     Manis
## Mononykus             Mononykus
## Nothronychus       Nothronychus
## Puma                       Puma
## Shishugounykus   Shishugounykus
## Tamandua               Tamandua
## Therizinosaurus Therizinosaurus
## Tugulusaurus       Tugulusaurus
```

## 6.4 Diverging Lollipop Chart of Hook and Pull to piercing

```
df.reorder<-df.distance[order(df.distance[,1]),] ###
df.reorder
```

```
##                         HP              S       DBD    Mya             Clade
## Beipiaosaurus   -4.8119130  1.06951704316  5.842826 -125.0   Therizinosauria
## Tugulusaurus    -4.6993735 -0.99782902631  5.278012 -139.8 Alvarezsaurioidea
## Puma            -4.0573384 -1.23170276248  4.324205    0.0           Mammals
## Manis           -3.7706807  2.81282503185  6.583252    0.0           Mammals
## Bannykus        -3.1274861  4.00290607736  7.127775 -125.0 Alvarezsaurioidea
## Eremotherium    -3.0204992  0.62171573923  3.345604   -4.9           Mammals
## Erliansaurus    -3.0028848  1.52569691709  3.924232  -85.0   Therizinosauria
## Therizinosaurus -2.9170911  1.39081517303  3.965694  -72.1   Therizinosauria
## Guanlong        -2.8250120 -2.08810872050 -4.294270 -160.0  NonManirtaptoran
## Falcarius       -2.7523864  4.75000435351  7.495996 -129.0   Therizinosauria
## Haplocheirus    -1.3642107  3.42307597924  4.782339 -166.1 Alvarezsaurioidea
## Tamandua        -1.3270426  6.68231617036  7.967349    0.0           Mammals
## Nothronychus    -1.1325578  4.73251064548  3.646024  -92.0   Therizinosauria
## Shishugounykus   0.6579661  3.69681258825  4.050616 -166.1 Alvarezsaurioidea
## Alxasaurus       1.0307173  7.91540618686  6.885227 -113.0   Therizinosauria
## Mononykus        1.0315736  0.00001610996 -1.031558  -72.1 Alvarezsaurioidea
## Aorun            1.6721670  5.62053158714  5.300444 -166.1 Alvarezsaurioidea
## Linhenykus       1.6862632  0.00003533802 -1.686229  -83.6 Alvarezsaurioidea
## Allosaurus       3.2540967  6.84504771183  4.514882 -155.0  NonManirtaptoran
##                           Genus
## Beipiaosaurus     Beipiaosaurus
## Tugulusaurus       Tugulusaurus
## Puma                       Puma
## Manis                     Manis
## Bannykus               Bannykus
## Eremotherium       Eremotherium
## Erliansaurus       Erliansaurus
## Therizinosaurus Therizinosaurus
## Guanlong               Guanlong
## Falcarius             Falcarius
## Haplocheirus       Haplocheirus
## Tamandua               Tamandua
## Nothronychus       Nothronychus
## Shishugounykus   Shishugounykus
## Alxasaurus           Alxasaurus
## Mononykus             Mononykus
## Aorun                     Aorun
## Linhenykus           Linhenykus
## Allosaurus           Allosaurus
```

```
df.reorder$Genus <- factor(df.reorder$Genus, levels = c('Shishugounykus', 'Alxasaurus','Mononykus','Nothronychus', 'Tamandua' ,'Haplocheirus','Aorun', 
                                                        'Linhenykus' , 'Falcarius','Guanlong' ,'Therizinosaurus','Erliansaurus', 'Bannykus',
                                                        'Eremotherium','Allosaurus','Manis','Puma','Tugulusaurus','Beipiaosaurus'  ))

g1<-ggplot(df.reorder, aes(x=Genus, y=HP, label=HP))
g1<-g1+ geom_point(stat='identity',  aes(col=Clade), size=6)
g1<-g1+geom_segment(aes(y = 0, x =Genus, yend = HP, xend = Genus))
g1<-g1+theme(axis.text.y = element_text( face = "italic"))
g1<-g1+ theme(legend.direction = 'horizontal', legend.position = 'top')
g1<-g1+ labs(title="Divergence between Hook and Pull and Piercing", 
           subtitle="Negative-to Lower stress VS Postive-to Higher stress")

g1<-g1+ coord_flip()
g1
```

```
ggsave(file='Diverging Lollipop Chart of HP to P.svg', plot=g1,units="mm", width=300, height=300)
ggsave(file='Diverging Lollipop Chart of HP to P.pdf', plot=g1,units="mm", width=300, height=300)
```

## 6.5 Diverging Lollipop Chart of Scratchdigging to Piercing

```
df.reorder<-df.distance[order(df.distance[,2]),] 
df.reorder
```

```
##                         HP              S       DBD    Mya             Clade
## Guanlong        -2.8250120 -2.08810872050 -4.294270 -160.0  NonManirtaptoran
## Puma            -4.0573384 -1.23170276248  4.324205    0.0           Mammals
## Tugulusaurus    -4.6993735 -0.99782902631  5.278012 -139.8 Alvarezsaurioidea
## Mononykus        1.0315736  0.00001610996 -1.031558  -72.1 Alvarezsaurioidea
## Linhenykus       1.6862632  0.00003533802 -1.686229  -83.6 Alvarezsaurioidea
## Eremotherium    -3.0204992  0.62171573923  3.345604   -4.9           Mammals
## Beipiaosaurus   -4.8119130  1.06951704316  5.842826 -125.0   Therizinosauria
## Therizinosaurus -2.9170911  1.39081517303  3.965694  -72.1   Therizinosauria
## Erliansaurus    -3.0028848  1.52569691709  3.924232  -85.0   Therizinosauria
## Manis           -3.7706807  2.81282503185  6.583252    0.0           Mammals
## Haplocheirus    -1.3642107  3.42307597924  4.782339 -166.1 Alvarezsaurioidea
## Shishugounykus   0.6579661  3.69681258825  4.050616 -166.1 Alvarezsaurioidea
## Bannykus        -3.1274861  4.00290607736  7.127775 -125.0 Alvarezsaurioidea
## Nothronychus    -1.1325578  4.73251064548  3.646024  -92.0   Therizinosauria
## Falcarius       -2.7523864  4.75000435351  7.495996 -129.0   Therizinosauria
## Aorun            1.6721670  5.62053158714  5.300444 -166.1 Alvarezsaurioidea
## Tamandua        -1.3270426  6.68231617036  7.967349    0.0           Mammals
## Allosaurus       3.2540967  6.84504771183  4.514882 -155.0  NonManirtaptoran
## Alxasaurus       1.0307173  7.91540618686  6.885227 -113.0   Therizinosauria
##                           Genus
## Guanlong               Guanlong
## Puma                       Puma
## Tugulusaurus       Tugulusaurus
## Mononykus             Mononykus
## Linhenykus           Linhenykus
## Eremotherium       Eremotherium
## Beipiaosaurus     Beipiaosaurus
## Therizinosaurus Therizinosaurus
## Erliansaurus       Erliansaurus
## Manis                     Manis
## Haplocheirus       Haplocheirus
## Shishugounykus   Shishugounykus
## Bannykus               Bannykus
## Nothronychus       Nothronychus
## Falcarius             Falcarius
## Aorun                     Aorun
## Tamandua               Tamandua
## Allosaurus           Allosaurus
## Alxasaurus           Alxasaurus
```

```
df.reorder$Genus <- factor(df.reorder$Genus, levels = c('Mononykus', 'Linhenykus','Eremotherium','Tugulusaurus', 'Beipiaosaurus','Therizinosaurus','Erliansaurus','Puma','Guanlong', 
                                                        'Manis','Haplocheirus' , 'Shishugounykus','Bannykus', 'Falcarius' ,'Nothronychus','Aorun','Tamandua',     
                                                        'Allosaurus','Alxasaurus'))
g2<-ggplot(df.reorder, aes(x=Genus, y=S, label=S))
g2<-g2+ geom_point(stat='identity',  aes(col=Clade), size=6)
g2<-g2+geom_segment(aes(y = 0, x =Genus, yend = S, xend = Genus))
g2<-g2+theme(axis.text.y = element_text( face = "italic"))
g2<-g2+ theme(legend.direction = 'horizontal', legend.position = 'top')
g2<-g2+ labs(title="Divergence between Scratch-digging and Piercing", 
           subtitle="Negative-to Lower stress VS Postive-to Higher stress")
g2<-g2+ coord_flip()
g2
```

```
ggsave(file='Diverging Lollipop Chart of Sd to piercing.svg', plot=g2,units="mm", width=300, height=300)
ggsave(file='Diverging Lollipop Chart of Sd to piercing.pdf', plot=g2,units="mm", width=300, height=300)
```

## 6.6 Diverging Lollipop Chart of Scratchdigging to Hook and Pull

```
df.reorder<-df.distance[order(df.distance[,3]),] 
df.reorder
```

```
##                         HP              S       DBD    Mya             Clade
## Guanlong        -2.8250120 -2.08810872050 -4.294270 -160.0  NonManirtaptoran
## Linhenykus       1.6862632  0.00003533802 -1.686229  -83.6 Alvarezsaurioidea
## Mononykus        1.0315736  0.00001610996 -1.031558  -72.1 Alvarezsaurioidea
## Eremotherium    -3.0204992  0.62171573923  3.345604   -4.9           Mammals
## Nothronychus    -1.1325578  4.73251064548  3.646024  -92.0   Therizinosauria
## Erliansaurus    -3.0028848  1.52569691709  3.924232  -85.0   Therizinosauria
## Therizinosaurus -2.9170911  1.39081517303  3.965694  -72.1   Therizinosauria
## Shishugounykus   0.6579661  3.69681258825  4.050616 -166.1 Alvarezsaurioidea
## Puma            -4.0573384 -1.23170276248  4.324205    0.0           Mammals
## Allosaurus       3.2540967  6.84504771183  4.514882 -155.0  NonManirtaptoran
## Haplocheirus    -1.3642107  3.42307597924  4.782339 -166.1 Alvarezsaurioidea
## Tugulusaurus    -4.6993735 -0.99782902631  5.278012 -139.8 Alvarezsaurioidea
## Aorun            1.6721670  5.62053158714  5.300444 -166.1 Alvarezsaurioidea
## Beipiaosaurus   -4.8119130  1.06951704316  5.842826 -125.0   Therizinosauria
## Manis           -3.7706807  2.81282503185  6.583252    0.0           Mammals
## Alxasaurus       1.0307173  7.91540618686  6.885227 -113.0   Therizinosauria
## Bannykus        -3.1274861  4.00290607736  7.127775 -125.0 Alvarezsaurioidea
## Falcarius       -2.7523864  4.75000435351  7.495996 -129.0   Therizinosauria
## Tamandua        -1.3270426  6.68231617036  7.967349    0.0           Mammals
##                           Genus
## Guanlong               Guanlong
## Linhenykus           Linhenykus
## Mononykus             Mononykus
## Eremotherium       Eremotherium
## Nothronychus       Nothronychus
## Erliansaurus       Erliansaurus
## Therizinosaurus Therizinosaurus
## Shishugounykus   Shishugounykus
## Puma                       Puma
## Allosaurus           Allosaurus
## Haplocheirus       Haplocheirus
## Tugulusaurus       Tugulusaurus
## Aorun                     Aorun
## Beipiaosaurus     Beipiaosaurus
## Manis                     Manis
## Alxasaurus           Alxasaurus
## Bannykus               Bannykus
## Falcarius             Falcarius
## Tamandua               Tamandua
```

```
df.reorder$Genus <- factor(df.reorder$Genus, levels = c('Mononykus', 'Linhenykus','Eremotherium','Nothronychus','Erliansaurus', 'Therizinosaurus','Shishugounykus','Guanlong', 
                                                        'Puma','Allosaurus' , 'Haplocheirus','Aorun', 'Tugulusaurus' ,'Beipiaosaurus','Manis','Alxasaurus','Bannykus',     
                                                        'Falcarius','Tamandua'))
M3<-mean(DBD)
g3<-ggplot(df.reorder, aes(x=Genus, y=DBD, label=DBD))
g3<-g3+ geom_point(stat='identity',  aes(col=Clade), size=6)
g3<-g3+geom_segment(aes(y = 0, x =Genus, yend = DBD, xend = Genus))
g3<-g3+theme(axis.text.y = element_text( face = "italic"))
g3<-g3+ theme(legend.direction = 'horizontal', legend.position = 'top')
g3<-g3+labs(title="Divergence between Scratch-digging and Hook and Pull", 
           subtitle="Absolute distance")
g3<-g3+ coord_flip()
g3
```

```
ggsave(file='Diverging Lollipop Chart of S to H P.svg', plot=g3,units="mm", width=300, height=300)
ggsave(file='Diverging Lollipop Chart of S to H P.pdf', plot=g3,units="mm", width=300, height=300)
```

## 6.7 Combined figures need previous hull figures

```
##need hull code. r#
myplot<-grid.arrange(g1,g2,g3,USEfigure2, ncol=2, nrow=2)
```

```
myplot
```

```
## TableGrob (2 x 2) "arrange": 4 grobs
##   z     cells    name           grob
## 1 1 (1-1,1-1) arrange gtable[layout]
## 2 2 (1-1,2-2) arrange gtable[layout]
## 3 3 (2-2,1-1) arrange gtable[layout]
## 4 4 (2-2,2-2) arrange gtable[layout]
```

```
ggsave(file='Diverging Lollipop Charts.svg', plot=myplot,units="mm", width=700, height=400)
ggsave(file='Diverging Lollipop Charts.pdf', plot=myplot,units="mm", width=700, height=400)

##need hull code. r#
myplot2<-grid.arrange(g1,g2,g3,USEfigure, ncol=2, nrow=2)
```

```
myplot2
```

```
## TableGrob (2 x 2) "arrange": 4 grobs
##   z     cells    name           grob
## 1 1 (1-1,1-1) arrange gtable[layout]
## 2 2 (1-1,2-2) arrange gtable[layout]
## 3 3 (2-2,1-1) arrange gtable[layout]
## 4 4 (2-2,2-2) arrange gtable[layout]
```

```
ggsave(file='Diverging Lollipop Charts with area.svg', plot=myplot2,units="mm", width=700, height=400)
ggsave(file='Diverging Lollipop Charts with area.pdf', plot=myplot2,units="mm", width=700, height=400)
```

## 6.8 Diverging Lollipop Chart of Hook and Pull to piercing in Alvarezsauroids

```
dis.Alvarezsaurioidea<-df.distance %>% filter(Clade == "Alvarezsaurioidea")
dis.Alvarezsaurioidea
```

```
##                        HP              S       DBD    Mya             Clade
## Aorun           1.6721670  5.62053158714  5.300444 -166.1 Alvarezsaurioidea
## Bannykus       -3.1274861  4.00290607736  7.127775 -125.0 Alvarezsaurioidea
## Haplocheirus   -1.3642107  3.42307597924  4.782339 -166.1 Alvarezsaurioidea
## Linhenykus      1.6862632  0.00003533802 -1.686229  -83.6 Alvarezsaurioidea
## Mononykus       1.0315736  0.00001610996 -1.031558  -72.1 Alvarezsaurioidea
## Shishugounykus  0.6579661  3.69681258825  4.050616 -166.1 Alvarezsaurioidea
## Tugulusaurus   -4.6993735 -0.99782902631  5.278012 -139.8 Alvarezsaurioidea
##                         Genus
## Aorun                   Aorun
## Bannykus             Bannykus
## Haplocheirus     Haplocheirus
## Linhenykus         Linhenykus
## Mononykus           Mononykus
## Shishugounykus Shishugounykus
## Tugulusaurus     Tugulusaurus
```

```
df.reorder.age<-dis.Alvarezsaurioidea[order(dis.Alvarezsaurioidea[,4]),] ###
df.reorder.age
```

```
##                        HP              S       DBD    Mya             Clade
## Aorun           1.6721670  5.62053158714  5.300444 -166.1 Alvarezsaurioidea
## Haplocheirus   -1.3642107  3.42307597924  4.782339 -166.1 Alvarezsaurioidea
## Shishugounykus  0.6579661  3.69681258825  4.050616 -166.1 Alvarezsaurioidea
## Tugulusaurus   -4.6993735 -0.99782902631  5.278012 -139.8 Alvarezsaurioidea
## Bannykus       -3.1274861  4.00290607736  7.127775 -125.0 Alvarezsaurioidea
## Linhenykus      1.6862632  0.00003533802 -1.686229  -83.6 Alvarezsaurioidea
## Mononykus       1.0315736  0.00001610996 -1.031558  -72.1 Alvarezsaurioidea
##                         Genus
## Aorun                   Aorun
## Haplocheirus     Haplocheirus
## Shishugounykus Shishugounykus
## Tugulusaurus     Tugulusaurus
## Bannykus             Bannykus
## Linhenykus         Linhenykus
## Mononykus           Mononykus
```

```
df.reorder.age$Genus <- factor(df.reorder.age$Genus, levels = c('Aorun','Haplocheirus', 'Shishugounykus', 'Tugulusaurus',
                                                                'Bannykus','Linhenykus','Mononykus'  ))

g1<-ggplot(df.reorder.age, aes(x=Mya, y=HP, label=HP))
g1<-g1+ geom_point(colour = "blue", size=4)
g1<-g1+ geom_segment(aes(y = 0, x =Mya, yend = HP, xend = Mya))
g1<-g1+geom_text_repel(aes(label = Genus), size = 4)
g1<-g1+theme(axis.text.y = element_text( face = "italic"))
g1<-g1+ theme(legend.direction = 'horizontal', legend.position = 'top')
g1<-g1+ labs(title="Divergence between Hook and Pull and Piercing in Alvarezsauroidea", 
             subtitle="Negative-to Lower stress VS Postive-to Higher stress")
g1<-g1+ coord_flip()
g1
```

```
ggsave(file='Diverging Lollipop Chart of HP-P in Alvare.svg', plot=g1,units="mm", width=300, height=300)
ggsave(file='Diverging Lollipop Chart of HP-P in Alvare.pdf', plot=g1,units="mm", width=300, height=300)
```

## 6.9 Diverging Lollipop Chart of Scratch-digging to Piercing in Alvarezsauroids

```
g2<-ggplot(df.reorder.age, aes(x=Mya, y=S, label=S))
g2<-g2+ geom_point(colour = "blue", size=4)
g2<-g2+geom_segment(aes(y = 0, x =Mya, yend = S, xend = Mya))
g2<-g2+geom_text_repel(aes(label = Genus), size = 4)
g2<-g2+theme(axis.text.y = element_text( face = "italic"))
g2<-g2+ theme(legend.direction = 'horizontal', legend.position = 'top')
g2<-g2+ labs(title="Divergence between Scratch-digging and Piercing in Alvarezsauroidea", 
             subtitle="Negative-to Lower stress VS Postive-to Higher stress")
g2<-g2+ coord_flip()
g2
```

```
ggsave(file='Diverging Lollipop Chart of S-P in Alvare.svg', plot=g2,units="mm", width=300, height=300)
ggsave(file='Diverging Lollipop Chart of S-P in Alvare.pdf', plot=g2,units="mm", width=300, height=300)
```

## 6.10 Diverging Lollipop Chart of Hook and Pull to piercing in Therizinosauria

```
dis.Therizinosauria<-df.distance %>% filter(Clade == "Therizinosauria")
dis.Therizinosauria
```

```
##                        HP        S      DBD    Mya           Clade
## Alxasaurus       1.030717 7.915406 6.885227 -113.0 Therizinosauria
## Beipiaosaurus   -4.811913 1.069517 5.842826 -125.0 Therizinosauria
## Erliansaurus    -3.002885 1.525697 3.924232  -85.0 Therizinosauria
## Falcarius       -2.752386 4.750004 7.495996 -129.0 Therizinosauria
## Nothronychus    -1.132558 4.732511 3.646024  -92.0 Therizinosauria
## Therizinosaurus -2.917091 1.390815 3.965694  -72.1 Therizinosauria
##                           Genus
## Alxasaurus           Alxasaurus
## Beipiaosaurus     Beipiaosaurus
## Erliansaurus       Erliansaurus
## Falcarius             Falcarius
## Nothronychus       Nothronychus
## Therizinosaurus Therizinosaurus
```

```
df.reorder.age<-dis.Therizinosauria[order(dis.Therizinosauria[,4]),] ###
df.reorder.age
```

```
##                        HP        S      DBD    Mya           Clade
## Falcarius       -2.752386 4.750004 7.495996 -129.0 Therizinosauria
## Beipiaosaurus   -4.811913 1.069517 5.842826 -125.0 Therizinosauria
## Alxasaurus       1.030717 7.915406 6.885227 -113.0 Therizinosauria
## Nothronychus    -1.132558 4.732511 3.646024  -92.0 Therizinosauria
## Erliansaurus    -3.002885 1.525697 3.924232  -85.0 Therizinosauria
## Therizinosaurus -2.917091 1.390815 3.965694  -72.1 Therizinosauria
##                           Genus
## Falcarius             Falcarius
## Beipiaosaurus     Beipiaosaurus
## Alxasaurus           Alxasaurus
## Nothronychus       Nothronychus
## Erliansaurus       Erliansaurus
## Therizinosaurus Therizinosaurus
```

```
df.reorder.age$Genus <- factor(df.reorder.age$Genus, levels = c('Falcarius','Beipiaosaurus', 'Alxasaurus', 'Nothronychus',
                                                                'Erliansaurus','Therizinosaurus' ))
g3<-ggplot(df.reorder.age, aes(x=Mya, y=HP, label=HP))
g3<-g3+ geom_point(colour = "red", size=4)
g3<-g3+geom_segment(aes(y = 0, x =Mya, yend = HP, xend = Mya))
g3<-g3+geom_text_repel(aes(label = Genus), size = 4)
g3<-g3+theme(axis.text.y = element_text( face = "italic"))
g3<-g3+ theme(legend.direction = 'horizontal', legend.position = 'top')
g3<-g3+ labs(title="Divergence between Hook and Pull and Piercing in Therizinosauria", 
             subtitle="Negative-to Lower stress VS Postive-to Higher stress")
g3<-g3+ coord_flip()
g3
```

```
ggsave(file='Diverging Lollipop Chart of HP-P in Theri.svg', plot=g3,units="mm", width=300, height=300)
ggsave(file='Diverging Lollipop Chart of HP-P in Theri.pdf', plot=g3,units="mm", width=300, height=300)
```

## 6.11 Diverging Lollipop Chart of Scratch-digging to Piercing in Therizinosauria

```
g4<-ggplot(df.reorder.age, aes(x=Mya, y=S, label=S))
g4<-g4+ geom_point(colour = "red", size=4)
g4<-g4+geom_segment(aes(y = 0, x =Mya, yend = S, xend = Mya))
g4<-g4+geom_text_repel(aes(label = Genus), size = 4)
g4<-g4+theme(axis.text.y = element_text( face = "italic"))
g4<-g4+ theme(legend.direction = 'horizontal', legend.position = 'top')
g4<-g4+ labs(title="Divergence between Scratch-digging and Piercing in Therizinosauria", 
             subtitle="Negative-to Lower stress VS Postive-to Higher stress")
g4<-g4+ coord_flip()
g4
```

```
ggsave(file='Diverging Lollipop Chart of S-P in Theri.svg', plot=g4,units="mm", width=300, height=300)
ggsave(file='Diverging Lollipop Chart of S-P in Theri.pdf', plot=g4,units="mm", width=300, height=300)
```

## 6.12 Combined figure of lollipop figures

```
myplot<-grid.arrange(g1,g2,g3,g4, ncol=2, nrow=2)
```

```
myplot
```

```
## TableGrob (2 x 2) "arrange": 4 grobs
##   z     cells    name           grob
## 1 1 (1-1,1-1) arrange gtable[layout]
## 2 2 (1-1,2-2) arrange gtable[layout]
## 3 3 (2-2,1-1) arrange gtable[layout]
## 4 4 (2-2,2-2) arrange gtable[layout]
```

```
ggsave(file='Diverging Lollipop Charts A VS T.svg', plot=myplot,units="mm", width=600, height=400)
ggsave(file='Diverging Lollipop Charts A VS T.pdf', plot=myplot,units="mm", width=600, height=400)
```
