# Supplementary material for: Functional space analyses reveal the function and evolution of the most bizarre theropod manual unguals
Source: Commun Biol. 2023 Feb 16;6:181. doi: 10.1038/s42003-023-04552-4 (PMC9935540; doi:10.1038/s42003-023-04552-4)
Supplement: Supplementary file 5 — Supplementary Data 2 [file 42003_2023_4552_MOESM5_ESM.zip › 3D shape analyses file/3D MSA meothod_R_markdown.html]

Untitled


# Untitled

#### Zichuan Qin of UoB

#### 2022-02-22

# 1. Packages needed

These are the packages needed in Mophological PCA.

```
# Check whether necessary packages have been installed. If not, these will be installed...
if(!"usethis" %in% installed.packages()) install.packages("usethis")
if(!"devtools" %in% installed.packages()) install.packages("devtools")
if(!"ggplot2" %in% installed.packages()) install.packages("ggplot2")
if(!"plyr" %in% installed.packages()) install.packages("plyr")
if(!"scales" %in% installed.packages()) install.packages("scales")
if(!"grid" %in% installed.packages()) install.packages("grid")
if(!"ggrepel" %in% installed.packages()) install.packages("ggrepel")
if(!"ggbiplot" %in% installed.packages()) install.packages("ggbiplot")
if(!"stats" %in% installed.packages()) install.packages("stats")
if(!"dplyr" %in% installed.packages()) install.packages("dplyr")
if(!"gridExtra" %in% installed.packages()) install.packages("gridExtra")
if(!"svglite" %in% installed.packages()) install.packages("svglite")
if(!"ggthemr" %in% installed.packages()) install.packages("ggthemr")
if(!"tvthemes" %in% installed.packages()) install.packages("tvthemes")
if(!"ggExtra" %in% installed.packages()) install.packages("ggExtra")
if(!"ggridges" %in% installed.packages()) install.packages("ggridges")


# Make the packages available in your R session.
lapply(c("usethis","devtools","ggplot2","plyr","scales","grid","ggrepel","ggbiplot","stats","dplyr",
         "gridExtra","svglite","ggthemr","tvthemes","ggExtra","ggridges"),
       require,                 # Using require instead of library
       character.only = TRUE)   # apply concatenated strings to require function
```

```
## [[1]]
## [1] TRUE
## 
## [[2]]
## [1] TRUE
## 
## [[3]]
## [1] TRUE
## 
## [[4]]
## [1] TRUE
## 
## [[5]]
## [1] TRUE
## 
## [[6]]
## [1] TRUE
## 
## [[7]]
## [1] TRUE
## 
## [[8]]
## [1] TRUE
## 
## [[9]]
## [1] TRUE
## 
## [[10]]
## [1] TRUE
## 
## [[11]]
## [1] TRUE
## 
## [[12]]
## [1] TRUE
## 
## [[13]]
## [1] TRUE
## 
## [[14]]
## [1] TRUE
## 
## [[15]]
## [1] TRUE
## 
## [[16]]
## [1] TRUE
```

# 2. Normal PCA

## 2.1 Reading data

```
Claw<-read.csv('Claw data-noname.csv',header = TRUE,row.names=1)
```

## 2.2 Principal component analysis

```
##PCA results 
Claw.pca<-prcomp(Claw[,c(1:3)],center=TRUE,scale. = TRUE)
summary(Claw.pca)
```

```
## Importance of components:
##                           PC1    PC2     PC3
## Standard deviation     1.3648 1.0473 0.20136
## Proportion of Variance 0.6209 0.3656 0.01351
## Cumulative Proportion  0.6209 0.9865 1.00000
```

```
str(Claw.pca)
```

```
## List of 5
##  $ sdev    : num [1:3] 1.365 1.047 0.201
##  $ rotation: num [1:3, 1:3] 0.504 -0.468 0.726 0.685 0.728 ...
##   ..- attr(*, "dimnames")=List of 2
##   .. ..$ : chr [1:3] "L.W" "L.D" "D.W"
##   .. ..$ : chr [1:3] "PC1" "PC2" "PC3"
##  $ center  : Named num [1:3] 3.8 2.4 1.73
##   ..- attr(*, "names")= chr [1:3] "L.W" "L.D" "D.W"
##  $ scale   : Named num [1:3] 1.388 0.769 0.734
##   ..- attr(*, "names")= chr [1:3] "L.W" "L.D" "D.W"
##  $ x       : num [1:239, 1:3] -0.717 -1.028 -0.934 -1.132 -1.022 ...
##   ..- attr(*, "dimnames")=List of 2
##   .. ..$ : chr [1:239] "1" "2" "3" "4" ...
##   .. ..$ : chr [1:3] "PC1" "PC2" "PC3"
##  - attr(*, "class")= chr "prcomp"
```

```
##Write the PCA results in .csv
write.csv(Claw.pca$x,'Claw.pca$x.csv')

##Plot PCA
ggbiplot(Claw.pca) #Normal plot#
```

```
ggbiplot(Claw.pca, labels=rownames(Claw))  #Plot with labels#
```

## 2.3 Groups Principal component analysis

```
##Groups PCA
group<-read.csv('Group data.csv',header = F) ## import Groups files

Claw.group<-c(group[,c(1)])

ggbiplot(Claw.pca,ellipse=TRUE, groups=Claw.group)
```

# 3. PCA with Hulls

## 3.1 Add a functions of making covex hulls

```
StatBag <- ggproto("Statbag", Stat,
                   compute_group = function(data, scales, prop = 0.5) {
                     
                     #################################
                     #################################
                     # originally from aplpack package, plotting functions removed
                     plothulls_ <- function(x, y, fraction, n.hull = 1,
                                            col.hull, lty.hull, lwd.hull, density=0, ...){
                       # function for data peeling:
                       # x,y : data
                       # fraction.in.inner.hull : max percentage of points within the hull to be drawn
                       # n.hull : number of hulls to be plotted (if there is no fractiion argument)
                       # col.hull, lty.hull, lwd.hull : style of hull line
                       # plotting bits have been removed, BM 160321
                       # pw 130524
                       if(ncol(x) == 2){ y <- x[,2]; x <- x[,1] }
                       n <- length(x)
                       if(!missing(fraction)) { # find special hull
                         n.hull <- 1
                         if(missing(col.hull)) col.hull <- 1
                         if(missing(lty.hull)) lty.hull <- 1
                         if(missing(lwd.hull)) lwd.hull <- 1
                         x.old <- x; y.old <- y
                         idx <- chull(x,y); x.hull <- x[idx]; y.hull <- y[idx]
                         for( i in 1:(length(x)/3)){
                           x <- x[-idx]; y <- y[-idx]
                           if( (length(x)/n) < fraction ){
                             return(cbind(x.hull,y.hull))
                           }
                           idx <- chull(x,y); x.hull <- x[idx]; y.hull <- y[idx];
                         }
                       }
                       if(missing(col.hull)) col.hull <- 1:n.hull
                       if(length(col.hull)) col.hull <- rep(col.hull,n.hull)
                       if(missing(lty.hull)) lty.hull <- 1:n.hull
                       if(length(lty.hull)) lty.hull <- rep(lty.hull,n.hull)
                       if(missing(lwd.hull)) lwd.hull <- 1
                       if(length(lwd.hull)) lwd.hull <- rep(lwd.hull,n.hull)
                       result <- NULL
                       for( i in 1:n.hull){
                         idx <- chull(x,y); x.hull <- x[idx]; y.hull <- y[idx]
                         result <- c(result, list( cbind(x.hull,y.hull) ))
                         x <- x[-idx]; y <- y[-idx]
                         if(0 == length(x)) return(result)
                       }
                       result
                     } # end of definition of plothulls
                     #################################
                     
                     
                     # prepare data to go into function below
                     the_matrix <- matrix(data = c(data$x, data$y), ncol = 2)
                     
                     # get data out of function as df with names
                     setNames(data.frame(plothulls_(the_matrix, fraction = prop)), nm = c("x", "y"))
                     # how can we get the hull and loop vertices passed on also?
                   },
                   
                   required_aes = c("x", "y")
)

#' @inheritParams ggplot2::stat_identity
#' @param prop Proportion of all the points to be included in the bag (default is 0.5)
stat_bag <- function(mapping = NULL, data = NULL, geom = "polygon",
                     position = "identity", na.rm = FALSE, show.legend = NA, 
                     inherit.aes = TRUE, prop = 0.5, alpha = 0.3, ...) {
  layer(
    stat = StatBag, data = data, mapping = mapping, geom = geom, 
    position = position, show.legend = show.legend, inherit.aes = inherit.aes,
    params = list(na.rm = na.rm, prop = prop, alpha = alpha, ...)
  )
}


geom_bag <- function(mapping = NULL, data = NULL,
                     stat = "identity", position = "identity",
                     prop = 0.5, 
                     alpha = 0.3,
                     ...,
                     na.rm = FALSE,
                     show.legend = NA,
                     inherit.aes = TRUE) {
  layer(
    data = data,
    mapping = mapping,
    stat = StatBag,
    geom = GeomBag,
    position = position,
    show.legend = show.legend,
    inherit.aes = inherit.aes,
    params = list(
      na.rm = na.rm,
      alpha = alpha,
      prop = prop,
      ...
    )
  )
}

#' @rdname ggplot2-ggproto
#' @format NULL
#' @usage NULL
#' @export
GeomBag <- ggproto("GeomBag", Geom,
                   draw_group = function(data, panel_scales, coord) {
                     n <- nrow(data)
                     if (n == 1) return(zeroGrob())
                     
                     munched <- coord_munch(coord, data, panel_scales)
                     # Sort by group to make sure that colors, fill, etc. come in same order
                     munched <- munched[order(munched$group), ]
                     
                     # For gpar(), there is one entry per polygon (not one entry per point).
                     # We'll pull the first value from each group, and assume all these values
                     # are the same within each group.
                     first_idx <- !duplicated(munched$group)
                     first_rows <- munched[first_idx, ]
                     
                     ggplot2:::ggname("geom_bag",
                                      grid:::polygonGrob(munched$x, munched$y, default.units = "native",
                                                         id = munched$group,
                                                         gp = grid::gpar(
                                                           col = first_rows$colour,
                                                           fill = alpha(first_rows$fill, first_rows$alpha),
                                                           lwd = first_rows$size * .pt,
                                                           lty = first_rows$linetype
                                                         )
                                      )
                     )
                     
                     
                   },
                   
                   default_aes = aes(colour = "NA", fill = "grey20", size = 0.5, linetype = 1,
                                     alpha = NA, prop = 0.5),
                   
                   handle_na = function(data, params) {
                     data
                   },
                   
                   required_aes = c("x", "y"),
                   
                   draw_key = draw_key_polygon
)

##Add a function of making convex hull#################


###Convex hull PCA###
```

## 3.2 M\_PCA figures with group hulls

```
##PCA wit axes and name

g <- ggbiplot(Claw.pca, obs.scale = 1, var.scale = 1, labels=rownames(Claw),
  groups = Claw.group, ellipse = FALSE, circle = TRUE)
g <- g + scale_color_discrete(name = '')
g <- g + theme(legend.direction = 'horizontal', legend.position = 'top')
g<-g + geom_bag(aes(group = Claw.group, fill = Claw.group), prop = 1) 
g
```

```
ggsave(file='PCA wit axe and name.svg', plot=g,units="mm", width=300, height=200)
ggsave(file='PCA wit axe and name.pdf', plot=g,units="mm", width=300, height=200)

##PCA without axes and name
g <- ggbiplot(Claw.pca, obs.scale = 1, var.scale = 1,alpha = 0,
               ellipse = FALSE, circle = TRUE,var.axes=FALSE)
g<-g+geom_point(aes(color = Claw.group,shape= Claw.group),size=3,alpha = 0.7)
g<-g+scale_shape_manual(values = c(17,16,16,16,15))
g<-g+scale_size_manual(values = c(2,1,1,1,2))
g<-g+geom_bag(aes(group =Claw.group, fill = Claw.group), prop = 1) 
g
```

```
ggsave(file='PCA without axe and name.svg', plot=g,units="mm", width=300, height=200)
ggsave(file='PCA without axe and name.pdf', plot=g,units="mm", width=300, height=200)
```

## 3.3 M\_PCA result analyses

```
PCdata<-read.csv("Claw.pca$x.csv")
PCdata<-cbind(PCdata,group)
PCdata<-PCdata[c(222:228,230:235),]
colnames(PCdata)<-c("Genera" ,  "PC1", "PC2", "PC3" ,"Clade")

plotPC1<-ggplot(PCdata, aes(x = PC1, y = Genera, fill = Clade)) +
  geom_density_ridges(alpha=0.8, stat="binline", bins=30,colour='grey') +
  theme_ridges() + 
  theme(axis.text.y = element_text( face = "italic"))+
  theme(legend.position = "none")+
  xlab('Coordinates on MPC1')+
  ylab('Genera')
plotPC1
```

```
ggsave(file='MPC1 divergence combine figure by clade.svg', plot=plotPC1,units="mm", width=400, height=200)
ggsave(file='MPC1 divergence combine figure by clade.pdf', plot=plotPC1,units="mm", width=400, height=200)

plotPC2<-ggplot(PCdata, aes(x = PC2, y = Genera, fill = Clade)) +
  geom_density_ridges(alpha=0.8, stat="binline", bins=30,colour='grey') +
  theme_ridges() + 
  theme(axis.text.y = element_text( face = "italic"))+
  theme(legend.position = "none")+
  xlab('Coordinates on MPC1')+
  ylab('Genera')
plotPC2
```

```
ggsave(file='MPC2 divergence combine figure by clade.svg', plot=plotPC2,units="mm", width=400, height=200)
ggsave(file='MPC2 divergence combine figure by clade.pdf', plot=plotPC2,units="mm", width=400, height=200)
```
