# Supplementary material for: Functional space analyses reveal the function and evolution of the most bizarre theropod manual unguals
Source: Commun Biol. 2023 Feb 16;6:181. doi: 10.1038/s42003-023-04552-4 (PMC9935540; doi:10.1038/s42003-023-04552-4)
Supplement: Supplementary file 6 — Reporting Summary [file 42003_2023_4552_MOESM6_ESM.pdf]

nature portfolio

Corresponding author(s): Zichuan Qin

Last updated by author(s): Dec 14, 2022

Reporting Summary

Nature Portfolio wishes to improve the reproducibility of the work that we publish. This form provides structure for consistency and transparency in reporting. For further information on Nature Portfolio policies, see our Editorial Policies and the Editorial Policy Checklist.

Statistics

For all statistical analyses, confirm that the following items are present in the figure legend, table legend, main text, or Methods section.

n/a	Confirmed	
☐	☒	The exact sample size (*n*) for each experimental group/condition, given as a discrete number and unit of measurement
☒	☐	A statement on whether measurements were taken from distinct samples or whether the same sample was measured repeatedly
☒	☐	The statistical test(s) used AND whether they are one- or two-sided<br>*Only common tests should be described solely by name; describe more complex techniques in the Methods section.*
☒	☐	A description of all covariates tested
☒	☐	A description of any assumptions or corrections, such as tests of normality and adjustment for multiple comparisons
☒	☐	A full description of the statistical parameters including central tendency (e.g. means) or other basic estimates (e.g. regression coefficient) AND variation (e.g. standard deviation) or associated estimates of uncertainty (e.g. confidence intervals)
☐	☒	For null hypothesis testing, the test statistic (e.g. *F*, *t*, *r*) with confidence intervals, effect sizes, degrees of freedom and *P* value noted<br>*Give P values as exact values whenever suitable.*
☒	☐	For Bayesian analysis, information on the choice of priors and Markov chain Monte Carlo settings
☒	☐	For hierarchical and complex designs, identification of the appropriate level for tests and full reporting of outcomes
☒	☐	Estimates of effect sizes (e.g. Cohen's *d*, Pearson's *r*), indicating how they were calculated

Our web collection on statistics for biologists contains articles on many of the points above.

Software and code

Policy information about availability of computer code

Data collection	The three-dimensional data for unguals of the 19 taxa (Fig. 2; 15 fossil unguals and 4 living animal unguals, details in supplementary Table S2) involved in this research were collected from the following sources:<br>(i) Published three-dimensional data of therizinosaurian unguals, from previous research articles7,12 and personal communications from Stephan Lautenschlager (University of Birmingham, Birmingham, UK), scanned three-dimensional data of Mononykus forelimbs provided by Mark Norell and Congyu Yu (American Museum of Natural History, New York, US).<br>(ii) High-resolution computed tomography and laser scanning of published therizinosaurian, Erliansaurus bellamanus, fossil materials (Institute of Vertebrate Paleontology and Paleoanthropology, Beijing, China). The ungual of IVPP V4025 was scanned by a 225kv (for small skeletal elements) micro-computerized-tomography apparatus at Key Laboratory of Vertebrate Evolution and Human Origins, Chinese Academy of Science (CAS). The ungual of LH V 0002 was scanned by an Artec3D Space Spider surface scanner at the Key Laboratory of Vertebrate Evolution and Human Origins, CAS. The three-dimensional segmentation and rebuilding of these data was performed in Avizo by the author.<br>(iii) Online public 3D content platform Sketchfab (https://sketchfab.com/).<br>All newly scanned tomography slices were cropped and adjusted using the open-source image processing package Fiji to similar size sizes, and then imported into the three-dimensional data visualization and analysis software Avizo 2021.1 (Visualization Science Group) and generated as three-dimensional ungual models.
Data analysis	3D data processing in software Avizo 2021.1 (Visualization Science Group) and Blender (version 3.2.2, Stichting Blender Foundation, http://www.blender.org). Finite-element analysis (FEA) in software Abaqus (version 6.141, Dassault Systemes Simulia Corp). Analysis of FEA outputs, functional-space analysis are accomplished in R, codes are available in supplementary materials.

For manuscripts utilizing custom algorithms or software that are central to the research but not yet described in published literature, software must be made available to editors and reviewers. We strongly encourage code deposition in a community repository (e.g. GitHub). See the Nature Portfolio guidelines for submitting code & software for further information.

Data

Policy information about availability of data

All manuscripts must include a data availability statement. This statement should provide the following information, where applicable:

- Accession codes, unique identifiers, or web links for publicly available datasets
- A description of any restrictions on data availability
- For clinical datasets or third party data, please ensure that the statement adheres to our policy

All data analysed in this paper, including all 3D data, codes and related datasets are available as part of the manuscript or in the supplementary materials.

Human research participants

Policy information about studies involving human research participants and Sex and Gender in Research.

Reporting on sex and gender	*Use the terms sex (biological attribute) and gender (shaped by social and cultural circumstances) carefully in order to avoid confusing both terms. Indicate if findings apply to only one sex or gender; describe whether sex and gender were considered in study design whether sex and/or gender was determined based on self-reporting or assigned and methods used. Provide in the source data disaggregated sex and gender data where this information has been collected, and consent has been obtained for sharing of individual-level data; provide overall numbers in this Reporting Summary. Please state if this information has not been collected. Report sex- and gender-based analyses where performed, justify reasons for lack of sex- and gender-based analysis.*
Population characteristics	*Describe the covariate-relevant population characteristics of the human research participants (e.g. age, genotypic information, past and current diagnosis and treatment categories). If you filled out the behavioural & social sciences study design questions and have nothing to add here, write "See above."*
Recruitment	*Describe how participants were recruited. Outline any potential self-selection bias or other biases that may be present and how these are likely to impact results.*
Ethics oversight	*Identify the organization(s) that approved the study protocol.*

Note that full information on the approval of the study protocol must also be provided in the manuscript.

Field-specific reporting

Please select the one below that is the best fit for your research. If you are not sure, read the appropriate sections before making your selection.

☐ Life sciences ☐ Behavioural & social sciences ☒ Ecological, evolutionary & environmental sciences

For a reference copy of the document with all sections, see nature.com/documents/nr-reporting-summary-flat.pdf

Ecological, evolutionary & environmental sciences study design

All studies must disclose on these points even when the disclosure is negative.

Study description	In this we perform state-of-the-art biomechanical analyses of the most bizarre manual structures from some of the weirdest theropod dinosaurs, the alvarezsauroids and therizinosaurians. Our results surprisingly reveal that the famous elongated, sickle-like claws of Therizinosaurus are decorative structures, but the short, stout claws from Late Cretaceous alvarezsauroids were effective digging tools rather than previously assumed vestigial structures as in T. rex.
Research sample	The three-dimensional data for unguals of the 19 taxa involved in this research.
Sampling strategy	We have collected all 3D data from related fossils.
Data collection	(i) Published three-dimensional data of therizinosaurian unguals, from previous research articles7,12 and personal communications from Stephan Lautenschlager (University of Birmingham, Birmingham, UK), scanned three-dimensional data of Mononykus forelimbs provided by Mark Norell and Congyu Yu (American Museum of Natural History, New York, US).<br>(ii) High-resolution computed tomography and laser scanning of published therizinosaurian, Erliansaurus bellamanus, fossil materials (Institute of Vertebrate Paleontology and Paleoanthropology, Beijing, China). The ungual of IVPP V4025 was scanned by a 225kv (for small skeletal elements) micro-computerized-tomography apparatus at Key Laboratory of Vertebrate Evolution and Human Origins, Chinese Academy of Science (CAS). The ungual of LH V 0002 was scanned by an Artec3D Space Spider surface scanner at the Key Laboratory of Vertebrate Evolution and Human Origins, CAS. The three-dimensional segmentation and rebuilding of these data was performed in Avizo by the author.<br>(iii) Online public 3D content platform Sketchfab (https://sketchfab.com/).

Timing and spatial scale	Data collecting start from 2018/09 to the 2021/10.
Data exclusions	No data excluded.
Reproducibility	The results of the analyses in this study can be reproduced and verified by re-anlyzing the given R code and close functional simulation in any FEA softwares.
Randomization	N/A. Not applicable to this study because there are all 3D claw data we can collected among all related fossil species.
Blinding	N/A. The study is not subject to study bias, and fossil data is very rare, which is not applicable to investigation other fossil materials without preserving claws information.

Did the study involve field work? ☐ Yes ☒ No

Reporting for specific materials, systems and methods

We require information from authors about some types of materials, experimental systems and methods used in many studies. Here, indicate whether each material, system or method listed is relevant to your study. If you are not sure if a list item applies to your research, read the appropriate section before selecting a response.

Materials & experimental systems

n/a	Involved in the study
☒	☐ Antibodies
☒	☐ Eukaryotic cell lines
☐	☒ Palaeontology and archaeology
☒	☐ Animals and other organisms
☒	☐ Clinical data
☒	☐ Dual use research of concern

Methods

n/a	Involved in the study
☒	☐ ChIP-seq
☒	☐ Flow cytometry
☒	☐ MRI-based neuroimaging

Palaeontology and Archaeology

Specimen provenance	(i) Published three-dimensional data of therizinosaurian unguals, from previous research articles7,12 and personal communications from Stephan Lautenschlager (University of Birmingham, Birmingham, UK), scanned three-dimensional data of Mononykus forelimbs provided by Mark Norell and Congyu Yu (American Museum of Natural History, New York, US).<br>(ii) High-resolution computed tomography and laser scanning of published therizinosaurian, Erliansaurus bellamanus, fossil materials (Institute of Vertebrate Paleontology and Paleoanthropology, Beijing, China). The ungual of IVPP V4025 was scanned by a 225kv (for small skeletal elements) micro-computerized-tomography apparatus at Key Laboratory of Vertebrate Evolution and Human Origins, Chinese Academy of Science (CAS). The ungual of LH V 0002 was scanned by an Artec3D Space Spider surface scanner at the Key Laboratory of Vertebrate Evolution and Human Origins, CAS. The three-dimensional segmentation and rebuilding of these data was performed in Avizo by the author.<br>(iii) Online public 3D content platform Sketchfab (https://sketchfab.com/).
Specimen deposition	All data are 3D models from fossil materials.
Dating methods	N/A. Our research based on 3D models of fossil materials. All dating information of these models are based on the dating researches of original fossil materials.

☒ Tick this box to confirm that the raw and calibrated dates are available in the paper or in Supplementary Information.

Ethics oversight	*Identify the organization(s) that approved or provided guidance on the study protocol, OR state that no ethical approval or guidance was required and explain why not.*

Note that full information on the approval of the study protocol must also be provided in the manuscript.
